# Computational Analysis of a Prebiotic Amino Acid Synthesis with Reference to Extant Codon–Amino Acid Relationships

**DOI:** 10.3390/life11121343

**Published:** 2021-12-04

**Authors:** Tolga Yaman, Jeremy N. Harvey

**Affiliations:** Division of Quantum Chemistry and Department of Chemistry, KU Leuven, Celestijnenlaan 200F, B-3001 Leuven, Belgium; tolga.yaman@kuleuven.be

**Keywords:** prebiotic synthesis, computational chemistry, density functional theory, amino acid, metabolism

## Abstract

Novel density functional theory calculations are presented regarding a mechanism for prebiotic amino acid synthesis from alpha-keto acids that was suggested to happen via catalysis by dinucleotide species. Our results were analysed with comparison to the original hypothesis (Copley et al., *PNAS*, **2005**, 102, 4442–4447). It was shown that the keto acid–dinucleotide hypothesis for possible prebiotic amino acid synthesis was plausible based on an initial computational analysis, and details of the structures for the intermediates and transition states showed that there was wide scope for interactions between the keto acid and dinucleotide moieties that could affect the free energy profiles and lead to the required proto-metabolic selectivity.

## 1. Introduction

The question of how life exactly originated on Earth has always been one of the most persistent scientific and philosophical questions. For the modern scientific world, maybe the most important part of the puzzle is abiogenesis, which is the formation of life from inanimate objects. The idea of abiogenesis was considered in seminal papers by Alexander Oparin [1] and John Haldane [2], with their suggestions on the topic being very similar such that the combination of their ideas was named the Oparin–Haldane Hypothesis. This consisted in suggesting that at some time on Earth, probably at very early times, life originated from prebiotic chemical reactions that occurred in a “primordial soup”. From then on, modern scientists have been working experimentally [3] and computationally [4] on the hypothesis. For example, recent years saw a surge of experimental [5,6,7,8,9], computational [10] and combined experimental–computational [11,12,13,14] papers about prebiotic nucleic acids and their synthesis, and the field has largely expanded its knowledge base.

Another field of prebiotic chemistry that is being worked on is prebiotic metabolism. As is known, metabolism is the system that sustains life in all extant living beings. It was also described by Oparin as one of the four so-called barriers between the living and the non-living; as such, its origin is a key to understanding how complex living systems initially arose. At this point, it can be argued that there exist two different points of view about prebiotic chemistry studies [15]: the genetics-first approach and the metabolism-first approach. The genetics-first approach argues that information-carrying systems, such as RNA/DNA, were formed first, possibly from the primordial soup, later paving the way for the formation of other biomolecules and eventually metabolism. Many of the important works that are related to prebiotic nucleic acid synthesis are in line with this idea. On the other hand, the metabolism-first approach argues that there should have existed a very primitive version of metabolism at the very early stages of life based on a set of self-organised reactions, with simple molecules or minerals as catalysts. This proto-metabolism could then lead to the creation of more complex biological molecules. In this vision, the proto-metabolism is thought to already have some resemblance to modern metabolism. Although it has received less attention than the genetics-first approach, the metabolism-first approach has yielded fruitful results in recent years, such as experimentally constructing successful non-enzymatic proto-metabolic cycles in prebiotic conditions [16,17,18,19,20,21,22,23,24,25]. A recent highlight in the field [26] was the discovery of a proto-metabolic system that has similarity to anabolic and catabolic cycles just by having glyoxylate and pyruvate reacting in the presence of iron. There are also computational works [27,28,29] that investigated the plausibility of possible ancient metabolic networks.

As described, studies about prebiotic metabolism have been more about the extant metabolic cycles and their possible prebiotic counterparts. On the other hand, specifically amino acids and their possible prebiotic synthesis have received less attention, although some important work can be highlighted. For example, the Strecker synthesis of amino acids and its possible occurrence under prebiotic conditions has been extensively debated, and it is considered an important candidate for prebiotic amino acid synthesis.

An important study by Parker et al. [30] analysed a reaction mixture that contained cyanamide and basic prebiotic molecules with conditions that were similar to the Miller–Urey experiment. They observed that the reaction mixture yielded amino acids and many dipeptides. The authors suggested that Strecker synthesis intermediates might have played a role in the whole reaction scheme. In his 2016 review [31], Sutherland analysed the idea of a possible cyanosulfidic mechanism that would lead to the prebiotic synthesis of many important biological molecules. In his review, he discussed that such a series of mechanisms could end up with the prebiotic synthesis of many of the precursors that are needed for the Strecker synthesis of amino acids, thus creating a connection between amino acid synthesis and prebiotic nucleotide synthesis.

Apart from the works related to Strecker synthesis, one can consider the work of Huber and Wachtershauser [32], where the authors performed an experiment where a variety of alpha-keto acids simply reacted with a high excess of ammonia in an acidic environment and subsequently reacted with iron as a catalyst and a reducing agent to form amino acids. The Russell group also considered iron systems as important catalysts and studied possible reactions in hydrothermal vent-like environments to yield amino acids [33].

It is well known that amino acids are the building blocks of proteins and proteins are centrally important for life. A recent study related to this connection is about peptide ligation using aminonitriles via the use of a prebiotically available starting species [34]. The study is an important one because how the peptides formed in the prebiotic process is a key question to be answered since extant biochemistry and prebiotic chemistry might use different routes for the synthesis of many molecules. Rather than using amino acids themselves, the authors showed that aminonitriles can be used for this purpose, which was different than what was previously thought.

Another very important field that contributed a lot to the origins of life in general is astrochemistry. The field itself is quite comprehensive and details about it are out of the scope of this study; however, important astrochemistry studies on prebiotic amino acid synthesis using computational methods should be noted [35,36,37].

Surveying the literature, we have come across an intriguing study on possible prebiotic amino acid synthesis by Copley et al. [38]. In their work, they suggest—based on general structural properties but without direct experimental support—a set of prebiotic reaction mechanisms that might have led to amino acid synthesis while also accounting in part for the modern codon relationship between amino acids and the genetic code. In this sense, it is a theory that combines aspects of the metabolism-first approach and the genetics-first approach, though the former aspect outweighs the latter.

Basically, in the authors’ suggested primitive amino acid synthetic route, dinucleotides play a catalytic role that prefigures the modern codon relationship. Because the catalyst species are dinucleotides, the authors suggest that the third base that is present in modern codons was a later addition, and indeed this third basis is associated with considerable redundancy in the modern genetic code (there are only 20 amino acids but 64 different three-base combinations such that many amino acids have multiple different coding XYZ codons, with the same X and Y but different Z; here, X, Y and Z denote individual bases). Overall, the authors suggested that the dinucleotides play a role in the prebiotic synthesis of the same amino acids as would be expected based on the trinucleotide counterparts in modern metabolism do, though, of course, this means that the formation of some amino acids cannot be accounted for. For example, the modern codons GGX all code for glycine; therefore, it is suggested that the GG dinucleotide played a role in the synthesis of glycine.

The authors also suggested that in this prebiotic dinucleotide synthesis route, some of the synthesised amino acids may have differed from the modern amino acids, as this allows for simpler synthetic routes. The ‘ancestral’ amino acids that were suggested in the paper are structurally related to the modern amino acids corresponding to the same dinucleotide proto-codon. For example, modern CGX codes for arginine, a complex amino acid. The authors suggest that the corresponding product from the CG dinucleotide could have been the related simpler species ornithine. Likewise, some of the AGX codons correspond to Ser, while the ancestral AG could lead to 2,4-diaminobutyric acid, and AC could have led to the formation of homoserine (with subsequent developments leading to the synthesis of threonine).

The specific chemistry that was suggested was a very simple form of the modern complex mechanism of translation, which is based, as already mentioned, on catalysis by dinucleotides. By analogy to modern metabolism in which alpha-keto acids commonly act as precursors to amino acids, also here, these species are the source of the carbon atoms in the produced amino acids. The alpha-keto acids are suggested to have formed esters with the 2’ OH group of the first nucleotide in an initial step. The prebiotic availability of alpha-keto acids is considered to be very likely [39,40]. In subsequent steps, these dinucleotide–alpha-keto acid esters would undergo a sequence of additional steps, with prebiotically available reactants, such as ammonia, to form the amino acids.

Showing that this mechanism took place is a very challenging task, as it requires demonstrating that the proposed reactions could have occurred reasonably rapidly, and selectively, under as yet unknown prebiotic reaction conditions. As life certainly did not originate in a single-step procedure, one might note that selectivity might not always be achievable during the initial steps. In some cases, the initial chemistry of the systems might be considered non-selective [41], though this does not change the important fact that issues related to selectivity will need to be addressed eventually. Using computational quantum chemical methods to try to demonstrate the correctness of this model would be even more challenging since it would require showing not only that the proposed reactions would proceed with reasonable kinetics but also that all possible alternative reactions (e.g., those that would lead to non-selective synthesis) are less favored. Of course, another problem with computational work is that it is by its nature approximate; therefore, the predicted kinetic and thermodynamic properties of the proposed reactions are associated with significant error bars, certainly for the complex chemistry that was proposed in the model put forward by Copley et al. However, computational methods can be used to establish in broad terms whether a given reaction could occur, and they yield a large amount of intriguing detailed structural information concerning the intermediates and transition states of the relevant steps that can be of great interest to experimental investigators.

Against that background, we decided to conduct a computational study of the proposal of Copley et al. As already mentioned, the scope of a computational study is intrinsically limited; therefore, we should immediately clarify some assumptions that we made at the outset of our study. To start with, we assumed that the dinucleotide–alpha-keto acid ester mechanism that was suggested by the authors of the paper was credible and that the proposed origin of the selectivity linking particular dinucleotides to particular amino acid products was also broadly plausible. A further assumption needed to be made regarding an initial source of selectivity: the authors do not suggest a detailed mechanism whereby specific keto acids (whose structure will determine which amino acids can be formed) selectively form esters with specific dinucleotides and nor do we. The origin of this selectivity will not be probed here. We also carried on with their assumption that the environment had probably already become chiral such that the dinucleotides are present in a chiral form. With this in hand, our aim was to quantitatively analyse the whole mechanistic suggestions and to check whether these mechanisms were indeed chemically possible.

In terms of calculations, we decided not to consider the cases where uracil is either of the two bases in the dinucleotide, as in those cases, the original paper did not provide complete mechanistic hypotheses. This left the nine dinucleotides shown in Table 1, with the mapping to amino acids also shown there, and we considered the mechanism for these cases.

## 2. Materials and Methods

Calculations were performed using DFT, with the Gaussian 16 program package Rev. A.03 [42]. All intermediate and transition state geometries were optimised with the B3LYP functional, as implemented in Gaussian, together with Grimme’s D3-BJ [43] dispersion correction and the SMD continuum solvation model [44], with parameters that were appropriate for water as the solvent. For all other atoms, the 6–31 g(d) basis set, as implemented in Gaussian, was used. Frequency calculations were carried out for the optimised structures at the same level of theory. All transition states were identified by having one negative eigenvalue in the Hessian matrix. For some transition state structures, the initial transition state search was carried out using the Zimmerman Group’s Growing String Method software [45,46,47]. The geometries that were obtained from the software were further optimised and subjected to frequency calculation at the same level of theory, with a check for the presence of one negative eigenvalue, using the Gaussian 16 package with the methods described above. Based on the masses, rotational constants and vibrational frequencies obtained in this way for each species, free energy corrections at 298 K were calculated using the quasi-harmonic approximation [48] whereby vibrational frequencies with a magnitude smaller than 100 cm^−1^ were set to 100 cm^−1^ for the statistical mechanical treatment. For the translational partition function, a volume corresponding to a standard state concentration of 1 mol dm^−3^ was used for all species, except for the water solvent (55.5 mol dm^−3^). Single-point energies at the optimised structures were calculated also by using the B3LYP functional, the D3-BJ dispersion correction and the SMD continuum model for water but using a larger basis set, namely, the Ahlrichs Def2TZVP basis set [49], as implemented in Gaussian.

## 3. Results

**Figure 1:**
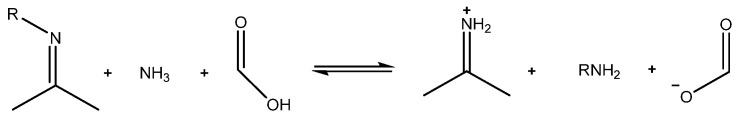
Mechanism of the modified Leuckart reaction, with R being one of the exocyclic bases of the dinucleotides in our study.

Before starting to study the proposed mechanisms, it was necessary to provide additional detail concerning some of the steps that are not fully defined in the initial study [38]. This was particularly so for the reductive amination step whereby a keto group is ultimately transformed into an amino group. This step plays a key role in all the suggested mechanisms, but no specific suggestion was made for the corresponding nature of the reagents that would carry out this step. It was already clear that this step would involve the prior formation of an imine via the condensation of an amine and the ketone. To make an amino acid, this should be an imine with no substituent on nitrogen (RR’C=NH or H-imine) but these are known to be unstable [50]. Therefore, the proposal was that, initially, a more stable substituted imine would be formed via the reaction of a nucleotide NH_2_ group with the ketone group, followed by a reaction with ammonia. The second point is the nature of the reduction step that was suggested to happen immediately after the H-imine formation. The authors did not specify how this reduction would be performed. Our suggestion was that the reducing agent would be formic acid/formate. The use of formate (with ammonia) has been a well-known method to reduce ketones to amines, with a prominent example being the Leuckart reaction [51,52], which is also modelled within the context of computational studies on prebiotic chemistry [53,54,55]. Although the Leuckart reaction is not usually performed with water as the solvent, we considered its mechanism as applicable to our situation (Figure 1). In terms of the availability of formate in prebiotic environments, it is known that formate was successfully synthesised under prebiotic conditions from hydrogen and carbon dioxide in a metabolism-first approach [56]. In addition to that, a recent experimental study [57] concluded that it would have been available under early Earth conditions. Indeed, some other important origin-of-life studies [58,59] also assumed that formic acid was present in prebiotic conditions.

In all our calculations, the starting species were chosen as the starting species of the original mechanistic proposals. We first discuss the cases where guanine is the first base of the nucleotide, i.e., the reactions with GX dinucleotides. Compared with the cases where the first base is either cytosine or adenine, these guanine cases have simpler proposed mechanisms.

### 3.1. GX Cases

**Figure 2:**
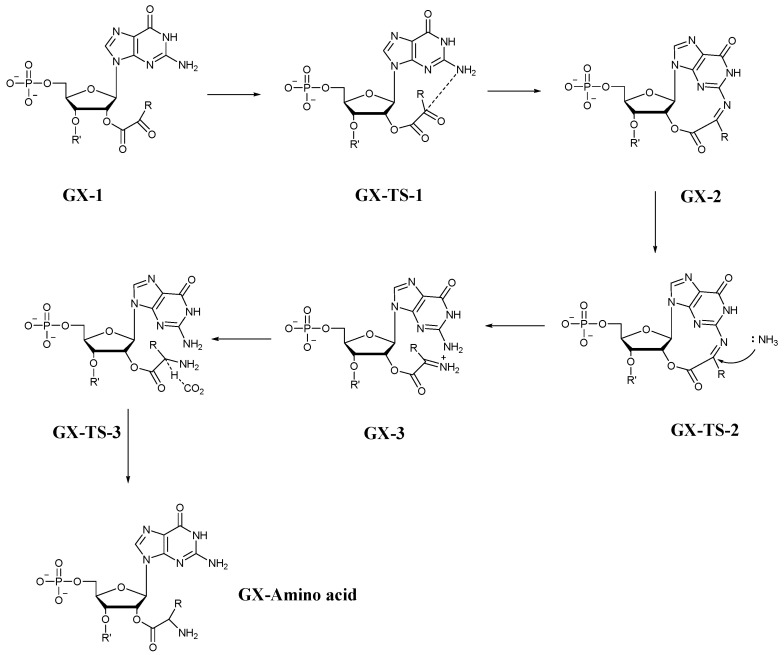
General mechanism for GX cases where R = H for GG, R = CH_3_ for GC and R = CH_2_COO^−^ for GA, while R’ represents the remainder of the dinucleotide.

The general outlook for the mechanism of GX cases is as follows and shown in Figure 2: The starting species **GX-1** underwent an intramolecular nucleophilic attack on the alpha ketone from the exocyclic amine of the first guanine, which was facilitated by an intramolecular hydrogen bond between the exocyclic amine group of the second guanine and the oxygen (not shown in the figure) of the ketone carbonyl group that was attacked. The transition state for this step was referred to as **GX-TS-1**. We modelled this transition state with three external water molecules. A set of preliminary calculations in a model transition state without the second nucleotide showed that the model transition state with three water molecules had lower free energy than with one or two water molecules. A similar test was performed, this time using the full system, and showed that using four explicit water molecules led to a TS with higher free energy than when using three such molecules. Eventually, after the transition state, the R-imine is formed, which is shown here as **GX-2**. The newly formed imine was attacked by ammonia, where the transition state structure of the attack was designated as **GX-TS-2**. This transition state was modelled in our calculation with the inclusion of one explicit water molecule. Afterwards, the reactive intermediate that was formed, namely, **GX-3**, reacted with formate where the hydride of the formate was transferred to the carbon of C=^+^NH_2_ via **GX-TS-3,** successfully forming the final amino acid moiety **GX-Amino acid** and releasing carbon dioxide.

#### 3.1.1. GG Case

**Scheme 1:**
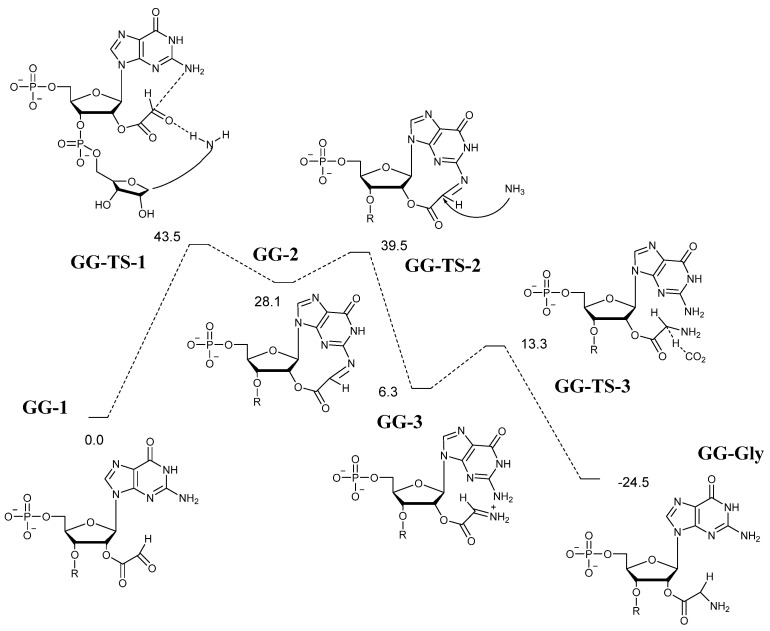
Free energy profile for the GG case. R represents the remainder of the dinucleotide. Relative free energies are in kilocalories per mole.

Out of the three possible cases that we studied for guanine as the first base, we first performed calculations for the GG case, where both bases of the dinucleotide were guanine. The alpha-keto acid that was assumed to be esterified was glyoxylic acid and the amino acid that was supposed to be formed at the end of the mechanism was glycine. In terms of the results, where **GG-1** was taken as the reference point in terms of free energy, **GG-TS-1** lay 43.5 kcal/mol higher than **GG-1**. This was possibly due to the strain on the first guanine, whose exocyclic amine made the attack, as initially, the intramolecular reactants of the initial species were not oriented conveniently for this reaction. The subsequent addition product **GG-2** lay 15.4 kcal/mol lower than **GG-TS-1**. The second transition state **GG-TS-2** lay 10.4 kcal/mol higher than **GG-2**. **GG-3**, which was formed after **GG-TS-2**, lay only 6.3 kcal/mol higher than the initial species. Its subsequent transition state **GG-TS-3** lay 7 kcal/mol higher than **GG-3**, thus 13.3 kcal/mol higher than **GG-1**. The final product with glycine moiety, that is, **GG-Gly**, lay 24.5 kcal/mol lower than the initial species. The free energy profile is shown in Scheme 1.

#### 3.1.2. GC Case

The picture for the GC case is similar. This time, the second base of the dinucleotide was cytosine and the alpha-keto acid that was esterified to the nucleotide was pyruvic acid, with the formed amino acid moiety being alanine. Again, for this case, the starting species **GC-1** was taken as the reference point in terms of free energy. **GC-TS-1** was the transition state for the similar nucleophilic attack of the guanine’s exocyclic amine to the alpha ketone, only this time the interaction of the second base with the oxygen was via the exocyclic amine hydrogens of cytosine. The transition state lay at a similar free energy relative to the reactants as in the GG case at 47.6 kcal/mol. The subsequent addition product **GC-2** lay 20.3 kcal/mol higher than the initial species. The transition state **GC-TS-2** lay 13.4 kcal/mol above **GC-2**. The intermediate **GC-3** lay 3 kcal/mol lower than the starting species and the final transition state **GC-3-TS** lay 11.1 kcal/mol higher than **GC-3**. The final product **GC-Ala** was 24.6 kcal/mol lower than the **GC-1**. The free energy profile can be found in the Supplementary Materials section.

#### 3.1.3. GA Case

The final case for the dinucleotides starting with guanine was the GA case. Here, the second base of the dinucleotide was adenine and the alpha-keto acid that was esterified was oxaloacetic acid. The target amino acid moiety was aspartic acid. It has to be noted that for the case of GA, Copley et al. argued that the target amino acid moiety could have also been glutamic acid if the alpha-keto acid that was esterified was alpha-ketoglutaric acid. However, since the two amino acids are structurally similar, they suggest that mechanisms that can differentiate between them must have been a product of more complex later evolutionary steps [27]. Thus, both versions of the mechanism were actually possible but we chose to model the one that led to aspartic acid. For this part, the first transition state was modelled with four explicit water molecules, as the carboxyl group at the end of the esterified acid chain also interacted with the solvent. Apart from that, the calculations were similar. **GA-TS-1** lay 38.9 kcal/mol higher than **GA-1** in terms of free energy. The addition product **GA-2** lay 25.4 kcal/mol higher than **GA-1**. The free energy of **GA-TS-2** was 10.0 kcal/mol higher than **GA-2**. **GA-3** lay 3.3 kcal/mol lower than **GA-1** and **GA-TS-3** lay 13.8 kcal/mol higher than **GA-3**. The final product **GA-Asp** lay 22.0 kcal/mol lower than **GA-1**. The free energy profile can be found in the Supplementary Materials section.

### 3.2. CX/AX Cases

After finishing with the calculations of the cases where guanine was the first base, the next steps were the cases where cytosine or adenine was the first base of the possible dinucleotides. It was better to analyse both these cases within one framework because the original hypothesis of the authors also described it in this way for practical chemical reasons. They suggested a mechanism for the cases where the first base was C. One can see (Table 1) that the proposed product amino acids in the cases of CX and AX were very similar in structure, with the only difference being that the AX dinucleotides led to amino acid products that had one fewer sp^3^ carbon than in the case of CX dinucleotides. The esterified alpha-keto acid for CX cases was alpha-ketoglutaric acid, whereas, for the AX cases, it was oxaloacetic acid, which has one fewer sp^3^ carbon than alpha-ketoglutaric acid. The main visible difference between the mechanistic suggestions of a specific CX/AX pair can be located in the final steps of the CC and AC routes. Because the target amino acid was proline for CC, there was an additional intramolecular cyclisation step. For the AC case, there was no such step.

#### 3.2.1. CA/AA Cases

After establishing that the CX and AX routes were similar, we could move on to the analysis of the subgroups. Unlike the GX case, the CX and AX cases had intrinsically different mechanistic routes, depending on the second base. This was even more pronounced in the cases where the second base was adenine. For the cases where the second base was guanine or cytosine, the mechanisms were similar in terms of the organic chemistry and the outlook differed only towards the end. Thus, it was better to start with the case where the second base was A. For the cases of CA and AA, starting with species **CA-1**, the hypothesis suggested that one of the terminal carboxylate oxygens of the ketone-bearing ester will initiate a nucleophilic attack towards the diphosphate group, shown here as **CA-TS-1**. This nucleophilic substitution reaction would create the phosphate anhydride **CA-2**. The newly formed phosphate anhydride would then be attacked by ammonia to form an amide, in which the transition state would be **CA-TS-2**. After the formation of amide, **CA-3**, an intramolecular reductive amination process would happen, as in the case of the GX cases, whose transition state is shown as **CA-TS-3**. However, this time, the exocyclic base that attacks the alpha ketone would be the second base, namely, adenine, rather than the first one. As in the cases of GX, the addition product **CA-4** would be attacked by ammonia, as **CA-TS-4** and its product **CA-5** would react with formate to yield the final product **CA-Gln** through **CA-TS-5**. The final products were glutamine and asparagine moieties for CA and AA, respectively. For the case of AA, all the species **CA-n** and **CA-TS-n** could be renamed as **AA-n** and **AA-TS-n**. The final product would be **AA-Asp**.

**Scheme 2:**
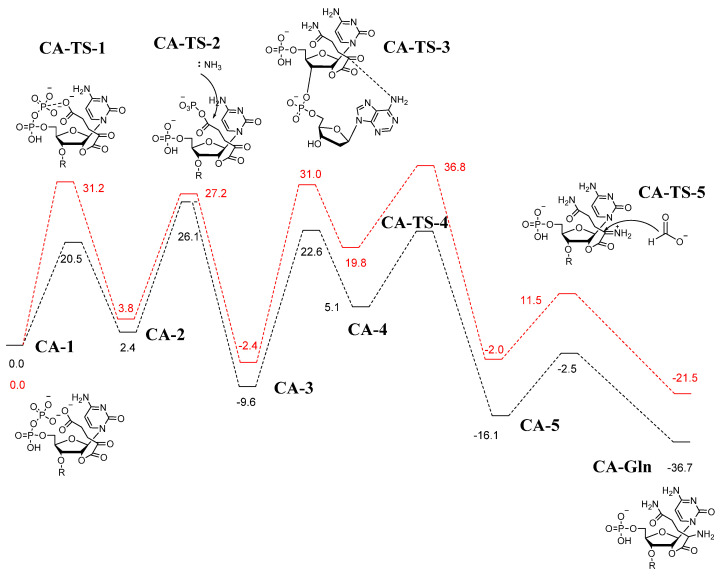
Combined free energy profiles of the CA and AA cases. Black lines correspond to the CA case and red lines correspond to AA. The indicated structures are for CA; for AA, the only change in geometries would be that the first base would be adenine and the keto acid chain has one fewer sp^3^ carbon.

Viewing the results of the computations (Scheme 2), the free energy profiles of the two cases had many similarities. Except for the nucleophilic substitution step, the other steps had very similar transition state barriers. This was especially true for the amidation reaction, which was generally independent of the keto acid chain length and the first base. The AA case had an overall barrier of 36.9 kcal/mol and the CA case had an overall barrier of 26.1 kcal/mol.

#### 3.2.2. Other CX/AX Cases

**Figure 3:**
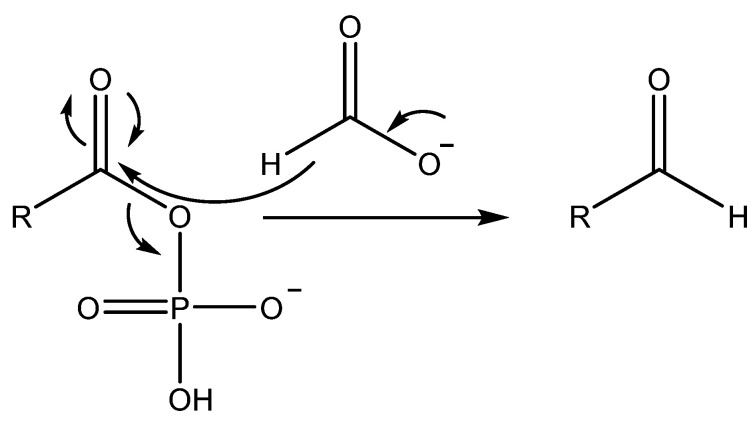
Anhydride to aldehyde reduction using formate.

Next, we moved on to the cases for CC/AC and CG/AG. For these cases, there was a further step whose nature needed to be defined prior to carrying out the computations. A key step in the proposed mechanisms was the reduction of a mixed C,P anhydride to yield an aldehyde. The authors of the original paper did not suggest any specific mechanism for this step, while they did note that NADH (nicotinamide adenine dinucleotide), or a prebiotic version of NADH, would be able to carry out the reduction. Since their (and thus our) fundamental assumption about mechanisms was based on the idea that dinucleotides were present, it is not a priori unreasonable to assume that NADH could be present. On the other hand, from a prebiotic perspective, it seemed more logical to us to suggest a simpler reducing agent, and we accordingly proposed again that formate would act as the reductant. Although formate fit into the conditions and scope of our study, it is definitely worth mentioning that other simple inorganic species, such as some minerals, were also used in origins of life studies as reducing agents [60]. As shown in Figure 3, the anhydride reacted with formate, where the hydride from formate was transferred to the carbonyl carbon while the PO_4_H^−^ group left. We were indeed able to locate transition states for this type of step.

##### CG/AG Cases

After the reduction reaction that formed the terminal aldehyde **CG-2**, which was itself formed after the reaction of **CG-1** with the transition state of **CG-TS-1**, the routes in the CG/AG and CC/AC cases diverged depending on the nature of the second base. For the cases of CG and AG, the target amino acids were ornithine and 2,4-diaminobutyric acid. First, there was a reductive amination on the terminal aldehydic carbon of the keto acid, which was initiated by the nucleophilic attack of the exocyclic amine of the guanine. As in other cases, the formation of this imine was followed by an ammonia attack to form an H-imine and then reduction using formate. These steps were similar in nature to those proposed in other mechanisms discussed here and involved intermediates and TSs that are designated here as **CG-TS-2**, **CG-3**, **CG-TS-3**, **CG-4**, **CG-TS-4** and **CG-5**. After the formation of the terminal amine **CG-5**, this time, there was another reductive amination on the alpha ketone of the keto acid moiety, again by the attack of the exocyclic amine group of guanine to the alpha ketone. Consequently, there was again the same set of reactions that transformed it to an amine, which were the species from **CG-5** to **CG-Orn** with the same mechanistic logic. Eventually, the amino acid moieties were formed. The species in the mechanism for the AG case are labelled in an analogous way, with the eventual product being **AG-Dab**.

**Scheme 3:**
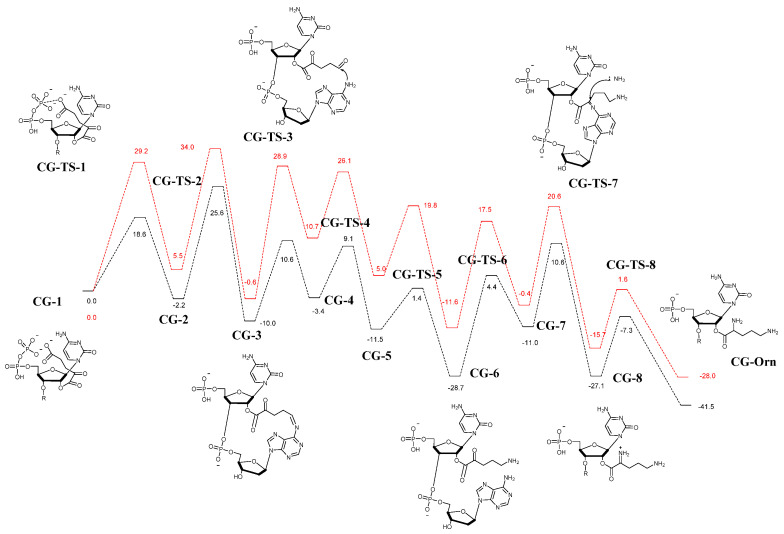
Combined free energy profiles for the CG and AG cases. Black lines correspond to CG and red lines correspond to AG. The geometries are for CG; for AG, the only change in the geometries would be that the first base would be adenine and the keto acid chain has one fewer sp^3^ carbon.

Looking at the resulting energy profile in Scheme 3, the CG and AG cases had milder free energy profiles compared to those found in the GX cases. The highest barriers for the two cases were around 30–35 kcal/mol. Especially for the first exocyclic nucleophilic attack in the CG case, the terminal carbon of the lengthy keto acid chain aligned well with the second base guanine without much strain, leading to a transition state free energy barrier of 20 kcal/mol. In both cases, the free energy barriers for the reduction of the phosphate anhydride were very similar, as expected from the chemistry since neither the first base nor the chain length played a big role in this reduction process. Considering the transition states of the subsequent formate reductions and nucleophilic ammonia attacks on the addition products, it is also visible that the calculated barriers were similar and, hence, the obtained free energy profiles were more or less parallel to each other.

##### CC/AC Cases

For the CC and AC cases, the amino acids to be formed were proline and homoserine. After the aldehyde formation through phosphoryl transfer, via **CC-1**, **CC-TS-1** and **CC-2**, the exocyclic amine of the cytosine in the second base position initiated the reductive amination by attacking the alpha ketone of the keto acid, which was followed by an attack from ammonia and a reduction using formate, which produced the intermediates and the transition states from **CC-2** to **CC-6**. Until that point, the AC mechanism worked the same as the CC mechanism and, indeed, the numbering of the species (see the Supplementary Materials) is analogous. Afterwards, the final stage for the CC and AC mechanisms differed. For the CC case, the amine that was recently formed initiated a nucleophilic attack on the terminal aldehyde, performing a cyclisation reaction, designated as **CC-TS-6**. The resulting cyclic imine **CC-7** could be protonated on its nitrogen centre **CC-8**. Here, the protonation was assumed to be made via a reaction with the formic acid, thus forming formate as a by-product. After this, formate could perform another reduction reaction **CC-TS-8**, this time reducing terminal aldehyde to an alcohol. Thus, the proline moiety **CC-Pro** would be synthesised. For the case of AC, the synthesis of the amino acid moiety ended when the aldehyde was reduced to a secondary alcohol with **AC-TS-6**. Once again, we suggest formate as the reactant for the reduction. After the reduction reaction, the amino acid moiety **AC-Hsr** was formed.

Going over the results for the CC/AC cases, the AC case had an overall barrier of 44 kcal/mol, whereas the CC case had an overall barrier of 37 kcal/mol. The transition state barriers for the phosphoryl transfer in the first step were quite different for the two cases and this situation was the only distinct disparity between similar individual reaction barriers for the CX/AX cases. The rest of the individual step barriers had a quite high similarity to each other, as in the other cases. The fact that the CC and AC mechanisms diverged at the end did not significantly affect the overall barrier since the aldehyde to alcohol reduction in the AC case and the cyclisation process in the CC case had lower barriers than other steps. The free energy profile can be found in the Supplementary Materials section.

## 4. Discussion

When analysed as a whole, the set of calculated free energy pathways corresponding to the suggested dinucleotide–catalysed amino acid syntheses had overall barriers of between 20 and 40 kcal/mol. A free energy barrier of 20 kcal/mol corresponded to a reaction half-life in the order of minutes (depending on the temperature, molecularity and concentrations); therefore, such reactions could clearly account for suggested reactivity. For a barrier of 30 kcal/mol, the expected timescale is of the order of several decades at 25 °C but a year or so at 50 °C; therefore, such reactions could have played a role provided the right temperature and concentration conditions applied. For a barrier of 40 kcal/mol, a timescale of billions of years is expected (though dropping to ‘only’ millions of years at 50 °C); therefore, such reactions cannot be expected to play a role. Another problem with very high barriers is that the products of such reactions possibly will not have half-lives as long as the reactions that formed them; therefore, there will not be a real scenario for those species formed by the high free-energy barrier reaction to be accumulated for possible further steps. Hence, based on our calculations, the steps as we have modelled them here would most likely need to be somewhat adjusted to provide an accurate model of the chemistry.

However, when considering the consequences of free energy barriers to the system kinetics and the associated implications in terms of plausibility of the relevance of the suggested catalysis for proto-metabolism, as previously mentioned, it must be remembered that comparisons need to be drawn cautiously. First, the timescale for the origins of life must have been high; therefore, even relatively slow reactions may be relevant. Here, an important point should be made. The high timescale is described from a chemist’s perspective. It concerns the prebiotic/biochemical reactions in the origins of life context. From a geochemical point of view, such timescales are definitely not high. To illustrate, Miller [61] described a hypothetical origination of life in less than 100 years as “extremely rapid”. At the same time, Russell [62] also described a possible time range for the origination of life, and in that range, geochemically high numbers, such as millions of years, are definitely out of the question. Thus, an average barrier of around 30 kcal/mol is quite reasonable for the feasibility of the hypothesis.

A more important issue is that of computational error. This has several origins. First, the species that were considered here had numerous possible conformational isomers, with each displaying different interatomic interactions. Conformational degrees of freedom and the detailed analysis to choose the best conformer of a system has always been an important aspect of computational studies [63]. However, as the molecules got bigger and bulkier and as the atoms and moieties that made them up were more susceptible to interactions, such as pi-stacking and hydrogen bonding, identifying the conformer with the lowest free energy was very difficult, and we expect that for at least some of the reactions, this was not achieved, which could lead to overestimating (or underestimating) reaction barriers. Other computational aspects, such as the treatment of solvation, the level of theory or the treatment of entropic effects, can also fairly easily affect the calculated free energies by up to 10 kcal/mol. Based on this, although we do not by any means claim that our computations show that the suggested reactions played a role in the origin of life, they are broadly consistent with such a role.

One important thing to be noted here is that in our study, only a rather rough procedure was used to analyse the possible conformations for the different intermediates and TSs. The difficulty in finding the lowest energy conformer for every species had already been described, but one should add the fact that the conformer-related errors might be as large as 5–10 kcal/mol [63]. This means that some barriers in the study would still be relatively high, even without conformer-related errors.

It is also important to underline that surface chemistry must have played a big role in prebiotic chemistry, and thus, in chemical systems like the one we have studied. It is known that adsorption reactions play a key role in establishing high local concentrations of species in the prebiotic era [64]. Some of the questions regarding the eventual selectivity in this study might be explained by such chemical processes. Moreover, surface chemistry might have also been involved in some of the discussed mechanistic steps, such as reduction.

In this context, we should reiterate that a thorough predictive study of possible prebiotic roles for this chemistry would face enormous challenges: we would need to study every single proposed reaction, but also demonstrate that these reactions would be quicker than competing non-selective reactions, including non-catalysed varieties of the reactions we have studied. This goes beyond the scope of our work.

What chemical conclusions can be reached based on our DFT work? We would first like to highlight the fact that our calculations show that some mechanistic suggestions appear to be generally plausible. For example, reaction steps, such as phosphoryl transfers and Leuckart reaction-type reductions, appeared to proceed with quite reasonable barriers. Indeed, going beyond the reductive amination steps, looking at the free energy profiles for the reactions we have studied, formate seemed to be a plausible prebiotic reactant. Here, it must be noted again that several computational studies that include the Leuckart reaction exist in the prebiotic chemistry literature. Our study used formate for all reductive purposes including the Leuckart-type reactions and modelled the system with water as the solvent. Within our calculations, individual reactions with formate as the hydride transfer reactant usually yielded reasonable barriers between 10–25 kcal/mol. This showed that a basic molecule with a high possibility of prebiotic existence can be a key point for the prebiotic amino acid synthesis mechanisms. Furthermore, our results from modelling formate as a general reductant coupled with the new experimental studies regarding the prebiotic availability of formic acid [55,56,57] reaffirmed the idea that prebiotic metabolic processes must be non-enzymatic in the modern sense, like using formate rather than NADH or a similar molecule.

Turning to the comparison of the routes involving the different dinucleotides, some cautious observations can be made. For example, one can observe that all GX cases had similar energy profiles, as might perhaps be expected since the fundamental chemistry was more or less the same. The high barrier corresponding to the exocyclic amine attack transition state was due to the strain associated with the nucleophilic attack of the NH_2_ group on the alpha ketone. As noted above, the raw barriers as calculated here were too high to allow for the steps as modelled to represent an effective prebiotic synthetic route, unless the computational error was significant. It is likely that small modifications of the suggested mechanism might exist and correspond to lower barriers, but such modified mechanisms go beyond the scope of this work. The exocyclic attack transition states did differ in free energy between the three cases by some 5–10 kcal/mol, which can be seen in part as a reflection of computational error (we may have located different conformers for the different cases) or may reflect genuine differences in the secondary interactions in each structure. By and large, though, the intermediates and TSs for the different GX cases had similar relatives free energies compared to the starting products.

For the CX/AX cases, it was generally visible that those steps of mechanisms that involved only a part of the dinucleotide–keto ester species, such as formate reduction, had very similar barriers. This was expected and reassuring given the similar mechanisms suggested for both bases such that we expected the pathways to be similar in terms of free energy profiles. On the other hand, some intramolecular steps had different transition state barriers for the two bases. This was expected, of course, given that the dinucleotides are different from each other. Still, it should be beneficial to analyse the reasons for these disparities. There were two main reasons for this situation, and they were also responsible for some differences in the energy profiles of GX cases, though as those cases were more straightforward and shorter, the differences were not as pronounced as in the other cases.

## 5. Conclusions

We presented new quantum mechanical calculations regarding the suggested dinucleotide-based prebiotic amino acid mechanism in this paper. We first analysed the mechanistic suggestions and elaborated the model to make specific suggestions for some of the co-reactants and mechanisms. For example, we suggest that formate could be a fairly common co-reactant for the required reduction reactions. Our calculations for all of the mechanistic routes yielded a picture where nearly all the systems had a free energy barrier of between 20 and 40 kcal/mol for the proposed synthesis. In terms of kinetics and also in comparison with previous experimental and computational works on prebiotic chemistry and conditions of the prebiotic world, the barriers were indeed reasonable, even after taking into account likely computational errors for these challenging systems. This means that the suggested mechanisms can be considered to indeed be plausible. Our calculations also showed that our suggestion of using formate as a prebiotic reductant for many reduction reactions was a reasonable choice. Another main outcome regarding the calculations was that changes in the bases and the keto acids indeed created differences in terms of the free energy barriers. This became visible in the cases of dual comparisons (same first base and different second base). This hinted that the structure-dependent reactivity that was needed in order to have a selective system to establish some kind of protometabolic genetic code is plausible, even for such simple reactive systems.

On the other hand, the systems offered several big challenges in terms of the calculations. First, the molecules had very high degrees of conformational freedom. This means that in all the calculations, it was very difficult to know the exact lowest energy conformation since many interatomic interactions were possible throughout the transition states and the intermediates. This would surely mean that the calculations had some margins of error that would require a very large amount of work in order to be improved upon. Referring to the discussion related to the possibility of surface-assisted reactions, one might argue that such a scheme could have a positive impact on the system in terms of conformations. Still, computationally detecting the lowest energy conformers would again be quite difficult. Overall, our calculations for the nine cases suggested that the original hypothesis for the prebiotic amino acid synthesis was a reasonable one, though more extensive experimental and computational work is clearly still needed.

## Figures and Tables

**Table 1:** Corresponding pairs of dinucleotides and amino acids that were studied here following the suggestion in [27]. In extant metabolism, 6 of these 9 mappings have remained the same, but in the modern genetic code AGX, ACX and CGX correspond to different amino acids, as explained in the text.

	Adenine	Guanine	Cytosine
Adenine	Asparagine	2,4-Diaminobutyric acid	Homoserine
Guanine	Aspartic acid	Glycine	Alanine
Cytosine	Glutamine	Ornithine	Proline

## Data Availability

All relevant data are included in the Supplementary Materials.

## Supplementary Materials

The following are available online at https://www.mdpi.com/article/10.3390/life11121343/s1, Figures S1–S3: additional free energy profiles, Table S1: Cartesian coordinates and total energies.
