# Supplementary material for: Computational Analysis of a Prebiotic Amino Acid Synthesis with Reference to Extant Codon–Amino Acid Relationships
Source: Life (Basel). 2021 Dec 4;11(12):1343. doi: 10.3390/life11121343 (PMC8707928; doi:10.3390/life11121343)
Supplement: Supplementary file 1 [file life-11-01343-s001.zip › life-1462856-supplementary.pdf]

Computational Analysis of a Prebiotic Amino Acid Synthesis with Reference to Extant Codon-Amino Acid Relationships

Tolga Yaman[1] and Jeremy N. Harvey[1,*]

Supplementary Materials

Content:
A. Figures
B. Optimized Cartesian Coordinates

A. Figures

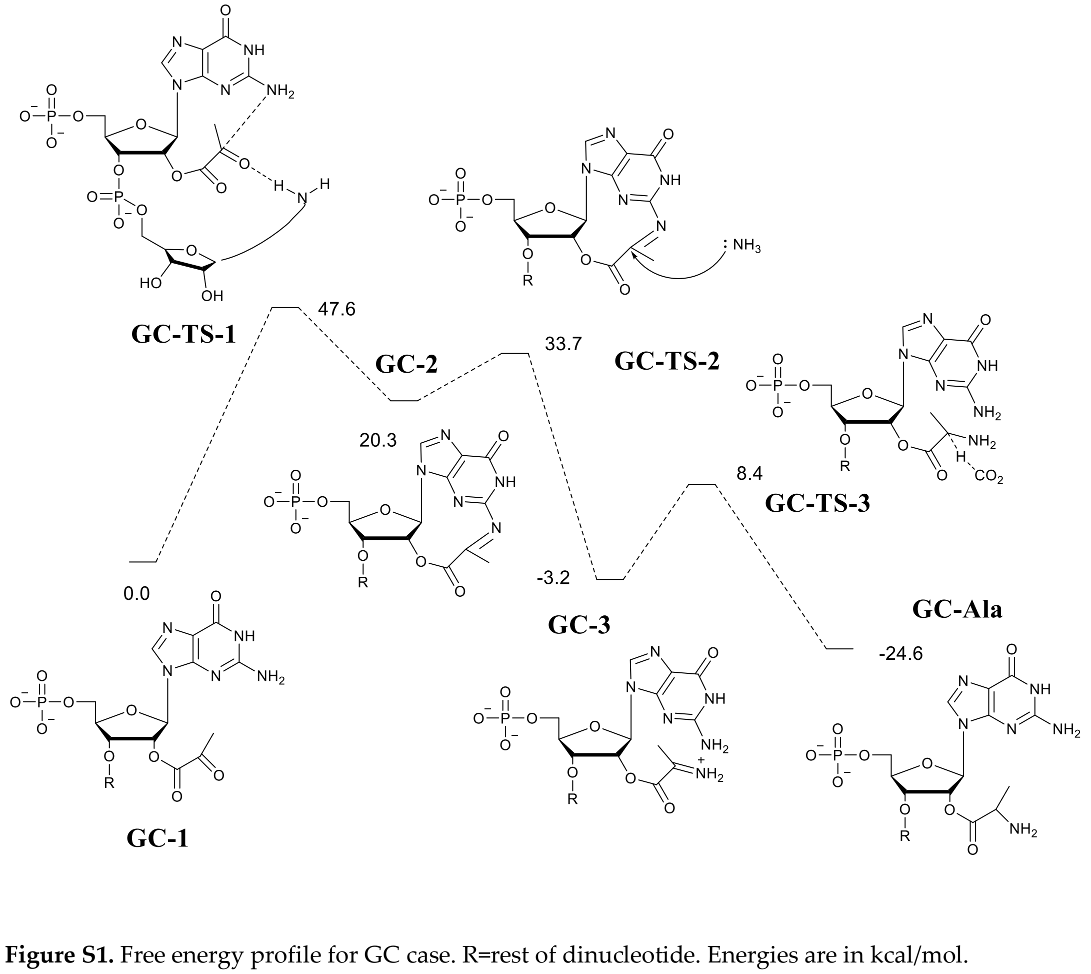

Figure S1. Free energy profile for GC case. R=rest of dinucleotide. Energies are in kcal/mol.

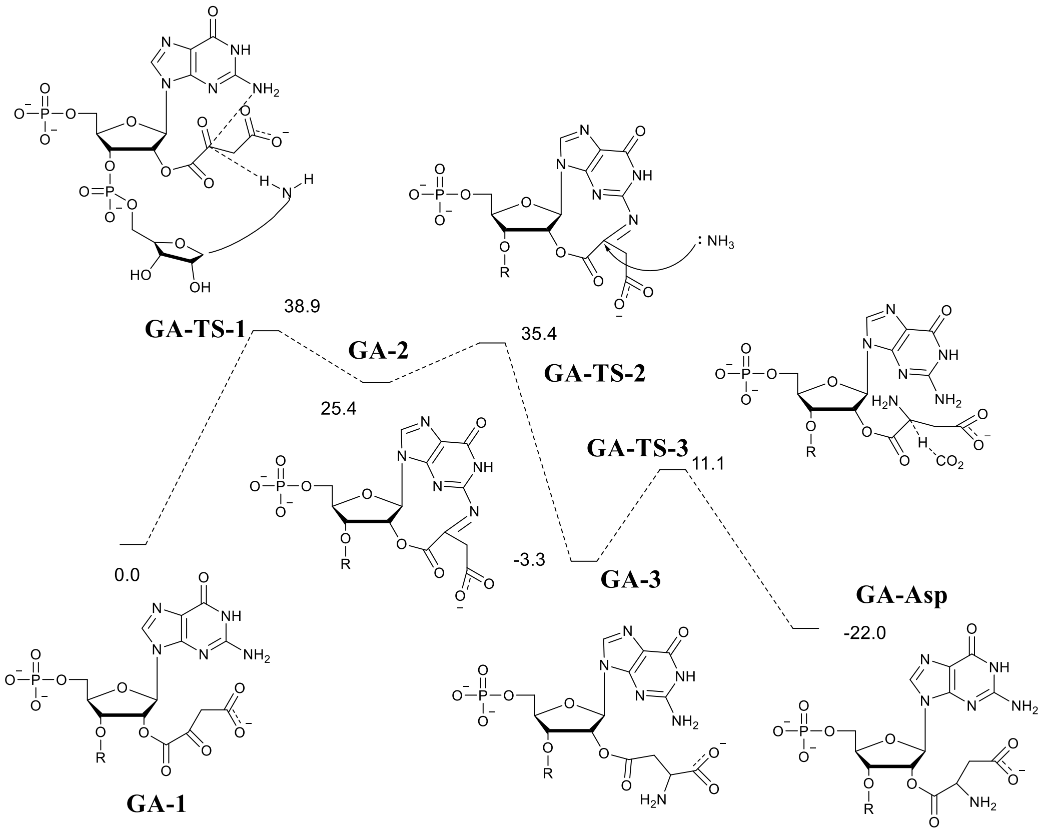

Figure S2. Free energy profile for GA case. R=rest of dinucleotide. Energies are in kcal/mol.

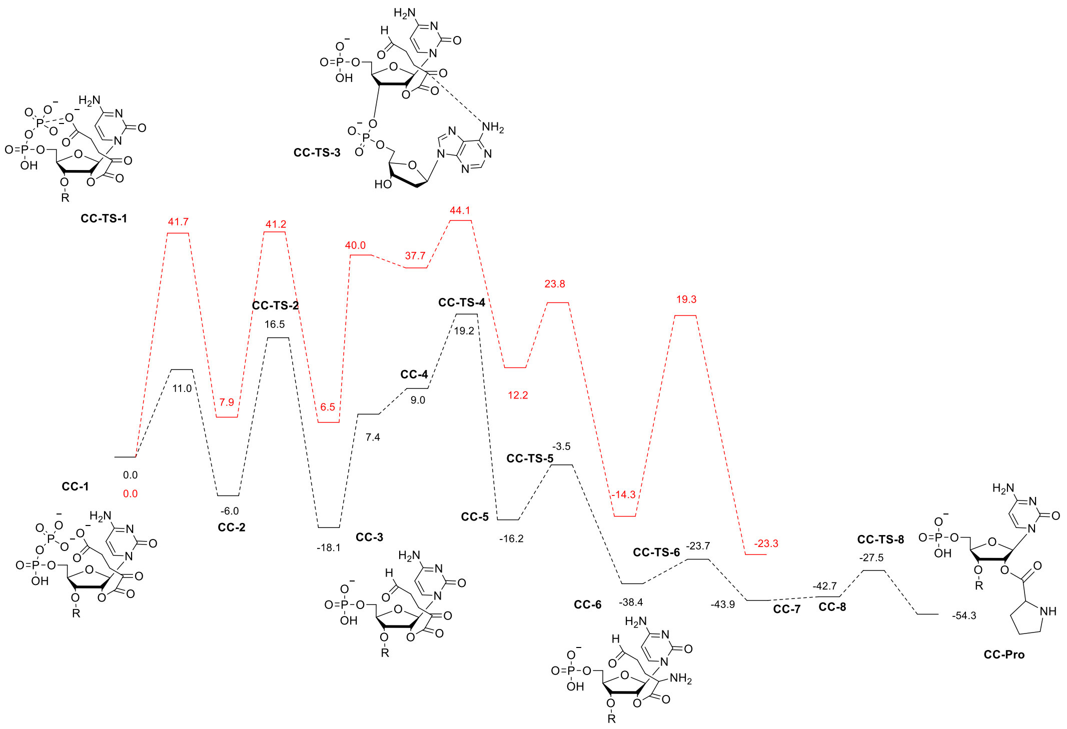

Figure S3. Combined free energy profile of CC and AC cases. Black lines correspond to CC and red lines correspond to AC. Geometries are for CC, for AC the only change in geometries would be that the first base would be adenine and the keto acid chain has one fewer sp³ carbon.

B. Optimized Cartesian Coordinates

Table S1.

GG-1
E: -3591.39195
G: -3590.86048

O	-4.98630	-1.20707	-1.19508
C	-3.69158	-1.61554	-0.78799
H	-3.64399	-2.70876	-0.68151
H	-3.42980	-1.17900	0.18082
C	-2.66642	-1.19959	-1.83393
H	-2.84619	-1.71544	-2.78008
O	-2.76898	0.23730	-2.09466
C	-1.56489	0.86394	-1.70227
H	-0.87147	0.97642	-2.54094
C	-1.22894	-1.43400	-1.35087
H	-1.14964	-2.28704	-0.67918
C	-0.97417	-0.09942	-0.66842
H	-1.52646	-0.03054	0.26825
O	-0.34634	-1.55860	-2.47015
P	0.55582	-2.92731	-2.68139
O	1.08136	-2.82315	-4.08878
O	-0.20359	-4.13117	-2.18869
O	1.78186	-2.71558	-1.60475
C	2.74002	-1.67619	-1.84631
H	2.22735	-0.72385	-2.00285
H	3.33480	-1.90677	-2.73675
C	3.65664	-1.56342	-0.64291
H	4.33936	-0.72776	-0.82138
O	2.89897	-1.25405	0.55327
C	2.76371	-2.40152	1.38352
H	3.23279	-2.18509	2.34530
C	4.44148	-2.82248	-0.28545
H	4.67319	-3.43180	-1.16714
C	3.50169	-3.55748	0.69188
H	2.82483	-4.19977	0.13146
O	5.62902	-2.44763	0.40248
H	5.73118	-3.11635	1.10901
O	0.41068	0.15728	-0.42908
O	4.20895	-4.29895	1.67648
H	4.46023	-5.15021	1.27835
P	-5.96422	-0.53485	0.02986
O	-6.07060	-1.62690	1.10654
O	-5.20374	0.72115	0.50192
O	-7.25399	-0.24757	-0.74911
N	-1.82410	2.19241	-1.18557
C	-0.84843	3.15931	-1.06835
C	-2.88061	2.59130	-0.37750
C	-1.39383	4.12910	-0.23141
H	-3.76486	1.97282	-0.25636
N	-2.66947	3.76108	0.18407
C	-0.57184	5.22906	0.15581
N	0.39690	3.09462	-1.59197
C	1.17180	4.10895	-1.22206
N	0.71516	5.12817	-0.41457
N	2.44466	4.20333	-1.66546
H	3.07874	4.79358	-1.11445
H	2.86661	3.32895	-1.99245
O	-0.84320	6.18136	0.90221
H	1.36857	5.85755	-0.14161
N	1.35451	-2.66137	1.65084
C	0.56869	-1.80047	2.38900
C	0.48230	-3.57871	1.07774
C	-0.73822	-2.25644	2.23575
H	0.82328	-4.37240	0.43480
N	-0.76879	-3.38333	1.42254
C	-1.79739	-1.51285	2.83498
N	1.01009	-0.73379	3.08773
C	0.03031	-0.04525	3.64947
N	-1.28992	-0.41338	3.56316
N	0.32334	1.05429	4.39622
H	-0.42131	1.73125	4.52233
H	1.21749	1.47321	4.16481
O	-3.02043	-1.70704	2.77555
H	-1.99352	0.18709	3.98476
C	0.69612	1.03758	0.53617
C	2.17673	1.38577	0.51929
H	2.73178	1.12051	-0.38503
O	-0.09394	1.56158	1.29227
O	2.65885	2.01694	1.44233
O	4.07915	1.77731	-2.11294
H	4.52858	2.14242	-1.31254
H	4.68058	1.99558	-2.84435
O	5.01107	2.93904	0.23155
H	4.28830	2.61112	0.81386
H	5.83019	2.60106	0.63195
O	4.19644	5.58431	0.21161
H	4.61235	4.69189	0.28837
H	3.51565	5.57122	0.90574

GG-TS-1
E: -3591.32110
G: -3590.79129

O	-5.17196	-0.74476	-0.46922
C	-3.95518	-0.78003	0.25585
H	-3.98793	-1.54214	1.04723
H	-3.75277	0.19031	0.72685
C	-2.84522	-1.13223	-0.72302
H	-3.11970	-2.05025	-1.24394
O	-2.71938	-0.09943	-1.73813
C	-1.59908	0.70799	-1.47254
H	-0.83586	0.57879	-2.24463
C	-1.46452	-1.22117	-0.07044
H	-1.51278	-1.63086	0.94002
C	-1.04804	0.24908	-0.09211
H	-1.52728	0.79842	0.71817
O	0.37493	0.42494	-0.01739
P	-6.42685	0.18974	0.21011
O	-6.53120	-0.28563	1.66757
O	-5.95729	1.64958	0.06309
O	-7.60355	-0.20042	-0.69411
N	-2.01527	2.11937	-1.48888
C	-1.11925	3.12852	-1.25522
C	-3.26542	2.65638	-1.22370
C	-1.85880	4.24024	-0.88068
H	-4.16664	2.06199	-1.26679
N	-3.21391	3.93206	-0.89195
C	-1.15606	5.35555	-0.31405
N	0.21671	2.94599	-1.18118
C	0.81997	3.87252	-0.48904
N	0.22814	5.04974	-0.13797
N	2.06956	3.52489	0.06383
O	-1.59398	6.43998	0.07640
H	0.75623	5.73203	0.40163
C	0.84230	1.42949	0.76329
C	2.25195	1.90879	0.31090
O	0.23205	1.90726	1.69973
O	3.25149	1.75243	1.15397
H	2.16953	3.94451	0.99413
H	3.25402	3.69017	-0.45162
O	4.44409	3.45723	-0.55097
H	4.93329	4.24602	-0.12151

H	4.43504	2.72630	0.13633
O	-0.48018	-1.94089	-0.81143
P	-0.76830	-3.09108	-1.96149
O	-1.38197	-2.44438	-3.17956
O	-1.39159	-4.31620	-1.34298
O	0.80483	-3.45565	-2.24791
C	1.65855	-2.44104	-2.80411
H	1.16101	-1.46804	-2.79611
H	1.89378	-2.70287	-3.84105
C	2.93909	-2.34657	-1.99510
H	3.56741	-1.57013	-2.44371
O	2.63340	-1.94629	-0.63759
C	2.89620	-3.00902	0.27064
H	3.77788	-2.75761	0.86611
C	3.73159	-3.64626	-1.86973
H	3.58341	-4.30844	-2.73106
C	3.18486	-4.25844	-0.56843
H	2.27751	-4.82059	-0.78624
O	5.11062	-3.33804	-1.69572
H	5.43876	-4.00715	-1.06263
O	4.14154	-5.05857	0.11061
H	4.13292	-5.93964	-0.30094
N	1.78800	-3.14966	1.20040
C	1.47923	-2.19414	2.14480
C	0.74525	-4.06641	1.22814
C	0.28309	-2.60964	2.72349
H	0.72000	-4.92375	0.57562
N	-0.15863	-3.79153	2.13880
C	-0.31046	-1.78552	3.72453
N	2.20506	-1.09119	2.41196
C	1.65351	-0.31374	3.33114
N	0.48510	-0.64557	3.97724
N	2.27687	0.84589	3.68092
H	1.68384	1.51968	4.15487
H	2.77041	1.26005	2.87219
O	-1.37612	-1.94528	4.33811
H	0.09478	0.01259	4.64585
O	5.76262	5.48126	0.43960
H	6.48294	5.40961	-0.24312
H	6.17029	5.12509	1.24811
O	7.51587	4.94117	-1.56498
H	7.14637	5.46155	-2.29962
H	7.10097	4.06829	-1.68179
H	2.41321	1.54349	-0.71392

GG-2

E: -3514.89419
G: -3514.38929

O	-4.20190	-3.64953	-0.71943
C	-3.12562	-2.99065	-0.07700
H	-2.52839	-3.69422	0.52038
H	-3.50589	-2.21725	0.60204
C	-2.22162	-2.38604	-1.14498
H	-1.79041	-3.17525	-1.76357
O	-2.97891	-1.53072	-2.05231
C	-2.82939	-0.18196	-1.69142
H	-2.37687	0.39050	-2.50317
C	-1.13369	-1.47298	-0.57796
H	-0.72868	-1.82315	0.37223
C	-1.92498	-0.17806	-0.42753
H	-2.54005	-0.23195	0.46768
O	-1.10557	1.00987	-0.36110
P	-5.49565	-4.11995	0.29428
O	-4.84459	-4.98438	1.38627
O	-6.09751	-2.80266	0.81952
O	-6.37842	-4.88103	-0.70254
N	-4.16088	0.40823	-1.41637
C	-4.23427	1.70067	-0.95725
C	-5.27536	-0.22936	-0.89974
C	-5.38639	1.80474	-0.19666
H	-5.45040	-1.28389	-1.04424
N	-6.05685	0.58250	-0.21674
C	-5.49328	2.92299	0.69902
N	-3.21040	2.56948	-1.04140
C	-3.26104	3.50544	-0.12687
N	-4.35175	3.76391	0.63898
N	-2.04478	4.17101	0.15446
O	-6.38120	3.15650	1.53325
H	-4.26428	4.48462	1.38677
C	-1.38725	1.85883	0.64014
C	-1.09578	3.33864	0.40077
O	-1.89979	1.54720	1.69838
H	-0.08617	3.70426	0.57670
O	1.57155	-0.87074	0.37241
P	1.44969	-0.94824	-1.12713
O	-0.10528	-1.33851	-1.55581
O	2.31986	-1.87292	-1.94624
O	1.58605	0.53716	-1.81484
C	1.78903	1.73043	-1.04713
H	1.19909	2.51809	-1.52454
C	3.24809	2.14376	-1.02760
H	3.30714	3.17789	-0.66871
O	3.97193	1.28965	-0.10339
C	5.17609	0.85306	-0.72084
H	5.98159	1.57914	-0.57430
C	3.97017	2.03150	-2.37934
H	3.26762	1.94289	-3.21490
C	4.83097	0.76567	-2.20767
H	4.23876	-0.12905	-2.41324
O	4.82587	3.15713	-2.55713
H	5.60262	2.80525	-3.03551
O	6.02088	0.79866	-2.97877
H	5.80369	0.47619	-3.86980
N	5.61428	-0.37242	-0.10073
C	6.67182	-0.48961	0.77645
C	5.00600	-1.62122	-0.15319
C	6.65794	-1.81900	1.19279
H	4.13513	-1.80010	-0.77034
N	5.60725	-2.51085	0.60115
C	7.65399	-2.25808	2.11341
N	7.52360	0.49536	1.13513
C	8.45076	0.08922	1.98688
N	8.52631	-1.20080	2.45415
N	9.42026	0.94812	2.39749
H	9.90505	0.73895	3.26242
H	9.21975	1.92885	2.24348
O	7.82139	-3.38161	2.60949
H	9.26923	-1.44342	3.10430
H	1.43839	1.59625	-0.02235
O	-3.83782	5.67937	2.60880
H	-3.44629	5.08933	3.27634
H	-3.05961	6.05698	2.16254
O	-4.10572	-0.24621	2.54269
H	-3.61498	0.53896	2.24891
H	-5.05367	0.02212	2.51373
O	-6.83402	0.44156	2.46575
H	-6.81247	0.19338	1.51386
H	-6.78130	1.41751	2.39768

GG-TS-2

E: -3418.57524
G: -3418.07905

O	-5.51814	-2.91459	0.00855
C	-4.31199	-2.51902	0.63802
H	-3.96862	-3.28134	1.35197
H	-4.44891	-1.57732	1.18389

C	-3.25313	-2.33857	-0.43882
H	-3.11323	-3.27335	-0.98686
O	-3.70788	-1.34522	-1.40435
C	-2.94959	-0.16554	-1.29088
H	-2.26597	-0.05316	-2.13592
C	-1.91449	-1.82326	0.09323
H	-1.70151	-2.16345	1.10802
C	-2.14546	-0.31405	0.02586
H	-2.75049	0.00747	0.87332
O	-0.91480	0.43161	0.00239
P	-6.96133	-2.47342	0.80160
O	-6.85598	-3.10637	2.19920
O	-6.95071	-0.93171	0.81649
O	-8.00699	-3.09933	-0.12945
N	-3.84288	1.00404	-1.30381
C	-3.28871	2.26033	-1.29803
C	-5.10895	1.15582	-0.75244
C	-4.23827	3.12483	-0.78275
H	-5.76250	0.30997	-0.57812
N	-5.39668	2.41214	-0.47063
C	-3.80016	4.44332	-0.40516
N	-1.97527	2.47686	-1.49732
C	-1.54450	3.57713	-0.91555
N	-2.38676	4.57692	-0.52029
N	-0.18566	3.61612	-0.60735
O	-4.46773	5.37637	0.05613
H	-1.95099	5.38764	-0.05905
C	-0.92151	1.62326	0.63337
C	0.17052	2.59097	0.14102
O	-1.74869	1.94012	1.46572
H	1.20044	2.25117	0.15267
O	-0.34733	6.28194	0.53230
H	0.08780	5.48466	0.16106
H	-0.38988	6.09255	1.48487
N	0.56660	3.33932	2.15080
H	0.72621	2.66455	2.89779
H	-0.25540	3.89353	2.38229
H	1.37302	3.95846	2.08596
O	1.28521	-0.99168	-1.40187
P	0.71058	-1.97475	-0.41565
O	-0.88325	-2.24626	-0.80266
O	0.80319	-1.75690	1.07845
O	1.28999	-3.48359	-0.71984
C	2.26602	-3.74087	-1.74053
H	2.10575	-4.77620	-2.05404
C	3.70082	-3.60698	-1.25144
H	4.34681	-4.10324	-1.98505
O	4.08122	-2.20781	-1.18229
C	4.69617	-1.94234	0.07256
H	5.77536	-2.11511	0.02119
C	3.97180	-4.19612	0.14043
H	3.17561	-4.87589	0.46276
C	4.06195	-2.94601	1.03592
H	3.06153	-2.62322	1.33176
O	5.23279	-4.85978	0.13775
H	5.60060	-4.69808	1.02925
O	4.90935	-3.13274	2.15828
H	4.38313	-3.55830	2.85633
N	4.52273	-0.54829	0.41141
C	5.45531	0.44276	0.17826
C	3.38180	0.09071	0.88467
C	4.83140	1.63152	0.54927
H	2.48317	-0.45827	1.13051
N	3.53617	1.38959	0.99079
C	5.55355	2.85419	0.42908
N	6.70308	0.26656	-0.30771
C	7.37725	1.39988	-0.41865
N	6.85148	2.62261	-0.07624
N	8.67003	1.38417	-0.83681
H	9.05664	2.23952	-1.21931
H	8.95954	0.53501	-1.30680
O	5.18950	4.00774	0.69968
H	7.42836	3.45191	-0.18896
H	2.10789	-3.08344	-2.59923

GG-3

E: -3342.64080
G: -3342.15333

O	4.03707	4.01604	-0.83692
C	2.97618	3.36009	-0.16580
H	2.18118	4.06930	0.10551
H	3.33262	2.88143	0.75451
C	2.39488	2.30828	-1.09691
H	2.02942	2.77494	-2.01424
O	3.44207	1.36608	-1.48487
C	3.19200	0.10814	-0.90448
H	2.80946	-0.60477	-1.64149
C	1.28882	1.46381	-0.45105
H	0.70962	2.01926	0.28675
C	2.13164	0.35837	0.17299
H	2.58711	0.69601	1.10337
O	1.41653	-0.86832	0.39521
P	5.30977	4.57742	0.14771
O	4.64317	5.50825	1.17327
O	5.91957	3.30567	0.77286
O	6.19819	5.27087	-0.89194
N	4.41872	-0.45299	-0.35766
C	4.61896	-1.78397	-0.06671
C	5.47337	0.24490	0.21140
C	5.82271	-1.82455	0.63204
H	5.54387	1.32653	0.15694
N	6.34532	-0.55032	0.79299
C	6.32129	-3.09088	1.06588
N	3.79119	-2.81509	-0.38011
C	4.25665	-3.99314	0.02474
N	5.44672	-4.13597	0.68999
N	3.51722	-5.11031	-0.16370
O	7.35740	-3.34903	1.68851
H	5.74107	-5.06854	0.96877
C	1.86643	-1.64726	1.37706
C	1.40420	-3.05845	1.17051
O	2.57752	-1.33374	2.30791
O	-1.46033	0.83816	0.27014
P	-1.16958	0.69712	-1.20113
O	0.45164	0.93725	-1.47668
O	-1.90585	1.51776	-2.23349
O	-1.30040	-0.86027	-1.69921
C	-1.46998	-1.94849	-0.78180
H	-0.87046	-2.78052	-1.16339
C	-2.92091	-2.38547	-0.70532
H	-2.96183	-3.35138	-0.18877
O	-3.67755	-1.41477	0.06287
C	-4.85883	-1.06811	-0.64987
H	-5.67764	-1.75180	-0.40664
C	-3.62167	-2.50269	-2.06669
H	-2.90626	-2.55406	-2.89475
C	-4.48233	-1.22724	-2.12326
H	-3.88299	-0.37995	-2.46466
O	-4.47489	-3.64399	-2.06602
H	-5.24495	-3.37975	-2.60746
O	-5.65740	-1.38786	-2.90054
H	-5.41716	-1.24362	-3.83151
N	-5.29613	0.24770	-0.25334
C	-6.33825	0.51374	0.61056
C	-4.71941	1.47420	-0.56213
C	-6.34731	1.89875	0.75945
H	-3.86352	1.54982	-1.21886
N	-5.32579	2.48200	0.01897
C	-7.33980	2.49097	1.59376

N	-7.15927	-0.39837	1.17498
C	-8.08009	0.14904	1.95155
N	-8.17911	1.50417	2.15573
N	-9.01977	-0.63065	2.54757
H	-9.49641	-0.26167	3.36237
H	-8.79752	-1.61783	2.58880
O	-7.53028	3.68736	1.85620
H	-8.91621	1.85593	2.76057
H	-1.11203	-1.67965	0.21419
H	2.76323	-5.03932	-0.83629
H	0.88020	-3.35521	0.27021
N	1.64201	-3.91596	2.08868
H	1.38368	-4.89680	1.97863
H	2.14685	-3.63647	2.93457
H	3.96542	-6.01575	-0.08829

GG-TS-3

E: -3531.95011
G: -3531.44561

O	3.20935	4.52054	-0.27967
C	2.36072	3.53627	0.28492
H	1.45892	3.99187	0.71861
H	2.87971	2.98690	1.07964
C	1.94049	2.57882	-0.81939
H	1.40810	3.12191	-1.60364
O	3.12276	1.98719	-1.43759
C	3.20304	0.61695	-1.11330
H	2.92468	-0.01393	-1.96251
C	1.09812	1.39525	-0.32856
H	0.45240	1.65002	0.51202
C	2.20486	0.41475	0.03642
H	2.65628	0.68802	0.98994
O	1.76564	-0.95141	0.08588
P	4.38722	5.18232	0.75792
O	3.59669	5.79713	1.92341
O	5.27016	3.98736	1.17393
O	5.06573	6.18792	-0.18042
N	4.56612	0.26917	-0.74956
C	5.07285	-1.00993	-0.78635
C	5.44528	1.02495	0.01477
C	6.26598	-0.95208	-0.07036
H	5.27218	2.07735	0.21511
N	6.48668	0.33060	0.41659
C	7.01531	-2.15353	0.10199
N	4.48685	-2.08206	-1.36885
C	5.17584	-3.19988	-1.19811
N	6.36832	-3.24641	-0.51801
N	4.68895	-4.37747	-1.66242
O	8.08523	-2.32931	0.70082
H	6.83835	-4.14203	-0.41779
C	2.46126	-1.76616	0.88714
C	2.07227	-3.19494	0.67054
O	3.31553	-1.44179	1.68762
H	3.94179	-4.30452	-2.34165
H	1.41612	-3.47103	-0.14602
N	2.81239	-4.09997	1.24019
H	2.64918	-5.08913	1.08094
H	3.48545	-3.82827	1.95420
H	5.33594	-5.14471	-1.80077
C	0.27236	-3.37994	2.75709
H	0.73313	-3.02265	1.72940
O	0.98527	-4.22076	3.32268
O	-0.81072	-2.83320	3.02593
O	-1.78886	1.16795	0.02216
P	-1.28437	0.56757	-1.26471
O	0.33432	0.88668	-1.42118
O	-1.89214	0.91366	-2.60152
O	-1.29295	-1.07188	-1.19971
C	-1.43122	-1.78202	0.03832
H	-0.75658	-2.63887	-0.01017
C	-2.84542	-2.30067	0.21829
H	-2.84451	-3.00141	1.06003
O	-3.74133	-1.20141	0.52977
C	-4.86958	-1.25445	-0.33427
H	-5.66175	-1.87508	0.09513
C	-3.42071	-2.98577	-1.02902
H	-2.63624	-3.28264	-1.73401
C	-4.35057	-1.90862	-1.61462
H	-3.77786	-1.19500	-2.21187
O	-4.20033	-4.11280	-0.63825
H	-4.93871	-4.14021	-1.27863
O	-5.43869	-2.45343	-2.34339
H	-5.12671	-2.63894	-3.24526
N	-5.43032	0.06594	-0.48767
C	-6.56520	0.52333	0.14927
C	-4.91274	1.14767	-1.19011
C	-6.68207	1.86075	-0.22180
H	-4.00794	1.06635	-1.77513
N	-5.63763	2.23330	-1.06006
C	-7.78314	2.61615	0.27786
N	-7.37336	-0.19996	0.95433
C	-8.39723	0.49827	1.41682
N	-8.60354	1.82105	1.10806
N	-9.33732	-0.10364	2.19204
H	-9.90516	0.48497	2.79074
H	-9.05327	-0.98379	2.60499
O	-8.07306	3.80434	0.07656
H	-9.41398	2.29411	1.49923
H	-1.14603	-1.15655	0.88534

GG-Gly

E: -3343.41967
G: -3342.92290

O	3.23098	4.28267	-0.58316
C	2.37958	3.36369	0.07909
H	1.47659	3.86458	0.45687
H	2.89298	2.90344	0.93177
C	1.96196	2.28957	-0.91231
H	1.43318	2.74130	-1.75502
O	3.14329	1.62758	-1.45342
C	3.21545	0.30062	-0.97415
H	2.93456	-0.42001	-1.74765
C	1.11825	1.17606	-0.28492
H	0.47572	1.53077	0.52130
C	2.21770	0.23604	0.19228
H	2.67891	0.62911	1.09843
O	1.75809	-1.09639	0.42573
P	4.41475	5.03133	0.38686
O	3.63321	5.70627	1.52531
O	5.32470	3.87898	0.86023
O	5.06022	5.98631	-0.62494
N	4.57894	-0.00857	-0.57895
C	5.08583	-1.28519	-0.50084
C	5.44764	0.80938	0.13219
C	6.26887	-1.16778	0.22572
H	5.27808	1.87743	0.23405
N	6.48306	0.15190	0.60590
C	7.01119	-2.35056	0.51311
N	4.51401	-2.40037	-1.00844
C	5.19341	-3.49961	-0.72836
N	6.37085	-3.49159	-0.02052
N	4.71200	-4.71242	-1.10643
O	8.07191	-2.47712	1.14220
H	6.83463	-4.37650	0.16621
C	2.43910	-1.82001	1.34992
C	1.89676	-3.22114	1.45678
O	3.34960	-1.37284	2.02123
H	3.98912	-4.68639	-1.81493

H	1.74380	-3.60794	0.44554
N	2.82766	-4.07885	2.18563
H	2.38117	-4.98756	2.29824
H	2.93029	-3.70335	3.12781
H	5.36752	-5.48091	-1.18629
O	-1.76855	0.95758	0.12458
P	-1.26849	0.23822	-1.10167
O	0.34330	0.55012	-1.30934
O	-1.89149	0.44796	-2.46061
O	-1.26214	-1.38813	-0.87138
C	-1.45761	-1.98308	0.41817
H	-0.78869	-2.84496	0.47096
C	-2.88411	-2.47147	0.59186
H	-2.91992	-3.11611	1.47766
O	-3.77188	-1.34326	0.80351
C	-4.88463	-1.44768	-0.07676
H	-5.69045	-2.03003	0.37989
C	-3.43689	-3.23511	-0.62033
H	-2.63942	-3.58252	-1.28625
C	-4.34709	-2.19463	-1.29756
H	-3.75750	-1.53026	-1.93345
O	-4.23240	-4.32895	-0.17192
H	-4.95280	-4.39915	-0.82932
O	-5.42561	-2.78270	-2.00675
H	-5.10541	-3.01249	-2.89532
N	-5.42736	-0.13586	-0.33230
C	-6.55501	0.38555	0.26740
C	-4.88729	0.88528	-1.10552
C	-6.64596	1.69594	-0.19588
H	-3.98228	0.74609	-1.67986
N	-5.59253	1.99022	-1.05390
C	-7.73154	2.50558	0.24963
N	-7.37807	-0.26515	1.11783
C	-8.38946	0.48222	1.52833
N	-8.56866	1.78515	1.13012
N	-9.34203	-0.04903	2.33910
H	-9.90471	0.58824	2.89085
H	-9.07543	-0.90421	2.81160
O	-7.99661	3.68363	-0.03013
H	-9.36853	2.30104	1.48694
H	-1.19749	-1.28415	1.21651
H	0.89737	-3.13254	1.91289

GC-1

E: -3483.07549
G: -3482.53346

O	4.47424	-3.72920	0.35803
C	3.21750	-3.31963	-0.14798
H	2.65460	-4.17156	-0.55574
H	3.34735	-2.58398	-0.95274
C	2.41538	-2.70627	0.98983
H	2.23022	-3.45119	1.76702
O	3.17872	-1.63242	1.61241
C	2.63588	-0.37979	1.27262
H	2.20052	0.10989	2.14743
C	1.10010	-2.08435	0.50782
C	1.55672	-0.65230	0.21550
H	1.98164	-0.59872	-0.78647
O	0.52783	0.33138	0.35379
P	5.70077	-3.96652	-0.80212
O	5.13096	-5.01652	-1.76992
O	5.91617	-2.58342	-1.44741
O	6.85369	-4.44649	0.08843
N	3.66827	0.50927	0.74075
C	3.46260	1.86199	0.54409
C	4.67821	0.13523	-0.13930
C	4.39593	2.23812	-0.41974
H	5.02292	-0.88772	-0.21145
N	5.15604	1.14723	-0.82595
C	4.37899	3.58240	-0.89791
N	2.52122	2.62300	1.14929
C	2.50868	3.87868	0.71518
N	3.37462	4.33397	-0.25725
N	1.67714	4.79611	1.24959
O	5.09298	4.11097	-1.76333
H	3.29882	5.30299	-0.55408
C	0.42655	1.27325	-0.58658
C	-0.57072	2.37185	-0.17268
O	1.04208	1.31734	-1.63070
O	-0.53081	3.39887	-0.83705
H	1.43283	5.60313	0.66530
H	0.91524	4.45819	1.84191
O	0.71696	6.75429	-0.69259
H	-0.17818	6.53505	-0.34327
H	0.85879	6.06650	-1.36540
C	-1.47552	2.12895	0.98345
H	-2.20297	2.93626	1.06853
H	-1.97389	1.16249	0.87499
H	-0.87516	2.07205	1.89858
O	-1.57920	5.64500	0.42609
H	-2.51244	5.80790	0.20660
H	-1.34410	4.82670	-0.06875
O	-0.75298	4.65008	2.89324
H	-1.16502	5.01911	2.07675
H	-0.39383	5.43466	3.34034
O	-1.99315	-0.91713	2.25907
P	-1.45649	-1.83111	1.18725
O	0.13323	-2.13932	1.55439
O	-1.55867	-1.49654	-0.28091
O	-2.03532	-3.35880	1.42094
C	-3.38454	-3.55465	1.87047
H	-3.54380	-3.02505	2.81540
H	-3.47829	-4.62839	2.05027
C	-4.46214	-3.15249	0.87811
H	-5.40974	-3.58440	1.23733
O	-4.58077	-1.72441	0.81131
C	-5.33940	-1.40369	-0.35932
H	-6.39443	-1.31481	-0.10275
C	-4.25221	-3.56610	-0.58583
H	-3.21172	-3.39862	-0.86520
C	-5.17162	-2.57828	-1.34692
H	-4.75049	-2.27413	-2.30727
O	-4.55243	-4.92323	-0.84574
H	-5.52592	-4.98378	-0.82302
O	-6.46555	-3.15007	-1.54661
H	-6.42633	-3.67427	-2.36423
C	-3.74512	0.06648	-1.50586
C	-3.28085	1.29941	-1.82664
C	-4.05094	2.41636	-1.37632
N	-4.92397	-0.09239	-0.83176
C	-5.66079	1.04337	-0.39243
N	-5.19094	2.28052	-0.69849
H	-2.34926	1.42691	-2.36335
H	-3.19448	-0.83136	-1.74085
O	-6.70639	0.86894	0.25533
N	-3.63363	3.67137	-1.66505
H	-2.66034	3.80383	-1.91184
H	-4.06834	4.43309	-1.15742
H	0.72152	-2.57560	-0.38927

GC-TS-1

E: -3483.00444
G: -3482.46342

O	-4.99823	-3.26726	-0.10850
C	-3.88653	-2.72454	0.58167
H	-3.55151	-3.39488	1.38582
H	-4.14338	-1.75706	1.03351
C	-2.75912	-2.56088	-0.42551

H	-2.53320	-3.52752	-0.88046
O	-3.16858	-1.68452	-1.51496
C	-2.65613	-0.39115	-1.31838
H	-2.01552	-0.10333	-2.15503
C	-1.48919	-1.91872	0.12885
C	-1.83532	-0.43111	0.00190
H	-2.44962	-0.12173	0.84825
O	-0.69096	0.42812	-0.09554
P	-6.50581	-3.19781	0.68630
O	-6.26333	-3.85888	2.05355
O	-6.84609	-1.69869	0.77146
O	-7.37874	-4.00225	-0.28511
N	-3.76608	0.58051	-1.25712
C	-3.50837	1.90711	-1.03029
C	-5.07663	0.38095	-0.85225
C	-4.67297	2.46353	-0.52565
H	-5.53470	-0.59650	-0.85881
N	-5.66308	1.49034	-0.44557
C	-4.59023	3.77340	0.05538
N	-2.27115	2.44182	-1.07573
C	-2.15949	3.53777	-0.38139
N	-3.23778	4.22889	0.09544
N	-0.86686	3.90826	0.04063
O	-5.48136	4.46618	0.55285
H	-3.08422	5.07987	0.63137
C	-0.62115	1.49688	0.73264
C	0.26064	2.67298	0.18840
O	-1.19271	1.56039	1.80304
O	1.11547	3.14076	1.08634
H	-0.93328	4.23139	1.01267
H	-0.03088	4.85227	-0.40408
O	1.04831	5.35391	-0.40495
H	1.08999	6.25303	0.07198
H	1.41919	4.68548	0.25506
C	0.83333	2.38542	-1.19811
H	1.41690	3.24195	-1.54050
H	1.50274	1.52414	-1.11556
H	0.06066	2.15640	-1.93113
O	1.68107	7.57309	0.80990
H	2.60602	7.28108	0.61029
H	1.57001	7.36568	1.75420
O	3.93418	6.22540	-0.07935
H	4.48180	6.76685	-0.67365
H	3.29907	5.79498	-0.67990
O	1.77551	-1.15171	-1.39878
P	1.15745	-2.00130	-0.31771
O	-0.41991	-2.28277	-0.74385
O	1.20374	-1.61146	1.14040
O	1.71695	-3.54597	-0.42143
C	2.72425	-3.97153	-1.34884
H	2.58015	-3.49246	-2.32155
H	2.57733	-5.04849	-1.46334
C	4.15228	-3.73859	-0.87067
H	4.80588	-4.42543	-1.42918
O	4.55087	-2.38537	-1.13392
C	5.57846	-1.98517	-0.22313
H	6.54376	-1.94168	-0.72506
C	4.39225	-3.92875	0.63417
H	3.53057	-3.55496	1.19016
C	5.62679	-3.03786	0.90159
H	5.60430	-2.58014	1.89360
O	4.57537	-5.27798	1.01757
H	5.44689	-5.53571	0.66422
O	6.82894	-3.79025	0.73029
H	7.00053	-4.25414	1.56714
C	4.08560	-0.40110	0.84174
C	3.61825	0.85087	1.03327
C	4.40812	1.92367	0.50425
N	5.27677	-0.62661	0.20743
C	6.04665	0.45889	-0.28650
N	5.58348	1.72308	-0.10180
H	2.66826	1.03221	1.51706
H	3.53014	-1.26855	1.16675
O	7.11790	0.21905	-0.87385
N	3.94338	3.17684	0.64741
H	2.93973	3.27630	0.84044
H	4.41089	3.94164	0.17394
H	-1.27005	-2.20695	1.15800

GC-2

E: -3406.58905
G: -3406.07603

O	3.62402	3.60312	-0.83172
C	2.57111	2.92886	-0.16747
H	1.99639	3.61925	0.46652
H	2.97736	2.14527	0.48384
C	1.61795	2.34506	-1.20503
H	1.13384	3.14589	-1.76652
O	2.32082	1.53411	-2.19273
C	2.25641	0.17497	-1.84521
H	1.79604	-0.40790	-2.64498
C	0.59000	1.39031	-0.59855
H	0.24268	1.70057	0.38726
C	1.41843	0.10914	-0.53517
H	2.08167	0.16241	0.32501
O	0.64566	-1.11239	-0.44152
P	4.91484	4.11704	0.16792
O	4.26033	5.03458	1.21348
O	5.51017	2.82539	0.75917
O	5.80580	4.82668	-0.85904
N	3.63395	-0.34030	-1.64177
C	3.80728	-1.59710	-1.12102
C	4.75668	0.37342	-1.25945
C	5.02086	-1.60691	-0.45384
H	4.86534	1.42100	-1.49052
N	5.62866	-0.36048	-0.59805
C	5.25359	-2.67174	0.48232
N	2.82784	-2.51755	-1.08939
C	2.99241	-3.40387	-0.14087
N	4.15390	-3.56253	0.55191
N	1.86407	-4.14882	0.24393
O	6.21693	-2.82687	1.24944
H	4.15104	-4.26919	1.30884
C	0.96336	-1.90042	0.60367
C	0.81423	-3.42515	0.46138
O	1.41033	-1.49555	1.65937
O	-2.02614	0.89022	0.55541
P	-2.04643	1.04047	-0.94397
O	-0.49956	1.24927	-1.50569
O	-2.89446	2.09379	-1.61642
O	-2.43270	-0.36919	-1.68766
C	-2.48193	-1.61085	-0.97322
H	-2.00386	-2.36480	-1.60516
C	-3.91480	-2.02366	-0.70133
H	-3.91354	-3.06100	-0.34596
O	-4.46713	-1.17288	0.33427
C	-5.76788	-0.74745	-0.06280
H	-6.52269	-1.48607	0.20819
C	-4.85619	-1.90490	-1.91148
H	-4.30446	-1.80149	-2.85232
C	-5.68801	-0.64860	-1.58632
H	-5.15536	0.25041	-1.90625
O	-5.71835	-3.03866	-1.95848
H	-6.57662	-2.68589	-2.26761
O	-6.99802	-0.70085	-2.13007
H	-6.94366	-0.42556	-3.06089
H	-1.93564	-1.53749	-0.03214
O	3.39019	-5.58478	2.35412
H	3.11225	-5.03064	3.10404

H	2.61107	-5.56370	1.76504
O	3.81753	0.15272	2.32727
H	3.46394	-0.66860	1.95107
H	4.79519	0.07395	2.22656
O	6.60366	-0.07912	2.03452
H	6.48500	0.18770	1.09689
H	6.62740	-1.05557	1.94684
C	-0.50011	-4.03660	0.79044
H	-0.93317	-3.55818	1.67571
H	-1.19525	-3.86896	-0.04091
H	-0.38963	-5.11041	0.95357
C	-7.09159	0.39126	1.68955
C	-5.41727	1.62049	0.43983
C	-5.67402	2.73657	1.16209
H	-4.66221	1.60803	-0.33585
C	-6.69109	2.64081	2.16408
H	-5.12982	3.65604	0.98753
O	-7.67230	-0.68803	1.89973
N	-7.36112	1.51162	2.40671
N	-7.00386	3.72634	2.89280
H	-6.47166	4.58033	2.80098
H	-7.67332	3.64665	3.64636
N	-6.11565	0.46328	0.65647

GC-TS-2

E: -3310.27037
G: -3309.76292

O	-4.30605	-3.69763	0.25238
C	-3.15117	-3.01103	0.70258
H	-2.55102	-3.64467	1.37104
H	-3.42709	-2.10228	1.25243
C	-2.31149	-2.63739	-0.50835
H	-2.05179	-3.53379	-1.07648
O	-3.09272	-1.78375	-1.39654
C	-2.59715	-0.46697	-1.37537
H	-2.08469	-0.22414	-2.30992
C	-1.05375	-1.84150	-0.15283
H	-0.65596	-2.10098	0.82957
C	-1.59921	-0.41617	-0.19209
H	-2.12938	-0.19585	0.73397
O	-0.56762	0.56307	-0.39959
P	-5.68650	-3.55645	1.24346
O	-5.21983	-3.99766	2.64058
O	-6.07905	-2.06811	1.16183
O	-6.65400	-4.51658	0.54069
N	-3.71049	0.48772	-1.23585
C	-3.44012	1.83447	-1.25839
C	-4.90427	0.36156	-0.53623
C	-4.48485	2.47212	-0.61240
H	-5.33812	-0.60511	-0.31278
N	-5.41744	1.52547	-0.18621
C	-4.30240	3.85689	-0.26433
N	-2.24547	2.33603	-1.61942
C	-1.98926	3.51015	-1.07038
N	-2.98157	4.30003	-0.55359
N	-0.65776	3.86179	-0.95318
O	-5.09659	4.62258	0.29643
H	-2.68338	5.18683	-0.12874
C	-0.71694	1.73021	0.25800
C	0.07053	2.90924	-0.36891
O	-1.43758	1.86732	1.22787
O	-1.17738	6.38009	0.29824
H	-1.09743	6.14438	1.23797
H	-0.69856	5.65261	-0.16098
C	1.48683	2.65475	-0.78937
H	2.01784	2.03720	-0.06334
H	1.46153	2.11478	-1.74327
H	2.01595	3.59892	-0.93925
N	0.58650	3.68180	1.52993
H	-0.29025	3.96617	1.96139
H	1.16478	4.50421	1.36477
H	1.07926	3.04650	2.15681
O	1.80223	-0.56544	-2.09018
P	1.48270	-1.50719	-0.95647
O	-0.07165	-2.06186	-1.16511
O	1.66715	-1.10537	0.48727
O	2.23362	-2.95458	-1.20753
C	3.36688	-3.05249	-2.08040
H	3.41684	-4.09623	-2.40305
C	4.67423	-2.69474	-1.39712
H	5.49320	-2.94364	-2.08311
O	4.69870	-1.27046	-1.12868
C	5.24738	-1.06505	0.16903
H	6.34026	-1.09877	0.12420
C	4.92364	-3.39338	-0.04996
H	4.22050	-4.21574	0.12289
C	4.72954	-2.25630	0.97031
H	3.66394	-2.14398	1.17862
O	6.26760	-3.86272	0.00840
H	6.53296	-3.74409	0.94187
O	5.49850	-2.42761	2.14977
H	5.00324	-3.01847	2.74240
H	3.22320	-2.41769	-2.95866
C	5.51181	1.36184	0.00677
C	4.01530	0.46951	1.68426
C	3.72616	1.72476	2.11003
H	3.58436	-0.40120	2.15199
C	4.35141	2.80827	1.41760
H	3.04734	1.89490	2.93620
O	6.32248	1.12601	-0.90420
N	5.20657	2.62132	0.41072
N	4.10591	4.07586	1.80618
H	3.35682	4.27125	2.45644
H	4.45400	4.83680	1.23785
N	4.88057	0.26080	0.64807

GC-3

E: -3234.34653
G: -3233.84809

O	-4.27417	-3.55556	-0.01710
C	-3.13705	-2.93680	0.55724
H	-2.57818	-3.64209	1.18868
H	-3.43254	-2.08350	1.18126
C	-2.23350	-2.46646	-0.57077
H	-1.94944	-3.31315	-1.19945
O	-2.94986	-1.53005	-1.42940
C	-2.49917	-0.21518	-1.20604
H	-1.97155	0.17220	-2.08184
C	-0.98977	-1.71653	-0.08639
H	-0.62666	-2.07287	0.87872
C	-1.54869	-0.29445	-0.00283
H	-2.09208	-0.15097	0.93046
O	-0.53260	0.70878	-0.12771
P	-5.66954	-3.60176	0.96168
O	-5.21633	-4.27495	2.26718
O	-6.08352	-2.12740	1.13482
O	-6.61574	-4.43307	0.08659
N	-3.62625	0.67334	-0.94161
C	-3.51817	2.04515	-0.90515
C	-4.82022	0.35248	-0.31015
C	-4.68744	2.49108	-0.29391
H	-5.13638	-0.67235	-0.15793
N	-5.49363	1.41802	0.06571
C	-4.84317	3.89132	-0.06628
N	-2.46177	2.77082	-1.33953
C	-2.58779	4.06397	-1.09290
N	-3.70998	4.60739	-0.51598
N	-1.56028	4.90945	-1.36216

O	-5.78648	4.49848	0.45897
H	-3.73728	5.60940	-0.34731
C	-0.54419	1.72914	0.71440
C	0.62883	2.65758	0.44068
O	-1.32214	1.93432	1.62173
C	1.40100	2.59815	-0.80750
H	1.83429	1.59416	-0.92207
H	0.70827	2.72156	-1.64884
H	2.17490	3.36560	-0.83664
O	1.92213	-0.32836	-1.87546
P	1.57136	-1.31415	-0.78850
O	0.01867	-1.83238	-1.08534
O	1.70407	-0.97283	0.67514
O	2.34641	-2.74242	-1.05514
C	3.39562	-2.84856	-2.02776
H	3.42015	-3.89542	-2.34282
C	4.75649	-2.47838	-1.46451
H	5.51170	-2.72162	-2.22132
O	4.79577	-1.05191	-1.20475
C	5.36344	-0.82267	0.07639
H	6.45498	-0.77822	0.00716
C	5.12892	-3.16540	-0.14338
H	4.48411	-4.02508	0.07118
C	4.93972	-2.04269	0.89083
H	3.88121	-1.97788	1.15107
O	6.49758	-3.55853	-0.17676
H	6.81367	-3.44609	0.74109
O	5.76840	-2.18528	2.03219
H	5.32807	-2.80174	2.64216
H	3.17303	-2.22268	-2.89547
C	5.36418	1.62182	-0.12683
C	4.14621	0.60922	1.69816
C	3.77284	1.83681	2.14572
H	3.87121	-0.29226	2.22240
C	4.20042	2.95854	1.38115
H	3.17023	1.94855	3.03744
O	6.11567	1.46361	-1.09952
N	4.96221	2.85266	0.30030
N	3.76354	4.21166	1.73513
H	3.55960	4.35094	2.72044
H	4.26092	4.98277	1.30104
N	4.91077	0.47541	0.57791
H	-1.76529	5.89431	-1.48325
N	0.88465	3.48096	1.39252
H	0.31142	3.47241	2.23871
H	1.72356	4.08183	1.36184
H	-0.83805	4.53429	-1.96495

GC-TS-3

E: -3423.64782
G: -3423.13483

O	3.88168	3.99187	0.31444
C	2.91035	3.15927	0.92200
H	2.28087	3.72855	1.62087
H	3.39146	2.34918	1.48364
C	2.02426	2.57793	-0.16881
H	1.52189	3.38062	-0.71342
O	2.83620	1.85173	-1.13725
C	2.62536	0.46279	-1.00635
H	2.04766	0.06700	-1.84640
C	1.00365	1.56490	0.35527
H	0.65002	1.79653	1.36082
C	1.83813	0.28730	0.30160
H	2.51546	0.23878	1.15410
O	1.03444	-0.89840	0.25988
P	5.33907	4.17868	1.17623
O	4.91568	4.68694	2.56398
O	5.97177	2.77138	1.20084
O	6.07405	5.19666	0.29638
N	3.90100	-0.23471	-0.99539
C	4.05791	-1.56412	-1.31465
C	5.05838	0.15543	-0.33327
C	5.33073	-1.90315	-0.86134
H	5.19729	1.16462	0.04188
N	5.94443	-0.81270	-0.25699
C	5.77285	-3.25022	-1.01639
N	3.13658	-2.35364	-1.91260
C	3.53891	-3.60736	-2.04076
N	4.77777	-4.04075	-1.63404
N	2.69980	-4.54411	-2.55281
O	6.84963	-3.76408	-0.68183
H	5.01457	-5.02186	-1.75539
C	1.56458	-2.01170	0.77672
C	0.64395	-3.20373	0.59357
O	2.62956	-2.09018	1.35621
H	1.89142	-4.18000	-3.04210
N	1.19922	-4.35862	0.98059
H	0.69190	-5.22504	0.84641
H	1.95091	-4.34391	1.66139
H	3.10294	-5.38808	-2.94295
C	-1.20219	-2.49473	2.42938
H	-0.26282	-2.72148	1.61451
O	-1.89776	-3.50116	2.51939
O	-1.11776	-1.35972	2.87500
C	-0.33675	-3.24485	-0.54612
H	-1.03932	-4.06823	-0.38934
H	-0.88585	-2.30656	-0.62954
H	0.21538	-3.42616	-1.47476
O	-1.91364	-0.30937	-0.57903
P	-1.59897	1.06038	-0.03250
O	-0.08817	1.50321	-0.56044
O	-1.73009	1.37055	1.43687
O	-2.48517	2.19959	-0.81324
C	-2.85143	2.03670	-2.19215
H	-2.50680	2.92076	-2.73782
C	-4.35740	1.91327	-2.33561
H	-4.59661	1.91668	-3.40527
O	-4.79232	0.65620	-1.76513
C	-5.80681	0.87941	-0.78802
H	-6.79741	0.76197	-1.22712
C	-5.16989	3.00776	-1.63296
H	-4.57218	3.90638	-1.44064
C	-5.62584	2.32736	-0.32853
H	-4.84713	2.42676	0.42937
O	-6.31442	3.32252	-2.42071
H	-7.02395	3.49831	-1.77126
O	-6.87008	2.82108	0.14493
H	-6.69326	3.64351	0.63265
H	-2.37368	1.15094	-2.61790
C	-6.50857	-1.31005	0.11919
C	-4.64345	-0.13211	1.11119
C	-4.36739	-1.19294	1.90296
H	-4.04752	0.76724	1.14975
C	-5.18603	-2.35265	1.73554
H	-3.54140	-1.16581	2.60006
O	-7.44525	-1.30894	-0.69813
N	-6.22572	-2.38604	0.89953
N	-4.91308	-3.45584	2.46737
H	-3.96641	-3.54838	2.82701
H	-5.38405	-4.31189	2.20022
N	-5.69447	-0.14821	0.23621

GC-Ala

E: -3235.10873
G: -3234.60091

O	-4.31001	-3.51220	-0.18624
C	-3.19164	-2.98302	0.50219
H	-2.68753	-3.75785	1.09717

H	-3.50485	-2.18396	1.18679
C	-2.20891	-2.43991	-0.52436
H	-1.85183	-3.24810	-1.16660
O	-2.86284	-1.47557	-1.39627
C	-2.50238	-0.16002	-1.03611
H	-1.93580	0.32081	-1.83768
C	-1.04255	-1.68773	0.11161
H	-0.75071	-2.09398	1.08171
C	-1.63710	-0.27955	0.22876
H	-2.25390	-0.19432	1.12378
O	-0.61296	0.70521	0.24698
P	-5.71457	-3.75027	0.74863
O	-5.26892	-4.65390	1.91057
O	-6.14604	-2.33886	1.19337
O	-6.64304	-4.40993	-0.27931
N	-3.70054	0.64096	-0.81747
C	-3.74479	2.01393	-0.88106
C	-4.88585	0.23049	-0.22413
C	-4.98594	2.36566	-0.35117
H	-5.10820	-0.81242	-0.03608
N	-5.68758	1.23621	0.05051
C	-5.32668	3.74790	-0.27277
N	-2.77248	2.82343	-1.35940
C	-3.08011	4.10560	-1.26896
N	-4.27956	4.55532	-0.77001
N	-2.17306	5.05156	-1.63029
O	-6.36180	4.27627	0.15888
H	-4.44498	5.55731	-0.72514
C	-0.70826	1.74702	1.10139
C	0.59410	2.52016	1.10110
O	-1.68712	1.97162	1.78790
H	-1.40044	4.71547	-2.19190
N	0.55112	3.57032	2.11993
H	1.47565	4.00139	2.13980
H	0.43097	3.13208	3.03300
H	-2.51361	5.97669	-1.86525
C	0.89532	3.13064	-0.27083
H	1.86872	3.63176	-0.22861
H	0.94210	2.36010	-1.04217
H	0.13422	3.86980	-0.53821
O	2.01871	-0.10041	-1.18175
P	1.57422	-1.23878	-0.29948
O	0.05941	-1.70869	-0.79545
O	1.60337	-1.14417	1.20766
O	2.40654	-2.60637	-0.67996
C	3.18395	-2.72586	-1.87987
H	3.04644	-3.74842	-2.24396
C	4.66339	-2.49381	-1.61883
H	5.22154	-2.83210	-2.49951
O	4.91340	-1.07902	-1.42774
C	5.61856	-0.87602	-0.20674
H	6.69489	-0.91032	-0.38260
C	5.20426	-3.19411	-0.36601
H	4.56638	-4.02707	-0.04989
C	5.22755	-2.06011	0.67631
H	4.23066	-1.95063	1.10537
O	6.53590	-3.64091	-0.60528
H	6.99554	-3.52992	0.25043
O	6.21376	-2.24695	1.67878
H	5.85102	-2.86277	2.33833
H	2.83092	-2.02841	-2.64360
C	6.11483	1.52547	-0.24508
C	4.29793	0.72892	1.14130
C	4.03406	1.99638	1.54212
H	3.69580	-0.10395	1.47455
C	4.83818	3.03790	0.98557
H	3.23409	2.21258	2.23694
O	7.02207	1.25161	-1.04909
N	5.83594	2.79958	0.13268
N	4.59881	4.31615	1.34179
H	3.75093	4.54415	1.84407
H	5.07076	5.05933	0.84320
N	5.33708	0.46127	0.29422
H	1.35737	1.75344	1.29416

GA-1

E: -3820.04045
G: -3819.45988

O	-6.33601	-2.27662	-0.19600
C	-5.06732	-2.44299	0.41314
H	-4.95748	-3.46647	0.79839
H	-4.93891	-1.75243	1.25361
C	-3.97397	-2.20185	-0.61765
H	-4.03386	-2.94346	-1.41800
O	-4.16545	-0.88391	-1.22937
C	-3.01999	-0.09868	-0.97740
H	-2.29181	-0.16485	-1.79029
C	-2.55964	-2.20015	-0.00284
C	-2.44431	-0.70942	0.29917
H	-3.07793	-0.46581	1.15241
O	-1.13528	-0.19796	0.52033
P	-7.44252	-1.27886	0.63589
O	-7.66500	-1.97522	1.98855
O	-6.74316	0.09057	0.75549
O	-8.63759	-1.30652	-0.32324
N	-3.32283	1.31002	-0.80730
C	-2.36449	2.28177	-1.06302
C	-4.14872	1.83722	0.18928
C	-2.68176	3.34448	-0.21900
H	-4.99542	1.26889	0.56082
N	-3.80994	3.05240	0.54413
C	-1.76737	4.43108	-0.14713
N	-1.33439	2.18083	-1.92648
C	-0.53357	3.24363	-1.91598
N	-0.74387	4.32600	-1.10041
N	0.51741	3.30659	-2.77198
O	-1.76804	5.38582	0.65926
H	-0.06226	5.11023	-1.09628
C	-0.94771	0.69461	1.50074
C	0.45583	1.33704	1.40211
O	-1.75071	0.99620	2.35729
O	0.82562	1.99247	2.35871
H	1.31632	3.90565	-2.52941
H	0.77880	2.40989	-3.16558
O	1.02365	6.54319	-0.81523
H	1.80058	6.16074	-1.27857
H	1.12295	6.20384	0.10836
C	1.21620	1.06750	0.14362
H	1.64478	0.06361	0.23595
H	0.50938	0.98046	-0.68567
O	0.95018	5.19794	1.62245
H	1.27334	4.37413	1.19106
H	-0.01579	5.20557	1.45358
O	5.09456	3.20371	-2.03378
H	4.36434	2.59495	-1.73183
H	5.46241	3.51691	-1.19017
O	0.58287	-1.58373	-1.66747
P	-0.00881	-2.63866	-0.76635
O	-1.65230	-2.65147	-1.00926
O	0.27187	-2.68445	0.71635
O	0.27879	-4.13002	-1.40719
C	1.45768	-4.35869	-2.19243
H	1.51783	-3.62672	-3.00347
H	1.33035	-5.35362	-2.62606
C	2.75962	-4.36040	-1.40996
H	3.53064	-4.79096	-2.06792
O	3.13808	-3.02494	-1.03927
C	4.12455	-3.10867	-0.00796

H	5.12395	-3.00613	-0.43781
C	2.76574	-5.11594	-0.07537
H	1.85601	-4.88185	0.47970
C	3.99301	-4.50450	0.63957
H	3.86395	-4.44972	1.72264
O	2.83160	-6.52175	-0.21066
H	3.72419	-6.71430	-0.55275
O	5.17659	-5.23486	0.32040
H	5.22721	-5.98637	0.93476
H	-2.48335	-2.82182	0.89179
N	3.95014	-1.99791	0.90224
C	4.60016	-0.77694	0.82716
C	3.00120	-1.86515	1.90191
C	4.01003	0.01744	1.81577
H	2.32826	-2.67190	2.14541
N	3.01208	-0.68689	2.48263
C	4.47789	1.34192	1.91762
N	5.59082	-0.41265	0.00144
C	5.97934	0.84817	0.22770
N	5.50053	1.72549	1.12376
N	3.95831	2.23837	2.80483
H	4.17278	3.20495	2.58272
H	2.97766	2.09504	3.02932
H	6.78637	1.21557	-0.39943
C	2.29446	2.05378	-0.29343
O	3.07808	1.61270	-1.17764
O	2.29291	3.23253	0.17541
O	2.86863	4.83395	-1.89918
H	3.74026	4.46285	-2.17698
H	2.70611	4.31759	-1.06450

GA-TS-1

E: -3819.97565
G: -3819.39426

O	-3.22170	-5.04096	-0.73504
C	-2.40874	-4.23947	0.10441
H	-1.80051	-4.86190	0.77554
H	-3.02198	-3.57034	0.72371
C	-1.48316	-3.43156	-0.79146
H	-0.91141	-4.10908	-1.42930
O	-2.25290	-2.58489	-1.69173
C	-2.27994	-1.26381	-1.22401
H	-1.89630	-0.58357	-1.98774
C	-0.54639	-2.48363	-0.04654
H	-0.26242	-2.85872	0.93759
C	-1.39453	-1.20769	0.05420
H	-2.01186	-1.24664	0.95183
O	-0.61681	0.00163	0.05948
P	-4.61300	-5.73438	-0.03455
O	-4.10709	-6.45148	1.22723
O	-5.55481	-4.55194	0.26094
O	-5.07728	-6.65569	-1.17037
N	-3.67549	-0.85207	-0.94193
C	-3.92525	0.38340	-0.41323
C	-4.80120	-1.60546	-0.66526
C	-5.19402	0.34695	0.13981
H	-4.87064	-2.65335	-0.91848
N	-5.74555	-0.91270	-0.05030
C	-5.59724	1.45358	0.96636
N	-2.97758	1.32801	-0.26510
C	-3.27821	2.24048	0.60070
N	-4.51505	2.37282	1.15482
N	-2.17051	2.97344	1.14058
O	-6.66945	1.65009	1.53944
H	-4.68109	3.11530	1.83118
C	-0.81334	0.85851	1.08602
C	-0.54476	2.37880	0.76386
O	-1.16904	0.51109	2.19451
O	0.21658	2.95169	1.63553
H	-2.16221	2.81154	2.15562
O	2.43079	-0.51690	-1.06259
P	1.97560	-1.62997	-0.15408
O	0.60530	-2.25930	-0.85602
O	1.75008	-1.44213	1.32380
O	2.97831	-2.93052	-0.26873
C	3.81609	-3.19024	-1.40095
H	3.51926	-2.57889	-2.25743
H	3.69161	-4.24575	-1.66363
C	5.27903	-2.92928	-1.08241
H	5.88028	-3.33243	-1.90437
O	5.52502	-1.50289	-1.01219
C	5.92723	-1.11617	0.29027
H	7.00322	-0.91410	0.30022
C	5.76956	-3.49612	0.24927
H	5.18344	-4.36413	0.57321
C	5.59905	-2.30319	1.20364
H	4.55920	-2.25745	1.52993
O	7.15158	-3.82248	0.14306
H	7.52381	-3.63445	1.02656
O	6.49783	-2.32274	2.30061
H	6.11460	-2.89449	2.98723
N	5.22225	0.10861	0.65724
C	5.04560	1.20601	-0.16943
C	4.38336	0.31147	1.73961
C	4.09620	2.00278	0.47454
H	4.34717	-0.38387	2.56344
N	3.70808	1.43637	1.68100
C	3.64118	3.13980	-0.22204
N	5.62971	1.47131	-1.35020
C	5.15670	2.60216	-1.88548
N	4.20705	3.42769	-1.41768
C	-0.27934	2.64042	-0.73104
H	0.77673	2.39705	-0.87432
H	-0.87953	1.96737	-1.33807
C	-0.57566	4.07134	-1.18162
O	-1.79388	4.33423	-1.41964
O	0.37132	4.90757	-1.29487
O	-1.85216	6.86569	-2.29332
H	-1.12329	7.25191	-1.77864
H	-1.80564	5.89832	-2.03274
O	0.13832	5.71975	1.40979
H	0.29349	5.74241	0.44251
H	0.16253	4.74076	1.58006
O	-2.58868	5.60814	1.37566
H	-3.01477	6.09690	0.62414
H	-1.62743	5.85341	1.36941
H	-2.22987	4.03474	1.04664
O	-3.93265	6.97375	-0.57163
H	-4.45089	6.22463	-0.91231
H	-3.19162	7.05081	-1.23319
N	2.67329	3.95264	0.27066
H	2.13540	4.46915	-0.43026
H	2.04458	3.52069	0.95114
H	5.59091	2.88841	-2.84034

GA-2

E: -3743.53918
G: -3742.98469

O	-5.80905	-2.59486	-0.25071
C	-4.62171	-2.29096	0.46012
H	-4.35930	-3.10093	1.15553
H	-4.73825	-1.36777	1.04189
C	-3.49791	-2.12248	-0.54914
H	-3.40203	-3.02504	-1.15698
O	-3.82189	-1.03179	-1.46197
C	-3.01416	0.08714	-1.19688
H	-2.29984	0.25628	-2.00605
C	-2.15489	-1.75322	0.08125

H	-2.02016	-2.18628	1.07336
C	-2.26215	-0.23143	0.12231
H	-2.85017	0.08662	0.98329
O	-0.96817	0.40331	0.15720
P	-7.26927	-2.14166	0.50143
O	-7.21610	-2.78752	1.89610
O	-7.23920	-0.60037	0.52975
O	-8.29539	-2.74413	-0.46641
N	-3.84852	1.29952	-1.09900
C	-3.23403	2.50891	-0.89887
C	-5.13077	1.44898	-0.58872
C	-4.16149	3.34904	-0.30874
H	-5.83666	0.62699	-0.55430
N	-5.36737	2.67192	-0.14970
C	-3.68167	4.58034	0.26100
N	-1.90211	2.68004	-1.01327
C	-1.44877	3.67800	-0.29865
N	-2.25136	4.65058	0.21307
N	-0.08476	3.63043	0.04542
O	-4.31495	5.47872	0.81754
H	-1.83339	5.38632	0.77770
C	-0.85158	1.48998	0.93855
C	0.25042	2.47641	0.52483
O	-1.53923	1.73208	1.91022
O	1.34427	-1.32681	-0.67312
P	0.42801	-2.41965	-0.18175
O	-1.10200	-2.16321	-0.79017
O	0.32056	-2.76207	1.28232
O	0.74621	-3.82629	-0.96831
C	1.42683	-3.85457	-2.23052
H	1.39996	-2.87295	-2.70972
H	0.89992	-4.57277	-2.86620
C	2.87074	-4.29903	-2.06999
H	3.29061	-4.46067	-3.06857
O	3.64088	-3.25272	-1.42445
C	4.16575	-3.70324	-0.18834
H	5.22696	-3.94876	-0.30378
C	3.06716	-5.54828	-1.21128
H	2.17657	-6.18729	-1.19865
C	3.36884	-4.95940	0.17615
H	2.43010	-4.69059	0.66327
O	4.20475	-6.26806	-1.67535
H	4.60278	-6.65672	-0.87204
O	4.16194	-5.80939	0.98818
H	3.56762	-6.45417	1.40873
N	4.05401	-2.64018	0.80364
C	4.50469	-1.34082	0.65305
C	3.44589	-2.69096	2.04420
C	4.13797	-0.68516	1.83427
H	3.02463	-3.60510	2.43111
N	3.47872	-1.55040	2.69656
C	4.47568	0.67747	1.92963
N	5.15349	-0.79399	-0.38706
C	5.42758	0.49725	-0.17009
N	5.14633	1.24681	0.90654
C	1.64893	2.05344	0.82217
H	1.69712	1.77268	1.88101
H	1.83133	1.13638	0.24470
C	2.69458	3.12478	0.48613
O	3.04986	3.19753	-0.73127
O	3.09212	3.85469	1.43372
O	5.16206	4.95841	-1.12296
H	4.75880	5.63897	-0.53123
H	4.50564	4.22639	-1.01411
O	3.49441	6.43649	0.52994
H	3.43123	5.55674	0.97668
H	2.81820	6.32528	-0.17922
O	1.72024	5.52339	-1.44072
H	0.92679	5.23202	-0.94983
H	2.28661	4.71972	-1.37010
O	3.83993	6.17942	-3.25276
H	2.95125	6.15692	-2.85606
H	4.38264	5.72604	-2.56340
N	4.18471	1.44276	3.02214
H	4.06368	2.43138	2.81007
H	3.46580	1.06784	3.63081
H	5.95320	1.01181	-0.97019

GA-TS-2

E: -3570.77104
G: -3570.25147

O	-5.13312	-3.14135	0.07415
C	-3.93203	-2.65040	0.64209
H	-3.53801	-3.34570	1.39693
H	-4.09734	-1.67972	1.12739
C	-2.90946	-2.50080	-0.47361
H	-2.76209	-3.45987	-0.97538
O	-3.41669	-1.57435	-1.47937
C	-2.72195	-0.35311	-1.42367
H	-2.10643	-0.21306	-2.31541
C	-1.56716	-1.92663	-0.00876
H	-1.34564	-2.19496	1.02648
C	-1.83147	-0.43042	-0.15756
H	-2.39161	-0.07467	0.70684
O	-0.63286	0.34598	-0.30354
P	-6.56656	-2.78749	0.92276
O	-6.32336	-3.29725	2.35154
O	-6.72064	-1.25564	0.83020
O	-7.59082	-3.58411	0.10533
N	-3.67622	0.77468	-1.37997
C	-3.18625	2.05926	-1.38295
C	-4.90630	0.85352	-0.73802
C	-4.13578	2.86328	-0.77664
H	-5.51014	-0.02502	-0.54659
N	-5.23334	2.08704	-0.40337
C	-3.73887	4.19455	-0.39470
N	-1.90765	2.35566	-1.68056
C	-1.49239	3.46781	-1.10757
N	-2.34811	4.40895	-0.61515
N	-0.11695	3.57962	-0.92455
O	-4.41762	5.07666	0.14346
H	-1.91417	5.22983	-0.17186
C	-0.62975	1.54519	0.32021
C	0.37798	2.54011	-0.29162
O	-1.39191	1.83355	1.22069
C	1.77161	2.05939	-0.52385
H	2.09943	1.43960	0.31302
H	1.69462	1.39705	-1.39307
C	2.85189	3.11985	-0.83537
O	-0.25510	6.20435	0.23661
H	-0.14243	6.04346	1.18852
H	0.19471	5.43633	-0.17660
N	0.95753	3.32446	1.84041
H	1.29215	2.77099	2.62673
H	0.21765	3.94466	2.15915
H	1.73249	3.86441	1.44751
O	2.94671	4.13051	-0.07303
O	3.59755	2.87125	-1.82048
O	1.34402	-1.54220	0.69456
P	0.97950	-2.66233	-0.24725
O	-0.52946	-2.36169	-0.88989
O	1.06807	-4.09524	0.21304
O	1.80867	-2.61115	-1.65895
C	1.87774	-1.40679	-2.43988
H	1.21201	-0.64121	-2.03651
H	1.54653	-1.65273	-3.45379
C	3.29840	-0.87419	-2.49293
H	3.32192	-0.02708	-3.18707
O	3.68153	-0.38931	-1.18535

C	4.81122	-1.09058	-0.69468
H	5.71569	-0.49287	-0.84571
C	4.36104	-1.90505	-2.88074
H	3.94215	-2.73327	-3.46434
C	4.89405	-2.37843	-1.51541
H	4.22588	-3.14324	-1.11665
O	5.40880	-1.25844	-3.59551
H	6.22379	-1.72601	-3.32660
O	6.23921	-2.82443	-1.56270
H	6.23357	-3.74818	-1.86584
N	4.63950	-1.28346	0.73664
C	4.38546	-0.24009	1.60866
C	4.36712	-2.44176	1.44383
C	3.99264	-0.84150	2.80584
H	4.48526	-3.41934	1.00202
N	4.00873	-2.22616	2.68992
C	3.62661	0.02945	3.85201
N	4.48164	1.07772	1.37123
C	4.09805	1.79627	2.43069
N	3.68100	1.36257	3.63250
N	3.25156	-0.41662	5.07602
H	2.78484	0.24468	5.68415
H	2.96560	-1.38352	5.15966
H	4.12351	2.87477	2.30406

GA-3

E: -3571.29644
G: -3570.76261

O	4.48013	-3.80497	0.54662
C	3.41840	-3.31881	-0.25422
H	2.89646	-4.14147	-0.76380
H	3.79764	-2.63269	-1.02204
C	2.42440	-2.59487	0.63958
H	2.01509	-3.28087	1.38467
O	3.09190	-1.52491	1.36892
C	2.69579	-0.26746	0.86705
H	2.03530	0.25114	1.56778
C	1.29919	-1.92047	-0.14662
H	1.02980	-2.46261	-1.05391
C	1.93916	-0.56359	-0.43954
H	2.61981	-0.61754	-1.28897
O	0.94645	0.44444	-0.67190
P	5.95681	-4.05091	-0.26765
O	5.61852	-5.00580	-1.42446
O	6.39077	-2.64570	-0.73282
O	6.81688	-4.65007	0.85145
N	3.86169	0.57592	0.66627
C	3.83387	1.95341	0.60934
C	5.08582	0.17752	0.14301
C	5.07346	2.31734	0.08541
H	5.36393	-0.86684	0.05929
N	5.84467	1.19542	-0.19285
C	5.34387	3.70042	-0.12827
N	2.79752	2.74874	0.96303
C	3.04097	4.03795	0.75211
N	4.24100	4.49364	0.24781
N	2.09436	4.96684	0.98921
O	6.36509	4.23572	-0.58438
H	4.36091	5.49315	0.10818
C	1.27653	1.49797	-1.40947
C	0.19267	2.54945	-1.31941
O	2.29424	1.66143	-2.04900
O	-1.98783	-0.44992	0.88400
P	-1.34644	-1.54580	0.07371
O	0.17549	-1.75488	0.71281
O	-1.26876	-1.49665	-1.42968
O	-2.01587	-3.00651	0.42464
C	-2.63855	-3.26473	1.69068
H	-2.35130	-2.51238	2.42967
H	-2.29241	-4.24510	2.03202
C	-4.15111	-3.28802	1.55253
H	-4.57880	-3.63896	2.49775
O	-4.64083	-1.94519	1.30590
C	-5.30004	-1.87583	0.05176
H	-6.38439	-1.90838	0.20163
C	-4.67236	-4.13940	0.39387
H	-3.97198	-4.93570	0.11642
C	-4.84417	-3.10778	-0.73325
H	-3.87810	-2.93103	-1.20801
O	-5.94564	-4.67736	0.73610
H	-6.44897	-4.68786	-0.10155
O	-5.84122	-3.46649	-1.67572
H	-5.43875	-4.08498	-2.30920
N	-4.98305	-0.61144	-0.59444
C	-5.25001	0.63672	-0.06126
C	-4.32440	-0.38211	-1.78867
C	-4.74503	1.55257	-0.98891
H	-4.00860	-1.18963	-2.42945
N	-4.17543	0.89348	-2.07063
C	-4.88591	2.91605	-0.66562
N	-5.86450	0.93288	1.09452
C	-5.94393	2.25504	1.28005
N	-5.50606	3.24537	0.48663
C	-1.18764	2.24347	-0.90972
H	-1.84198	2.41603	-1.77390
H	-1.29315	1.20864	-0.60156
C	-1.69808	3.20511	0.21805
O	-2.28123	2.67107	1.17663
O	-1.46886	4.44394	0.03536
O	-0.32679	4.84825	2.51373
H	-0.85134	4.86177	1.67652
H	-0.46101	3.93778	2.82657
N	-4.46504	3.91539	-1.48862
H	-4.31480	4.81433	-1.04542
H	-3.77316	3.66824	-2.18582
H	-6.43086	2.57555	2.19732
H	1.26019	4.72825	1.54044
H	2.36340	5.94279	0.98737
N	0.56571	3.75609	-1.57428
H	1.53465	3.96826	-1.81161
H	-0.10611	4.50317	-1.34408

GA-TS-3

E: -3684.15829
G: -3683.63093

O	-4.35851	-4.03270	-0.17768
C	-3.30356	-3.40569	0.53017
H	-2.75218	-4.13375	1.14236
H	-3.69128	-2.62619	1.19713
C	-2.34765	-2.79176	-0.47975
H	-1.96173	-3.56444	-1.14883
O	-3.05934	-1.82415	-1.30812
C	-2.61775	-0.51663	-1.00904
H	-1.95869	-0.12727	-1.79029
C	-1.19349	-2.01750	0.16259
H	-0.87043	-2.43824	1.11575
C	-1.84363	-0.64504	0.31031
H	-2.52474	-0.63380	1.16123
O	-0.89045	0.41460	0.44179
P	-5.86493	-4.08398	0.61459
O	-5.59166	-4.79766	1.94882
O	-6.28129	-2.60863	0.77738
O	-6.69862	-4.87889	-0.39721
N	-3.75207	0.38534	-0.91814
C	-3.68368	1.74837	-1.11015
C	-4.96401	0.13147	-0.28866
C	-4.88850	2.24628	-0.61691
H	-5.26971	-0.87410	-0.02077

N	-5.67922	1.21971	-0.11455
C	-5.10423	3.65522	-0.64292
N	-2.64238	2.42912	-1.64033
C	-2.83773	3.74021	-1.66315
N	-3.99294	4.32597	-1.19691
N	-1.91441	4.56534	-2.21297
O	-6.08448	4.30474	-0.24975
H	-4.07830	5.33766	-1.24059
C	-1.28850	1.51695	1.08917
C	-0.24101	2.61271	1.00560
O	-2.33298	1.64191	1.69651
O	1.92428	-0.44392	-0.93939
P	1.44174	-1.69524	-0.25088
O	-0.11369	-1.97246	-0.76701
O	1.50914	-1.87495	1.24356
O	2.16807	-3.02592	-0.88839
C	2.81179	-3.02468	-2.16845
H	2.46678	-2.18690	-2.77988
H	2.53934	-3.96029	-2.66653
C	4.32390	-2.95971	-2.02940
H	4.76553	-3.16353	-3.01085
O	4.72346	-1.62502	-1.62703
C	5.40166	-1.65244	-0.38372
H	6.48237	-1.57870	-0.54633
C	4.91835	-3.90421	-0.98370
H	4.27245	-4.76826	-0.78989
C	5.05097	-3.00027	0.25359
H	4.08736	-2.94273	0.76285
O	6.21414	-4.32067	-1.40314
H	6.73302	-4.39727	-0.57864
O	6.09026	-3.39943	1.13185
H	5.74280	-4.11353	1.69325
N	4.97773	-0.50648	0.41238
C	4.89220	0.79782	-0.04196
C	4.43280	-0.50308	1.68402
C	4.29435	1.51409	1.00070
H	4.39714	-1.39752	2.28623
N	4.02848	0.68244	2.08066
C	4.01164	2.87260	0.75555
N	5.27390	1.29572	-1.22900
C	4.98229	2.59718	-1.32849
N	4.38140	3.39806	-0.43543
C	0.59914	2.68247	-0.26538
H	1.50841	2.08625	-0.14735
H	0.02657	2.20396	-1.06172
C	0.99900	4.09488	-0.74427
O	0.99442	4.25974	-1.99418
O	1.31414	4.98012	0.11049
N	3.39969	3.68209	1.66291
H	2.82349	4.40713	1.23001
H	2.91250	3.20081	2.41520
H	5.26435	3.07398	-2.26399
H	-0.95461	4.21917	-2.27687
H	-1.98349	5.55090	-1.98722
N	-0.62819	3.76923	1.56456
H	-1.27453	3.74918	2.34451
H	-0.00105	4.56165	1.41817
C	1.02313	1.51103	3.06385
H	0.62052	1.92387	1.90570
O	0.78826	0.32375	3.19864
O	1.47202	2.47366	3.67433

GA-Asp

E: -3572.06158
G: -3571.51550

O	4.60538	-3.84957	0.32239
C	3.47354	-3.38546	-0.39182
H	2.99391	-4.20235	-0.94995
H	3.76236	-2.60881	-1.11090
C	2.47065	-2.81882	0.60228
H	2.13407	-3.60071	1.28737
O	3.10438	-1.78827	1.41466
C	2.61106	-0.51274	1.06301
H	1.96392	-0.10920	1.84639
C	1.28369	-2.14168	-0.08126
H	1.02981	-2.59492	-1.04111
C	1.81974	-0.71326	-0.23308
H	2.47839	-0.64411	-1.09933
O	0.78972	0.25896	-0.32243
P	6.07095	-3.88329	-0.54506
O	5.79183	-4.78395	-1.75960
O	6.35147	-2.41342	-0.91751
O	7.02074	-4.47074	0.50630
N	3.71343	0.42153	0.89716
C	3.58754	1.78780	1.01587
C	4.89952	0.18927	0.21224
C	4.73080	2.31509	0.41640
H	5.24291	-0.81343	-0.02083
N	5.54459	1.29918	-0.07120
C	4.86132	3.73224	0.32941
N	2.55427	2.44626	1.58566
C	2.66937	3.76052	1.49973
N	3.75394	4.37673	0.91917
N	1.73445	4.58362	2.05245
O	5.77037	4.40481	-0.18110
H	3.75511	5.39100	0.85631
C	0.49605	0.78461	-1.54187
C	-0.85288	1.47227	-1.48844
O	1.19928	0.61922	-2.51903
O	-1.89357	-0.65328	1.03193
P	-1.36538	-1.82729	0.24673
O	0.16184	-2.17581	0.80279
O	-1.36862	-1.84312	-1.26263
O	-2.13011	-3.21089	0.70433
C	-2.87106	-3.31616	1.92709
H	-2.54965	-2.55904	2.64689
H	-2.66274	-4.30750	2.34080
C	-4.36712	-3.18799	1.68978
H	-4.88560	-3.50732	2.60034
O	-4.71198	-1.80031	1.43870
C	-5.34885	-1.66671	0.18010
H	-6.43641	-1.64367	0.30967
C	-4.89382	-3.97312	0.48641
H	-4.23897	-4.80976	0.21802
C	-4.94101	-2.90513	-0.61978
H	-3.94273	-2.77883	-1.04363
O	-6.21612	-4.42792	0.75686
H	-6.67657	-4.39397	-0.10452
O	-5.91077	-3.17404	-1.61818
H	-5.51916	-3.80468	-2.24672
N	-4.94714	-0.40620	-0.42775
C	-5.10722	0.84127	0.14887
C	-4.32514	-0.18993	-1.64230
C	-4.56445	1.74742	-0.76856
H	-4.09341	-0.99821	-2.31807
N	-4.09620	1.08090	-1.89352
C	-4.55034	3.10255	-0.38162
N	-5.66048	1.14563	1.33286
C	-5.61638	2.46112	1.56887
N	-5.09609	3.43655	0.80676
C	-0.97535	2.45244	-0.30957
H	-2.02843	2.66985	-0.13431
H	-0.59381	1.99077	0.60764
C	-0.21346	3.75836	-0.53557
O	-0.74403	4.81568	-0.05764
O	0.88519	3.70316	-1.15337
O	1.20719	6.65688	0.07187
H	1.87306	6.26007	-0.51506
H	0.43261	6.03013	-0.04409

N	-4.03696	4.09439	-1.16341
H	-3.77858	4.94058	-0.66761
H	-3.35965	3.81236	-1.86264
H	-6.05143	2.78848	2.50929
H	0.85739	4.10513	2.23058
H	1.59156	5.46379	1.53991
N	-1.21341	2.00719	-2.79823
H	-2.22930	2.08999	-2.80715
H	-0.82215	2.94332	-2.89301
H	-1.53792	0.64632	-1.28257

CA-1

E: -3973.71767
G: -3973.19790

O	2.56588	-1.21246	-1.87230
C	1.70858	-2.25786	-2.36497
H	2.35167	-3.03209	-2.79012
H	1.04010	-1.88747	-3.14744
C	0.87600	-2.85696	-1.24625
H	0.29297	-3.67176	-1.67969
O	1.69886	-3.45028	-0.21129
C	1.78026	-2.60927	0.90800
H	1.37394	-3.12916	1.78094
C	-0.03286	-1.87133	-0.49844
H	-0.40282	-1.06688	-1.13272
C	0.92138	-1.37033	0.57863
H	1.54388	-0.57713	0.17356
O	0.30446	-0.91657	1.78765
P	2.59722	0.25997	-2.54986
O	2.44727	0.20433	-4.03872
O	1.48365	1.11746	-1.80356
O	3.96489	0.80317	-2.00778
C	3.23107	-1.48827	2.45167
C	4.34121	-0.80134	2.81344
C	5.40491	-0.77475	1.86138
N	3.16825	-2.20210	1.28400
C	4.32709	-2.31864	0.46747
N	5.39441	-1.52494	0.76559
C	0.12382	0.41034	1.91538
C	-0.03636	0.81151	3.38552
C	-0.52347	2.21718	3.65150
H	0.28311	2.90330	3.36036
H	-0.65377	2.30044	4.73446
C	-1.81576	2.55540	2.90926
H	-2.29682	3.44450	3.32612
H	-1.60434	2.77088	1.85856
C	-2.77844	1.36631	2.93947
O	-4.00878	1.56432	2.77811
O	0.18214	1.20217	0.99517
O	0.47093	0.09920	4.24889
O	-2.22840	0.22307	3.09899
O	4.37203	-3.13696	-0.46265
N	6.49007	0.01102	2.08733
H	7.03644	0.24995	1.26751
H	6.37773	0.76275	2.75679
H	4.37878	-0.23404	3.73447
H	2.33532	-1.49084	3.06126
P	4.22664	2.00722	-0.69432
O	5.74578	1.90921	-0.58661
O	3.68432	3.29330	-1.31606
O	3.42780	1.43863	0.47558
H	0.53663	1.12749	-2.17467
O	-1.11476	-2.54659	0.14702
P	-2.28875	-3.23165	-0.79948
O	-1.90854	-4.64816	-1.15949
O	-2.65555	-2.27318	-1.91086
O	-3.45881	-3.37703	0.33045
C	-3.82247	-2.38159	1.30020
H	-2.94900	-2.09750	1.89288
H	-4.54434	-2.87857	1.95241
C	-4.46627	-1.12672	0.74354
H	-4.99543	-0.62960	1.56750
O	-3.44145	-0.24262	0.26478
C	-4.10135	0.71896	-0.53923
H	-4.62544	1.45504	0.07909
C	-5.44678	-1.29771	-0.44011
H	-5.24041	-2.22842	-0.96946
C	-5.12877	-0.07066	-1.35910
H	-4.70672	-0.39918	-2.31047
O	-6.80385	-1.31261	-0.03098
H	-7.13755	-0.41357	-0.22533
O	-6.26551	0.75715	-1.56308
H	-6.77189	0.38934	-2.30709
N	-3.08043	1.43380	-1.27075
C	-2.58261	2.67879	-0.90526
C	-2.09352	0.82265	-2.01376
C	-1.31387	2.76086	-1.47921
H	-2.20347	-0.19404	-2.36664
N	-1.04076	1.59114	-2.18896
C	-0.54908	3.90439	-1.16246
N	-3.18564	3.61720	-0.16499
C	-2.39044	4.67933	0.01480
N	-1.13735	4.87162	-0.41909
H	-2.81124	5.49169	0.60115
N	0.72627	4.09138	-1.57730
H	1.29820	3.27559	-1.78260
H	1.23166	4.83595	-1.11176

CA-TS-1

E: -3973.67847
G: -3973.16147

O	-4.01023	-2.00731	0.58906
C	-3.55762	-3.30594	0.17807
H	-4.42303	-3.83364	-0.23284
H	-3.17673	-3.87946	1.02827
C	-2.47113	-3.24876	-0.88599
H	-2.16470	-4.27584	-1.10175
O	-2.96783	-2.68470	-2.13066
C	-2.44727	-1.39558	-2.33334
H	-1.75522	-1.40722	-3.18218
C	-1.25252	-2.40209	-0.51662
H	-1.06471	-2.40184	0.55609
C	-1.68517	-1.04001	-1.04480
H	-2.33357	-0.52673	-0.33974
O	-0.59605	-0.17569	-1.40468
P	-3.77835	-1.47792	2.13075
O	-4.35099	-2.73516	3.02674
O	-2.31020	-1.38365	2.45556
O	-4.70078	-0.28412	2.26988
C	-2.90341	0.80907	-3.11733
C	-3.54533	1.99695	-3.01843
C	-4.79843	1.97690	-2.33171
N	-3.46375	-0.36003	-2.66752
C	-4.81686	-0.35625	-2.21387
N	-5.43331	0.84673	-2.05081
C	-0.12686	0.64860	-0.46340
C	0.93413	1.58975	-1.04847
C	0.78601	3.03407	-0.67940
H	1.03945	3.13107	0.38585
H	1.52078	3.59945	-1.25729
C	-0.65698	3.55474	-0.86174
H	-0.63165	4.48539	-1.44065
H	-1.27342	2.86409	-1.44725
C	-1.40578	3.85596	0.45286
O	-0.74764	3.95574	1.51807
O	-0.46621	0.68287	0.69940
O	1.83374	1.11733	-1.72598
O	-2.66803	4.01115	0.34125

O	-5.41071	-1.42383	-2.00158
N	-5.38835	3.15332	-1.98927
H	-6.08737	3.08466	-1.25725
H	-4.76078	3.94325	-1.88540
H	-3.09125	2.91743	-3.36148
H	-1.90640	0.72480	-3.53318
P	-3.75934	1.96453	1.44307
O	-5.11873	2.62133	1.47572
O	-2.87146	2.02426	2.65622
O	-3.31935	1.23998	0.20234
H	-5.30706	-2.85455	2.88165
O	1.32525	-4.21351	0.39028
P	1.35238	-3.01631	-0.52884
O	-0.11678	-2.84262	-1.26551
O	2.35185	-2.91735	-1.65224
O	1.45087	-1.63551	0.35951
C	1.40490	-1.65818	1.79334
H	1.45379	-2.68968	2.14627
H	0.46155	-1.21488	2.12492
C	2.54584	-0.86013	2.38256
H	2.45767	-0.92804	3.47614
O	3.81275	-1.42858	1.97695
C	4.78463	-0.40714	1.82965
H	5.66266	-0.63138	2.43917
C	2.64843	0.61872	1.99240
H	2.46016	0.74048	0.92579
C	4.13209	0.90837	2.27005
H	4.51436	1.76559	1.70771
O	1.73939	1.46672	2.66347
H	1.80870	1.28757	3.61752
O	4.38249	1.05369	3.66356
H	3.96349	1.88414	3.94700
N	5.22501	-0.35541	0.43312
C	6.39142	0.23513	-0.00706
C	4.52656	-0.73393	-0.69719
C	6.33675	0.15921	-1.40243
H	3.57613	-1.23828	-0.63095
N	5.16347	-0.45559	-1.81730
C	7.43101	0.70723	-2.10055
N	7.37917	0.77517	0.72717
C	8.35420	1.25755	-0.05089
N	8.43991	1.26049	-1.39097
H	9.19243	1.71426	0.46873
N	7.52902	0.66811	-3.45228
H	6.68792	0.48790	-3.98457
H	8.21587	1.27388	-3.88343

CA-2

E: -3973.70025
G: -3973.18245

O	-0.38858	3.79576	-1.04145
C	-1.64838	3.64341	-0.37308
H	-2.32695	3.17933	-1.09058
H	-2.06329	4.61290	-0.07854
C	-1.48114	2.74804	0.85333
H	-0.82420	3.23180	1.57664
O	-2.77850	2.53925	1.47308
C	-3.07271	1.15473	1.50743
H	-2.79777	0.73844	2.48161
C	-0.92367	1.36258	0.51172
H	-0.37225	1.35842	-0.42673
C	-2.19193	0.51757	0.43539
H	-2.64892	0.57116	-0.55051
O	-1.98411	-0.85246	0.80421
P	0.68428	4.94608	-0.54512
O	-0.02637	6.33480	-1.08199
O	0.69662	5.06979	0.95825
O	1.94135	4.65469	-1.32615
C	-5.07150	-0.01631	2.20230
C	-6.29289	-0.54737	1.95942
C	-6.92820	-0.16118	0.73752
N	-4.49532	0.87516	1.33577
C	-5.17106	1.25204	0.14239
N	-6.40974	0.74725	-0.08422
C	-1.60697	-1.69690	-0.15885
C	-1.40016	-3.09750	0.41948
C	-2.56302	-4.04973	0.44538
H	-2.83622	-4.25164	-0.59936
H	-2.20346	-4.98014	0.89043
C	-3.80534	-3.51901	1.21901
H	-4.23760	-4.32982	1.80707
H	-3.52245	-2.71921	1.91181
C	-4.86577	-2.99763	0.27042
O	-6.00517	-3.45324	0.24078
O	-1.37863	-1.41275	-1.31553
O	-0.27268	-3.37776	0.79556
O	-4.40768	-2.03739	-0.51895
O	-4.61496	2.03126	-0.64683
N	-8.12612	-0.70055	0.40727
H	-8.38271	-0.63615	-0.57118
H	-8.38300	-1.56672	0.86493
H	-6.74330	-1.26222	2.63543
H	-4.49075	-0.26720	3.08171
P	-4.88041	-1.88283	-2.24652
O	-4.16813	-0.56846	-2.55574
O	-6.40552	-1.84297	-2.26255
O	-4.24710	-3.15156	-2.81909
H	-0.02280	6.36836	-2.05531
O	2.05573	0.06886	2.56996
P	1.44339	0.45413	1.25095
O	-0.11341	0.88820	1.59104
O	1.43765	-0.48363	0.06584
O	2.00463	1.91096	0.72406
C	3.13453	2.55904	1.31669
H	3.17148	2.35594	2.39049
H	2.99216	3.63002	1.16218
C	4.43126	2.13759	0.65665
H	5.24549	2.76678	1.04653
O	4.69257	0.75848	0.96157
C	5.64183	0.27545	0.02051
H	6.66391	0.41986	0.38399
C	4.43890	2.19679	-0.88041
H	3.44647	1.94553	-1.25685
C	5.45711	1.09368	-1.27611
H	5.08481	0.46949	-2.09154
O	4.78210	3.46836	-1.39645
H	5.75523	3.51502	-1.33494
O	6.72288	1.65434	-1.61744
H	6.68203	1.91287	-2.55379
N	5.44759	-1.14878	-0.12533
C	6.39668	-2.13646	0.05016
C	4.27239	-1.78967	-0.47284
C	5.72598	-3.33518	-0.20965
H	3.35534	-1.23527	-0.62735
N	4.39476	-3.09866	-0.53161
C	6.48972	-4.51452	-0.10319
N	7.68876	-2.00039	0.38788
C	8.30110	-3.18770	0.45593
N	7.79523	-4.41105	0.23353
H	9.35378	-3.16237	0.72494
N	5.95958	-5.74763	-0.28644
H	5.06679	-5.82131	-0.75605
H	6.60388	-6.51867	-0.40771

CA-TS-2

E: -4106.69093
G: -4106.10995

O	0.23536	4.42885	1.54562

C	1.26432	3.92286	0.68251
H	1.79985	3.15789	1.24824
H	1.97597	4.70657	0.40658
C	0.62819	3.34788	-0.58233
H	-0.03428	4.09479	-1.02124
O	1.66152	3.03654	-1.55849
C	1.74073	1.63306	-1.71795
H	1.18700	1.33407	-2.61295
C	-0.12737	2.03803	-0.34532
H	-0.61233	1.99112	0.62979
C	1.02946	1.04741	-0.50139
H	1.66140	1.03702	0.38809
O	0.68136	-0.29959	-0.83947
P	-0.20300	6.01492	1.41234
O	1.16188	6.78760	1.92081
O	-0.36355	6.41122	-0.03436
O	-1.32206	6.18668	2.40808
C	3.26204	0.15032	-2.85252
C	4.43651	-0.50692	-2.98851
C	5.50639	-0.10202	-2.12889
N	3.10386	1.14669	-1.92433
C	4.16865	1.47385	-1.04334
N	5.37779	0.88399	-1.24668
C	0.38244	-1.14774	0.15138
C	0.27576	-2.56075	-0.42402
C	1.33609	-3.53283	0.00675
H	1.38297	-3.50647	1.09985
H	1.01202	-4.52751	-0.30500
C	2.72474	-3.19446	-0.60327
H	3.19799	-4.11622	-0.95034
H	2.61653	-2.54821	-1.48322
C	3.72465	-2.48548	0.31733
O	4.91850	-2.34393	-0.10323
O	0.21412	-0.87040	1.31801
O	-0.63606	-2.81683	-1.19352
O	3.09427	-1.40449	0.92466
O	3.97607	2.29220	-0.13167
N	6.71283	-0.71355	-2.25287
H	7.35299	-0.53750	-1.48582
H	6.66166	-1.69431	-2.53664
H	4.56066	-1.29025	-3.72381
H	2.39524	-0.07480	-3.46169
P	3.70745	-0.53465	2.29073
O	2.68758	0.60560	2.37835
O	5.14048	-0.11954	1.96113
O	3.59683	-1.56817	3.43637
H	1.34062	6.58944	2.85732
O	5.78437	-3.56083	-2.33698
H	5.06188	-3.40955	-2.96743
H	5.42713	-3.16837	-1.49033
N	3.85669	-3.68162	1.71214
H	4.77978	-4.09993	1.61209
H	3.15600	-4.42059	1.76236
H	3.81088	-3.07835	2.55972
O	-1.89658	-0.18598	-2.65894
P	-2.15893	0.65174	-1.43170
O	-1.08356	1.91278	-1.40328
O	-2.21648	0.03897	-0.05800
O	-3.50071	1.58336	-1.68141
C	-4.56356	1.08113	-2.49906
H	-4.15307	0.58104	-3.38088
H	-5.14002	1.95148	-2.82564
C	-5.51483	0.14954	-1.77372
H	-6.33344	-0.08000	-2.47289
O	-4.85475	-1.07078	-1.39303
C	-5.50046	-1.63613	-0.24394
H	-5.95170	-2.58887	-0.52614
C	-6.11915	0.66954	-0.46708
H	-5.34121	1.13987	0.13993
C	-6.58281	-0.64065	0.19897
H	-6.67348	-0.56535	1.28500
O	-7.14177	1.62929	-0.64613
H	-7.82337	1.21557	-1.20517
O	-7.81423	-1.08327	-0.36792
H	-8.52063	-0.54129	0.02258
N	-4.51832	-1.92322	0.78758
C	-3.62613	-2.97926	0.74879
C	-4.11864	-1.13213	1.85139
C	-2.76286	-2.77789	1.83025
H	-4.62925	-0.21270	2.09553
N	-3.09934	-1.61940	2.51939
C	-1.75002	-3.73898	2.01047
N	-3.59514	-4.00561	-0.11399
C	-2.60961	-4.86124	0.17963
N	-1.70996	-4.79807	1.17393
H	-2.52189	-5.72564	-0.47309
N	-0.83885	-3.66608	3.02164
H	-0.65432	-2.73767	3.38368
H	-0.00886	-4.23446	2.89397

CA-3

E: -3463.48143
G: -3462.92358

O	-2.19781	-2.53895	-1.52913
C	-1.04797	-3.24476	-1.05097
H	-0.23316	-2.98105	-1.72506
H	-1.21017	-4.32608	-1.11106
C	-0.65823	-2.87170	0.38516
H	-1.34033	-3.31875	1.10692
O	0.67612	-3.41531	0.62448
C	1.56484	-2.34086	0.85369
H	1.67778	-2.17889	1.92982
C	-0.56771	-1.35862	0.63794
H	-1.27708	-0.79848	0.03517
C	0.88012	-1.11161	0.25255
H	0.98788	-1.08073	-0.82938
O	1.46165	0.06158	0.83964
P	-3.70788	-2.87189	-0.96018
O	-3.88727	-4.46565	-1.33597
O	-3.74890	-2.80332	0.54584
O	-4.61608	-2.01642	-1.81161
C	3.93121	-2.07708	1.15421
C	5.16365	-1.80635	0.66911
C	5.36252	-2.00924	-0.73326
N	2.92175	-2.53670	0.34180
C	3.15872	-2.70618	-1.05205
N	4.41026	-2.48449	-1.53340
C	2.46033	0.63019	0.16248
C	3.28305	1.58696	1.03098
C	3.88371	2.79433	0.35961
H	3.48380	2.91157	-0.64862
H	3.56199	3.66411	0.94384
C	5.42742	2.76689	0.37140
H	5.78430	3.71152	-0.05352
H	5.78749	2.70419	1.39849
C	6.07187	1.63216	-0.41087
O	6.96570	0.92427	0.09939
O	2.79594	0.37300	-0.97649
O	3.50098	1.26601	2.18748
O	2.21738	-3.04368	-1.78596
N	6.57785	-1.73807	-1.26729
H	6.63015	-1.72194	-2.27991
H	7.13447	-1.03508	-0.78544
H	5.94100	-1.42156	1.31376
H	3.67108	-1.92643	2.19482
H	-3.94888	-4.58740	-2.30040
O	6.38741	0.56545	2.83620
H	5.43829	0.76526	2.75573
H	6.70327	0.68805	1.91465

O	-1.33090	0.34850	4.00982
P	-1.49752	0.28332	2.51282
O	-0.75835	-1.11145	2.03368
O	-1.07494	1.44219	1.64390
O	-3.03490	-0.18229	2.13756
C	-4.13612	0.59064	2.63534
H	-3.80388	1.29695	3.40231
H	-4.84453	-0.10449	3.09787
C	-4.85307	1.31975	1.51871
H	-5.73276	1.81984	1.94842
O	-3.98589	2.30995	0.93476
C	-4.14046	2.34850	-0.48231
H	-4.36700	3.37324	-0.77892
C	-5.29556	0.42879	0.35173
H	-4.55856	-0.35377	0.18118
C	-5.29890	1.41040	-0.83211
H	-5.17397	0.91414	-1.79730
O	-6.53007	-0.22704	0.56852
H	-7.20409	0.46865	0.67071
O	-6.48671	2.20156	-0.82528
H	-7.19136	1.66294	-1.22261
N	-2.88504	1.99770	-1.13560
C	-1.80654	2.85651	-1.23589
C	-2.45577	0.78299	-1.64203
C	-0.79052	2.11187	-1.84310
H	-3.10257	-0.08207	-1.68037
N	-1.22058	0.81554	-2.09669
C	0.41344	2.79488	-2.10656
N	-1.73638	4.14045	-0.85480
C	-0.53775	4.66592	-1.13022
N	0.51872	4.08543	-1.72142
H	-0.39606	5.70322	-0.83892
N	1.44632	2.23044	-2.79104
H	1.51497	1.22089	-2.73972
H	2.33866	2.70196	-2.68927
N	5.68504	1.46986	-1.68625
H	4.86974	1.94549	-2.05002
H	6.01423	0.65865	-2.19595

CA-TS-3

E: -3463.43818
G: -3462.87825

O	1.76160	3.53856	-1.37126
C	0.49416	4.12227	-1.04570
H	-0.15066	3.95327	-1.91230
H	0.58819	5.20147	-0.88498
C	-0.12407	3.44874	0.17939
H	0.38960	3.75576	1.09001
O	-1.52441	3.84821	0.26882
C	-2.27362	2.67048	0.53262
H	-2.22328	2.40999	1.59494
C	-0.09207	1.90798	0.09374
H	0.61631	1.55330	-0.65135
C	-1.53463	1.62649	-0.29412
H	-1.65280	1.85668	-1.35199
O	-1.38120	-0.35324	-2.07633
P	3.12037	3.86523	-0.49149
O	3.42580	5.43064	-0.90991
O	2.83108	3.89727	0.98788
O	4.16544	2.93776	-1.06278
C	-4.55497	2.30160	1.16631
C	-5.61052	1.51237	0.85954
C	-5.60298	0.97081	-0.46841
N	-3.68854	2.75865	0.19249
C	-4.04538	2.53567	-1.18115
N	-4.92849	1.54800	-1.45936
C	-1.98699	-0.55945	-1.04240
C	-2.88397	-1.80526	-0.83479
C	-3.25413	-2.15504	0.61122
H	-4.11014	-1.50682	0.81807
H	-3.64349	-3.17561	0.60471
C	-2.22588	-1.95744	1.73670
H	-1.22871	-1.71733	1.36275
H	-2.11256	-2.88550	2.30740
C	-2.65841	-0.88107	2.72851
O	-3.84414	-0.52902	2.85827
O	-2.07605	0.32732	-0.03694
O	-3.89635	-1.74278	-1.66385
O	-3.47396	3.21090	-2.04835
N	-6.25083	-0.19627	-0.73332
H	-5.69386	-0.82739	-1.31522
H	-6.63186	-0.65868	0.08579
H	-6.27785	1.13804	1.62512
H	-4.30761	2.58182	2.18405
H	3.62178	5.49975	-1.86153
O	-4.28902	-4.38966	-1.84282
H	-4.38866	-3.39660	-1.84967
H	-4.51449	-4.63050	-0.92842
O	1.18916	-0.35829	2.92236
P	1.16684	0.04492	1.46539
O	0.21977	1.38925	1.39057
O	0.71729	-0.91797	0.40152
O	2.60093	0.70694	1.01592
C	3.81945	0.37470	1.68641
H	3.61842	0.14417	2.73584
H	4.45855	1.26066	1.63520
C	4.55070	-0.77585	1.02675
H	5.47534	-0.94834	1.59599
O	3.74668	-1.97387	1.06173
C	3.97094	-2.76163	-0.10868
H	4.28007	-3.76216	0.19538
C	4.91001	-0.59183	-0.45165
H	4.08204	-0.11503	-0.98229
C	5.07073	-2.05678	-0.90841
H	4.97417	-2.19231	-1.98811
O	6.04438	0.22186	-0.67118
H	6.78224	-0.16554	-0.16789
O	6.31120	-2.59061	-0.45391
H	7.00262	-2.25392	-1.04840
N	2.72062	-2.90456	-0.85857
C	1.59043	-3.52119	-0.36100
C	2.30097	-2.21686	-1.98611
C	0.54844	-3.16337	-1.24270
H	2.98585	-1.63448	-2.58448
N	1.02910	-2.37092	-2.26975
C	-0.72306	-3.54454	-0.83624
N	1.46066	-4.26905	0.73180
C	0.19543	-4.66030	0.94098
N	-0.89393	-4.33169	0.22646
H	0.02551	-5.30548	1.79661
N	-1.89578	-3.05227	-1.49616
H	-1.63381	-2.64315	-2.39845
H	-2.61178	-3.79682	-1.66945
N	-1.68290	-0.38102	3.51369
H	-0.68899	-0.51304	3.31169
H	-1.93661	0.36641	4.14866

CA-4

E: -3310.57063
G: -3310.06144

O	0.61918	3.10416	-1.26010
C	-0.62541	3.42491	-0.62113
H	-1.40352	3.21386	-1.35435
H	-0.66392	4.48993	-0.36969
C	-0.87616	2.58257	0.63843
H	-0.32014	2.97510	1.48821
O	-2.29868	2.64471	0.96624
C	-2.86386	1.34616	0.83919

H	-2.88676	0.85405	1.81551
C	-0.55393	1.09964	0.45073
H	0.26643	0.91507	-0.23830
C	-1.89624	0.59213	-0.06681
H	-2.03925	0.87685	-1.10810
O	-3.54622	-1.15692	-1.56976
P	2.05129	3.59806	-0.61115
O	1.94746	5.23784	-0.72665
O	2.10867	3.30024	0.86567
O	3.09525	3.06333	-1.56462
C	-5.14752	0.58956	1.05310
C	-6.39630	0.37829	0.57675
C	-6.71879	0.98504	-0.67873
N	-4.24401	1.36307	0.36990
C	-4.57683	1.88118	-0.91156
N	-5.83839	1.70376	-1.37769
C	-2.60951	-1.53597	-0.90481
C	-1.71579	-2.74879	-1.08850
C	-1.75053	-3.88601	-0.10766
H	-2.73972	-4.34748	-0.22987
H	-1.00272	-4.62163	-0.40281
C	-1.54982	-3.52929	1.37427
H	-0.57788	-3.05538	1.51785
H	-1.53830	-4.47101	1.93732
C	-2.65382	-2.67458	1.97573
O	-3.81373	-2.68621	1.52611
O	-1.98883	-0.82185	0.06340
O	-3.71024	2.48926	-1.56075
N	-7.95844	0.84481	-1.18107
H	-8.15709	1.18840	-2.11133
H	-8.62050	0.21791	-0.74524
H	-7.11374	-0.22371	1.11931
H	-4.80114	0.16336	1.98683
H	1.95986	5.51829	-1.65939
O	0.56213	-1.17288	3.36186
P	0.86198	-0.60450	1.98991
O	-0.29583	0.53313	1.74155
O	0.99624	-1.53399	0.81270
O	2.15535	0.41080	2.06381
C	3.40237	-0.05908	2.60090
H	3.25761	-0.98374	3.16799
H	3.76152	0.71045	3.29139
C	4.46677	-0.25484	1.53821
H	5.41456	-0.45327	2.05980
O	4.15215	-1.37952	0.69629
C	4.54634	-1.13065	-0.65520
H	5.22851	-1.91796	-0.97687
C	4.65797	0.92354	0.57649
H	3.69539	1.37597	0.34512
C	5.23455	0.23817	-0.67719
H	5.03799	0.79909	-1.59421
O	5.46602	1.96162	1.09635
H	6.34209	1.57549	1.27503
O	6.63318	0.00156	-0.52594
H	7.08709	0.84098	-0.71150
N	3.36615	-1.20522	-1.51175
C	2.65924	-2.37928	-1.71748
C	2.47261	-0.17854	-1.79535
C	1.37308	-1.97970	-2.11225
H	2.75450	0.86602	-1.72796
N	1.29302	-0.59651	-2.18854
C	0.39685	-2.98360	-2.10085
N	3.05590	-3.64195	-1.54325
C	2.06920	-4.52230	-1.76018
N	0.76603	-4.26805	-1.99063
H	2.34139	-5.57148	-1.69917
N	-0.91213	-2.49478	-2.05558
N	-2.30060	-1.96235	3.06602
H	-1.31381	-1.76545	3.25708
H	-2.98380	-1.30648	3.42814

CA-TS-4

E: -3443.56888
G: -3443.00578

O	-1.06779	-2.93654	-1.37353
C	0.20565	-3.32048	-0.83843
H	0.94459	-3.00095	-1.57742
H	0.26794	-4.40845	-0.73265
C	0.49260	-2.65133	0.50910
H	-0.14177	-3.07174	1.28793
O	1.88405	-2.91108	0.86409
C	2.60224	-1.68145	0.91512
H	2.69448	-1.31538	1.93637
C	0.34496	-1.13089	0.47499
H	-0.41108	-0.78266	-0.22518
C	1.75895	-0.73135	0.07063
H	1.90653	-0.92897	-0.99257
O	3.69464	0.91901	-1.16152
P	-2.47283	-3.54027	-0.75529
O	-2.31724	-5.15532	-1.04085
O	-2.52010	-3.39970	0.74509
O	-3.54728	-2.94320	-1.63349
C	2.72829	1.35404	-0.57806
C	1.92394	2.63304	-0.80401
C	2.00514	3.73825	0.21754
H	3.05083	4.04676	0.26561
H	1.41098	4.57956	-0.13256
C	1.54718	3.33617	1.63153
H	0.55361	2.88390	1.61402
H	1.47624	4.25430	2.22847
C	2.53928	2.42442	2.33821
O	3.74136	2.39964	2.02199
O	1.99383	0.65153	0.32075
H	-2.32402	-5.33846	-1.99738
O	-0.84199	0.87636	3.54510
P	-1.07253	0.45778	2.10844
O	0.08476	-0.66581	1.80464
O	-1.12512	1.50691	1.02858
O	-2.38518	-0.52993	1.99660
C	-3.64076	-0.09970	2.54659
H	-3.49483	0.74240	3.22972
H	-4.04298	-0.93905	3.12272
C	-4.66403	0.25767	1.48530
H	-5.60996	0.46219	2.00871
O	-4.27083	1.43837	0.76176
C	-4.61112	1.32767	-0.62243
H	-5.21544	2.18958	-0.90620
C	-4.90631	-0.81138	0.41462
H	-3.97655	-1.31510	0.15542
C	-5.39623	0.02371	-0.78435
H	-5.21369	-0.46902	-1.74387
O	-5.79873	-1.83475	0.81558
H	-6.59182	-1.40819	1.18445
O	-6.77359	0.36086	-0.64696
H	-7.27440	-0.47055	-0.70955
N	-3.38864	1.37622	-1.42073
C	-2.57818	2.49927	-1.48687
C	-2.56967	0.30766	-1.76434
C	-1.31227	2.02975	-1.86958
H	-2.93794	-0.70874	-1.80816
N	-1.34315	0.65797	-2.07047
C	-0.25266	2.93344	-1.71714
N	-2.87904	3.76702	-1.19808
C	-1.81060	4.57281	-1.28080
N	-0.52654	4.23008	-1.48956
H	-1.99668	5.63054	-1.11995
N	1.01144	2.35325	-1.67492
N	2.04814	1.69725	3.36417
H	1.04289	1.53414	3.46128

H	2.66698	0.99561	3.75533
N	3.67929	3.64261	-2.34525
H	4.62219	4.00426	-2.20391
H	3.18176	4.32874	-2.91134
H	3.76987	2.80171	-2.91184
O	1.72444	0.02302	-3.30254
H	1.33483	0.76605	-2.78677
H	2.64475	0.01449	-2.99320
C	4.14197	-2.46760	-0.80008
C	5.36562	-2.50845	-1.37756
C	6.43224	-1.86590	-0.66898
N	3.95451	-1.85637	0.40756
C	5.02023	-1.18576	1.06006
N	6.25488	-1.24394	0.49844
O	4.79729	-0.57583	2.12098
N	7.66832	-1.86417	-1.20088
H	8.43958	-1.49781	-0.65890
H	7.87448	-2.41883	-2.02012
H	5.53061	-3.00472	-2.32533
H	3.26525	-2.92787	-1.23913

CA-5

E: -3367.63879
G: -3367.08032

O	1.81881	-2.98754	-0.45787
C	0.47724	-3.14213	-0.92171
H	-0.13516	-3.29666	-0.03404
H	0.39379	-4.02753	-1.56084
C	-0.05239	-1.93438	-1.69876
H	0.48804	-1.81189	-2.63733
O	-1.44306	-2.22045	-2.00373
C	-2.29262	-1.28021	-1.37012
H	-2.61624	-0.52419	-2.09149
C	-0.04041	-0.60264	-0.93781
H	0.76038	-0.55139	-0.20274
C	-1.42914	-0.59950	-0.30056
H	-1.43048	-1.16322	0.62517
O	-3.63230	0.15351	1.25239
P	3.11419	-2.96941	-1.47248
O	2.89803	-4.34666	-2.34923
O	3.01595	-1.83354	-2.46053
O	4.30312	-3.09313	-0.54914
C	-4.70730	-1.49216	-1.38592
C	-5.88409	-1.91529	-0.86963
C	-5.81402	-2.80817	0.24691
N	-3.50857	-1.91308	-0.87251
C	-3.48126	-2.78115	0.25173
N	-4.65433	-3.21528	0.76948
C	-2.89495	0.96666	0.74009
C	-3.10300	2.45580	0.94496
C	-3.48900	3.33174	-0.19164
H	-4.34106	2.87763	-0.70953
H	-3.78786	4.31040	0.19145
C	-2.29765	3.46332	-1.15833
H	-1.39202	3.73468	-0.60907
H	-2.08950	2.50701	-1.64065
C	-2.54568	4.49794	-2.24417
O	-3.58797	5.16811	-2.31128
O	-1.85453	0.75226	-0.04563
O	-2.38222	-3.12081	0.72792
N	-6.94840	-3.26168	0.80298
H	-6.90512	-3.88577	1.59736
H	-7.85115	-2.97914	0.44804
H	-6.83113	-1.58882	-1.27943
H	-4.65131	-0.80813	-2.22420
H	2.97284	-5.13294	-1.77882
O	0.68649	2.76824	-2.67228
P	0.92547	1.81469	-1.52149
O	0.06126	0.46332	-1.88875
O	0.68839	2.26741	-0.10417
O	2.42333	1.14549	-1.65486
C	3.55926	2.00076	-1.44648
H	3.26042	3.05327	-1.41988
H	4.23313	1.86188	-2.29770
C	4.32532	1.64953	-0.18876
H	5.21689	2.29264	-0.16227
O	3.53341	1.90157	0.98877
C	3.78797	0.91560	1.98595
H	4.06731	1.42236	2.91092
C	4.75916	0.18726	-0.05989
H	3.95738	-0.46789	-0.39767
C	4.93355	0.04509	1.46188
H	4.89034	-0.99112	1.80353
O	5.90067	-0.14639	-0.82362
H	6.61561	0.45765	-0.55549
O	6.14274	0.66046	1.89939
H	6.87032	0.06023	1.66416
N	2.56348	0.15929	2.26046
C	1.41192	0.71752	2.78072
C	2.25685	-1.16521	1.99464
C	0.47743	-0.32156	2.81969
H	2.96706	-1.83717	1.54006
N	1.03066	-1.49704	2.33727
C	-0.82004	0.00513	3.26034
N	1.20634	1.98775	3.16814
C	-0.05525	2.18641	3.54207
N	-1.06634	1.29381	3.59757
H	-0.31763	3.19841	3.83783
N	-1.80988	-0.91327	3.39128
N	-1.52640	4.60517	-3.11510
H	-0.70086	4.00436	-3.02883
H	-1.60387	5.25804	-3.88463
H	-1.72366	-1.76831	2.85086
H	-2.75270	-0.54350	3.43537
N	-2.94218	2.89184	2.14182
H	-3.09456	3.87968	2.35137
H	-2.54317	2.28064	2.88815

CA-TS-5

E: -3556.93526
G: -3556.36227

O	-1.67005	-3.91285	-1.19118
C	-1.08226	-2.66988	-1.59913
H	-0.41091	-2.89538	-2.43325
H	-1.85674	-1.97830	-1.94139
C	-0.29117	-2.01425	-0.48586
H	-0.90939	-1.93262	0.40746
O	0.86532	-2.82880	-0.18235
C	1.92739	-1.96601	0.17560
H	1.83567	-1.65388	1.21816
C	0.23015	-0.61581	-0.90722
H	0.00955	-0.42652	-1.95828
C	1.75296	-0.73441	-0.71630
H	2.24799	-0.87271	-1.67294
O	4.24948	0.29517	-1.18704
P	-3.17663	-3.91391	-0.51716
O	-2.91363	-3.29650	0.98869
O	-4.06377	-2.91335	-1.21944
O	-3.55764	-5.36919	-0.42399
C	4.10693	-2.47308	1.08045
C	5.41807	-2.77762	0.94193
C	5.84066	-3.23209	-0.34842
N	3.22923	-2.60352	0.03504
C	3.69754	-3.01167	-1.24507
N	5.00387	-3.34824	-1.38254
C	3.50567	0.82370	-0.38265
C	3.89314	2.04795	0.42284
C	2.84587	2.77980	1.23500

H	2.29672	2.04791	1.82884
H	3.36027	3.45537	1.92284
C	1.85862	3.56773	0.36645
H	1.00433	3.85227	0.98971
H	1.44789	2.93927	-0.42901
C	2.44182	4.83972	-0.23029
O	3.53610	5.31380	0.13098
O	2.27721	0.42497	-0.06377
O	2.89378	-3.04855	-2.19220
N	7.12737	-3.56866	-0.53999
H	7.44532	-3.81878	-1.46682
H	7.81245	-3.42553	0.18898
H	6.11149	-2.66977	1.76590
H	3.69027	-2.10279	2.00794
H	-2.29864	-3.85827	1.49331
N	1.66963	5.42317	-1.16307
H	0.74206	5.04746	-1.39236
H	1.96376	6.31020	-1.55326
N	4.88891	2.74500	-0.12979
H	5.01360	3.71890	0.12812
H	5.47799	2.31958	-0.83586
C	5.21733	0.55070	2.21925
H	4.42539	1.23263	1.49397
O	6.34657	0.64235	1.75903
O	4.60295	0.02940	3.14150
O	0.27586	-0.24329	2.19098
P	-0.67335	0.59889	1.37166
O	-0.34654	0.49091	-0.23082
O	-0.83288	2.06885	1.66806
O	-2.15419	-0.12334	1.31484
C	-2.96824	-0.10189	2.49476
H	-2.60074	0.64282	3.20749
H	-2.94461	-1.08833	2.97023
C	-4.39341	0.23366	2.10460
H	-5.00886	0.24785	3.01268
O	-4.43745	1.55147	1.51326
C	-5.02581	1.50126	0.22296
H	-5.63169	2.39888	0.09922
C	-5.02135	-0.72001	1.07072
H	-4.25127	-1.18660	0.45931
C	-5.86918	0.22886	0.20120
H	-6.05358	-0.15838	-0.80395
O	-5.74907	-1.78732	1.64911
H	-6.44598	-1.39547	2.20477
O	-7.09776	0.55232	0.84679
H	-7.68377	-0.21612	0.74452
N	-3.99200	1.51755	-0.81807
C	-3.07898	2.54560	-0.97116
C	-3.57982	0.51349	-1.68033
C	-2.17956	2.10261	-1.94415
H	-4.10001	-0.42911	-1.75975
N	-2.52095	0.83481	-2.39001
C	-1.06840	2.92908	-2.19485
N	-3.03409	3.72114	-0.32066
C	-1.96677	4.43780	-0.67612
N	-1.00218	4.11827	-1.55741
H	-1.84994	5.40418	-0.19335
N	-0.07801	2.59245	-3.06045
H	0.01435	1.61036	-3.29073
H	0.79286	3.10260	-2.96643

CA-Gln

E: -3368.40422
G: -3367.83477

O	-1.40102	-3.20435	-0.58550
C	-0.14562	-3.50079	0.03940
H	0.59740	-3.50050	-0.75715
H	-0.16824	-4.49796	0.49145
C	0.25360	-2.46891	1.10158
H	-0.31820	-2.60909	2.01822
O	1.66150	-2.66834	1.40924
C	2.41008	-1.54192	0.96331
H	2.58863	-0.86392	1.80248
C	0.14182	-1.01882	0.63845
H	-0.70331	-0.83786	-0.01967
C	1.49391	-0.82267	-0.04025
H	1.52136	-1.29475	-1.01993
O	3.54824	0.24749	-1.50709
P	-2.82203	-3.48549	0.19757
O	-2.77349	-5.11301	0.44802
O	-2.81953	-2.86849	1.57450
O	-3.87971	-3.13334	-0.82245
C	4.81930	-1.35715	1.07412
C	6.06600	-1.53591	0.57876
C	6.18115	-2.34385	-0.59707
N	3.72663	-1.93480	0.48424
C	3.87759	-2.70217	-0.70011
N	5.12536	-2.90014	-1.19411
C	2.78416	0.98942	-0.91468
C	2.86451	2.50408	-0.90472
C	3.44323	3.01319	0.43826
H	4.44678	2.59790	0.58786
H	3.55449	4.09883	0.33875
C	2.52980	2.69530	1.62397
H	1.48490	2.84864	1.33971
H	2.60491	1.63746	1.89817
C	2.80674	3.49919	2.87896
O	3.85217	4.15031	3.05411
O	1.75886	0.57126	-0.15957
O	2.86712	-3.17610	-1.24574
N	7.39685	-2.57213	-1.12446
H	7.47400	-3.07085	-2.00082
H	8.21150	-2.09604	-0.76274
H	6.93124	-1.08692	1.04906
H	4.62288	-0.75754	1.95501
H	-2.81980	-5.59411	-0.39752
O	-0.55318	1.79624	3.21132
P	-0.86894	1.14373	1.88157
O	0.08937	-0.18668	1.80869
O	-0.80287	1.96097	0.61612
O	-2.30422	0.34491	1.97873
C	-3.49180	1.05856	2.35429
H	-3.25177	2.06932	2.69796
H	-3.95072	0.51604	3.18717
C	-4.49299	1.11408	1.21900
H	-5.41948	1.55839	1.60970
O	-3.99445	1.94020	0.14999
C	-4.27905	1.35728	-1.12052
H	-4.77327	2.10696	-1.73940
C	-4.81027	-0.23918	0.57106
H	-3.91541	-0.85791	0.54994
C	-5.19929	0.16262	-0.86204
H	-5.06942	-0.64874	-1.58164
O	-5.79230	-0.98880	1.26007
H	-6.60555	-0.45330	1.26855
O	-6.53632	0.65923	-0.90388
H	-7.12711	-0.11238	-0.89969
N	-3.03194	1.00540	-1.79394
C	-2.11992	1.94838	-2.23132
C	-2.40126	-0.22228	-1.92005
C	-0.99364	1.22468	-2.63354
H	-2.88102	-1.14801	-1.63177
N	-1.19646	-0.13634	-2.44376
C	0.11770	1.97862	-3.06158
N	-2.25895	3.28242	-2.24457
C	-1.14304	3.88261	-2.67228
N	0.01230	3.32732	-3.06939
H	-1.17097	4.96884	-2.70296
N	1.28092	1.41927	-3.48961

N	1.82265	3.42870	3.79827
H	0.97834	2.87431	3.62858
H	1.94936	3.89136	4.68948
H	1.41115	0.43822	-3.27240
H	2.12069	1.99630	-3.37965
H	1.83975	2.87867	-0.98220
N	3.59608	2.92570	-2.10233
H	3.71701	3.93687	-2.05673
H	4.53249	2.52220	-2.06779

AA-1

E: -4006.80331
G: -4006.29758

O	2.44630	-2.28705	-1.49811
C	1.59927	-3.38543	-1.85063
H	2.23805	-4.25052	-2.04568
H	1.00527	-3.16819	-2.74260
C	0.67826	-3.66751	-0.67553
H	0.13014	-4.59200	-0.87247
O	1.43742	-3.86741	0.54534
C	1.41138	-2.70977	1.35042
H	1.03429	-2.96830	2.34343
C	-0.30095	-2.53464	-0.35479
H	-0.55858	-1.95794	-1.24000
C	0.47046	-1.69305	0.66814
H	1.02914	-0.90579	0.16885
O	-0.38750	-1.12311	1.65815
P	2.68487	-0.92220	-2.32399
O	3.82373	-1.03904	-3.28760
O	1.30273	-0.58250	-3.04366
O	2.87393	0.03093	-1.07957
C	-0.49623	0.20941	1.71283
C	-1.26802	0.63384	2.97308
C	-1.14258	2.06685	3.35343
H	-0.19009	2.48727	3.02891
H	-1.22637	2.14389	4.44267
C	-2.31617	2.93450	2.75813
O	-2.07124	4.16132	2.66252
O	-0.04174	0.99966	0.91292
O	-1.95318	-0.18381	3.56920
O	-3.39025	2.33474	2.48666
P	3.44049	1.69628	-1.18341
O	4.92803	1.58269	-0.87199
O	3.11583	2.04778	-2.64735
O	2.56596	2.35711	-0.12538
H	0.80491	0.24781	-2.72055
O	-1.47299	-3.05141	0.26474
P	-2.79514	-3.36319	-0.67609
O	-2.83759	-4.82864	-1.03425
O	-2.87802	-2.33336	-1.78041
O	-3.95168	-3.15578	0.46192
C	-3.95496	-2.11427	1.45212
H	-3.00745	-2.11020	1.99506
H	-4.75366	-2.39130	2.14446
C	-4.25062	-0.71813	0.93687
H	-4.57554	-0.10994	1.79024
O	-3.05272	-0.12353	0.39840
C	-3.49142	0.93032	-0.44272
H	-3.84203	1.78163	0.14888
C	-5.32614	-0.60359	-0.17242
H	-5.54013	-1.58299	-0.60649
C	-4.66142	0.33369	-1.22601
H	-4.29585	-0.24344	-2.07627
O	-6.53000	-0.03607	0.32275
H	-6.64313	0.79116	-0.19056
O	-5.52575	1.38168	-1.63990
H	-6.05894	1.05208	-2.38315
N	-2.36851	1.40090	-1.21628
C	-1.84870	2.68242	-1.16494
C	-1.45548	0.63429	-1.89653
C	-0.63218	2.63047	-1.85072
H	-1.62706	-0.41850	-2.06930
N	-0.41082	1.32836	-2.30458
C	0.06820	3.85594	-1.96984
N	-2.41514	3.75802	-0.60603
C	-1.64292	4.84005	-0.74176
N	-0.47099	4.95245	-1.37860
H	-2.01860	5.75194	-0.28477
N	1.20444	4.00598	-2.68294
H	1.85664	3.19936	-2.74491
H	1.65869	4.90163	-2.54029
N	2.77161	-2.17221	1.50113
C	3.08905	-0.94777	2.05416
C	3.93226	-2.64676	0.92767
C	4.45051	-0.76379	1.79145
H	3.95226	-3.59601	0.41783
N	4.96642	-1.84822	1.09275
C	5.00941	0.45336	2.22547
N	2.28081	-0.09049	2.69758
C	2.92187	1.03121	3.03825
N	4.21365	1.34569	2.85244
H	2.32214	1.78531	3.54153
N	6.33075	0.74922	2.07397
H	6.80808	0.26475	1.32293
H	6.56159	1.73362	2.14682

AA-TS-1

E: -4006.76222
G: -4006.25500

O	-4.36340	1.63854	-0.23440
C	-4.12385	2.99795	-0.56709
H	-4.91245	3.60603	-0.10881
H	-4.15064	3.17024	-1.64918
C	-2.77515	3.49080	-0.02889
H	-2.76303	4.58308	-0.06704
O	-2.62632	3.10055	1.37366
C	-1.62599	2.10860	1.48823
H	-0.72273	2.51151	1.95177
C	-1.52816	2.93918	-0.73495
H	-1.68288	2.77602	-1.80051
C	-1.37388	1.65224	0.04884
H	-2.18476	0.99378	-0.24385
O	-0.16588	0.88999	-0.02293
P	-4.62325	0.47580	-1.43778
O	-6.02392	0.73691	-1.98973
O	-3.47819	0.62116	-2.43768
O	-4.57228	-0.81622	-0.55531
C	-0.12807	0.00833	-1.04975
C	0.35435	-1.37327	-0.59092
C	-0.03214	-2.55082	-1.46166
H	-0.79502	-2.26766	-2.18411
H	0.85763	-2.89030	-1.99495
C	-0.49537	-3.69306	-0.55466
O	-1.71191	-3.66310	-0.08762
O	-0.44355	0.27147	-2.19000
O	1.02961	-1.49550	0.41663
O	0.29972	-4.59583	-0.26001
P	-2.99667	-2.24869	-0.46342
O	-3.84859	-2.83516	0.79335
O	-3.36921	-2.78973	-1.81973
O	-2.01749	-1.14624	-0.11532
N	-2.06695	1.00060	2.33940
C	-1.16560	0.07722	2.85667
C	-3.27335	0.33789	2.24293
C	-1.89568	-1.09712	3.04627
H	-4.13875	0.81769	1.81778
N	-3.22826	-0.89905	2.69288
C	-1.15339	-2.24279	3.38646

N	0.13662	0.25114	3.11584
C	0.71571	-0.88562	3.51460
N	0.16425	-2.10284	3.64754
H	1.77540	-0.82291	3.74724
N	-1.71611	-3.48138	3.47644
H	-2.54428	-3.62921	2.90950
H	-1.05540	-4.25006	3.44689
H	-3.83437	-2.15202	1.52021
O	-0.45598	3.86301	-0.51069
P	0.90895	3.87527	-1.44265
O	1.38425	5.30640	-1.41346
O	0.68290	3.13424	-2.73426
O	1.94964	2.93825	-0.57928
C	2.20536	3.26376	0.79013
H	1.28643	3.15554	1.37264
H	2.56003	4.29714	0.88104
C	3.27247	2.34309	1.33477
H	3.41984	2.58962	2.39491
O	2.84114	0.97117	1.22406
C	3.93237	0.12641	0.89640
H	4.01773	-0.68022	1.62629
C	4.63080	2.40179	0.61964
H	4.48954	2.54431	-0.45416
C	5.19536	0.99880	0.90311
H	5.92887	0.66500	0.16624
O	5.45860	3.45701	1.06787
H	5.68373	3.24911	1.99340
O	5.73926	0.95317	2.22262
H	6.66990	1.22500	2.16427
N	3.68781	-0.51634	-0.39086
C	3.80713	-1.86860	-0.64120
C	3.17246	0.04900	-1.54040
C	3.39311	-2.03569	-1.96583
H	2.92850	1.09984	-1.59431
N	2.99262	-0.82369	-2.51251
C	3.40136	-3.35491	-2.46344
N	4.21404	-2.83625	0.19559
C	4.16511	-4.03783	-0.38950
N	3.80232	-4.35137	-1.64325
H	4.46871	-4.87809	0.22933
N	2.98452	-3.66633	-3.71190
H	2.87589	-2.92635	-4.39189
H	3.17395	-4.59718	-4.05979

AA-2

E: -4006.78096
G: -4006.27860

O	-1.12299	2.26490	1.79268
C	0.05502	2.70211	1.10322
H	0.91250	2.38063	1.70194
H	0.08668	3.79167	1.00998
C	0.10160	2.06177	-0.28150
H	-0.76703	2.37060	-0.86228
O	1.29491	2.52776	-0.96692
C	2.18208	1.45048	-1.19321
H	2.15035	1.11899	-2.23534
C	0.19685	0.53533	-0.22326
H	-0.24416	0.13475	0.68805
C	1.70806	0.32938	-0.26658
H	2.14079	0.44158	0.72962
O	2.15108	-0.91001	-0.82641
P	-2.50058	3.16598	1.68203
O	-2.17253	4.43132	2.68717
O	-2.62985	3.77817	0.31000
O	-3.57852	2.29210	2.27516
C	2.10779	-1.98361	-0.03373
C	2.93610	-3.13997	-0.62175
C	4.27548	-2.79314	-1.22203
H	4.73165	-3.68570	-1.64999
H	4.13287	-2.03497	-2.00191
C	5.20874	-2.18299	-0.17077
O	6.38645	-2.50336	-0.08531
O	1.49640	-2.07239	1.00949
O	2.50133	-4.27152	-0.52191
O	4.58074	-1.30858	0.58571
P	5.18794	-0.70123	2.17932
O	4.05105	0.28053	2.45800
O	6.54798	-0.08878	1.86552
O	5.19808	-1.98003	3.00704
H	-2.14011	4.13121	3.61337
O	-2.09145	-1.54710	-2.48606
P	-1.79766	-0.91451	-1.15165
O	-0.38505	-0.07461	-1.37545
O	-1.70286	-1.72549	0.11289
O	-2.85067	0.31047	-0.83208
C	-3.29136	1.13569	-1.92322
H	-2.99003	0.69131	-2.87432
H	-2.83679	2.12642	-1.83203
C	-4.80025	1.28187	-1.88425
H	-5.11061	1.82722	-2.78454
O	-5.42429	-0.02094	-1.91336
C	-6.28407	-0.18297	-0.79466
H	-7.15158	-0.75416	-1.12514
C	-5.36102	2.00607	-0.64376
H	-4.67361	1.92652	0.19730
C	-6.65870	1.22763	-0.35006
H	-6.97296	1.28404	0.69494
O	-5.56110	3.39288	-0.84789
H	-6.21941	3.47905	-1.56063
O	-7.70832	1.65994	-1.21352
H	-8.08149	2.46928	-0.82666
N	-5.62827	-0.95181	0.26421
C	-5.23303	-2.26857	0.11668
C	-5.10678	-0.53418	1.47790
C	-4.52755	-2.57853	1.28039
H	-5.23833	0.47098	1.84488
N	-4.46124	-1.47880	2.12469
C	-3.98570	-3.87664	1.35889
N	-5.46380	-3.09057	-0.92036
C	-4.91449	-4.29309	-0.71832
N	-4.20439	-4.73022	0.33385
H	-5.05902	-5.01912	-1.51412
N	-3.22178	-4.28662	2.39708
H	-3.22022	-3.73905	3.24684
H	-3.02597	-5.27558	2.48094
N	3.54266	1.87697	-0.92029
C	4.68197	1.31730	-1.46639
C	3.96676	2.68024	0.12139
C	5.74224	1.85545	-0.73125
H	3.25863	3.22739	0.72577
N	5.27440	2.72142	0.24915
C	7.02942	1.38561	-1.05798
N	4.77807	0.44144	-2.47946
C	6.04938	0.09228	-2.70604
N	7.15845	0.50095	-2.06967
H	6.20865	-0.62019	-3.51095
N	8.14870	1.81774	-0.41852
H	8.01683	2.17098	0.52182
H	8.97310	1.24169	-0.54386

AA-TS-2

E: -4139.76586
G: -4139.20244

O	-0.20641	4.73925	1.24282
C	0.83163	4.36628	0.33006
H	1.64444	3.94675	0.93017
H	1.21819	5.23545	-0.21118
C	0.32074	3.32867	-0.66588

H	-0.50763	3.73925	-1.24414
O	1.40626	3.01123	-1.57832
C	1.78830	1.65770	-1.42667
H	1.38140	1.03584	-2.22931
C	-0.09493	2.01189	-0.00814
H	-0.45143	2.16107	1.01091
C	1.21367	1.22010	-0.07968
H	1.88314	1.49805	0.73676
O	1.06408	-0.19811	-0.11036
P	-1.20950	5.99291	0.85212
O	-0.16803	7.27104	0.86329
O	-1.68459	5.87852	-0.57427
O	-2.17659	6.07712	2.00515
C	0.80869	-0.79260	1.05933
C	0.77921	-2.32440	0.94486
C	1.69977	-2.98646	-0.03661
H	1.42763	-4.03637	-0.15276
H	1.66187	-2.47945	-1.00534
C	3.17396	-2.87833	0.44465
O	4.05926	-3.48100	-0.21128
O	0.63560	-0.21527	2.11365
O	0.09673	-2.93216	1.75348
O	3.34970	-1.58069	0.89154
P	4.65883	-0.95787	1.83416
O	4.22774	0.50821	1.91054
O	5.94107	-1.24280	1.04993
O	4.54801	-1.73246	3.16458
H	0.17661	7.42291	1.76152
N	3.23205	1.54896	-1.49876
C	3.92526	0.45108	-1.96456
C	4.16480	2.36988	-0.89374
C	5.26328	0.70037	-1.64372
H	3.87069	3.30049	-0.43103
N	5.39495	1.91417	-0.98215
C	6.18238	-0.31070	-1.98004
N	3.43896	-0.63552	-2.58606
C	4.40313	-1.51536	-2.86089
N	5.72076	-1.41034	-2.61516
H	4.09240	-2.43341	-3.35104
N	7.51590	-0.21749	-1.73008
H	7.77678	0.42388	-0.98992
H	8.00219	-1.10732	-1.69200
N	3.06294	-3.79925	2.09538
H	3.49636	-4.70804	1.94511
H	2.13123	-3.92672	2.48943
H	3.64258	-3.20340	2.71674
O	6.72766	-3.56163	-0.99591
H	6.52944	-2.96044	-1.74427
H	5.90411	-3.46985	-0.47070
O	-1.73271	-0.95192	0.11305
P	-2.25321	0.45521	-0.05239
O	-1.10027	1.38732	-0.80898
O	-2.80766	1.21759	1.12168
O	-3.37845	0.51423	-1.24251
C	-3.11210	-0.13464	-2.49772
H	-2.12208	-0.59779	-2.49272
H	-3.13944	0.62489	-3.28593
C	-4.16362	-1.18638	-2.78998
H	-3.94977	-1.61499	-3.77754
O	-4.08773	-2.24645	-1.81346
C	-5.35715	-2.48124	-1.21993
H	-5.48327	-3.55856	-1.11390
C	-5.61895	-0.67860	-2.76639
H	-5.70839	0.19042	-2.11196
C	-6.38733	-1.87838	-2.17158
H	-7.31929	-1.59600	-1.67648
O	-6.09410	-0.26819	-4.03409
H	-6.07685	-1.05784	-4.60434
O	-6.63246	-2.85417	-3.18115
H	-7.43064	-2.57677	-3.66098
N	-5.40669	-1.89884	0.12203
C	-4.56822	-2.29826	1.14847
C	-6.01469	-0.73450	0.56418
C	-4.73968	-1.35198	2.15990
H	-6.73195	-0.19864	-0.03885
N	-5.66683	-0.38853	1.78263
C	-3.92911	-1.50261	3.30305
N	-3.75405	-3.36533	1.18206
C	-3.05242	-3.40492	2.31958
N	-3.08345	-2.55538	3.35857
H	-2.36507	-4.23959	2.42299
N	-3.93359	-0.61538	4.32235
H	-4.67948	0.06385	4.38150
H	-3.45899	-0.86825	5.17889

AA-3

E: -3420.10096
G: -3419.58381

O	3.53934	3.88355	0.65320
C	4.07541	2.61490	0.26373
H	4.26346	2.05043	1.18170
H	5.02489	2.72692	-0.26857
C	3.08042	1.87671	-0.62454
H	2.85068	2.47680	-1.50627
O	3.70283	0.63119	-1.05103
C	2.89528	-0.45486	-0.63102
H	2.17972	-0.74734	-1.40440
C	1.78162	1.48742	0.09470
H	1.52307	2.18640	0.89093
C	2.15309	0.08815	0.58737
H	2.81025	0.14060	1.45794
O	1.05000	-0.77109	0.86093
P	3.87187	5.20458	-0.28380
O	5.48889	5.39914	-0.02603
O	3.74213	4.86652	-1.74753
O	3.08821	6.32609	0.34791
C	0.34889	-0.52637	1.97965
C	-0.86213	-1.46947	2.09286
C	-1.11202	-2.48811	1.00430
H	-2.17686	-2.71725	0.98711
H	-0.81099	-2.09211	0.03168
C	-0.36065	-3.80652	1.23680
O	-0.93663	-4.89037	1.12044
O	0.65293	0.27487	2.83521
O	-1.55180	-1.37238	3.08891
H	5.66126	5.64297	0.90101
N	3.70126	-1.61958	-0.35829
C	3.32331	-2.91768	-0.65374
C	4.77680	-1.71540	0.50637
C	4.22406	-3.72711	0.04178
H	5.25762	-0.83008	0.89727
N	5.13467	-2.95516	0.75595
C	4.02816	-5.11963	-0.07359
N	2.30328	-3.32862	-1.42274
C	2.21598	-4.66313	-1.44116
N	2.99868	-5.56595	-0.82916
H	1.40306	-5.07656	-2.03188
N	4.84527	-6.02286	0.51354
H	5.46927	-5.70822	1.24449
H	4.52883	-6.98305	0.55970
N	0.94056	-3.70019	1.59885
H	1.44473	-2.84210	1.41025
H	1.49292	-4.55098	1.57876
O	-1.32260	0.77816	0.58554
P	-0.83866	1.70917	-0.50520
O	0.74456	1.38655	-0.88127
O	-1.03536	3.19496	-0.36312
O	-1.44982	1.28438	-1.96063
C	-1.24034	-0.03874	-2.48564

H	-0.57460	-0.61721	-1.83984
H	-0.76412	0.06874	-3.46538
C	-2.55685	-0.76893	-2.65799
H	-2.35951	-1.70317	-3.19967
O	-3.10768	-1.10120	-1.36934
C	-4.46382	-0.68978	-1.26088
H	-5.03806	-1.51221	-0.83515
C	-3.63998	0.03407	-3.40446
H	-3.46290	1.10468	-3.29215
C	-4.94225	-0.36666	-2.67689
H	-5.70153	0.41997	-2.69635
O	-3.66421	-0.21561	-4.79721
H	-3.85931	-1.16379	-4.90582
O	-5.45704	-1.58119	-3.21668
H	-5.88757	-1.35948	-4.05953
N	-4.58204	0.41790	-0.32090
C	-4.55626	0.24589	1.05153
C	-4.43489	1.77859	-0.53374
C	-4.44069	1.52971	1.58730
H	-4.41109	2.19930	-1.52763
N	-4.37687	2.48050	0.57591
C	-4.39626	1.61965	2.99307
N	-4.63739	-0.90314	1.73913
C	-4.57745	-0.68670	3.05785
N	-4.47103	0.47852	3.71460
H	-4.62976	-1.57161	3.68598
N	-4.24148	2.79410	3.64724
H	-4.40288	3.65495	3.14220
H	-4.41936	2.80402	4.64336

AA-TS-3

E: -3496.50069
G: -3495.95757

O	2.97272	4.36954	1.02816
C	3.23790	3.43317	-0.02212
H	3.87065	2.65110	0.40629
H	3.78771	3.90140	-0.84445
C	1.93427	2.85040	-0.55989
H	1.31279	3.65239	-0.95842
O	2.24959	1.93823	-1.65126
C	1.91971	0.60935	-1.28117
H	0.97864	0.28579	-1.73087
C	1.12346	2.02750	0.45013
H	1.21753	2.38584	1.47595
C	1.80015	0.66721	0.23819
H	2.79712	0.72952	0.67919
O	1.18048	-0.52787	0.72188
P	2.74417	5.96213	0.64996
O	4.21900	6.38067	0.04453
O	1.78599	6.10886	-0.50512
O	2.49176	6.63170	1.97672
C	1.36556	-0.80051	2.02540
C	0.88575	-2.22492	2.44417
C	1.39557	-3.30306	1.47678
H	0.93292	-4.25212	1.76989
H	1.12424	-3.09992	0.44546
C	2.91011	-3.48715	1.53035
O	3.54286	-3.79550	0.50797
O	1.91318	-0.04576	2.80109
O	1.03988	-2.44320	3.70916
H	4.90951	6.30288	0.72691
N	2.94245	-0.30385	-1.74422
C	2.71955	-1.60584	-2.14721
C	4.31272	-0.15351	-1.65306
C	3.99114	-2.16126	-2.31030
H	4.75174	0.78473	-1.34642
N	4.97875	-1.23380	-1.99971
C	4.03235	-3.51493	-2.70333
N	1.54413	-2.22541	-2.33342
C	1.71765	-3.50231	-2.69001
N	2.86507	-4.17022	-2.88894
H	0.80865	-4.07742	-2.84742
N	5.19275	-4.17452	-2.93457
H	6.04811	-3.77958	-2.56595
H	5.14702	-5.18425	-2.98892
N	3.48814	-3.34924	2.73661
H	2.91973	-3.03880	3.52055
H	4.49284	-3.43822	2.82088
O	-1.34591	-0.05353	-0.72501
P	-1.38572	0.98741	0.35996
O	-0.23834	2.12603	0.01770
O	-1.29730	0.63536	1.83146
O	-2.72711	1.91549	0.19560
C	-3.14992	2.33453	-1.11659
H	-2.37032	2.13522	-1.85591
H	-3.31813	3.41478	-1.07078
C	-4.44717	1.67748	-1.55780
H	-4.73516	2.15101	-2.50644
O	-4.26502	0.26766	-1.80296
C	-5.29948	-0.50034	-1.20171
H	-5.63827	-1.24649	-1.91923
C	-5.62103	1.78662	-0.56972
H	-5.25163	1.79947	0.45764
C	-6.40919	0.48241	-0.83196
H	-7.00407	0.15950	0.02777
O	-6.38625	2.96733	-0.71952
H	-6.62839	3.04739	-1.65910
O	-7.22827	0.61136	-1.98774
H	-7.92396	1.25493	-1.76949
N	-4.74892	-1.21704	-0.03993
C	-3.63196	-2.02891	-0.16280
C	-4.73582	-0.78000	1.27893
C	-3.00206	-1.99715	1.09331
H	-5.51700	-0.14737	1.67294
N	-3.73973	-1.24330	1.99383
C	-1.69910	-2.48314	1.10679
N	-3.17521	-2.66113	-1.24186
C	-1.99261	-3.24849	-1.02905
N	-1.22300	-3.16007	0.07235
H	-1.58432	-3.83323	-1.84722
N	-0.85032	-2.08763	2.21995
H	-1.05404	-1.06701	2.33654
H	-1.14873	-2.58845	3.07138
O	-0.85102	-4.35465	4.09260
H	-0.01582	-3.82026	4.07998
H	-0.80021	-4.85931	3.26348

AA-4

E: -3343.62678
G: -3343.13316

O	0.01021	4.33551	0.55584
C	1.41004	4.13078	0.33334
H	1.89080	4.22182	1.31136
H	1.81582	4.90029	-0.33070
C	1.67932	2.73358	-0.21333
H	1.42899	2.66784	-1.27660
O	3.07548	2.44032	-0.03275
C	3.19992	1.02608	-0.06339
H	3.15941	0.63815	-1.08641
C	0.91190	1.62448	0.52600
H	0.55340	1.96416	1.49789
C	1.98536	0.51257	0.73122
H	2.22379	0.37648	1.78523
O	1.53775	-0.71913	0.17397
P	-1.00426	4.74137	-0.68242
O	-0.37562	6.18258	-1.17909
O	-0.84964	3.81187	-1.85854
O	-2.34268	4.93997	-0.01303

C	1.52707	-1.85501	0.89344
C	0.64878	-2.82583	0.12309
C	0.75278	-2.82997	-1.39113
H	0.70816	-3.86612	-1.73288
H	-0.11686	-2.30695	-1.79726
C	2.00691	-2.17684	-1.95406
O	1.94390	-1.26382	-2.78812
O	2.03226	-2.01585	1.98230
H	-0.47047	6.85862	-0.48430
N	4.48159	0.66691	0.49018
C	5.47871	-0.08335	-0.10470
C	4.96475	1.03869	1.73253
C	6.51443	-0.11939	0.83214
H	4.37295	1.64773	2.40069
N	6.17502	0.59179	1.97686
C	7.66842	-0.84415	0.46793
N	5.48229	-0.66992	-1.31375
C	6.62745	-1.32818	-1.53981
N	7.69720	-1.44813	-0.74311
H	6.69623	-1.83670	-2.49758
N	8.75443	-0.93331	1.26372
H	8.67742	-0.66156	2.23442
H	9.46854	-1.60827	1.02204
N	3.18687	-2.68522	-1.52983
H	3.21702	-3.36549	-0.78053
H	4.02037	-2.11341	-1.67224
O	-1.64770	-0.74508	-0.77357
P	-1.35059	0.24154	0.32569
O	-0.17296	1.22946	-0.29655
O	-0.95138	-0.13888	1.72597
O	-2.63365	1.25898	0.50610
C	-3.49069	1.59739	-0.59581
H	-2.98251	1.43584	-1.55011
H	-3.70824	2.66425	-0.49913
C	-4.81665	0.84872	-0.57932
H	-5.46843	1.34098	-1.31506
O	-4.65807	-0.53053	-0.97256
C	-5.45681	-1.39871	-0.17321
H	-6.04917	-2.03624	-0.82905
C	-5.54035	0.80809	0.77564
H	-4.81178	0.75003	1.58641
C	-6.35002	-0.50548	0.68963
H	-6.56916	-0.93923	1.66910
O	-6.32739	1.95477	1.03477
H	-6.97411	2.02766	0.31011
O	-7.55135	-0.30384	-0.04969
H	-8.17469	0.15805	0.53580
N	-4.57542	-2.27826	0.60340
C	-3.69923	-3.16101	-0.01291
C	-3.98631	-1.98073	1.82715
C	-2.61070	-3.29648	0.87185
H	-4.47439	-1.33841	2.54553
N	-2.84608	-2.58749	2.04026
C	-1.44905	-3.81584	0.28791
N	-3.80154	-3.73282	-1.21149
C	-2.71597	-4.45749	-1.52207
N	-1.54287	-4.48802	-0.87002
H	-2.76921	-5.03861	-2.43739
N	-0.24218	-3.38223	0.86071

AA-TS-4

E: -3476.62244
G: -3476.06795

O	0.41543	4.40008	-0.85803
C	-1.00469	4.31550	-0.68838
H	-1.43053	4.29960	-1.69544
H	-1.38643	5.19377	-0.15856
C	-1.39464	3.02585	0.02621
H	-1.21572	3.09997	1.10293
O	-2.79322	2.77646	-0.20899
C	-2.98342	1.38129	-0.00691
H	-2.97862	1.12695	1.05784
C	-0.64574	1.78817	-0.49993
H	-0.16037	1.99065	-1.45352
C	-1.77359	0.74047	-0.70334
H	-1.96516	0.57761	-1.76235
O	-1.43017	-0.49846	-0.08420
P	1.39946	4.88330	0.37706
O	0.88061	6.42733	0.63686
O	1.09651	4.14286	1.65427
O	2.78358	4.87129	-0.22522
C	-1.70688	-1.66175	-0.68960
C	-1.02893	-2.76977	0.12946
C	-1.10140	-2.58313	1.64979
H	-1.18394	-3.56053	2.12775
H	-0.15599	-2.14487	1.96988
C	-2.19402	-1.66094	2.17612
O	-1.91008	-0.68769	2.88938
O	-2.31396	-1.77543	-1.73585
H	1.04933	6.98450	-0.14386
N	-3.47143	-1.97368	1.87354
H	-3.68919	-2.72590	1.23768
H	-4.18883	-1.26797	2.03128
O	1.77635	-0.49261	1.21396
P	1.54448	0.39542	0.01779
O	0.31064	1.40324	0.47339
O	1.25090	-0.10570	-1.37053
O	2.80904	1.43349	-0.17045
C	3.82303	1.62554	0.82227
H	3.42166	1.46683	1.82695
H	4.13460	2.66923	0.73287
C	5.05160	0.74905	0.61528
H	5.85545	1.17679	1.23228
O	4.81642	-0.60555	1.05040
C	5.49524	-1.54398	0.21213
H	6.12395	-2.17857	0.83701
C	5.55326	0.60947	-0.82720
H	4.70271	0.51163	-1.50579
C	6.33120	-0.72260	-0.77104
H	6.43816	-1.19965	-1.74885
O	6.30957	1.71246	-1.28766
H	7.03020	1.85689	-0.64946
O	7.60841	-0.53765	-0.16731
H	8.16710	-0.07772	-0.81669
N	4.50187	-2.40168	-0.43952
C	3.54953	-3.09181	0.30088
C	3.92805	-2.22701	-1.69377
C	2.44400	-3.24857	-0.54639
H	4.46201	-1.74030	-2.49682
N	2.73165	-2.74661	-1.81024
C	1.22547	-3.54598	0.10377
N	3.62600	-3.47094	1.58239
C	2.47235	-4.00391	2.00437
N	1.29520	-4.04101	1.36297
H	2.47769	-4.42285	3.00743
N	0.06005	-3.18182	-0.52060
N	-2.38341	-4.21362	-0.23978
H	-2.08026	-4.46545	-1.18745
H	-3.39197	-4.05408	-0.25825
H	-2.17398	-4.97889	0.40106
O	-1.03100	-4.11602	-2.93271
H	-1.65054	-3.36707	-2.99911
H	-0.42035	-3.78291	-2.22804
N	-4.26136	0.98938	-0.54155
C	-5.35331	0.52027	0.16588
C	-4.63699	0.99288	-1.87410
C	-6.33020	0.26424	-0.79816
H	-3.96211	1.33383	-2.64589
N	-5.86364	0.56944	-2.07159

C	-7.55906	-0.24518	-0.32905
N	-5.47479	0.33233	1.49069
C	-6.68114	-0.15742	1.80925
N	-7.70821	-0.44982	1.00063
H	-6.84865	-0.34528	2.86623
N	-8.59958	-0.50621	-1.14783
H	-8.43906	-0.54990	-2.14519
H	-9.37725	-1.03628	-0.77627

AA-5

E: -3400.69724
G: -3400.15313

O	-0.30172	3.76526	1.26989
C	0.94295	3.79114	0.56468
H	1.72531	3.63102	1.31176
H	1.11323	4.76141	0.08733
C	0.99129	2.67800	-0.48308
H	0.30477	2.89345	-1.30110
O	2.34118	2.61094	-1.02080
C	2.92508	1.35902	-0.70325
H	2.85535	0.65950	-1.54142
C	0.69844	1.30036	0.10560
H	0.01716	1.34557	0.95402
C	2.10855	0.83783	0.47788
H	2.44481	1.27192	1.42024
O	2.20867	-0.58812	0.51297
P	-1.63922	4.48851	0.62531
O	-1.33477	6.08371	0.90570
O	-1.64986	4.35503	-0.87744
O	-2.77925	3.99754	1.48069
C	1.74737	-1.19668	1.60190
C	1.49059	-2.66165	1.32048
C	1.68260	-3.26716	-0.01919
H	0.94920	-4.06284	-0.16728
H	1.57577	-2.51559	-0.79712
C	3.08307	-3.92418	-0.03752
O	3.25695	-5.01468	0.51196
O	1.59285	-0.71174	2.70159
H	-1.34145	6.27573	1.86038
N	4.33121	1.53494	-0.42009
C	5.33844	0.65205	-0.75392
C	4.91634	2.52603	0.34465
C	6.49507	1.18616	-0.18012
H	4.33209	3.34569	0.73674
N	6.21134	2.36269	0.50274
C	7.68649	0.45827	-0.38034
N	5.25088	-0.48322	-1.46718
C	6.44426	-1.08025	-1.58401
N	7.62973	-0.68890	-1.09637
H	6.45472	-2.00854	-2.14920
N	8.88326	0.87509	0.08581
H	8.90931	1.60672	0.78317
H	9.65367	0.21928	0.07008
N	4.03660	-3.22451	-0.66604
H	3.90262	-2.27406	-1.00059
H	4.98585	-3.58066	-0.65417
O	-1.03146	-1.56139	-1.77990
P	-0.91494	-0.71474	-0.54341
O	0.19807	0.44483	-0.92560
O	-0.58242	-1.33922	0.80158
O	-2.24923	0.19227	-0.27631
C	-2.66594	1.08873	-1.33090
H	-2.29383	0.73108	-2.29506
H	-2.25797	2.08398	-1.13596
C	-4.17575	1.16871	-1.38800
H	-4.43538	1.88770	-2.17368
O	-4.72964	-0.11134	-1.77058
C	-5.58011	-0.60375	-0.75252
H	-6.37529	-1.17401	-1.23248
C	-4.88320	1.58342	-0.07650
H	-4.23242	1.42504	0.78514
C	-6.10475	0.63323	-0.02965
H	-6.46340	0.43355	0.98194
O	-5.25204	2.94941	-0.04649
H	-5.92045	3.06414	-0.74685
O	-7.15690	1.15766	-0.83603
H	-7.67273	1.76168	-0.27685
N	-4.84261	-1.50552	0.13990
C	-4.10465	-2.58705	-0.30796
C	-4.50596	-1.34661	1.47315
C	-3.38477	-3.03156	0.80360
H	-4.93124	-0.56268	2.08102
N	-3.65962	-2.24858	1.91657
C	-2.50819	-4.11274	0.59357
N	-4.06651	-3.11627	-1.54142
C	-3.20137	-4.13260	-1.61072
N	-2.43243	-4.65371	-0.63998
H	-3.10434	-4.60695	-2.58368
N	-1.77230	-4.65707	1.59785
H	-0.99494	-5.24490	1.32018
H	-1.61752	-4.08188	2.41638
N	1.23325	-3.38236	2.35550
H	1.07693	-4.38621	2.27301
H	1.17442	-2.94744	3.27781

AA-TS-5

E: -3589.99670
G: -3589.43902

O	-1.30333	4.90278	-1.75010
C	-2.33350	4.40202	-0.89376
H	-3.04101	3.88192	-1.54533
H	-2.85482	5.22753	-0.39850
C	-1.80721	3.42424	0.15539
H	-1.18216	3.93169	0.89015
O	-2.96462	2.86652	0.83686
C	-2.86449	1.45622	0.82897
H	-2.32585	1.07899	1.70432
C	-1.02148	2.25041	-0.44984
H	-0.65661	2.47922	-1.44959
C	-2.07716	1.13972	-0.43973
H	-2.71779	1.20624	-1.32212
O	-1.57049	-0.18775	-0.30532
P	-0.05325	5.81441	-1.16811
O	-0.83737	6.93795	-0.25332
O	0.78973	5.02714	-0.19564
O	0.55615	6.43842	-2.39783
C	-0.97775	-0.73100	-1.36916
C	-0.62647	-2.18210	-1.11770
C	-0.39079	-2.67146	0.29921
H	0.65686	-2.47241	0.54651
H	-1.02112	-2.10790	0.98418
C	-0.64238	-4.16812	0.41004
O	-0.00902	-4.98427	-0.27730
O	-0.83403	-0.19056	-2.44820
H	-1.36054	7.53672	-0.81563
N	-4.18448	0.86896	0.85680
C	-4.50281	-0.34505	1.43609
C	-5.27383	1.22971	0.08595
C	-5.79905	-0.62898	0.99850
H	-5.27187	2.14576	-0.48640
N	-6.26856	0.37290	0.15751
C	-6.35186	-1.85034	1.43601
N	-3.74422	-1.10715	2.23899
C	-4.37873	-2.23384	2.58255
N	-5.60945	-2.64397	2.23982
H	-3.82797	-2.90769	3.23433
N	-7.60588	-2.24379	1.11162
H	-8.06663	-1.78633	0.33598

H	-7.84370	-3.21443	1.27335
N	-1.58598	-4.53179	1.29546
H	-2.17305	-3.84453	1.75025
H	-1.84071	-5.50979	1.36976
N	0.06166	-2.75954	-2.10315
H	0.47038	-3.67686	-1.95464
H	0.08248	-2.33927	-3.02464
C	-3.08080	-3.23076	-1.43007
H	-2.00722	-2.56402	-1.21583
O	-4.01076	-2.74572	-0.80305
O	-2.81204	-4.14086	-2.19922
O	1.38752	-0.19677	-0.21129
P	1.46371	1.31222	-0.24194
O	1.79428	2.03544	-1.52054
O	2.49688	1.87179	0.89512
C	2.36802	1.48550	2.27405
H	1.41213	0.98737	2.45626
H	2.39922	2.40343	2.86892
C	3.51007	0.58670	2.70670
H	3.46060	0.47626	3.79853
O	3.36576	-0.71441	2.10715
C	4.59757	-1.17671	1.56569
H	4.78665	-2.18364	1.93800
C	4.90902	1.09181	2.30967
H	4.84730	1.68537	1.39611
C	5.68723	-0.21308	2.04060
H	6.48699	-0.08992	1.30552
O	5.50674	1.92559	3.28392
H	5.61132	1.38281	4.08588
O	6.19931	-0.74488	3.26110
H	6.99642	-0.23540	3.48570
N	4.49538	-1.29528	0.11742
C	3.92408	-2.38273	-0.51797
C	4.63804	-0.32050	-0.85599
C	3.79966	-2.00984	-1.85756
H	5.05514	0.65021	-0.63306
N	4.26129	-0.71401	-2.05217
C	3.25320	-2.97470	-2.72807
N	3.57916	-3.56412	0.01906
C	3.08260	-4.39335	-0.90482
N	2.90969	-4.18040	-2.21975
H	2.78329	-5.37461	-0.55010
N	3.02241	-2.73189	-4.03878
H	2.81885	-3.52270	-4.63635
H	3.45895	-1.92716	-4.46819
O	0.06320	1.92099	0.41256

AA-Asn

E: -3401.46283
G: -3400.90766

O	0.96042	3.89275	-1.27462
C	-0.37619	4.15866	-0.83346
H	-1.00632	4.12567	-1.72650
H	-0.45560	5.15349	-0.38376
C	-0.83259	3.07992	0.14482
H	-0.34192	3.20791	1.11118
O	-2.26735	3.19785	0.32182
C	-2.78080	1.88668	0.47606
H	-2.64527	1.50905	1.49482
C	-0.55160	1.65185	-0.35924
H	-0.00922	1.64680	-1.30227
C	-1.96151	1.06532	-0.51455
H	-2.33658	1.21589	-1.52657
O	-2.02390	-0.31534	-0.16537
P	2.26327	4.28194	-0.33709
O	2.43504	5.89447	-0.63577
O	1.92151	4.19045	1.12905
O	3.40862	3.50610	-0.93738
C	-2.10242	-1.21963	-1.15883
C	-1.94295	-2.63996	-0.62615
C	-2.82489	-2.93215	0.59318
H	-2.60247	-3.94539	0.94009
H	-2.53851	-2.25683	1.40546
C	-4.34225	-2.83433	0.48601
O	-5.03813	-3.26878	1.41548
O	-2.26747	-0.93508	-2.33150
H	2.73733	6.04589	-1.54914
N	-4.19697	1.87184	0.19524
C	-5.11995	1.04176	0.80610
C	-4.81550	2.37040	-0.93798
C	-6.26546	1.12145	0.01016
H	-4.29588	3.04269	-1.60522
N	-6.05773	1.96516	-1.07564
C	-7.36659	0.34245	0.42084
N	-4.96954	0.31082	1.92084
C	-6.07834	-0.38273	2.20167
N	-7.24459	-0.40949	1.53779
H	-6.03424	-1.00642	3.08941
N	-8.54948	0.34521	-0.23729
H	-8.57552	0.71391	-1.17868
H	-9.20875	-0.39015	-0.01559
N	-4.89783	-2.26220	-0.60423
H	-4.36766	-1.85665	-1.36238
H	-5.89782	-2.09831	-0.59905
O	0.92510	-1.36750	1.31226
P	1.06902	-0.37407	0.18633
O	0.18248	0.95173	0.64100
O	0.76354	-0.73531	-1.24400
O	2.58468	0.25926	0.17089
C	3.10656	0.84308	1.37773
H	2.59395	0.43035	2.25165
H	2.94548	1.92334	1.35026
C	4.58601	0.54380	1.49180
H	4.98093	1.10554	2.34680
O	4.78337	-0.86295	1.76801
C	5.58056	-1.46317	0.76377
H	6.19612	-2.22634	1.24006
C	5.42862	0.86244	0.23729
H	4.79977	0.87917	-0.65372
C	6.41493	-0.32735	0.17669
H	6.79032	-0.52896	-0.82924
O	6.06485	2.12543	0.28749
H	6.62857	2.12649	1.08144
O	7.49743	-0.11985	1.07966
H	8.11197	0.49941	0.65126
N	4.72810	-2.11926	-0.23683
C	3.72111	-3.01326	0.08890
C	4.52118	-1.79151	-1.56665
C	2.97422	-3.17746	-1.08029
H	5.16824	-1.10505	-2.09111
N	3.50472	-2.41701	-2.11476
C	1.80676	-3.95991	-0.97701
N	3.48240	-3.60338	1.27114
C	2.38283	-4.36202	1.22538
N	1.54216	-4.56857	0.19860
H	2.12636	-4.87964	2.14621
N	0.95363	-4.14376	-2.02130
H	-0.00357	-4.39848	-1.77661
H	1.01004	-3.44344	-2.75211
H	-0.91283	-2.65701	-0.25328
N	-2.07191	-3.65832	-1.66945
H	-3.05864	-3.87198	-1.81565
H	-1.75539	-3.24814	-2.54858

CG-1

E: -4048.96114
G: -4048.43851

O	2.73009	-1.01467	-2.04627

C	1.96224	-2.11906	-2.56046
H	2.67030	-2.81702	-3.01328
H	1.25779	-1.78909	-3.32984
C	1.19174	-2.83399	-1.46356
H	0.67465	-3.67334	-1.93393
O	2.07713	-3.40299	-0.46482
C	2.03761	-2.65307	0.72135
H	1.61976	-3.26534	1.52672
C	0.20205	-1.97053	-0.66556
H	-0.24682	-1.17354	-1.25827
C	1.11156	-1.45455	0.43939
H	1.69007	-0.60245	0.08845
O	0.47500	-1.10405	1.67475
P	2.51989	0.48864	-2.61454
O	2.45369	0.53726	-4.10902
O	1.21382	1.03868	-1.88507
O	3.73582	1.23067	-1.96127
C	3.32260	-1.64767	2.46955
C	4.32506	-0.87583	2.95428
C	5.39099	-0.58530	2.04905
N	3.36938	-2.19718	1.21515
C	4.54444	-2.04588	0.42551
N	5.50329	-1.18664	0.87015
C	0.08280	0.17261	1.82300
C	0.02472	0.56195	3.30753
C	-0.57706	1.90854	3.63258
H	0.11011	2.67336	3.24618
H	-0.58140	1.98876	4.72355
C	-1.97755	2.10368	3.05555
H	-2.50133	2.92501	3.55317
H	-1.92583	2.36057	1.99420
C	-2.79970	0.81884	3.15641
O	-4.05452	0.89459	3.09365
O	-0.10287	0.94443	0.90314
O	0.68415	-0.08862	4.11407
O	-2.12912	-0.26456	3.25917
O	4.69897	-2.70270	-0.61453
N	6.35503	0.30176	2.40311
H	6.89265	0.70029	1.64215
H	6.14798	0.93352	3.16674
H	4.27238	-0.43824	3.94282
H	2.42582	-1.84763	3.04190
P	3.76035	2.11604	-0.39114
O	5.27114	2.23606	-0.20808
O	3.01547	3.40292	-0.73079
O	3.04402	1.16005	0.56124
H	0.38217	1.08670	-2.44274
O	-0.80559	-2.77799	-0.05406
P	-1.97408	-3.45633	-1.00763
O	-1.55152	-4.83844	-1.44670
O	-2.40702	-2.46016	-2.05902
O	-3.10902	-3.69448	0.14472
C	-3.42652	-2.76593	1.19432
H	-2.52600	-2.51258	1.75926
H	-4.11163	-3.30884	1.84980
C	-4.11264	-1.48847	0.75608
H	-4.58448	-1.04888	1.64474
O	-3.14170	-0.55625	0.24982
C	-3.88813	0.47163	-0.38242
H	-4.28301	1.17305	0.35886
C	-5.16435	-1.60156	-0.36408
H	-4.89522	-2.40531	-1.04882
C	-5.06447	-0.22278	-1.08987
H	-4.86739	-0.35040	-2.15680
O	-6.46927	-1.86600	0.11841
H	-6.85820	-0.98752	0.29686
O	-6.22542	0.57288	-0.88369
H	-6.88922	0.29953	-1.53909
N	-2.98947	1.21443	-1.23411
C	-2.47686	2.46953	-0.96186
C	-2.20138	0.66985	-2.23008
C	-1.42372	2.63916	-1.85288
H	-2.34368	-0.34688	-2.56873
N	-1.27103	1.50299	-2.64342
C	-0.61021	3.80599	-1.74996
N	-2.92240	3.32667	-0.02221
C	-2.17376	4.41586	0.07788
N	-1.08783	4.65637	-0.72947
H	-0.54269	5.50026	-0.57519
N	-2.43566	5.32921	1.04224
H	-2.06642	6.26696	0.94275
H	-3.33679	5.24886	1.49599
O	0.39403	4.11757	-2.40170

CG-TS-1

E: -4048.92509
G: -4048.40478

O	-4.22037	-1.94482	0.67024
C	-3.79784	-3.25811	0.27534
H	-4.67913	-3.77920	-0.10969
H	-3.40966	-3.82276	1.12821
C	-2.73101	-3.23661	-0.80945
H	-2.44786	-4.27229	-1.01585
O	-3.24254	-2.68095	-2.05225
C	-2.69197	-1.41019	-2.29020
H	-2.01486	-1.45742	-3.14971
C	-1.48883	-2.40910	-0.47488
H	-1.28508	-2.39300	0.59494
C	-1.89971	-1.04795	-1.02225
H	-2.52437	-0.50654	-0.31689
O	-0.79864	-0.21453	-1.41775
P	-3.96995	-1.39950	2.20329
O	-4.56504	-2.63029	3.12120
O	-2.49780	-1.33353	2.51765
O	-4.86618	-0.18447	2.33030
C	-3.09949	0.78953	-3.11263
C	-3.70761	1.99648	-3.03383
C	-4.95673	2.02547	-2.34045
N	-3.68536	-0.35367	-2.62967
C	-5.03008	-0.30223	-2.15557
N	-5.61737	0.91932	-2.02615
C	-0.30096	0.62362	-0.50388
C	0.76794	1.53223	-1.12591
C	0.66094	2.98606	-0.77820
H	0.93369	3.09597	0.28076
H	1.39959	3.52262	-1.37819
C	-0.77171	3.53786	-0.95000
H	-0.72876	4.46901	-1.52720
H	-1.40478	2.86112	-1.53375
C	-1.50679	3.85407	0.36882
O	-0.83870	3.95745	1.42751
O	-0.62121	0.69098	0.66291
O	1.64144	1.02949	-1.81550
O	-2.76867	4.01813	0.26576
O	-5.64547	-1.34718	-1.89711
N	-5.51671	3.22435	-2.02736
H	-6.21454	3.19216	-1.29177
H	-4.87131	4.00269	-1.94944
H	-3.23271	2.89606	-3.40308
H	-2.11063	0.66924	-3.53867
P	-3.88960	2.03033	1.43334
O	-5.23387	2.71705	1.47036
O	-2.98305	2.09650	2.63224
O	-3.48399	1.26747	0.20400
H	-5.52887	-2.71169	3.00430
O	1.08083	-4.24356	0.42703
P	1.10585	-3.06572	-0.51705
O	-0.37465	-2.88982	-1.23103
O	2.08593	-3.00272	-1.65982

O	1.23601	-1.66867	0.34115
C	1.20983	-1.66249	1.77554
H	1.24164	-2.68792	2.14760
H	0.28088	-1.19270	2.11129
C	2.37603	-0.87830	2.33313
H	2.30284	-0.92365	3.42909
O	3.62316	-1.48171	1.91912
C	4.61625	-0.48528	1.74120
H	5.49496	-0.71568	2.34720
C	2.50401	0.59044	1.91344
H	2.30185	0.69606	0.84767
C	3.99784	0.85321	2.16182
H	4.38966	1.68924	1.57452
O	1.62360	1.47055	2.58198
H	1.68929	1.29541	3.53696
O	4.27364	1.02376	3.54777
H	3.85835	1.85881	3.82336
N	5.04462	-0.47493	0.34055
C	6.23513	0.04120	-0.11305
C	4.31338	-0.83315	-0.78139
C	6.16928	-0.05141	-1.50363
H	3.33878	-1.28543	-0.69924
N	4.96049	-0.60482	-1.90335
C	7.27966	0.40774	-2.27074
N	7.24020	0.52291	0.65413
C	8.26806	0.95240	-0.05838
N	8.30217	0.90312	-1.43128
N	9.33427	1.52630	0.56058
H	9.40362	1.33949	1.55362
H	10.21881	1.53670	0.06557
O	7.43220	0.41987	-3.50113
H	9.12094	1.26398	-1.91359

CG-2

E: -4048.95040
G: -4048.42943

O	0.23573	3.34162	1.41050
C	1.47084	3.37484	0.68182
H	2.23280	2.93799	1.32904
H	1.76658	4.40017	0.43903
C	1.32281	2.55359	-0.59728
H	0.55861	3.00127	-1.23449
O	2.58225	2.55407	-1.31953
C	3.07376	1.23132	-1.42860
H	2.81962	0.82075	-2.41068
C	0.97222	1.09012	-0.31682
H	0.48038	0.96431	0.64620
C	2.34595	0.42308	-0.35603
H	2.84838	0.47854	0.60743
O	2.30257	-0.93778	-0.80516
P	-0.91677	4.48477	1.12570
O	-0.27886	5.80839	1.87480
O	-0.96315	4.85363	-0.33711
O	-2.13615	3.99074	1.86290
C	5.18747	0.39783	-2.25903
C	6.48348	0.04345	-2.09190
C	7.11090	0.46399	-0.87741
N	4.52910	1.15658	-1.32729
C	5.20002	1.58367	-0.14818
N	6.50601	1.25123	0.00832
C	2.09930	-1.88402	0.11379
C	2.05279	-3.25984	-0.55171
C	3.32914	-4.04214	-0.68872
H	3.68105	-4.26664	0.32743
H	3.07463	-4.98461	-1.17866
C	4.44732	-3.30127	-1.47963
H	4.95640	-4.01079	-2.13351
H	4.02378	-2.51162	-2.10975
C	5.47252	-2.69119	-0.54502
O	6.66175	-2.99201	-0.58416
O	1.89947	-1.70707	1.29666
O	0.95629	-3.66445	-0.90590
O	4.92792	-1.84139	0.31312
O	4.57714	2.24819	0.69450
N	8.38668	0.08854	-0.62009
H	8.67609	0.14816	0.34940
H	8.74291	-0.71205	-1.12800
H	7.00083	-0.56803	-2.81940
H	4.60819	0.09925	-3.12446
P	5.45209	-1.71105	2.02660
O	4.58911	-0.52039	2.43774
O	6.95864	-1.46973	1.98669
O	5.01476	-3.08077	2.54766
H	-0.23574	5.67115	2.83812
O	-1.79829	-0.72012	-2.25610
P	-1.25720	-0.16580	-0.96476
O	0.17233	0.55893	-1.37591
O	-1.07634	-1.02169	0.26171
O	-2.12360	1.13845	-0.45839
C	-2.65843	2.05067	-1.43235
H	-2.38360	1.73776	-2.44262
H	-2.24335	3.04320	-1.23997
C	-4.16551	2.10612	-1.30057
H	-4.55482	2.81843	-2.04119
O	-4.71646	0.80082	-1.56347
C	-5.78607	0.50737	-0.67603
H	-6.68908	0.27210	-1.24154
C	-4.67659	2.50627	0.09305
H	-3.98874	2.15738	0.86471
C	-6.01773	1.75337	0.19098
H	-6.28938	1.49212	1.21685
O	-4.79461	3.90588	0.26423
H	-5.46197	4.20597	-0.37901
O	-7.05703	2.50694	-0.43325
H	-7.33410	3.19078	0.19952
N	-5.47218	-0.69281	0.08902
C	-6.23970	-1.83494	0.13998
C	-4.36515	-0.92799	0.89178
C	-5.54820	-2.70534	0.98204
H	-3.59041	-0.18707	1.00810
N	-4.37589	-2.11983	1.44217
C	-6.09999	-3.99536	1.23499
N	-7.41393	-2.03810	-0.49794
C	-7.92355	-3.23675	-0.26869
N	-7.31958	-4.16629	0.54345
N	-9.06995	-3.61944	-0.89045
H	-9.60863	-4.37029	-0.47414
H	-9.61923	-2.86394	-1.28163
H	-7.76682	-5.07005	0.66971
O	-5.65769	-4.91141	1.94311

CG-TS-2

E: -4238.69612
G: -4238.14625

O	0.54391	-3.16840	1.69597
C	-0.64327	-3.55534	0.98974
H	-1.48697	-3.12651	1.53247
H	-0.76030	-4.64342	0.96904
C	-0.58667	-3.00723	-0.43410
H	0.25811	-3.45304	-0.96129
O	-1.80727	-3.35287	-1.13382
C	-2.49621	-2.17396	-1.52954
H	-2.26131	-1.93033	-2.57029
C	-0.47805	-1.48544	-0.48297
H	-0.02074	-1.07556	0.41471
C	-1.93949	-1.06235	-0.62629
H	-2.44161	-1.04638	0.33727
P	1.88488	-4.12396	1.63727

O	1.44873	-5.38380	2.60832
O	2.05896	-4.73410	0.26754
O	2.97088	-3.30138	2.28484
C	-4.65922	-2.19171	-2.61974
C	-6.01275	-2.19963	-2.60909
C	-6.64435	-2.39556	-1.33995
N	-3.93568	-2.38577	-1.47474
C	-4.59779	-2.56228	-0.23132
N	-5.95408	-2.57307	-0.21211
O	-3.91946	-2.70741	0.79980
N	-7.98780	-2.42306	-1.26659
H	-8.43073	-2.45028	-0.35762
H	-8.54788	-2.16639	-2.06777
H	-6.58723	-2.05485	-3.51490
H	-4.08317	-2.03450	-3.52375
H	1.32343	-5.08356	3.52640
O	1.99170	0.24599	-2.85498
P	1.48935	-0.02463	-1.46128
O	0.24561	-1.10064	-1.65715
O	1.06371	1.08133	-0.53501
O	2.55949	-0.92961	-0.59767
C	3.35656	-1.90543	-1.28939
H	3.13478	-1.88560	-2.35894
H	3.12006	-2.89685	-0.89412
C	4.82631	-1.60925	-1.06838
H	5.41348	-2.30255	-1.68446
O	5.12645	-0.26138	-1.49613
C	5.78420	0.45602	-0.46212
H	6.54669	1.08203	-0.92617
C	5.30976	-1.70999	0.38988
H	4.49520	-1.48836	1.08159
C	6.38583	-0.60692	0.45401
H	6.59185	-0.25222	1.46640
O	5.77586	-2.99932	0.73968
H	6.48522	-3.22470	0.11212
O	7.58827	-1.04288	-0.17466
H	8.04306	-1.62947	0.45253
N	4.84782	1.33657	0.24690
C	4.10553	2.32767	-0.36077
C	4.39572	1.27366	1.55987
C	3.27272	2.83393	0.63463
H	4.80326	0.57631	2.27469
N	3.46734	2.15971	1.83032
C	2.29026	3.80295	0.27859
N	4.16507	2.68559	-1.66316
C	3.25024	3.58037	-1.99332
N	2.37417	4.13077	-1.08820
N	3.11268	3.97044	-3.28844
H	2.67768	4.86698	-3.47160
H	3.89211	3.73825	-3.89162
H	1.66514	4.77706	-1.42370
O	1.42973	4.33920	0.99310
O	-4.23602	0.46803	-0.96620
C	-3.13431	0.92417	-1.21833
C	-2.93128	2.39743	-1.61668
C	-1.63111	3.07851	-1.27631
H	-0.88499	2.36222	-0.93224
H	-1.26074	3.54036	-2.19948
C	-1.84197	4.17697	-0.22445
H	-0.92021	4.75478	-0.12139
H	-2.63519	4.86143	-0.54941
C	-2.21922	3.68610	1.18260
O	-2.35017	4.56042	2.09410
O	-1.98741	0.25490	-1.20160
O	-3.86224	2.95152	-2.17640
O	-3.32302	2.71327	0.99156
P	-4.07297	2.02790	2.29516
O	-4.10188	0.44560	1.83454
O	-5.48375	2.57454	2.34795
O	-3.20113	2.06704	3.52689
H	-4.40948	0.33446	0.91257
C	-0.56632	1.89837	2.24972
H	-1.31153	2.87364	1.45578
O	-0.82873	0.80747	1.80896
O	0.03222	2.55409	3.06545

CG-3

E: -3406.88652
G: -3406.36967

O	-0.63401	-3.24763	1.43230
C	-1.86443	-3.17495	0.69997
H	-2.62894	-2.82643	1.39608
H	-2.16398	-4.15595	0.31849
C	-1.70702	-2.19156	-0.45723
H	-0.95407	-2.56304	-1.15359
O	-2.96879	-2.07513	-1.16187
C	-3.43898	-0.73736	-1.09611
H	-3.15639	-0.19339	-2.00260
C	-1.33584	-0.78169	0.00485
H	-0.80711	-0.78333	0.95694
C	-2.70417	-0.10894	0.09579
H	-3.20380	-0.33761	1.03422
P	0.50274	-4.36847	1.02070
O	-0.16417	-5.76178	1.59809
O	0.55774	-4.55698	-0.47549
O	1.72018	-3.98712	1.82556
C	-5.56249	0.03209	-1.97944
C	-6.89593	0.24598	-1.89141
C	-7.56396	-0.31080	-0.75422
N	-4.89292	-0.69510	-1.03157
C	-5.58605	-1.21128	0.09596
N	-6.92394	-1.00981	0.18559
O	-4.94925	-1.83491	0.96187
N	-8.89027	-0.14127	-0.61957
H	-9.35571	-0.47691	0.21302
H	-9.40378	0.44320	-1.26436
H	-7.42860	0.81295	-2.64390
H	-4.96321	0.41600	-2.79641
H	-0.21068	-5.74435	2.57073
O	1.21621	1.43677	-1.79840
P	0.80807	0.67406	-0.56493
O	-0.56822	-0.13200	-1.01075
O	0.60793	1.35949	0.76248
O	1.82608	-0.58305	-0.27062
C	2.05356	-1.52881	-1.33322
H	1.75207	-1.10092	-2.29330
H	1.46409	-2.42909	-1.14038
C	3.51834	-1.90557	-1.40074
H	3.63542	-2.63033	-2.21579
O	4.31805	-0.74736	-1.72779
C	5.27099	-0.50018	-0.70935
H	6.16672	-0.10077	-1.18483
C	4.11780	-2.50196	-0.10868
H	3.52636	-2.21777	0.76270
C	5.51988	-1.85328	-0.04891
H	5.92453	-1.78379	0.96355
O	4.15107	-3.91771	-0.10908
H	4.67015	-4.18640	-0.88790
O	6.42589	-2.54883	-0.90086
H	6.69106	-3.35992	-0.43553
N	4.76783	0.50599	0.23237
C	4.32494	1.75189	-0.15987
C	4.39625	0.37123	1.56439
C	3.73756	2.31185	0.97287
H	4.61071	-0.52055	2.13211
N	3.79995	1.43493	2.04639
C	3.12983	3.59618	0.86658
N	4.45012	2.29283	-1.39198
C	3.86608	3.47390	-1.49668

N	3.24282	4.10124	-0.44409
N	3.82326	4.10845	-2.69674
H	3.68463	5.11211	-2.70327
H	4.46387	3.75428	-3.39609
H	2.80347	5.00401	-0.60181
O	2.55532	4.26301	1.74033
O	-4.61256	1.75506	0.79164
C	-3.52081	2.10682	0.39516
C	-3.08457	3.59125	0.37011
C	-1.64804	3.95224	0.08175
H	-1.12814	3.14236	-0.42764
H	-1.66864	4.82790	-0.57429
C	-0.89221	4.34297	1.36949
H	0.10013	4.72928	1.11941
H	-1.43422	5.15494	1.87523
C	-0.72588	3.24155	2.38943
O	0.09087	3.30455	3.29358
O	-2.54432	1.30896	-0.03704
O	-3.94042	4.41457	0.63955
H	-1.41582	2.38025	2.32893

CG-TS-3

E: -3406.86261
G: -3406.34308

O	-2.60604	-3.11606	1.42131
C	-3.54605	-2.70468	0.42512
H	-4.30007	-2.12094	0.95949
H	-4.03366	-3.56903	-0.03675
C	-2.88121	-1.85509	-0.66534
H	-2.27821	-2.49434	-1.30749
O	-3.86623	-1.22009	-1.51820
C	-4.10113	0.11442	-1.12287
H	-3.98182	0.77512	-1.98364
C	-2.03986	-0.70929	-0.13149
H	-1.56552	-0.92873	0.82351
C	-3.06346	0.43733	-0.02759
H	-3.52522	0.45780	0.95776
P	-1.28730	-4.04988	1.05017
O	-1.53542	-5.33551	2.04639
O	-1.41063	-4.57044	-0.36045
O	-0.07252	-3.28155	1.51205
H	-1.20631	-5.14100	2.94188
O	0.89413	0.83161	-1.99352
P	0.40526	0.18199	-0.70817
O	-1.07218	-0.39034	-1.13317
O	0.36951	0.96771	0.57662
O	1.24309	-1.17451	-0.37731
C	1.35949	-2.19404	-1.38504
H	1.25974	-1.75749	-2.38284
H	0.57009	-2.93637	-1.23738
C	2.70295	-2.87236	-1.26338
H	2.72432	-3.70383	-1.98154
O	3.75056	-1.93728	-1.59761
C	4.90707	-2.18341	-0.83103
H	5.74735	-2.40220	-1.49574
C	3.07406	-3.40199	0.12690
H	2.72353	-2.71532	0.89891
C	4.61373	-3.39216	0.07252
H	5.06765	-3.30146	1.06214
O	2.51818	-4.66816	0.42438
H	2.82662	-5.28353	-0.26447
O	5.10179	-4.54465	-0.60808
H	5.02848	-5.29440	0.00649
N	5.27494	-0.98401	-0.04325
C	4.84320	0.32719	-0.11762
C	6.33496	-0.97906	0.84254
C	5.66594	1.03954	0.76027
H	6.88205	-1.88453	1.06275
N	6.59576	0.20532	1.35118
C	5.46721	2.45274	0.94041
N	3.82967	0.85270	-0.85672
C	3.69971	2.13280	-0.64415
N	4.42696	2.94133	0.14490
N	2.60136	2.82140	-1.33085
H	2.95847	3.30810	-2.16367
H	1.89833	2.08161	-1.63634
H	3.89378	4.03470	0.17101
O	6.10852	3.20816	1.69141
O	-2.26041	2.11253	1.93955
C	-1.96765	2.35348	0.78736
C	-1.01236	3.51183	0.42575
C	-0.54672	3.69627	-0.99297
H	-0.44545	2.72852	-1.48499
H	-1.36699	4.21216	-1.51432
C	0.73579	4.52707	-1.09874
H	0.96590	4.72067	-2.15190
H	0.58527	5.49781	-0.61973
C	1.95965	3.92087	-0.40778
O	2.93728	4.84815	-0.15053
O	-2.40960	1.68755	-0.28384
O	-0.68917	4.23239	1.35562
H	1.67027	3.33354	0.47432
O	1.48359	6.16232	1.83025
H	0.69853	5.59497	1.72577
H	2.10931	5.76864	1.17781
C	-6.34533	-0.81681	-0.57090
C	-7.61547	-0.66723	-0.11460
C	-8.01308	0.64820	0.27709
N	-5.50087	0.24976	-0.64245
C	-5.93631	1.54230	-0.28143
N	-7.19863	1.70324	0.18812
O	-5.13330	2.48579	-0.40618
N	-9.26642	0.85763	0.72856
H	-9.49693	1.75890	1.12559
H	-9.85753	0.07088	0.95940
H	-8.29470	-1.50808	-0.05623
H	-5.94619	-1.76400	-0.90357

CG-4

E: -3330.43504
G: -3329.93949

O	1.57787	3.24635	1.69292
C	2.76135	3.10028	0.90217
H	3.51323	2.67491	1.57199
H	3.12008	4.07031	0.54318
C	2.53809	2.15973	-0.28580
H	1.95771	2.66375	-1.05733
O	3.82882	1.80225	-0.84706
C	4.02017	0.39613	-0.78637
H	3.79793	-0.07865	-1.74214
C	1.84961	0.84801	0.08815
H	1.22288	0.93596	0.97407
C	3.04870	-0.08114	0.29162
H	3.47548	0.04833	1.28712
P	0.21275	3.97770	1.10991
O	0.21992	5.40489	1.93341
O	0.39347	4.34744	-0.34147
O	-0.93472	3.11930	1.58291
H	-0.07856	5.27391	2.85090
O	-0.50727	-1.13203	-2.15293
P	-0.29337	-0.46515	-0.81977
O	1.09659	0.40271	-1.03884
O	-0.24315	-1.23261	0.48000
O	-1.40097	0.71840	-0.56716
C	-1.67002	1.62892	-1.64443
H	-1.41581	1.16537	-2.60150
H	-1.06795	2.53352	-1.51463
C	-3.12939	2.01333	-1.64384

H	-3.29088	2.70456	-2.48331
O	-3.95136	0.84080	-1.83455
C	-5.17685	0.97417	-1.14782
H	-6.00249	0.89121	-1.85972
C	-3.66862	2.65864	-0.36573
H	-3.25881	2.15861	0.51373
C	-5.17386	2.36204	-0.48276
H	-5.67694	2.38082	0.48606
O	-3.35073	4.03051	-0.23383
H	-3.65169	4.47970	-1.04320
O	-5.80425	3.25291	-1.39835
H	-5.88374	4.11458	-0.95540
N	-5.34511	-0.10603	-0.14620
C	-4.72385	-1.33548	-0.00841
C	-6.36136	-0.08805	0.79359
C	-5.39554	-1.96138	1.04801
H	-7.03894	0.74882	0.87300
N	-6.42042	-1.17220	1.53153
C	-4.98780	-3.26148	1.47631
N	-3.70227	-1.85484	-0.73704
C	-3.30517	-3.03376	-0.32012
N	-3.89734	-3.71631	0.71133
N	-2.25706	-3.67728	-0.98372
H	-3.53516	-4.63440	0.95479
O	-5.46102	-3.96620	2.37833
O	2.24078	-1.72416	2.27634
C	2.19925	-2.10799	1.12650
C	1.54457	-3.45136	0.74552
C	1.25449	-3.77526	-0.69756
H	1.18352	-2.86686	-1.29274
H	2.12040	-4.33861	-1.07291
C	-0.01623	-4.63669	-0.86085
H	-0.17500	-4.85552	-1.91915
H	0.12617	-5.58361	-0.32543
C	-1.22104	-3.96544	-0.28640
O	2.71141	-1.45633	0.07328
O	1.26870	-4.19769	1.66845
H	-1.17391	-3.72059	0.77717
C	6.07759	0.83281	0.46310
C	7.35774	0.54181	0.79592
C	7.97079	-0.55332	0.10533
N	5.42029	0.10871	-0.49095
C	6.03694	-1.00114	-1.12032
N	7.32798	-1.28313	-0.80966
O	5.37666	-1.67627	-1.92969
N	9.25261	-0.86339	0.37019
H	9.66497	-1.68263	-0.05595
H	9.74903	-0.40375	1.12078
H	7.89382	1.11493	1.54149
H	5.51962	1.64603	0.90955

CG-TS-4

E: -3463.42882
G: -3462.87437

C	3.01082	1.97982	-0.01931
H	2.52213	2.81257	-0.52610
O	3.81650	1.26803	-0.99934
C	3.52434	-0.11902	-0.96516
H	2.89836	-0.41720	-1.80616
C	1.96263	0.97346	0.46278
H	1.60763	1.16842	1.47584
C	2.78755	-0.31439	0.35624
H	3.49851	-0.34515	1.18432
O	-0.02388	-0.96371	-1.72238
P	-0.34552	-0.05104	-0.55592
O	0.88387	1.05057	-0.47156
O	-0.66664	-0.59049	0.81494
O	-1.56343	0.95488	-0.97758
C	-1.79990	1.22601	-2.36888
H	-1.74919	0.29551	-2.93885
H	-1.04140	1.92085	-2.74689
C	-3.16240	1.85230	-2.54711
H	-3.25222	2.14331	-3.60203
O	-4.19805	0.88667	-2.25569
C	-5.24451	1.46950	-1.51257
H	-6.19677	1.19708	-1.97370
C	-3.47984	3.06558	-1.66481
H	-3.03607	2.94290	-0.67476
C	-5.01346	2.98637	-1.56044
H	-5.40172	3.52243	-0.69347
O	-2.99118	4.28892	-2.17891
H	-3.41399	4.42041	-3.04655
O	-5.62311	3.45695	-2.75936
H	-5.66662	4.42580	-2.70056
N	-5.24902	0.96357	-0.11948
C	-4.73855	-0.21832	0.39593
C	-5.93681	1.56644	0.92078
C	-5.13153	-0.22181	1.73891
H	-6.47124	2.49391	0.78219
N	-5.88512	0.89522	2.04796
C	-4.75328	-1.31649	2.57155
N	-4.02512	-1.17034	-0.24553
C	-3.61853	-2.16249	0.52815
N	-3.98708	-2.25111	1.85511
N	-2.81323	-3.11922	-0.04178
H	-3.69519	-3.05568	2.39984
O	-5.01830	-1.51054	3.76844
O	1.91085	-1.51958	2.58757
C	1.63379	-2.00339	1.51064
C	0.74863	-3.26082	1.38170
C	0.29895	-3.72165	0.02408
H	0.10893	-2.85727	-0.61494
H	1.15250	-4.24773	-0.42983
C	-0.93060	-4.64951	0.08514
H	-1.19127	-4.94797	-0.93335
H	-0.69569	-5.54099	0.67000
C	-2.08420	-3.91005	0.71078
O	2.05488	-1.54056	0.32707
O	0.45196	-3.81125	2.42807
H	-1.97524	-3.72631	1.77880
N	-3.27762	-5.66585	1.20466
H	-4.16704	-5.38117	1.61278
H	-2.83222	-6.32935	1.83717
H	-3.47163	-6.14259	0.32539
O	-1.93260	-2.68651	-2.70021
H	-1.21924	-2.06511	-2.43059
H	-2.36288	-2.86620	-1.83055
C	5.89707	-0.46580	-0.43930
C	7.02573	-1.21547	-0.46371
C	6.95593	-2.46410	-1.16161
N	4.76007	-0.89492	-1.06231
C	4.70351	-2.16522	-1.68720
N	5.83783	-2.90805	-1.74144
O	3.61999	-2.55275	-2.16051
N	8.05905	-3.22841	-1.25747
H	7.99093	-4.14881	-1.67077
H	8.89727	-2.98641	-0.74767
H	7.93845	-0.88121	0.01273
H	5.83646	0.49923	0.04656
O	3.15325	3.32456	1.98786
C	3.91823	2.52282	1.08074
H	4.35991	1.71217	1.66644
H	4.73123	3.09617	0.62509
P	3.05695	4.95237	1.71518
O	4.60378	5.43578	2.01655
O	2.83312	5.23538	0.25074
O	2.11666	5.46196	2.77704
H	4.82517	5.31336	2.95681

CG-5
E: -3387.48548
G: -3386.94023

O	-0.06156	-3.71288	1.24587
C	-1.35685	-3.78872	0.63994
H	-2.07668	-3.54453	1.42621
H	-1.56818	-4.79885	0.27444
C	-1.47491	-2.77544	-0.49993
H	-0.86668	-3.08884	-1.34795
O	-2.86309	-2.70823	-0.92400
C	-3.33875	-1.37595	-0.81319
H	-3.26522	-0.84306	-1.76178
C	-1.07491	-1.36305	-0.07625
H	-0.40522	-1.36608	0.78195
C	-2.43422	-0.73249	0.23434
H	-2.76394	-0.98381	1.24364
P	1.25115	-4.42722	0.54285
O	1.08394	-5.99701	1.01667
O	1.11216	-4.45283	-0.95909
O	2.43801	-3.79122	1.22199
H	1.23253	-6.08488	1.97534
O	0.92053	1.12542	-2.18610
P	0.71795	0.40446	-0.87941
O	-0.48267	-0.68929	-1.18939
O	0.42345	1.15186	0.40082
O	1.97149	-0.58439	-0.50907
C	2.58980	-1.33517	-1.57071
H	2.23451	-0.97544	-2.53961
H	2.32367	-2.38999	-1.46292
C	4.09218	-1.16975	-1.49373
H	4.53462	-1.67198	-2.36340
O	4.43109	0.23412	-1.56487
C	5.27049	0.59853	-0.48212
H	5.99226	1.32718	-0.85247
C	4.76942	-1.70419	-0.21778
H	4.08658	-1.65745	0.63272
C	5.92938	-0.70424	-0.03154
H	6.32841	-0.67649	0.98392
O	5.17559	-3.05567	-0.31901
H	5.79136	-3.11307	-1.07156
O	6.97234	-0.97077	-0.96556
H	7.50467	-1.69775	-0.60194
N	4.49461	1.22572	0.60074
C	3.61539	2.27723	0.43604
C	4.37324	0.83453	1.92891
C	3.03510	2.47634	1.68760
H	4.95214	0.02494	2.34364
N	3.52130	1.56297	2.60986
C	1.99636	3.44395	1.81994
N	3.35026	2.93366	-0.71536
C	2.36753	3.80862	-0.59809
N	1.75679	4.10113	0.59662
N	-3.30456	5.49319	0.14407
H	0.95155	4.72167	0.59504
O	1.32819	3.73778	2.82184
O	-1.91012	1.06200	2.23461
C	-1.98714	1.42369	1.08058
C	-1.66231	2.85810	0.63618
C	-1.69554	3.22121	-0.82905
H	-1.30256	2.40660	-1.43571
H	-1.04739	4.09206	-0.96777
C	-3.11848	3.53267	-1.32159
H	-3.10645	3.66682	-2.41201
H	-3.80760	2.69032	-1.15857
C	-3.78064	4.73515	-0.77857
O	-2.41961	0.68373	0.05141
O	-1.43916	3.67862	1.51247
H	-4.74662	5.01724	-1.18946
C	-5.20882	-2.28080	0.48185
C	-6.49874	-2.25159	0.89415
C	-7.33635	-1.24018	0.32230
N	-4.75053	-1.38635	-0.44370
C	-5.58682	-0.35745	-0.94138
N	-6.88570	-0.33745	-0.55413
O	-5.10184	0.48890	-1.71625
N	-8.63321	-1.18499	0.66944
H	-9.22082	-0.44928	0.30127
H	-9.01214	-1.81443	1.36280
H	-6.87966	-2.96304	1.61560
H	-4.48740	-3.00575	0.83665
N	1.96344	4.52537	-1.68601
H	1.03338	4.92836	-1.64498
H	2.17502	4.07765	-2.57045
H	-2.41954	5.28353	0.61660
H	-3.82416	6.30970	0.46265

CG-TS-5
E: -3576.78825
G: -3576.22781

O	0.07776	-4.28408	1.11363
C	-1.17612	-4.30428	0.42239
H	-1.95232	-4.22051	1.18816
H	-1.32031	-5.24299	-0.12140
C	-1.25973	-3.11766	-0.53636
H	-0.56277	-3.25647	-1.36329
O	-2.60869	-3.03589	-1.07025
C	-3.13090	-1.73831	-0.83382
H	-2.93456	-1.06538	-1.66923
C	-0.97715	-1.77939	0.14877
H	-0.38991	-1.89648	1.05738
C	-2.38637	-1.26220	0.41794
H	-2.81434	-1.71269	1.31562
P	1.44942	-4.87950	0.41082
O	1.38724	-6.47099	0.83525
O	1.32835	-4.86727	-1.09287
O	2.58321	-4.18811	1.12599
H	1.53784	-6.57771	1.79165
O	1.06728	1.10228	-1.29022
P	0.86432	0.08345	-0.19829
O	-0.32745	-0.90877	-0.77796
O	0.56920	0.46593	1.22794
O	2.13332	-0.95620	-0.11314
C	2.61508	-1.56176	-1.32672
H	2.15558	-1.08949	-2.19924
H	2.35104	-2.62207	-1.31917
C	4.11856	-1.41068	-1.41468
H	4.46007	-1.92604	-2.32136
O	4.46286	-0.01275	-1.54147
C	5.37992	0.37560	-0.53113
H	6.08772	1.07559	-0.97496
C	4.91227	-1.94402	-0.20456
H	4.29295	-1.94202	0.69336
C	6.05752	-0.91469	-0.07441
H	6.47862	-0.85695	0.93195
O	5.35732	-3.27743	-0.36748
H	5.92646	-3.28837	-1.15772
O	7.08022	-1.18882	-1.02901
H	7.62950	-1.90308	-0.66505
N	4.68083	1.06684	0.55810
C	3.87749	2.17368	0.36225
C	4.41784	0.62989	1.85154
C	3.19197	2.35311	1.56270
H	4.91636	-0.22687	2.27724
N	3.55349	1.38228	2.48748
C	2.16347	3.34047	1.62179
N	3.78039	2.89891	-0.77393
C	2.84001	3.82804	-0.70693
N	2.08223	4.05741	0.41650

H	1.34384	4.76096	0.35703
O	1.37937	3.59715	2.54991
C	-5.14285	-2.84228	0.01781
C	-6.47294	-2.85783	0.27412
C	-7.23525	-1.74297	-0.20255
N	-4.57835	-1.81087	-0.67709
C	-5.34323	-0.67717	-1.04753
N	-6.68060	-0.70385	-0.83405
O	-4.76077	0.29895	-1.55701
N	-8.56634	-1.72775	-0.01910
H	-9.10240	-0.91953	-0.30504
H	-9.02558	-2.45976	0.50443
H	-6.93821	-3.67790	0.80571
H	-4.46943	-3.63488	0.31784
N	2.61064	4.65346	-1.76069
H	1.73014	5.15956	-1.77190
H	2.95209	4.32405	-2.65467
N	-3.65017	4.63132	-0.87417
O	-4.28778	0.22692	1.75964
C	-3.34017	0.76552	1.22706
C	-3.11038	2.29515	1.26620
C	-1.81881	2.84205	0.72033
H	-1.00133	2.17734	1.01150
H	-1.64827	3.82348	1.17418
C	-1.83400	2.94959	-0.82494
H	-0.80810	2.89486	-1.19169
H	-2.37308	2.10313	-1.26906
C	-2.46019	4.21495	-1.36095
O	-2.36919	0.16152	0.54421
O	-4.00970	2.97321	1.73151
H	-2.33963	4.37490	-2.43202
H	-3.84001	4.51735	0.11748
H	-4.07062	5.45157	-1.29587
C	-0.94481	6.08210	-0.33620
H	-1.49811	5.11981	-0.98919
O	-1.79150	6.65383	0.32431
O	0.25998	6.13071	-0.57855

CG-6

E: -3388.24666
G: -3387.69108

O	0.07780	3.80540	1.22536
C	1.35571	3.80691	0.57800
H	2.10150	3.66448	1.36519
H	1.55165	4.76113	0.07900
C	1.42998	2.65689	-0.42367
H	0.75555	2.84800	-1.25941
O	2.78860	2.55953	-0.92424
C	3.23471	1.21491	-0.79904
H	2.99744	0.62500	-1.68409
C	1.08827	1.30071	0.20060
H	0.52672	1.40318	1.12702
C	2.46916	0.68833	0.41970
H	2.91907	1.06239	1.34146
P	-1.23937	4.49780	0.50826
O	-1.08240	6.07713	0.95444
O	-1.09422	4.49625	-0.99335
O	-2.42685	3.87237	1.19629
H	-1.25112	6.18199	1.90804
O	-1.11886	-1.41625	-1.30091
P	-0.87912	-0.42258	-0.19459
O	0.36585	0.51730	-0.74987
O	-0.61837	-0.83231	1.23106
O	-2.10259	0.67294	-0.10960
C	-2.54989	1.31017	-1.31956
H	-2.10237	0.82969	-2.19395
H	-2.24328	2.35894	-1.29801
C	-4.05737	1.22303	-1.42446
H	-4.36770	1.77742	-2.31932
O	-4.46001	-0.15488	-1.59536
C	-5.40935	-0.52978	-0.61114
H	-6.13889	-1.18755	-1.08360
C	-4.84201	1.75334	-0.20775
H	-4.23703	1.69046	0.69786
C	-6.03669	0.77662	-0.12837
H	-6.48031	0.71007	0.86776
O	-5.21945	3.11205	-0.32953
H	-5.76184	3.18378	-1.13527
O	-7.02599	1.12600	-1.09274
H	-7.53239	1.86961	-0.72549
N	-4.75564	-1.27638	0.47174
C	-3.96678	-2.39039	0.26042
C	-4.51827	-0.88674	1.78480
C	-3.31573	-2.62082	1.47082
H	-5.01206	-0.03429	2.22445
N	-3.68439	-1.67514	2.41791
C	-2.29997	-3.62055	1.52280
N	-3.84206	-3.07354	-0.89921
C	-2.89891	-3.99699	-0.85135
N	-2.18195	-4.28333	0.28534
H	-1.42674	-4.96159	0.23074
O	-1.55129	-3.92757	2.46264
C	5.27023	1.97937	0.29894
C	6.58920	1.86176	0.57875
C	7.31424	0.86032	-0.14442
N	4.68119	1.19469	-0.65339
C	5.42440	0.20650	-1.34056
N	6.74334	0.07830	-1.06568
O	4.84222	-0.51269	-2.17858
N	8.62668	0.69648	0.09091
H	9.12219	-0.06214	-0.35843
H	9.08142	1.19749	0.84166
H	7.07054	2.48726	1.31951
H	4.62400	2.69852	0.78696
N	-2.63763	-4.76612	-1.94444
H	-1.72373	-5.20351	-1.99208
H	-2.95750	-4.37040	-2.82061
N	4.04226	-3.10351	-0.85765
O	4.29547	-0.92565	1.65723
C	3.30517	-1.41964	1.15223
C	2.93220	-2.90302	1.30879
C	1.66776	-3.43236	0.66496
H	0.82741	-2.86366	1.08078
H	1.56879	-4.47015	0.99955
C	1.63373	-3.36381	-0.87026
H	0.78370	-3.94762	-1.24224
H	1.48341	-2.33338	-1.20095
C	2.94952	-3.89292	-1.43369
O	2.37451	-0.73496	0.46886
O	3.56728	-3.53619	2.14963
H	2.93885	-3.86290	-2.53243
H	4.91519	-3.62423	-0.82713
H	4.20435	-2.23794	-1.37616
H	3.08855	-4.93744	-1.13431

CG-TS-6

E: -3388.20041
G: -3387.64205

O	-0.23449	4.26361	1.58544
C	1.18458	4.21210	1.40597
H	1.60629	3.88251	2.35943
H	1.59034	5.19689	1.15756
C	1.52036	3.20103	0.32031
H	1.20983	3.58310	-0.65699
O	2.94047	2.96502	0.30231
C	3.10037	1.67735	-0.28637
H	2.90820	1.70440	-1.36011
C	0.84087	1.82853	0.52137

H	0.35033	1.74663	1.49059
C	2.03152	0.84900	0.42863
H	2.35415	0.58401	1.43375
P	-1.20217	5.06037	0.50243
O	-1.67560	6.35742	1.40268
O	-0.36953	5.61680	-0.62401
O	-2.38736	4.15892	0.25179
H	-2.39541	6.10746	2.00874
O	-0.91638	-0.29958	-1.85971
P	-1.12438	0.37036	-0.51243
O	-0.11439	1.67721	-0.52301
O	-0.96635	-0.37166	0.78665
O	-2.59251	1.08760	-0.45756
C	-3.39958	1.28403	-1.62403
H	-2.81967	1.08346	-2.52743
H	-3.71932	2.33053	-1.63203
C	-4.63358	0.40490	-1.59491
H	-5.21661	0.63059	-2.49911
O	-4.26660	-0.99147	-1.61595
C	-5.14245	-1.76191	-0.80926
H	-5.52338	-2.59433	-1.40438
C	-5.54688	0.54336	-0.37442
H	-4.95143	0.63307	0.53781
C	-6.27096	-0.81646	-0.37978
H	-6.71990	-1.05710	0.58453
O	-6.39953	1.67077	-0.41425
H	-6.86160	1.66031	-1.27090
O	-7.25174	-0.87495	-1.41040
H	-7.98578	-0.29757	-1.14002
N	-4.40872	-2.32588	0.34785
C	-3.10381	-2.77406	0.36207
C	-4.89032	-2.55122	1.62646
C	-2.86788	-3.21862	1.66223
H	-5.90605	-2.32050	1.90488
N	-4.00179	-3.08185	2.43863
C	-1.55761	-3.67656	2.02521
N	-2.20309	-2.72805	-0.64945
C	-1.00613	-3.05294	-0.26496
N	-0.66522	-3.55582	0.94437
H	0.33753	-3.80234	1.06428
O	-1.16926	-4.11771	3.11474
C	4.95133	0.98192	1.14080
C	6.15729	0.39742	1.32207
C	6.88158	0.03168	0.13990
N	4.45960	1.21172	-0.11725
C	5.22536	0.87656	-1.26944
N	6.43761	0.29415	-1.09059
O	4.75965	1.11989	-2.39472
N	8.07332	-0.57796	0.26179
H	8.54704	-0.91347	-0.56658
H	8.40331	-0.88485	1.16652
H	6.55525	0.21308	2.31162
H	4.33117	1.30659	1.96703
N	0.08258	-2.76925	-1.17878
H	0.12537	-3.48029	-1.91683
H	-0.16525	-1.84168	-1.61438
N	4.74255	-3.35698	0.58603
O	1.82171	-1.58077	1.59705
C	1.73114	-1.50793	0.39021
C	1.69740	-2.78853	-0.51547
C	2.53678	-2.60905	-1.78946
H	2.10073	-1.82036	-2.41108
H	2.46563	-3.55342	-2.34246
C	4.01473	-2.26100	-1.53379
H	4.44820	-2.00522	-2.50707
H	4.07855	-1.35665	-0.92330
C	4.88740	-3.33527	-0.87958
O	1.77103	-0.35248	-0.30658
O	1.84801	-3.88123	0.15545
H	5.93478	-3.10088	-1.10181
H	3.77684	-3.62432	0.78579
H	5.31853	-4.11773	0.94646
H	4.67669	-4.30843	-1.35483

CG-7

E: -3311.77185
G: -3311.23985

O	-0.00140	4.30015	1.45043
C	1.40617	4.21055	1.20693
H	1.86266	3.89401	2.14879
H	1.82442	5.17883	0.91648
C	1.66887	3.16298	0.13495
H	1.33492	3.53114	-0.83904
O	3.08245	2.88628	0.07407
C	3.20129	1.57938	-0.46445
H	3.02120	1.56625	-1.54093
C	0.95660	1.81908	0.39693
H	0.51579	1.77081	1.39213
C	2.11159	0.79701	0.26836
H	2.45913	0.48966	1.25401
P	-1.00550	5.08588	0.39408
O	-1.29433	6.48931	1.21016
O	-0.25300	5.48436	-0.84949
O	-2.26692	4.25872	0.33669
H	-1.88182	6.32867	1.97007
O	-1.01843	-0.28964	-1.84174
P	-1.10647	0.39209	-0.50141
O	-0.05095	1.67473	-0.59396
O	-0.87887	-0.30969	0.81216
O	-2.54804	1.17439	-0.34817
C	-3.38163	1.37316	-1.49423
H	-2.78342	1.34876	-2.40735
H	-3.83599	2.36452	-1.40092
C	-4.49627	0.34375	-1.58220
H	-4.99423	0.48986	-2.55121
O	-3.96919	-0.99913	-1.53078
C	-4.81658	-1.84753	-0.76654
H	-4.98601	-2.76268	-1.33559
C	-5.56776	0.38437	-0.48689
H	-5.11283	0.56991	0.49026
C	-6.10930	-1.05830	-0.54799
H	-6.67203	-1.35226	0.33879
O	-6.54220	1.39129	-0.67580
H	-6.93506	1.25477	-1.55653
O	-6.90371	-1.25220	-1.71488
H	-7.77502	-0.85937	-1.53731
N	-4.16535	-2.21597	0.50479
C	-2.88169	-2.71019	0.63161
C	-4.63776	-2.06293	1.80000
C	-2.66117	-2.82836	2.00306
H	-5.62703	-1.69126	2.01223
N	-3.77327	-2.42696	2.71942
C	-1.36753	-3.21533	2.47042
N	-2.00424	-2.96383	-0.36701
C	-0.79066	-3.19878	0.07237
N	-0.48443	-3.41001	1.39068
H	0.49496	-3.51897	1.64247
O	-0.97277	-3.36067	3.63529
C	5.14729	1.25760	0.97021
C	6.36780	0.73015	1.22523
C	6.97759	-0.02471	0.17089
N	4.54607	1.07656	-0.24514
C	5.14870	0.27283	-1.24672
N	6.38322	-0.23444	-1.00689
O	4.52991	0.06651	-2.30634
N	8.20414	-0.54206	0.35622
H	8.60686	-1.13773	-0.35474
H	8.65819	-0.48201	1.25699
H	6.86127	0.87645	2.17744

H	4.59535	1.85300	1.68640
N	0.22964	-3.34657	-0.86596
N	3.04898	-4.79132	0.35594
O	1.81845	-1.72900	1.30700
C	1.62280	-1.53062	0.12532
C	1.28974	-2.62699	-0.88124
C	2.34438	-2.82291	-1.93157
H	2.39585	-1.88929	-2.50551
H	2.02198	-3.62302	-2.60399
C	3.74667	-3.11813	-1.35764
H	4.47213	-2.96333	-2.16318
H	3.99320	-2.39366	-0.57195
C	3.91080	-4.53559	-0.81212
O	1.76796	-0.35880	-0.50524
H	4.95008	-4.65951	-0.48953
H	2.09872	-4.96622	0.03263
H	3.34589	-5.66765	0.78349
H	3.74579	-5.25300	-1.63229

CG-TS-7

E: -3444.76023
G: -3444.16895

O	-0.38793	4.63025	-0.63881
C	-1.79825	4.40787	-0.53419
H	-2.17877	4.35671	-1.55828
H	-2.29584	5.23162	-0.01343
C	-2.06127	3.07613	0.15766
H	-1.91450	3.15959	1.23886
O	-3.41534	2.67110	-0.11226
C	-3.47828	1.28113	0.14771
H	-3.59482	1.07783	1.21437
C	-1.17684	1.93143	-0.35846
H	-0.85858	2.11764	-1.38608
C	-2.13776	0.71265	-0.35167
H	-2.24110	0.29601	-1.35006
P	0.46178	5.24016	0.64095
O	0.31954	6.86404	0.38538
O	-0.25756	4.97843	1.93910
O	1.88527	4.79366	0.41424
H	0.78446	7.12620	-0.42921
O	1.33420	0.00183	1.51743
P	1.09425	0.65611	0.18157
O	-0.05429	1.81711	0.49503
O	0.69176	-0.06667	-1.07793
O	2.39962	1.57613	-0.23650
C	3.49873	1.71500	0.66892
H	3.14868	1.64474	1.70083
H	3.92297	2.71142	0.51297
C	4.59977	0.69407	0.43106
H	5.33615	0.83341	1.23609
O	4.08998	-0.65329	0.49061
C	4.79771	-1.50528	-0.40757
H	5.15886	-2.36873	0.15360
C	5.34436	0.75255	-0.90479
H	4.63849	0.88110	-1.73071
C	5.95342	-0.66381	-0.95942
H	6.28509	-0.95461	-1.95678
O	6.28012	1.80780	-1.00231
H	6.87742	1.73921	-0.23678
O	7.02836	-0.79448	-0.03325
H	7.79518	-0.33719	-0.41761
N	3.89765	-1.99010	-1.46612
C	2.64183	-2.53883	-1.26082
C	4.03492	-1.86640	-2.84324
C	2.10427	-2.71325	-2.53670
H	4.92796	-1.46783	-3.29651
N	2.99373	-2.29713	-3.51458
C	0.74928	-3.13575	-2.65369
N	2.04545	-2.76003	-0.07454
C	0.74718	-3.02076	-0.16880
N	0.15960	-3.30271	-1.38675
H	-0.84708	-3.42607	-1.42176
O	0.07905	-3.33286	-3.68119
C	-5.02955	1.20963	-1.74956
C	-6.01516	0.60745	-2.45859
C	-6.59246	-0.57195	-1.88902
N	-4.63697	0.71365	-0.53851
C	-5.17038	-0.50124	-0.04422
N	-6.17590	-1.09359	-0.73078
O	-4.69889	-0.97893	1.00629
N	-7.58187	-1.20505	-2.54189
H	-8.02743	-2.00775	-2.11795
H	-7.97828	-0.81460	-3.38541
H	-6.34837	1.00077	-3.41034
H	-4.51982	2.10480	-2.08013
N	0.02869	-3.04128	0.99470
O	-2.24776	-1.90650	-0.96562
C	-1.78125	-1.58162	0.11110
C	-1.20192	-2.55282	1.15323
C	-1.59568	-2.20634	2.58116
H	-0.97717	-1.34136	2.84330
O	-1.69275	-0.31786	0.54361
C	-1.36308	-3.31752	3.60862
H	-2.14612	-4.08039	3.53046
H	-0.41063	-3.81393	3.39996
C	-1.34108	-2.79769	5.04656
H	-1.15932	-3.65854	5.70957
H	-2.33144	-2.40473	5.31289
N	-0.34947	-1.72270	5.21443
H	-0.13026	-1.63146	6.20513
H	0.51499	-2.01285	4.75241
H	-2.63709	-1.87231	2.60425
O	1.95186	-2.57937	3.15121
H	1.32265	-2.67690	2.39685
H	1.87593	-3.43353	3.60643
N	-2.60803	-3.93816	0.82274
H	-2.54597	-4.30318	-0.12619
H	-3.53442	-3.52964	0.95425
H	-2.47546	-4.70097	1.48458

CG-8

E: -3368.83222
G: -3368.25104

O	0.89721	4.27723	0.89532
C	2.16703	3.90504	0.34738
H	2.79732	3.62550	1.19643
H	2.63505	4.74789	-0.17184
C	2.02251	2.71534	-0.59875
H	1.47005	3.00793	-1.49109
O	3.35180	2.28230	-0.98440
C	3.47501	0.88299	-0.78150
H	3.19799	0.31706	-1.67165
C	1.32523	1.51463	0.05005
H	0.77080	1.79606	0.94444
C	2.50794	0.58824	0.36221
H	2.95134	0.83132	1.32858
P	-0.18561	5.14219	-0.00470
O	0.53610	6.62168	-0.08785
O	-0.23207	4.64307	-1.42685
O	-1.42517	5.19592	0.85192
H	0.52778	7.05859	0.78251
O	-1.42464	-0.60420	-1.57025
P	-0.90702	0.15093	-0.37744
O	0.46257	0.90385	-0.91302
O	-0.63036	-0.54050	0.94145
O	-1.85569	1.41449	0.04695
C	-2.56776	2.18326	-0.93710
H	-2.18263	1.98682	-1.94102

H	-2.41262	3.23720	-0.69925
C	-4.05023	1.88046	-0.86395
H	-4.57106	2.53350	-1.57687
O	-4.30030	0.50867	-1.23286
C	-5.14889	-0.12978	-0.28902
H	-5.91252	-0.68112	-0.83784
C	-4.66762	2.06112	0.53456
H	-3.92234	1.84815	1.30373
C	-5.76828	0.98164	0.55798
H	-6.03707	0.65799	1.56646
O	-5.13157	3.37304	0.78652
H	-5.80803	3.56922	0.11423
O	-6.92181	1.43094	-0.14822
H	-7.40787	2.02595	0.44701
N	-4.39778	-1.11484	0.49356
C	-3.90861	-2.28626	-0.03936
C	-3.92263	-1.05990	1.79982
C	-3.17986	-2.89010	0.98966
H	-4.15206	-0.23470	2.45680
N	-3.21623	-2.11070	2.13859
C	-2.53167	-4.12229	0.71838
N	-4.12441	-2.73904	-1.29012
C	-3.52531	-3.89380	-1.53373
N	-2.77419	-4.55719	-0.59151
H	-2.30586	-5.42086	-0.85316
O	-1.79723	-4.81229	1.46254
C	5.55337	1.30306	0.42631
C	6.82838	0.98995	0.75543
C	7.40160	-0.15031	0.10328
N	4.86290	0.55820	-0.49013
C	5.44722	-0.58352	-1.09425
N	6.72929	-0.89339	-0.78003
O	4.76884	-1.25995	-1.88879
N	8.67108	-0.49460	0.37634
H	9.06837	-1.32240	-0.04748
H	9.20364	0.00038	1.07795
H	7.38966	1.57627	1.47161
H	5.02433	2.14982	0.84567
N	-3.59018	-4.45232	-2.76668
O	1.80659	-0.90448	2.56983
C	1.64586	-1.32085	1.44188
C	0.86437	-2.59058	1.15237
C	0.82033	-3.22507	-0.19306
H	0.38751	-2.50561	-0.89467
H	0.15744	-4.09118	-0.13945
C	2.21686	-3.66289	-0.69039
H	2.05651	-4.20022	-1.63267
H	2.83607	-2.79200	-0.91578
C	2.94343	-4.57037	0.29675
O	2.13044	-0.79074	0.32081
H	2.27725	-5.40238	0.58016
H	-3.40184	-5.44345	-2.85997
H	-4.30447	-4.08020	-3.38005
N	0.28339	-3.10874	2.17583
H	-0.39936	-3.88353	2.06366
H	0.35254	-2.63171	3.07584
N	4.22544	-5.01046	-0.26971
H	4.67195	-5.63415	0.40232
H	4.02172	-5.59814	-1.07933
H	3.15721	-4.01579	1.21921

CG-TS-8

E: -3558.12043
G: -3557.52379

O	-0.38044	-2.38640	-2.75614
C	0.63595	-2.75969	-1.81925
H	1.59110	-2.71367	-2.35102
H	0.49105	-3.78227	-1.45673
C	0.62313	-1.79207	-0.64118
H	-0.36762	-1.79923	-0.19317
O	1.57383	-2.25466	0.35134
C	2.59016	-1.29350	0.54846
H	2.42111	-0.71293	1.45619
C	1.03672	-0.36309	-1.03076
H	0.85750	-0.16952	-2.08724
C	2.52400	-0.36744	-0.67075
H	3.12187	-0.74791	-1.50194
P	-1.91348	-2.96921	-2.55854
O	-1.72696	-4.54815	-3.00239
O	-2.29238	-3.02211	-1.09955
O	-2.73964	-2.22173	-3.57189
H	-1.50218	-4.61175	-3.94792
C	4.19519	-3.03009	-0.08552
C	5.41918	-3.60629	-0.02010
C	6.35434	-3.03182	0.90055
N	3.88217	-1.95946	0.70402
C	4.84693	-1.38061	1.56577
N	6.06749	-1.96593	1.65295
O	4.54307	-0.36191	2.21349
N	7.57521	-3.57915	1.02618
H	8.25186	-3.16109	1.65017
H	7.85455	-4.36257	0.45278
H	5.67561	-4.46432	-0.62821
H	3.40603	-3.38508	-0.73608
O	2.50711	2.00057	-2.13320
C	2.94678	1.95938	-1.00198
C	3.44462	3.17494	-0.24483
C	4.54436	3.01660	0.77883
H	4.18703	2.37593	1.58738
H	4.75645	4.00607	1.19645
C	5.82462	2.39358	0.18596
H	6.56387	2.37061	0.99660
H	5.63525	1.35272	-0.09707
C	6.40317	3.15373	-1.00331
O	3.05546	0.88528	-0.23093
H	6.48545	4.22233	-0.74301
N	3.30535	4.31911	-0.89614
H	3.70010	5.17447	-0.52075
H	2.67928	4.37853	-1.69356
N	7.68047	2.55176	-1.41350
H	8.04466	3.08703	-2.20142
H	8.35181	2.70292	-0.65939
H	5.71642	3.09145	-1.85469
C	1.40128	3.19064	1.58029
H	2.25649	3.04755	0.73127
O	1.34559	2.21850	2.33803
O	0.84873	4.28363	1.45382
O	-1.38617	2.37621	0.08386
P	-1.18872	0.98865	-0.47025
O	0.41649	0.64627	-0.23823
O	-1.59189	0.62744	-1.87576
O	-1.88663	-0.12393	0.51420
C	-1.45094	-0.21017	1.87862
H	-1.17050	0.77899	2.25340
H	-0.57787	-0.87010	1.94416
C	-2.57171	-0.78125	2.72062
H	-2.20698	-0.88577	3.74901
O	-3.68784	0.13755	2.74554
C	-4.83434	-0.46797	2.17768
H	-5.70773	-0.05537	2.68291
C	-3.13611	-2.13781	2.23744
H	-2.90954	-2.30067	1.18338
C	-4.65806	-1.96183	2.43858
H	-5.26016	-2.60207	1.79087
O	-2.60067	-3.25070	2.93007
H	-2.86980	-3.15272	3.86126
O	-5.00109	-2.17205	3.80654
H	-5.13571	-3.12639	3.92616
N	-4.93544	-0.15634	0.74413

C	-4.83300	1.11902	0.22829
C	-4.91752	-1.01277	-0.35069
C	-4.78939	0.95858	-1.15548
H	-4.97756	-2.08371	-0.24027
N	-4.85258	-0.38394	-1.49926
C	-4.60441	2.10909	-1.97712
N	-4.78204	2.26967	0.93637
C	-4.61060	3.33642	0.17604
N	-4.53912	3.28095	-1.19522
H	-4.37269	4.14037	-1.71137
O	-4.49831	2.18079	-3.20997
N	-4.45129	4.56038	0.74645
H	-4.66824	5.37682	0.18665
H	-4.73150	4.61985	1.71766

CG-Orn

E: -3369.58774
G: -3368.99580

O	2.07577	4.32856	0.24582
C	3.28741	3.63255	-0.06586
H	3.82553	3.51518	0.87904
H	3.90706	4.21825	-0.75205
C	2.99675	2.25332	-0.64198
H	2.57437	2.32919	-1.64749
O	4.24788	1.53934	-0.70281
C	3.94990	0.15838	-0.63029
H	3.64706	-0.24226	-1.60006
C	2.04329	1.40634	0.22094
H	1.87914	1.85934	1.19962
C	2.79483	0.05956	0.37223
H	3.18319	-0.05763	1.38320
P	1.20634	5.08712	-0.93631
O	2.31349	6.13937	-1.55584
O	0.87095	4.14548	-2.06531
O	0.12662	5.82699	-0.18493
H	2.55541	6.81113	-0.89328
O	-1.27704	-0.13206	-0.63442
P	-0.51584	0.71224	0.35772
O	0.81046	1.26443	-0.47071
O	-0.12134	0.20605	1.72077
O	-1.30583	2.11949	0.67057
C	-1.94623	2.88592	-0.35966
H	-1.60754	2.57642	-1.35210
H	-1.65774	3.92895	-0.20760
C	-3.45673	2.77698	-0.25898
H	-3.90085	3.52306	-0.93207
O	-3.89051	1.46918	-0.67837
C	-4.82670	0.91018	0.23304
H	-5.69798	0.57041	-0.32715
C	-4.01152	2.97729	1.16296
H	-3.26805	2.66887	1.89954
C	-5.22032	2.01950	1.21147
H	-5.42243	1.64051	2.21719
O	-4.33042	4.32333	1.46251
H	-4.99738	4.60635	0.81194
O	-6.37874	2.64627	0.66508
H	-6.69087	3.29033	1.32309
N	-4.26957	-0.28099	0.86545
C	-4.28094	-1.52581	0.27501
C	-3.45336	-0.39462	1.98403
C	-3.50525	-2.33995	1.10094
H	-3.25456	0.44585	2.63144
N	-3.00303	-1.61219	2.17171
C	-3.34924	-3.71151	0.74811
N	-4.93147	-1.85700	-0.86057
C	-4.79718	-3.13344	-1.17845
N	-4.08560	-4.02396	-0.41102
H	-3.98631	-4.98112	-0.73826
O	-2.68883	-4.60284	1.30983
C	6.00658	-0.01112	0.69186
C	7.05309	-0.72056	1.18063
C	7.18842	-2.06968	0.72154
N	5.14571	-0.56785	-0.21063
C	5.25715	-1.93102	-0.57702
N	6.31927	-2.63846	-0.11829
O	4.38190	-2.42876	-1.30894
N	8.23612	-2.80648	1.13448
H	8.27751	-3.78746	0.89222
H	8.85224	-2.46049	1.85686
H	7.75391	-0.28553	1.88151
H	5.80512	1.01643	0.96443
N	-5.33658	-3.60842	-2.33055
N	-1.99914	-4.15357	-3.00915
O	2.53145	-2.17212	1.91618
C	1.81257	-2.03936	0.94368
C	0.67526	-2.95144	0.52738
C	1.07688	-3.69625	-0.76837
H	1.54078	-2.99218	-1.46811
H	1.84836	-4.43124	-0.50246
C	-0.09339	-4.39344	-1.46774
H	0.30795	-5.03546	-2.26213
H	-0.61722	-5.05418	-0.76302
C	-1.09710	-3.42807	-2.09582
O	1.94423	-1.03210	0.04947
H	-1.63526	-2.88015	-1.30938
H	-2.42293	-4.91873	-2.48260
H	-2.78344	-3.54301	-3.23879
H	-0.55747	-2.67127	-2.67906
H	-5.48032	-4.60746	-2.41952
H	-6.04711	-3.02433	-2.75385
N	0.28477	-3.80592	1.64579
H	-0.65606	-4.15713	1.46619
H	0.90178	-4.61791	1.66716
H	-0.16206	-2.28646	0.30459

AG-1

E: -4082.05222
G: -4081.54337

O	2.24434	-2.00819	-1.58086
C	1.53706	-3.22830	-1.85524
H	2.26757	-4.03362	-1.97278
H	0.94878	-3.13842	-2.77188
C	0.60684	-3.54803	-0.69831
H	0.07604	-4.47412	-0.93257
O	1.34479	-3.78315	0.53058
C	1.30384	-2.64230	1.36053
H	0.85218	-2.90380	2.32193
C	-0.38462	-2.44007	-0.33131
H	-0.73813	-1.87562	-1.19307
C	0.44500	-1.59135	0.63290
H	1.06336	-0.89250	0.07812
O	-0.36056	-0.89170	1.58038
P	2.45635	-0.81352	-2.66372
O	3.72847	-0.97899	-3.43462
O	1.18727	-0.84568	-3.62677
O	2.30371	0.46162	-1.75797
C	-0.38910	0.44219	1.48419
C	-1.14455	1.06545	2.67057
C	-1.06553	2.55046	2.80186
H	-0.27317	2.96718	2.18013
H	-0.84640	2.78107	3.85125
C	-2.41348	3.28728	2.48355
O	-2.30547	4.53989	2.34928
O	0.12442	1.09688	0.60152
O	-1.76760	0.34337	3.43324
O	-3.47206	2.61545	2.44019
P	3.65049	1.46090	-1.11248
O	4.65649	0.41286	-0.64436

O	4.06924	2.28420	-2.32727
O	2.89597	2.21319	-0.01922
H	0.39467	-0.24920	-3.36163
O	-1.46918	-2.98752	0.41239
P	-2.86661	-3.44957	-0.33212
O	-2.87727	-4.94822	-0.51203
O	-3.12823	-2.56465	-1.52909
O	-3.90218	-3.14665	0.90078
C	-3.82778	-2.00970	1.77759
H	-2.84400	-1.96559	2.24961
H	-4.57786	-2.20185	2.54870
C	-4.14776	-0.66943	1.14327
H	-4.37164	0.03833	1.95240
O	-3.00772	-0.18225	0.41454
C	-3.49560	0.82649	-0.45687
H	-3.66898	1.75734	0.09057
C	-5.32541	-0.65296	0.14082
H	-5.49645	-1.65498	-0.25439
C	-4.83233	0.30049	-0.99342
H	-4.69329	-0.24886	-1.92626
O	-6.53321	-0.19800	0.72719
H	-6.61429	0.73627	0.44850
O	-5.69987	1.41027	-1.18111
H	-6.42065	1.12631	-1.76878
N	-2.48281	1.10228	-1.44628
C	-1.65033	2.20655	-1.43829
C	-1.93806	0.19839	-2.33670
C	-0.66882	1.94134	-2.38567
H	-2.35941	-0.78740	-2.47412
N	-0.86622	0.67732	-2.93103
C	0.35681	2.90656	-2.60311
N	-1.77198	3.27666	-0.63790
C	-0.75856	4.12543	-0.74874
N	0.22875	3.98052	-1.69653
N	2.67345	-2.17926	1.60352
C	3.04570	-0.99853	2.21253
C	3.84150	-2.79837	1.21212
C	4.44318	-0.98608	2.16372
H	3.82316	-3.74685	0.69928
N	4.92628	-2.12761	1.54060
C	5.07175	0.17656	2.65109
N	2.25889	-0.03550	2.72146
C	2.97182	1.01081	3.14470
N	4.30548	1.17133	3.14695
H	2.39774	1.84190	3.54699
N	6.42288	0.32043	2.67003
H	6.95618	-0.27170	2.04568
H	6.77200	1.26477	2.78057
O	1.27081	2.89894	-3.43818
H	0.97257	4.67151	-1.73834
N	-0.70568	5.20655	0.06400
H	0.18579	5.68177	0.14486
H	-1.23906	5.09109	0.93681

AG-TS-1

E: -4082.01203
G: -4081.50254

O	2.14217	-3.56622	0.71141
C	0.96910	-4.36132	0.60742
H	1.00464	-5.12345	1.39465
H	0.90367	-4.87783	-0.35733
C	-0.30421	-3.53612	0.80254
H	-1.14552	-4.21870	0.92996
O	-0.20247	-2.73234	2.02209
C	-0.09458	-1.36324	1.68772
H	-1.01237	-0.82085	1.92741
C	-0.61842	-2.52828	-0.31576
H	-0.30035	-2.87372	-1.30069
C	0.18795	-1.35162	0.18360
H	1.23765	-1.56674	0.02472
O	-0.06840	-0.04150	-0.33532
P	3.21426	-3.41174	-0.58899
O	3.92382	-4.76007	-0.71818
O	2.38193	-2.98501	-1.79700
O	4.15535	-2.30336	-0.00827
C	0.66138	0.27929	-1.43284
C	1.43964	1.58852	-1.23770
C	2.55083	1.87146	-2.22419
H	2.82836	0.96917	-2.76862
H	2.17829	2.61667	-2.93404
C	3.74766	2.46789	-1.48016
O	4.45428	1.68585	-0.71328
O	0.70749	-0.39476	-2.43857
O	1.13071	2.37539	-0.35832
O	4.00437	3.67380	-1.59718
P	4.22040	-0.24116	-0.53058
O	5.14961	-0.11826	0.79763
O	4.94184	-0.56010	-1.81388
O	2.72891	-0.13189	-0.28263
N	0.98629	-0.71605	2.43669
C	1.06071	0.66309	2.59748
C	2.28533	-1.17659	2.51216
C	2.41719	0.94822	2.76755
H	2.51393	-2.21877	2.36158
N	3.16472	-0.22587	2.74926
C	2.77257	2.30897	2.79073
N	0.05991	1.55417	2.60678
C	0.51496	2.80538	2.73224
N	1.78624	3.23103	2.80679
H	-0.24097	3.58426	2.76734
N	4.06961	2.72484	2.81734
H	4.74780	2.08475	2.41870
H	4.21456	3.69652	2.56719
H	4.56808	-0.24593	1.59906
O	-1.99445	-2.14910	-0.31529
P	-3.10434	-3.06582	-1.12208
O	-3.06722	-4.48421	-0.60438
O	-3.02811	-2.78572	-2.60114
O	-4.43668	-2.27535	-0.58243
C	-4.72546	-2.23189	0.82383
H	-3.94806	-2.74211	1.39884
H	-5.67672	-2.74580	0.99665
C	-4.86655	-0.79926	1.29580
H	-5.19052	-0.81989	2.34551
O	-3.59860	-0.11837	1.20989
C	-3.76844	1.22754	0.78945
H	-3.30888	1.90628	1.50911
C	-5.84548	0.06552	0.48561
H	-5.79518	-0.20008	-0.57214
C	-5.27973	1.48145	0.69879
H	-5.53057	2.17355	-0.10864
O	-7.19632	-0.09012	0.87470
H	-7.25733	0.21125	1.79893
O	-5.69786	1.99751	1.96162
H	-6.58917	2.36611	1.84259
N	-3.06936	1.44127	-0.47446
C	-2.24330	2.50403	-0.76012
C	-3.09895	0.64965	-1.61412
C	-1.82099	2.30055	-2.07580
H	-3.67157	-0.26264	-1.64856
N	-2.37007	1.13310	-2.59290
C	-0.93059	3.24777	-2.66045
N	-1.92910	3.51759	0.07246
C	-1.09128	4.38907	-0.46740
N	-0.66043	4.30686	-1.76963
H	0.01790	4.98678	-2.10150
O	-0.40506	3.23625	-3.78488
N	-0.59976	5.40772	0.27860
H	-0.21440	6.21857	-0.18978

H	-1.07285	5.58089	1.15620

AG-2

E: -4082.02488
G: -4081.51887

O	0.08987	3.00916	1.54935
C	1.26341	3.05645	0.72830
H	2.08415	2.64062	1.32017
H	1.52139	4.08275	0.45091
C	1.03114	2.22269	-0.52945
H	0.19256	2.63071	-1.09347
O	2.21383	2.29194	-1.36948
C	2.85815	1.03421	-1.41858
H	2.69606	0.54194	-2.38200
C	0.80857	0.74165	-0.22214
H	0.35942	0.59014	0.75813
C	2.23625	0.20879	-0.28802
H	2.76148	0.38540	0.65365
O	2.36732	-1.17470	-0.63301
P	-1.07357	4.16623	1.36834
O	-0.42284	5.43874	2.19084
O	-1.14797	4.62869	-0.06574
O	-2.27460	3.62231	2.10041
C	2.16682	-2.06163	0.34542
C	2.70694	-3.44870	-0.04351
C	4.08017	-3.49930	-0.66511
H	4.33680	-4.52918	-0.91260
H	4.07634	-2.88438	-1.57410
C	5.13682	-2.90423	0.27176
O	6.21278	-3.45284	0.46847
O	1.62474	-1.83940	1.40689
O	2.03482	-4.42812	0.21851
O	4.72870	-1.77240	0.80356
P	5.48343	-0.98459	2.24746
O	4.55370	0.22530	2.31652
O	6.92014	-0.71211	1.81732
O	5.28872	-2.05117	3.31795
H	-0.39357	5.25169	3.14621
O	-2.04736	-1.00016	-2.09341
P	-1.48265	-0.40821	-0.82971
O	0.03387	0.11361	-1.24460
O	-1.42115	-1.18139	0.46214
O	-2.21739	1.01262	-0.44904
C	-2.65862	1.89203	-1.49673
H	-2.35732	1.50938	-2.47482
H	-2.19833	2.86919	-1.33387
C	-4.16275	2.04873	-1.43923
H	-4.46662	2.75906	-2.22123
O	-4.79210	0.77694	-1.68467
C	-5.93745	0.60615	-0.85988
H	-6.82066	0.42735	-1.47522
C	-4.70837	2.52634	-0.08500
H	-4.10002	2.13015	0.72901
C	-6.11355	1.89717	-0.04771
H	-6.46432	1.69597	0.96874
O	-4.69754	3.93419	0.06438
H	-5.17118	4.31008	-0.69859
O	-7.04789	2.70583	-0.75861
H	-7.17368	3.51700	-0.23690
N	-5.77210	-0.58793	-0.04262
C	-6.63461	-1.66065	-0.00512
C	-4.73746	-0.88522	0.83261
C	-6.07057	-2.55369	0.90539
H	-3.90980	-0.20903	0.97487
N	-4.88165	-2.05136	1.41801
C	-6.74560	-3.78186	1.16615
N	-7.78010	-1.79004	-0.71064
C	-8.40240	-2.93350	-0.47676
N	-7.93166	-3.87643	0.40537
N	4.29096	1.22168	-1.26614
C	5.26603	0.34420	-1.70061
C	4.92159	2.11205	-0.41790
C	6.44920	0.78763	-1.10252
H	4.37010	2.90247	0.06868
N	6.21638	1.90775	-0.31538
C	7.59701	0.00528	-1.33426
N	5.13483	-0.71402	-2.51667
C	6.29746	-1.35538	-2.67846
N	7.49582	-1.06942	-2.14497
H	6.27096	-2.22839	-3.32506
N	8.80916	0.31361	-0.80149
H	8.79578	0.86806	0.04636
H	9.49383	-0.43349	-0.81011
O	-6.42745	-4.70354	1.93137
H	-8.46199	-4.73351	0.53636
N	-9.53403	-3.24506	-1.16197
H	-10.15717	-3.93667	-0.76190
H	-9.99241	-2.46335	-1.61414

AG-TS-2

E: -4271.76427
G: -4271.23060

O	0.64636	-3.05864	1.90107
C	-0.54932	-3.34738	1.16731
H	-1.38854	-2.97236	1.76083
H	-0.68275	-4.42276	1.01763
C	-0.49534	-2.63598	-0.18087
H	0.35945	-2.99246	-0.75594
O	-1.70608	-2.95384	-0.91850
C	-2.45371	-1.77928	-1.16039
H	-2.31811	-1.41703	-2.18349
C	-0.45019	-1.11115	-0.04066
H	-0.03045	-0.80283	0.91561
C	-1.92989	-0.75127	-0.15652
H	-2.42819	-0.86498	0.80839
O	-2.20391	0.54893	-0.67911
P	1.93460	-4.08295	1.77788
O	1.47280	-5.32943	2.75413
O	2.01159	-4.68100	0.39508
O	3.08651	-3.32812	2.39214
C	-2.09417	1.57683	0.16777
C	-2.61489	2.88184	-0.45804
C	-3.80918	2.80301	-1.35745
H	-3.92893	3.74837	-1.88899
H	-3.72092	1.97383	-2.06513
C	-5.10624	2.54978	-0.52668
O	-6.22495	2.60568	-1.10405
O	-1.62899	1.51866	1.28690
O	-2.08631	3.92228	-0.10372
O	-4.80907	1.38449	0.29783
P	-5.73518	1.02647	1.62532
O	-4.93724	-0.33940	2.07128
O	-7.14877	0.68788	1.21457
O	-5.48049	2.05970	2.69597
H	1.42998	-5.03989	3.68310
O	2.17652	0.77414	-2.09538
P	1.71881	0.19234	-0.78465
O	0.25455	-0.50602	-1.12317
O	1.61445	1.01866	0.47082
O	2.61181	-1.12278	-0.36309
C	3.09049	-2.01443	-1.38385
H	2.68864	-1.73442	-2.36060
H	2.75780	-3.02394	-1.13079
C	4.60323	-2.00121	-1.41722
H	4.93674	-2.70410	-2.19368
O	5.07260	-0.67805	-1.74219
C	6.24111	-0.35789	-1.00021
H	7.05486	-0.09141	-1.67641

C	5.28379	-2.36443	-0.08871
H	4.69755	-1.99152	0.75205
C	6.61787	-1.60256	-0.18440
H	7.03679	-1.34188	0.79112
O	5.42244	-3.75774	0.11363
H	5.95021	-4.10000	-0.62986
O	7.56060	-2.33693	-0.96473
H	7.91899	-3.04167	-0.39913
N	5.99594	0.82517	-0.18480
C	6.78271	1.95442	-0.16415
C	4.98026	1.03737	0.73637
C	6.19429	2.79646	0.77880
H	4.20923	0.30223	0.90261
N	5.06472	2.20554	1.33020
C	6.79491	4.06488	1.03135
N	7.88821	2.16986	-0.91092
C	8.43941	3.35073	-0.68610
N	7.94209	4.24896	0.22757
N	-3.86690	-2.06293	-0.99010
C	-4.90179	-1.39695	-1.62258
C	-4.43491	-2.77389	0.04486
C	-6.05830	-1.78912	-0.94416
H	-3.83076	-3.36882	0.71411
N	-5.74738	-2.65595	0.09989
C	-7.27126	-1.23523	-1.40029
N	-4.83464	-0.55195	-2.66214
C	-6.04495	-0.08530	-2.98961
N	-7.23448	-0.37828	-2.44179
H	-6.07291	0.61949	-3.81589
N	-8.47472	-1.56580	-0.86605
H	-8.46786	-1.92464	0.08101
H	-9.23973	-0.93668	-1.08032
O	6.44616	4.95355	1.82182
H	8.41717	5.13936	0.34748
N	9.51746	3.74603	-1.41300
H	10.11167	4.47105	-1.02839
H	10.00632	3.00126	-1.89468
H	-5.31329	-1.11862	1.61695
C	-5.37565	4.55524	1.18962
H	-5.01787	3.47272	0.33786
O	-4.38351	4.92278	1.77527
O	-6.55336	4.73424	0.98456

AG-3

E: -3439.96879
G: -3439.46439

O	0.30017	-2.91146	1.34671
C	-1.05529	-2.73639	0.91762
H	-1.57888	-2.21030	1.72061
H	-1.55392	-3.69663	0.76006
C	-1.10807	-1.93827	-0.38348
H	-0.48503	-2.42068	-1.13923
O	-2.47916	-1.93586	-0.85937
C	-3.07368	-0.66014	-0.66080
H	-3.22370	-0.15093	-1.61545
C	-0.73652	-0.46788	-0.22243
H	0.06303	-0.30672	0.50223
C	-2.08495	0.12234	0.20889
H	-2.26793	-0.03723	1.27170
O	-2.19153	1.51141	-0.10558
P	1.03803	-4.35438	1.03519
O	0.13858	-5.40369	1.93107
O	0.82177	-4.75545	-0.40111
O	2.42247	-4.21340	1.62340
C	-1.61076	2.35207	0.76564
C	-1.42755	3.73788	0.13866
C	-0.47686	4.67127	0.84165
H	-1.05596	5.55772	1.14390
C	0.58241	5.17711	-0.11176
O	1.68236	5.55413	0.25252
O	-1.30014	2.07631	1.90560
O	-2.03025	4.04437	-0.87417
H	0.26603	-5.23998	2.88279
O	0.48409	1.84072	-3.04603
P	0.68048	1.29994	-1.65651
O	-0.40867	0.06617	-1.50856
O	0.64936	2.20373	-0.44227
O	2.09085	0.47833	-1.53217
C	2.60255	-0.19054	-2.69189
H	2.98674	0.55399	-3.39570
H	1.82058	-0.77530	-3.19072
C	3.70698	-1.13198	-2.25923
H	4.21100	-1.50217	-3.15958
O	4.68522	-0.42325	-1.46375
C	4.80706	-1.03147	-0.18929
H	5.84055	-0.90747	0.13527
C	3.25483	-2.33982	-1.41322
H	2.32284	-2.12514	-0.88551
C	4.42259	-2.49138	-0.41619
H	4.15283	-3.03987	0.48486
O	3.01939	-3.49990	-2.18727
H	3.88871	-3.76814	-2.53795
O	5.52912	-3.11457	-1.06961
H	5.45053	-4.07043	-0.91689
N	3.92068	-0.38884	0.79658
C	3.79825	0.97268	0.98542
C	2.93307	-0.97697	1.58129
C	2.76160	1.12713	1.90566
H	2.78840	-2.04740	1.62077
N	2.23585	-0.10330	2.27034
C	2.33770	2.44030	2.25488
N	4.51736	1.94327	0.37798
C	4.09349	3.16044	0.67448
N	3.09458	3.41644	1.58249
N	-4.38522	-0.81370	-0.06700
C	-5.57326	-0.30242	-0.55349
C	-4.69909	-1.48256	1.10107
C	-6.54561	-0.69943	0.36844
H	-3.93662	-1.98715	1.67709
N	-5.97880	-1.44210	1.39811
C	-7.86667	-0.28729	0.09996
N	-5.77206	0.42556	-1.66286
C	-7.06187	0.75015	-1.80580
N	-8.09978	0.44890	-1.00981
H	-7.30621	1.34182	-2.68397
N	-8.91406	-0.62963	0.88647
H	-8.72468	-0.96989	1.81989
H	-9.78247	-0.12781	0.75081
O	1.42979	2.78283	3.03045
H	2.71587	4.36226	1.62157
N	4.62465	4.23631	0.03724
H	4.52742	5.14781	0.46710
H	5.49684	4.06954	-0.44829
H	0.29027	5.22993	-1.17494
H	-0.01576	4.23410	1.72728

AG-TS-3

E: -3439.92703
G: -3439.41888

O	0.08549	3.68415	1.55259
C	1.47658	3.69411	1.21320
H	2.01340	3.41993	2.12519
H	1.79569	4.69565	0.90637
C	1.79290	2.67047	0.12301
H	1.47355	3.03208	-0.85478
O	3.22619	2.45915	0.10254
C	3.45915	1.07448	-0.13806
H	3.36327	0.82149	-1.19862

C	1.14390	1.29715	0.36723
H	0.42706	1.31097	1.18587
C	2.35722	0.40903	0.67923
H	2.59201	0.45760	1.74260
P	-1.04154	4.36525	0.55652
O	-0.75841	5.97433	0.77827
O	-0.72166	4.10794	-0.89494
O	-2.36450	3.95915	1.15674
H	-0.86536	6.21729	1.71526
O	-0.67620	-0.82720	-2.19328
P	-0.72826	-0.17936	-0.82168
O	0.52147	0.88735	-0.85130
O	-0.69892	-1.00525	0.43897
O	-2.03707	0.78347	-0.67218
C	-2.61353	1.44090	-1.81269
H	-2.28945	0.94774	-2.73222
H	-2.28426	2.48320	-1.82565
C	-4.12055	1.38659	-1.70612
H	-4.53787	1.91525	-2.57304
O	-4.57272	0.01456	-1.75829
C	-5.48920	-0.26143	-0.72298
H	-6.30563	-0.85674	-1.13674
C	-4.72368	1.97215	-0.42095
H	-4.04612	1.82998	0.42332
C	-5.97952	1.10164	-0.22373
H	-6.33371	1.10472	0.80849
O	-4.97088	3.36364	-0.49315
H	-5.45961	3.53814	-1.31621
O	-7.02490	1.47919	-1.11322
H	-7.32837	2.36155	-0.84127
N	-4.84400	-1.05711	0.35119
C	-3.85355	-2.00940	0.21250
C	-5.21437	-1.09743	1.68417
C	-3.67133	-2.55101	1.48594
H	-5.99948	-0.47039	2.07690
N	-4.53421	-1.97097	2.39465
C	-2.68300	-3.57095	1.68949
N	-3.15483	-2.33469	-0.90578
C	-2.22096	-3.21099	-0.67429
N	-2.00575	-3.85867	0.49389
N	-1.26438	-3.47101	-1.72308
H	-1.72219	-3.96753	-2.49291
H	-0.97402	-2.52242	-2.07167
H	-1.26904	-4.59227	0.47238
O	-2.40058	-4.17271	2.73452
O	1.45929	-1.44741	2.38780
C	1.58574	-1.73855	1.21731
C	1.18801	-3.13921	0.69481
C	1.21570	-3.44388	-0.77859
H	1.25969	-2.53644	-1.38087
H	2.16764	-3.97391	-0.93393
C	0.15967	-4.45041	-1.33033
O	2.16000	-0.95220	0.28635
O	0.93042	-3.95853	1.55989
H	0.42230	-4.63639	-2.38767
O	-0.18853	-5.46282	-0.62154
N	4.80232	0.74615	0.25788
C	5.81982	0.30246	-0.56747
C	5.36350	0.90953	1.51187
C	6.94192	0.20525	0.25821
H	4.77695	1.27160	2.34413
N	6.63734	0.59389	1.55835
C	8.12848	-0.25174	-0.35052
N	5.75970	0.00995	-1.87486
C	6.94533	-0.40780	-2.33337
N	8.10071	-0.55697	-1.66735
H	6.98055	-0.66519	-3.38849
N	9.29910	-0.35951	0.32005
H	9.27697	-0.35684	1.33099
H	10.04387	-0.88493	-0.12006

AG-4

E: -3363.50313
G: -3363.02005

O	0.07680	3.93438	1.50486
C	1.48181	3.87974	1.22826
H	1.96921	3.60564	2.16791
H	1.85642	4.85809	0.90979
C	1.77277	2.81617	0.17671
H	1.42338	3.14562	-0.80302
O	3.19912	2.59043	0.11855
C	3.39107	1.23045	-0.23973
H	3.24261	1.06041	-1.31140
C	1.11126	1.45696	0.48163
H	0.54345	1.46610	1.41091
C	2.32151	0.50697	0.57573
H	2.64776	0.40791	1.61030
P	-0.97330	4.66356	0.45715
O	-0.54360	6.24972	0.59418
O	-0.67407	4.29283	-0.97300
O	-2.33271	4.41898	1.06295
H	-0.74295	6.58331	1.48742
O	-1.09948	-0.57501	-1.78719
P	-0.97752	0.06969	-0.42887
O	0.27205	1.13326	-0.61877
O	-0.78426	-0.72854	0.83520
O	-2.25876	1.06112	-0.13936
C	-2.88816	1.70962	-1.25770
H	-2.23437	1.68190	-2.13171
H	-3.05579	2.75383	-0.98284
C	-4.23018	1.07357	-1.58401
H	-4.57449	1.49323	-2.53839
O	-4.10805	-0.35752	-1.73921
C	-5.09196	-1.03276	-0.97347
H	-5.45827	-1.87363	-1.56236
C	-5.33570	1.28630	-0.53541
H	-4.90658	1.33709	0.46819
C	-6.17884	0.00487	-0.69749
H	-6.80371	-0.22781	0.16686
O	-6.06062	2.48692	-0.71574
H	-6.47196	2.43865	-1.59758
O	-6.97311	0.08472	-1.87756
H	-7.78068	0.57319	-1.64739
N	-4.51851	-1.57228	0.26949
C	-3.50782	-2.51030	0.34031
C	-4.76868	-1.18655	1.57811
C	-3.23014	-2.65247	1.69886
H	-5.52041	-0.45629	1.83093
N	-4.02844	-1.81860	2.45875
C	-2.16724	-3.51325	2.10730
N	-2.89290	-3.12712	-0.70167
C	-1.88550	-3.87856	-0.32327
N	-1.56432	-4.11733	0.98676
N	-1.14672	-4.60499	-1.26245
H	-0.74093	-4.68187	1.18069
O	-1.75098	-3.75515	3.24730
O	1.95083	-1.71163	2.10192
C	1.80610	-1.77385	0.90101
C	1.31464	-3.07896	0.23368
C	0.79337	-3.02933	-1.17890
H	0.15341	-2.14605	-1.31065
H	1.66186	-2.87764	-1.83326
C	0.03808	-4.26269	-1.60457
O	2.05538	-0.79098	0.02577
O	1.35218	-4.08371	0.91935
H	0.53638	-4.92245	-2.31417
N	4.74379	0.84787	0.07491
C	5.60517	0.13068	-0.73312
C	5.39926	1.03104	1.27876

C	6.75080	-0.06629	0.04209
H	4.93436	1.57199	2.09014
N	6.60478	0.50880	1.29940
C	7.79783	-0.79037	-0.56386
N	5.40242	-0.29939	-1.98752
C	6.46482	-0.97015	-2.44737
N	7.62423	-1.23952	-1.82735
H	6.37953	-1.35381	-3.46047
N	8.97925	-1.02190	0.05374
H	9.03491	-0.89170	1.05516
H	9.59249	-1.71666	-0.35294

AG-TS-4

E: -3496.49592
G: -3495.95579

O	0.27181	4.23716	0.97273
C	1.68431	4.04675	0.82926
H	2.06673	3.83245	1.83100
H	2.16314	4.95778	0.45649
C	1.98590	2.86253	-0.08136
H	1.76150	3.10589	-1.12224
O	3.38723	2.54268	0.04138
C	3.51723	1.15122	-0.20958
H	3.46673	0.92246	-1.27933
C	1.20041	1.58965	0.29195
H	0.61386	1.72435	1.19952
C	2.31747	0.54451	0.52096
H	2.52906	0.44258	1.58450
P	-0.62651	4.92834	-0.22910
O	0.07314	6.41501	-0.36564
O	-0.39081	4.25288	-1.55656
O	-2.01244	5.03672	0.35759
H	-0.05693	6.93106	0.45020
O	-1.32203	-0.28822	-1.81256
P	-1.02408	0.39523	-0.49971
O	0.35697	1.25335	-0.80262
O	-0.84651	-0.35732	0.79136
O	-2.13185	1.57240	-0.19465
C	-3.00685	2.01029	-1.24418
H	-2.55330	1.82400	-2.21935
H	-3.14916	3.08662	-1.12109
C	-4.36420	1.33241	-1.15301
H	-4.92974	1.59841	-2.05693
O	-4.22602	-0.10279	-1.10352
C	-5.08329	-0.66226	-0.11570
H	-5.57310	-1.53428	-0.54978
C	-5.21026	1.70026	0.07370
H	-4.57112	1.83571	0.95014
C	-6.08089	0.43860	0.24393
H	-6.51117	0.33209	1.24209
O	-5.93639	2.90418	-0.07803
H	-6.46046	2.82434	-0.89463
O	-7.10549	0.39678	-0.74456
H	-7.79219	1.02782	-0.47078
N	-4.31334	-1.11796	1.04762
C	-3.34802	-2.10579	1.01916
C	-4.29180	-0.60229	2.33602
C	-2.82466	-2.14706	2.31062
H	-4.94853	0.19386	2.64914
N	-3.42656	-1.20026	3.12084
C	-1.71900	-3.00678	2.58031
N	-2.96017	-2.82292	-0.06327
C	-1.92347	-3.60668	0.17262
N	-1.37177	-3.73538	1.43005
N	-1.38515	-4.42499	-0.79931
H	-0.57030	-4.35170	1.52402
O	-1.08259	-3.15640	3.63476
O	1.82004	-1.70967	2.02038
C	1.74090	-1.74577	0.81178
C	1.31174	-3.03182	0.06907
C	0.52063	-2.84741	-1.19501
H	-0.06309	-1.93294	-1.10278
H	1.24038	-2.66326	-2.00353
C	-0.39648	-4.00120	-1.56739
O	2.00810	-0.74129	-0.03395
O	1.59608	-4.09889	0.58268
H	0.03273	-4.76040	-2.21490
O	-3.26298	-5.19937	-2.77662
H	-2.71698	-5.79127	-3.32036
H	-2.68705	-5.03616	-1.98781
N	-1.20881	-3.01159	-3.16534
H	-0.64537	-2.95409	-4.01269
H	-1.44227	-2.06824	-2.84366
H	-2.05833	-3.55196	-3.34259
N	4.80179	0.70498	0.26267
C	5.76924	0.04577	-0.47273
C	5.30912	0.83762	1.54281
C	6.81519	-0.18241	0.42465
H	4.74404	1.33374	2.31881
N	6.50988	0.32408	1.68312
C	7.94064	-0.86001	-0.08692
N	5.72896	-0.31353	-1.76426
C	6.85118	-0.94543	-2.12693
N	7.93076	-1.23754	-1.38518
H	6.89719	-1.27074	-3.16271
N	9.04182	-1.11299	0.65789
H	8.96874	-1.04314	1.66416
H	9.71151	-1.77764	0.29192

AG-5

E: -3420.55045
G: -3420.01854

O	0.44491	-3.84348	1.30201
C	-0.90886	-3.99109	0.85846
H	-1.53689	-3.85835	1.74353
H	-1.08619	-4.99087	0.44877
C	-1.24888	-2.92046	-0.17609
H	-0.74541	-3.13438	-1.11873
O	-2.68612	-2.92523	-0.39496
C	-3.13015	-1.58120	-0.42444
H	-2.99664	-1.12591	-1.41138
C	-0.86830	-1.50343	0.27575
H	-0.19563	-1.50365	1.13104
C	-2.23537	-0.88855	0.59813
H	-2.55465	-1.12518	1.61386
P	1.71364	-4.33771	0.36525
O	1.77521	-5.95266	0.68816
O	1.36974	-4.24413	-1.10047
O	2.91262	-3.63292	0.94810
H	2.07824	-6.11141	1.60001
O	0.73159	1.26690	-1.77725
P	0.78549	0.40308	-0.53999
O	-0.28652	-0.81862	-0.83461
O	0.54455	0.99061	0.82934
O	2.19633	-0.41453	-0.42596
C	2.71633	-1.05860	-1.60543
H	2.23669	-0.64919	-2.49840
H	2.50844	-2.13021	-1.54835
C	4.20745	-0.82064	-1.68708
H	4.57359	-1.27695	-2.61543
O	4.46945	0.60007	-1.75330
C	5.37980	0.98508	-0.73792
H	6.01759	1.77047	-1.14446
C	5.04439	-1.35332	-0.50780
H	4.45138	-1.38060	0.40814
C	6.15529	-0.28713	-0.39819
H	6.64892	-0.26599	0.57509
O	5.51943	-2.67088	-0.70463

H	6.04721	-2.66264	-1.52284
O	7.11556	-0.45857	-1.43691
H	7.71769	-1.16853	-1.15828
N	4.66651	1.52417	0.43228
C	3.69094	2.50067	0.38282
C	4.68047	1.06874	1.74508
C	3.19248	2.59295	1.68122
H	5.35797	0.29849	2.07712
N	3.82513	1.69004	2.52084
C	2.08786	3.45703	1.93804
N	3.28749	3.18592	-0.71142
C	2.24687	3.96558	-0.47839
N	1.69530	4.13613	0.76771
N	-3.62461	3.75036	-1.81762
H	0.84951	4.69358	0.85603
O	1.47893	3.64837	3.00034
O	-1.88641	0.99812	2.57479
C	-1.92023	1.31515	1.40806
C	-1.62268	2.74907	0.93512
C	-1.66873	3.09991	-0.55314
H	-0.92046	2.46751	-1.06923
H	-1.38058	4.14837	-0.66007
C	-2.97269	2.83529	-1.19489
O	-2.25566	0.52327	0.38071
O	-1.32309	3.57474	1.76876
H	-3.42499	1.84555	-1.17984
N	1.70952	4.70714	-1.48947
H	0.74712	5.00517	-1.36898
H	1.91029	4.34404	-2.41426
H	-3.27760	4.70808	-1.88124
H	-4.51383	3.53749	-2.26888
N	-4.53865	-1.52574	-0.11593
C	-5.41984	-0.57826	-0.59806
C	-5.21605	-2.22510	0.86510
C	-6.60567	-0.78875	0.10956
H	-4.73677	-3.02471	1.41036
N	-6.46039	-1.82923	1.01961
C	-7.67908	0.07245	-0.20157
N	-5.20219	0.35795	-1.53649
C	-6.29080	1.11142	-1.73623
N	-7.48874	1.02872	-1.14030
H	-6.19372	1.89473	-2.48414
N	-8.89498	-0.03717	0.37611
H	-8.99074	-0.59742	1.21233
H	-9.54945	0.72439	0.25038

AG-TS-5

E: -3609.85037
G: -3609.30306

O	0.87389	3.88263	-1.30604
C	-0.47997	4.10620	-0.89580
H	-1.08862	4.03656	-1.80138
H	-0.60419	5.10551	-0.46602
C	-0.92514	3.03442	0.09861
H	-0.46203	3.20112	1.07120
O	-2.37099	3.11031	0.24246
C	-2.88550	1.78919	0.19471
H	-2.83042	1.29236	1.16812
C	-0.59554	1.61010	-0.37062
H	0.13427	1.59670	-1.17754
C	-1.96899	1.09287	-0.80606
H	-2.20653	1.39834	-1.82593
P	2.14461	4.28894	-0.33217
O	2.28185	5.90894	-0.60472
O	1.77250	4.16665	1.12428
O	3.32142	3.54911	-0.91747
H	2.57934	6.08122	-1.51604
O	0.78037	-1.27751	1.69925
P	0.90496	-0.40051	0.47909
O	-0.13622	0.85454	0.75057
O	0.70854	-0.94851	-0.91302
O	2.34609	0.37957	0.43690
C	2.86864	0.92053	1.66408
H	2.33720	0.49247	2.51799
H	2.72986	2.00513	1.66440
C	4.33918	0.58387	1.77116
H	4.70257	0.94784	2.74061
O	4.51252	-0.85184	1.73755
C	5.44648	-1.21650	0.73620
H	6.02155	-2.06378	1.11117
C	5.24518	1.14415	0.65919
H	4.68938	1.25712	-0.27346
C	6.30361	0.02977	0.52065
H	6.83801	0.04756	-0.43074
O	5.77702	2.42144	0.95368
H	6.26271	2.34226	1.79376
O	7.22526	0.07674	1.60592
H	7.85844	0.78818	1.41267
N	4.76004	-1.63050	-0.50046
C	3.72001	-2.53743	-0.56334
C	4.88230	-1.10519	-1.78179
C	3.29473	-2.52326	-1.89054
H	5.62797	-0.36744	-2.03159
N	4.03395	-1.62079	-2.63864
C	2.14603	-3.28477	-2.25595
N	3.20212	-3.24698	0.46501
C	2.12426	-3.93304	0.12915
N	1.64676	-4.00924	-1.15606
H	0.75846	-4.47595	-1.32128
O	1.58215	-3.36183	-3.35721
N	1.46965	-4.67325	1.07051
H	0.49740	-4.89022	0.87617
H	1.62827	-4.35247	2.01901
N	-4.27536	1.80262	-0.19203
C	-5.22837	0.92692	0.29414
C	-4.83746	2.40679	-1.30236
C	-6.33306	1.07976	-0.54587
H	-4.28666	3.13645	-1.87818
N	-6.07207	2.02053	-1.53702
C	-7.44542	0.25817	-0.26492
N	-5.12756	0.09462	1.34011
C	-6.23520	-0.63780	1.49538
N	-7.36451	-0.60952	0.77041
H	-6.21958	-1.34639	2.31939
N	-8.59780	0.32224	-0.97039
H	-8.59064	0.78701	-1.86866
H	-9.26294	-0.43111	-0.84971
N	-3.99420	-3.59455	1.05528
O	-1.57923	-0.70043	-2.85235
C	-1.73329	-1.08238	-1.71396
C	-1.58413	-2.56221	-1.30605
C	-1.80943	-2.95581	0.14048
H	-1.05283	-2.43473	0.73981
H	-1.63298	-4.03042	0.23271
C	-3.20287	-2.60941	0.62662
O	-2.08518	-0.32220	-0.66767
O	-1.25527	-3.35308	-2.16698
H	-3.72828	-1.78227	0.15965
H	-3.58462	-4.42049	1.48003
H	-4.94445	-3.36874	1.33001
C	-2.88504	-1.31746	2.92194
H	-2.86910	-1.76185	1.77689
O	-3.69144	-1.93978	3.61294
O	-2.08056	-0.39571	3.06513

AG-6

E: -3421.31050
G: -3420.76870

O	-0.58981	3.64017	1.35539
C	0.76074	3.85033	0.92700
H	1.39389	3.63969	1.79334
H	0.92387	4.88800	0.61839
C	1.11396	2.89112	-0.20847
H	0.59564	3.17904	-1.12359
O	2.54855	2.95894	-0.43444
C	3.05971	1.63909	-0.50951
H	3.00508	1.23628	-1.52563
C	0.77851	1.43163	0.12168
H	0.09416	1.34881	0.96387
C	2.15804	0.84334	0.43063
H	2.43922	1.02097	1.46915
P	-1.85790	4.27902	0.51236
O	-1.86789	5.84053	1.04065
O	-1.54065	4.36286	-0.95973
O	-3.06783	3.53809	1.02358
H	-2.12841	5.88492	1.97817
O	-0.87778	-1.17482	-2.11236
P	-0.84927	-0.42552	-0.80413
O	0.23624	0.80256	-1.04072
O	-0.57143	-1.11256	0.50807
O	-2.23519	0.42018	-0.55700
C	-2.86310	1.06776	-1.67564
H	-2.42018	0.71613	-2.61078
H	-2.71213	2.14778	-1.59677
C	-4.34362	0.75259	-1.67148
H	-4.78581	1.17821	-2.58140
O	-4.53511	-0.68051	-1.70594
C	-5.40052	-1.09153	-0.66072
H	-6.01898	-1.90445	-1.04280
C	-5.14167	1.25321	-0.45404
H	-4.51033	1.28621	0.43605
C	-6.21341	0.15308	-0.30689
H	-6.67833	0.12214	0.68029
O	-5.65845	2.55994	-0.61465
H	-6.19002	2.56169	-1.43021
O	-7.20858	0.27909	-1.31838
H	-7.80005	1.00287	-1.05241
N	-4.63593	-1.59196	0.49492
C	-3.61803	-2.52305	0.43175
C	-4.66667	-1.15383	1.81370
C	-3.11187	-2.60974	1.72745
H	-5.37695	-0.41884	2.15678
N	-3.78056	-1.74436	2.57913
C	-1.96482	-3.42270	1.96708
N	-3.18303	-3.16998	-0.67254
C	-2.10500	-3.90127	-0.45152
N	-1.55950	-4.08893	0.79400
H	-0.65671	-4.55444	0.86304
O	-1.33358	-3.58486	3.02188
N	-1.52246	-4.57844	-1.48404
H	-0.54736	-4.83106	-1.36021
H	-1.71732	-4.17118	-2.39181
N	4.45142	1.62944	-0.12220
C	5.41556	0.79029	-0.64870
C	4.99665	2.17256	1.02720
C	6.50942	0.90297	0.21302
H	4.43324	2.86304	1.63794
N	6.23107	1.78214	1.25380
C	7.63329	0.11033	-0.09836
N	5.33725	0.02302	-1.74626
C	6.45667	-0.68668	-1.92662
N	7.57569	-0.69325	-1.18485
H	6.46613	-1.34493	-2.79132
N	8.77487	0.14297	0.62745
H	8.74610	0.54945	1.55315
H	9.44535	-0.60015	0.47611
N	4.28768	-3.11339	-0.22124
O	1.94093	-1.13754	2.29308
C	1.97837	-1.41380	1.11271
C	1.73164	-2.84493	0.59631
C	1.92341	-3.17962	-0.86067
H	1.18036	-2.63824	-1.45764
H	1.72214	-4.25155	-0.95706
C	3.34850	-2.84197	-1.32257
O	2.23495	-0.54639	0.12212
O	1.36096	-3.65731	1.42820
H	3.42368	-1.78295	-1.56341
H	4.36796	-4.12255	-0.09507
H	5.21547	-2.78834	-0.49103
H	3.56509	-3.40188	-2.24379

AG-TS-6

E: -3421.26338
G: -3420.72285

O	0.37344	4.26839	-1.29651
C	-1.05439	4.17164	-1.36956
H	-1.29436	3.89604	-2.40025
H	-1.53297	5.12859	-1.13937
C	-1.53190	3.07353	-0.43030
H	-1.47219	3.40628	0.61027
O	-2.89173	2.72501	-0.74360
C	-3.08730	1.43515	-0.17694
H	-3.17974	1.49147	0.91244
C	-0.72089	1.77116	-0.55715
H	-0.15432	1.73652	-1.48727
C	-1.81396	0.67335	-0.56688
H	-1.90800	0.25690	-1.56674
P	1.09037	5.12874	-0.08127
O	1.05900	6.65815	-0.69808
O	0.20914	5.17258	1.14004
O	2.50623	4.61053	-0.02136
H	1.62956	6.72123	-1.48476
O	1.19069	-0.10430	1.96726
P	1.30723	0.51566	0.58349
O	0.15887	1.70066	0.55513
O	1.19660	-0.30848	-0.67016
O	2.66903	1.40938	0.47099
C	3.64482	1.51483	1.51394
H	3.20695	1.24010	2.47644
H	3.95335	2.56335	1.54952
C	4.87051	0.66779	1.23607
H	5.62530	0.92520	1.99341
O	4.55255	-0.73369	1.33963
C	5.42003	-1.50177	0.52148
H	6.04546	-2.13910	1.15276
C	5.49177	0.80192	-0.15522
H	4.70481	0.81761	-0.91263
C	6.29515	-0.50858	-0.26629
H	6.46091	-0.80318	-1.30343
O	6.26488	1.97164	-0.33538
H	6.99479	1.92770	0.30830
O	7.53405	-0.39915	0.43007
H	8.15592	0.06238	-0.15692
N	4.62262	-2.37474	-0.36479
C	3.33619	-2.84271	-0.18408
C	5.06365	-2.95255	-1.54287
C	3.06014	-3.64228	-1.29412
H	6.06504	-2.79102	-1.91217
N	4.16188	-3.71043	-2.12895
C	1.74945	-4.21858	-1.44416
N	2.48061	-2.56449	0.82621
C	1.29404	-3.04188	0.58408
N	0.88496	-3.83982	-0.41055
H	-0.31665	-4.06836	-0.24150
O	1.36050	-4.96125	-2.36376
N	0.23531	-2.60852	1.50646
H	0.25472	-3.19655	2.34967

H	0.49321	-1.61636	1.79547
N	-4.16163	-2.68792	0.73452
O	-1.46664	-1.89677	-1.33016
C	-1.42767	-1.63366	-0.14858
C	-1.26772	-2.73784	0.94682
C	-2.14332	-2.49944	2.19005
H	-1.66950	-1.76122	2.84622
H	-2.14386	-3.46062	2.71630
C	-3.57681	-2.03685	1.91294
O	-1.56869	-0.39205	0.36205
O	-1.40042	-3.96289	0.40787
H	-3.57783	-0.96248	1.72869
H	-4.17390	-3.69622	0.88513
H	-5.13549	-2.39690	0.66019
H	-4.15442	-2.19374	2.83750
N	-4.30581	0.85701	-0.67838
C	-5.39483	0.46421	0.07907
C	-4.56500	0.44011	-1.97314
C	-6.25313	-0.17480	-0.81857
H	-3.86635	0.63307	-2.77442
N	-5.71939	-0.17160	-2.10277
C	-7.43796	-0.71350	-0.27548
N	-5.59650	0.63645	1.39349
C	-6.74334	0.07206	1.78819
N	-7.65655	-0.58133	1.05287
H	-6.97192	0.15548	2.84735
N	-8.37576	-1.32696	-1.03239
H	-8.12691	-1.62271	-1.96697
H	-9.08893	-1.86299	-0.55458

AG-7

E: -3344.84410
G: -3344.32607

O	-0.38129	4.30776	1.53697
C	1.03367	4.23534	1.34066
H	1.45971	3.89979	2.29005
H	1.45101	5.21449	1.08838
C	1.35129	3.22047	0.25199
H	1.02183	3.59707	-0.72037
O	2.77938	3.00694	0.21415
C	2.97323	1.68812	-0.26177
H	2.82666	1.61660	-1.34472
C	0.68776	1.84459	0.47917
H	0.16188	1.79235	1.43129
C	1.89946	0.88675	0.47315
H	2.22019	0.67188	1.49180
P	-1.36119	5.04711	0.42321
O	-1.91181	6.32549	1.30559
O	-0.52910	5.63366	-0.68833
O	-2.49807	4.09171	0.14975
H	-2.58166	6.03353	1.94914
O	-0.98796	-0.46869	-1.79289
P	-1.22986	0.29797	-0.51878
O	-0.21685	1.61815	-0.59359
O	-1.10566	-0.30303	0.85682
O	-2.70630	1.02863	-0.55943
C	-3.43672	1.10314	-1.78757
H	-2.75441	1.04691	-2.63840
H	-3.94285	2.07353	-1.80899
C	-4.49111	0.01380	-1.90026
H	-4.90075	0.06532	-2.91893
O	-3.91144	-1.29336	-1.70090
C	-4.78584	-2.12593	-0.94959
H	-4.85398	-3.09070	-1.45394
C	-5.66299	0.07525	-0.91424
H	-5.31197	0.35221	0.08400
C	-6.12929	-1.39488	-0.91624
H	-6.75970	-1.65573	-0.06526
O	-6.65881	1.01473	-1.26633
H	-6.94306	0.80836	-2.17444
O	-6.79448	-1.71467	-2.13458
H	-7.69894	-1.36503	-2.06866
N	-4.24640	-2.35577	0.40369
C	-2.96403	-2.77904	0.69270
C	-4.84735	-2.11500	1.63061
C	-2.87324	-2.77292	2.08362
H	-5.86543	-1.77036	1.71350
N	-4.06348	-2.36216	2.65511
C	-1.61542	-3.05432	2.70000
N	-1.98736	-3.07886	-0.19407
C	-0.81310	-3.22122	0.37301
N	-0.62538	-3.29609	1.72744
H	0.32907	-3.32976	2.07663
O	-1.32759	-3.07965	3.90454
N	0.29285	-3.41588	-0.45408
O	1.65550	-1.57094	1.67737
C	1.53636	-1.46899	0.47312
C	1.32328	-2.65604	-0.45748
C	2.46525	-2.91048	-1.39873
H	2.52381	-2.07380	-2.10572
H	2.24770	-3.82234	-1.96274
C	3.80422	-3.03356	-0.66280
H	4.58875	-3.18517	-1.41737
H	4.04002	-2.09376	-0.15348
O	1.66793	-0.33825	-0.23272
N	4.31891	1.25530	0.02180
C	5.08277	0.43503	-0.78860
C	4.95985	1.29557	1.24774
C	6.16854	0.05066	0.00225
H	4.55365	1.86755	2.06935
N	6.07798	0.60615	1.27369
C	7.10293	-0.81827	-0.59861
N	4.84739	0.06837	-2.05777
C	5.79662	-0.75988	-2.50847
N	6.88861	-1.21406	-1.87375
H	5.67207	-1.11094	-3.52928
N	8.21762	-1.24392	0.03743
H	8.27801	-1.13021	1.04040
H	8.72815	-2.01626	-0.37114
N	3.72935	-4.09466	0.34972
H	3.60325	-4.98496	-0.13311
H	4.63763	-4.16265	0.80784

AG-TS-7

E: -3477.83471
G: -3477.25471

O	0.74423	4.53285	-1.04403
C	-0.68655	4.59158	-1.01245
H	-1.02203	4.48661	-2.04804
H	-1.03808	5.55212	-0.62321
C	-1.23175	3.42971	-0.19284
H	-1.10882	3.61692	0.87792
O	-2.63183	3.25640	-0.48846
C	-2.94728	1.95318	-0.04122
H	-3.04967	1.91258	1.04856
C	-0.56603	2.08208	-0.52193
H	-0.14786	2.08819	-1.52948
C	-1.75758	1.08604	-0.48566
H	-1.93679	0.67028	-1.47438
P	1.63143	5.09852	0.23109
O	1.75636	6.70220	-0.13797
O	0.83091	5.04864	1.50655
O	2.97518	4.42386	0.09990
H	2.31807	6.83132	-0.92292
O	1.47629	-0.10223	1.62182
P	1.41406	0.50085	0.24267
O	0.45035	1.84307	0.43334
O	0.95715	-0.21016	-1.00347

O	2.88022	1.15768	-0.14056
C	3.93507	1.16457	0.82654
H	3.52220	1.25569	1.83346
H	4.55168	2.04518	0.62334
C	4.83364	-0.06151	0.75426
H	5.53196	0.01584	1.60064
O	4.07937	-1.28382	0.88575
C	4.63719	-2.31850	0.08098
H	4.76716	-3.20273	0.70681
C	5.66274	-0.26337	-0.51762
H	5.05731	-0.06667	-1.40729
C	5.97602	-1.77197	-0.42278
H	6.31578	-2.20737	-1.36305
O	6.79669	0.57682	-0.60893
H	7.30923	0.46825	0.21150
O	6.92793	-2.03091	0.60524
H	7.80033	-1.77814	0.25987
N	3.72395	-2.66541	-1.02041
C	2.37116	-2.94094	-0.89333
C	3.97631	-2.63191	-2.38632
C	1.89885	-3.05945	-2.20118
H	4.95978	-2.44040	-2.78367
N	2.91741	-2.87406	-3.12111
C	0.49799	-3.19892	-2.41283
N	1.66068	-2.99061	0.25058
C	0.34754	-2.99345	0.06266
N	-0.19994	-3.18449	-1.19266
H	-1.20752	-3.14413	-1.30359
O	-0.12324	-3.30138	-3.48407
N	-0.45785	-2.85825	1.16008
O	-2.55010	-1.45191	-0.97326
C	-1.98786	-1.21110	0.07851
C	-1.63982	-2.24621	1.16591
C	-2.19470	-1.92644	2.54914
H	-3.26036	-1.69801	2.47785
H	-2.07988	-2.84123	3.13769
C	-1.45115	-0.78199	3.26954
H	-0.46934	-0.62296	2.82288
O	-1.56950	0.00883	0.44806
N	-3.10055	-3.52617	0.68705
H	-2.93504	-4.01431	-0.19219
H	-4.00983	-3.06604	0.63459
H	-3.11568	-4.21010	1.44333
O	0.56568	-3.19497	3.86689
H	-0.03241	-2.53501	4.29983
H	0.33110	-3.10252	2.91639
H	-2.01713	0.15078	3.15429
N	-1.23405	-1.14453	4.68416
H	-0.80596	-0.35045	5.15976
H	-2.13844	-1.28639	5.13481
N	-4.20295	1.52460	-0.61701
C	-5.00781	0.54132	-0.06757
C	-4.55420	1.60328	-1.95386
C	-5.83774	0.12067	-1.10855
H	-4.04916	2.28679	-2.62072
N	-5.54609	0.80674	-2.28221
C	-6.74405	-0.91655	-0.80561
N	-4.99312	0.06403	1.18640
C	-5.88276	-0.92292	1.34387
N	-6.73914	-1.43301	0.44479
H	-5.92343	-1.37087	2.33304
N	-7.63081	-1.39726	-1.70572
H	-7.49493	-1.17855	-2.68380
H	-8.09616	-2.26931	-1.48871

AG-8

E: -3401.90383
G: -3401.33769

O	0.11304	4.66305	0.63936
C	1.44504	4.45090	0.16046
H	2.08510	4.38929	1.04493
H	1.77854	5.29234	-0.45553
C	1.54039	3.14662	-0.62172
H	0.98386	3.21524	-1.55715
O	2.94263	2.91841	-0.91856
C	3.17469	1.52828	-0.84111
H	2.87724	1.00632	-1.75681
C	1.02583	1.92051	0.16310
H	0.59927	2.20087	1.12576
C	2.30522	1.08362	0.33063
H	2.79991	1.30799	1.27540
P	-1.06777	5.21513	-0.37720
O	-0.50119	6.70768	-0.78525
O	-1.09203	4.42781	-1.66268
O	-2.29000	5.32423	0.50003
H	-0.49484	7.29624	-0.00890
O	-1.53250	-0.65627	-0.98613
P	-1.07878	0.28581	0.09340
O	0.06315	1.23524	-0.63384
O	-0.56573	-0.21422	1.43080
O	-2.23054	1.36719	0.51584
C	-3.10746	1.98102	-0.44494
H	-2.70534	1.89327	-1.45742
H	-3.16305	3.03952	-0.18228
C	-4.50387	1.39175	-0.37565
H	-5.16633	2.02670	-0.98002
O	-4.52147	0.06140	-0.92746
C	-5.24942	-0.83741	-0.10022
H	-5.97082	-1.37343	-0.71687
C	-5.07802	1.26899	1.04674
H	-4.27056	1.10736	1.76263
C	-5.96171	0.00671	0.95832
H	-6.06253	-0.51106	1.91665
O	-5.76496	2.42553	1.48604
H	-6.46884	2.60503	0.83779
O	-7.24192	0.32761	0.42113
H	-7.72703	0.80980	1.11216
N	-4.34565	-1.84658	0.44673
C	-3.84727	-2.89210	-0.30152
C	-3.56561	-1.79237	1.59585
C	-2.81425	-3.43825	0.46064
H	-3.73304	-1.05386	2.36517
N	-2.66472	-2.74292	1.65345
C	-2.04222	-4.48890	-0.09955
N	-4.28144	-3.28211	-1.51554
C	-3.59546	-4.30104	-2.00989
N	-2.53603	-4.88299	-1.34983
H	-2.02076	-5.62781	-1.81291
O	-1.01840	-5.03858	0.36521
N	-3.88339	-4.78020	-3.24357
O	2.24756	-0.66964	2.46233
C	1.99240	-1.03384	1.33835
C	1.58358	-2.45279	0.99950
C	2.57857	-3.35908	0.36447
H	2.67562	-3.01720	-0.67608
H	2.18249	-4.37926	0.34899
C	3.94737	-3.29242	1.04477
H	4.63803	-3.92164	0.46659
H	4.33732	-2.27024	0.99428
O	2.07320	-0.32364	0.21484
N	4.58115	1.27006	-0.62442
C	5.21013	0.07423	-0.92690
C	5.40632	1.92281	0.27524
C	6.41220	0.10371	-0.21562
H	5.12843	2.88113	0.68873
N	6.52184	1.27500	0.52540
C	7.24493	-1.02703	-0.34800
N	4.77548	-0.91585	-1.72231
C	5.64396	-1.93301	-1.75287

N	6.82826	-2.04728	-1.13114
H	5.35768	-2.78152	-2.36838
N	8.45436	-1.11653	0.25145
H	8.66795	-0.47608	1.00462
H	8.89257	-2.02824	0.28714
N	3.81267	-3.67149	2.45549
H	3.56727	-4.66113	2.49757
H	4.72851	-3.59903	2.89720
H	-3.57594	-5.71488	-3.48506
H	-4.77658	-4.49813	-3.62802
N	0.36837	-2.77016	1.25065
H	-0.01598	-3.71232	1.03314
H	-0.28515	-2.01373	1.53526

AG-TS-8

E: -3591.19767
G: -3590.61656

O	0.57148	4.88271	1.27660
C	1.71696	4.47146	0.52497
H	2.44040	4.10323	1.25740
H	2.15623	5.32515	-0.00084
C	1.40909	3.36288	-0.48131
H	0.75509	3.72142	-1.27606
O	2.67672	2.96469	-1.07546
C	2.88906	1.58258	-0.84772
H	2.54766	0.97835	-1.69301
C	0.80336	2.11110	0.16184
H	0.26380	2.33857	1.08017
C	2.06201	1.26989	0.39687
H	2.58222	1.59142	1.30026
P	-0.75323	5.54750	0.54514
O	-0.07215	6.62315	-0.49892
O	-1.45483	4.53232	-0.32377
O	-1.48572	6.25123	1.65907
H	0.37456	7.34072	-0.01432
O	1.55551	-0.09369	2.68617
C	1.49427	-0.66667	1.61597
C	1.13983	-2.13757	1.49165
C	0.62742	-2.76165	0.20659
H	-0.39729	-2.40472	0.06866
H	0.57494	-3.83856	0.40039
C	1.41399	-2.53928	-1.08420
H	1.25671	-1.52370	-1.44627
O	1.82347	-0.13441	0.43678
N	4.29846	1.30978	-0.68192
C	4.95205	0.18785	-1.15755
C	5.15923	1.90499	0.22279
C	6.19527	0.19113	-0.52047
H	4.87848	2.80666	0.74817
N	6.31018	1.28075	0.33591
C	7.05453	-0.88479	-0.82345
N	4.50687	-0.72076	-2.03774
C	5.40634	-1.69097	-2.23362
N	6.62817	-1.82609	-1.69490
H	5.11340	-2.47514	-2.92677
N	8.30014	-0.99367	-0.30401
H	8.52963	-0.43870	0.50987
H	8.76233	-1.89023	-0.38860
N	0.62985	-2.63895	2.62650
H	0.00268	-3.44427	2.59464
H	0.68854	-2.08585	3.47333
C	3.68332	-2.95981	1.66010
H	2.50438	-2.51533	1.40309
O	3.58597	-3.80077	2.54029
O	4.51404	-2.40003	0.95821
N	0.89794	-3.46663	-2.10363
H	1.38919	-3.27664	-2.97658
H	1.17809	-4.41218	-1.84043
H	2.49026	-2.65496	-0.89597
O	-1.43999	-0.52575	-1.39120
P	-1.25193	0.45755	-0.26617
O	-0.03971	1.46226	-0.78955
O	-0.99233	0.02851	1.15473
O	-2.52812	1.48373	-0.14919
C	-3.24609	1.90960	-1.31530
H	-2.68298	1.68645	-2.22564
H	-3.37590	2.99247	-1.24096
C	-4.61156	1.25119	-1.37293
H	-5.16157	1.67503	-2.22375
O	-4.47233	-0.16869	-1.58584
C	-5.25486	-0.90967	-0.66206
H	-5.78310	-1.68752	-1.21376
C	-5.45317	1.40812	-0.09342
H	-4.80006	1.49520	0.77714
C	-6.23500	0.08004	-0.03201
H	-6.53518	-0.19758	0.98172
O	-6.27135	2.56264	-0.08903
H	-6.84397	2.50784	-0.87446
O	-7.36762	0.12712	-0.89619
H	-8.03963	0.67520	-0.45668
N	-4.40671	-1.59776	0.31234
C	-3.67135	-2.72566	0.02067
C	-4.11139	-1.27838	1.63329
C	-2.99545	-3.04668	1.20020
H	-4.54636	-0.42170	2.12486
N	-3.29286	-2.13097	2.20064
C	-2.15840	-4.19578	1.20996
N	-3.65197	-3.37795	-1.15967
C	-2.87376	-4.44841	-1.14611
N	-2.18254	-4.85678	-0.02982
H	-1.56430	-5.66044	-0.10269
O	-1.44497	-4.64495	2.12912
N	-2.69460	-5.17574	-2.27550
H	-2.35657	-6.12676	-2.19097
H	-3.36356	-5.00953	-3.01699

AG-Dab

E: -3402.65584
G: -3402.07781

O	-0.03573	4.89313	0.79995
C	1.32635	4.68838	0.40563
H	1.91666	4.63782	1.32476
H	1.69679	5.51582	-0.20712
C	1.42396	3.37056	-0.34701
H	0.87300	3.44031	-1.28800
O	2.81541	3.09425	-0.63162
C	2.96091	1.68572	-0.62116
H	2.60250	1.22913	-1.54968
C	0.87004	2.16970	0.44838
H	0.49841	2.45872	1.43097
C	2.09846	1.24783	0.55998
H	2.62908	1.43271	1.49408
P	-1.11594	5.55393	-0.26190
O	-0.92849	7.16649	0.02559
O	-0.66313	5.34618	-1.68385
O	-2.46985	5.09196	0.22061
H	-1.28333	7.40088	0.90159
O	-1.34115	-0.63552	-0.59396
P	-1.20836	0.49559	0.39348
O	-0.17078	1.57581	-0.32029
O	-0.83861	0.27210	1.83652
O	-2.58959	1.38427	0.46638
C	-3.30976	1.78009	-0.70906
H	-2.69056	1.67787	-1.60532
H	-3.56253	2.83642	-0.58433
C	-4.59336	0.98499	-0.87239
H	-5.20005	1.47602	-1.64562
O	-4.29978	-0.35634	-1.30976

C	-5.04059	-1.32010	-0.57202
H	-5.52784	-1.99476	-1.27609
C	-5.42969	0.83872	0.41137
H	-4.77577	0.84069	1.28494
C	-6.07880	-0.55166	0.24808
H	-6.30290	-1.03333	1.20378
O	-6.35629	1.88989	0.60778
H	-6.94733	1.89271	-0.16612
O	-7.25061	-0.46591	-0.55958
H	-7.95577	-0.09572	-0.00215
N	-4.14031	-2.14477	0.22844
C	-3.35442	-3.14067	-0.30945
C	-3.68309	-1.95653	1.52697
C	-2.49959	-3.54124	0.71795
H	-4.11758	-1.21689	2.18232
N	-2.72889	-2.79106	1.86404
C	-1.55666	-4.57573	0.45270
N	-3.42698	-3.60832	-1.57336
C	-2.54338	-4.56085	-1.82185
N	-1.66035	-5.02698	-0.87785
H	-1.00216	-5.75516	-1.14222
O	-0.71189	-5.09002	1.20617
N	-2.43875	-5.08620	-3.06926
O	2.25478	-0.49271	2.62472
C	1.82169	-0.89556	1.56305
C	1.30337	-2.28975	1.27219
C	2.18037	-2.96294	0.18708
H	2.03460	-2.44180	-0.76529
H	1.79689	-3.98312	0.05475
C	3.67989	-3.00521	0.48379
H	4.19349	-3.40149	-0.40548
H	4.05951	-1.98666	0.62785
O	1.77645	-0.13075	0.44075
N	4.35411	1.33530	-0.47818
C	4.96013	0.22734	-1.04248
C	5.22235	1.79765	0.49475
C	6.18803	0.10886	-0.38561
H	4.97411	2.65943	1.09719
N	6.33834	1.10801	0.56944
C	7.00371	-0.97346	-0.77480
N	4.49078	-0.57501	-2.01045
C	5.35095	-1.56229	-2.28380
N	6.55332	-1.80686	-1.73931
H	5.03862	-2.26136	-3.05481
N	8.23053	-1.19083	-0.24632
H	8.47394	-0.72255	0.61643
H	8.65802	-2.09276	-0.41378
N	3.98513	-3.77342	1.70563
H	3.72206	-4.74549	1.53898
H	4.99709	-3.78431	1.83197
H	-2.00739	-5.99757	-3.16961
H	-3.22680	-4.91045	-3.68045
N	1.15608	-3.03415	2.52303
H	0.58205	-3.84724	2.29797
H	2.08586	-3.38760	2.75638
H	0.30970	-2.14888	0.83822

CC-1

E: -3901.31911
G: -3900.81003

O	2.58035	0.16271	-2.23022
C	1.71702	-0.32955	-3.27811
H	2.38139	-0.64779	-4.08353
H	1.06242	0.46222	-3.65411
C	0.86055	-1.50678	-2.84484
H	0.29017	-1.81391	-3.72605
O	1.67413	-2.64675	-2.46204
C	1.62273	-2.86740	-1.07889
H	1.18867	-3.85046	-0.87008
C	-0.10032	-1.26001	-1.66739
H	-0.37310	-0.21348	-1.55515
C	0.73827	-1.74714	-0.49657
H	1.36265	-0.92334	-0.15688
O	0.01961	-2.27523	0.62205
P	2.23016	1.52256	-1.43594
O	2.16140	2.67810	-2.56816
O	0.94582	1.47447	-0.69213
O	3.56473	1.72416	-0.63411
C	2.87667	-3.11726	0.93707
C	3.71915	-2.54639	1.83135
C	4.59455	-1.53278	1.31930
N	2.95603	-2.84693	-0.40788
C	4.07046	-2.08936	-0.88878
N	4.78850	-1.36934	0.01253
C	-0.05069	-1.50041	1.72294
C	-0.24320	-2.34725	2.98834
C	-0.48746	-1.59070	4.27435
H	0.44469	-1.06681	4.52489
H	-0.65904	-2.34609	5.04664
C	-1.65681	-0.61063	4.19222
H	-2.02078	-0.34083	5.18767
H	-1.34729	0.31858	3.70316
C	-2.79847	-1.19848	3.36034
O	-3.95071	-0.70926	3.47583
O	0.12805	-0.29957	1.72748
O	0.07808	-3.53281	2.96056
O	-2.47449	-2.14720	2.56744
O	4.36723	-2.13039	-2.09098
N	5.27324	-0.72514	2.17247
H	5.45132	0.22576	1.81292
H	4.93225	-0.73341	3.12732
H	3.64507	-2.75773	2.89065
H	2.07100	-3.77702	1.23905
P	3.69531	2.27457	1.06621
O	5.18823	2.00961	1.28085
O	3.31232	3.75418	0.96918
O	2.72885	1.37557	1.82039
O	-1.27225	-2.06519	-1.79438
P	-2.44110	-1.50768	-2.83220
O	-2.45546	-2.37541	-4.06817
O	-2.30425	-0.01137	-2.98117
O	-3.78310	-1.91289	-1.98725
C	-3.86874	-1.93243	-0.55488
H	-2.99428	-2.43044	-0.13082
H	-4.75358	-2.53510	-0.33473
C	-4.05726	-0.59840	0.12974
H	-4.41221	-0.83105	1.14359
O	-2.82615	0.14240	0.23812
C	-3.20963	1.40035	0.80666
H	-3.26067	1.29515	1.89325
C	-5.00738	0.43019	-0.49212
H	-4.76745	0.56705	-1.54714
C	-4.64531	1.70074	0.31728
H	-4.71346	2.61418	-0.26844
O	-6.37591	0.08018	-0.40048
H	-6.61352	0.21684	0.53603
O	-5.47573	1.79397	1.48164
H	-6.26690	2.29772	1.22873
C	-1.27381	2.66376	1.52083
C	-2.03791	2.96197	-0.75531
C	-0.22204	3.48082	1.29589
H	-1.44892	2.18139	2.47394
C	-0.09796	4.02581	-0.02243
H	0.52515	3.66465	2.05582
N	-2.19179	2.39642	0.53605
N	-1.02142	3.83641	-0.96585
N	0.98131	4.79228	-0.30962
H	1.12560	4.95441	-1.30049
H	1.83912	4.54852	0.20822

O	-2.84412	2.64845	-1.64899
H	2.99761	2.75966	-3.06505

CC-TS-1

E: -3901.28890
G: -3900.78422

O	3.06614	-2.27574	-1.30610
C	2.29897	-3.48570	-1.21918
H	3.00070	-4.29951	-1.01492
H	1.78951	-3.69994	-2.16320
C	1.25480	-3.44813	-0.11398
H	0.68992	-4.38302	-0.16110
O	1.86752	-3.38923	1.20376
C	1.67650	-2.12639	1.78685
H	1.00650	-2.21526	2.64864
C	0.29585	-2.25707	-0.17733
H	0.17031	-1.89607	-1.19690
C	1.02462	-1.25012	0.70257
H	1.78204	-0.71515	0.13417
O	0.17994	-0.29663	1.36874
P	2.98693	-1.31795	-2.64361
O	3.19848	-2.39963	-3.86725
O	1.59882	-0.76086	-2.82914
O	4.19702	-0.41291	-2.54258
C	2.66280	-0.38605	3.08573
C	3.57713	0.59887	3.25146
C	4.78412	0.46919	2.49740
N	2.91732	-1.48978	2.31160
C	4.22662	-1.68248	1.77847
N	5.12030	-0.66150	1.89100
C	-0.02685	0.87949	0.76898
C	-0.66669	1.88291	1.74507
C	-0.19411	3.29628	1.60195
H	-0.44718	3.65186	0.59519
H	-0.73258	3.90213	2.33372
C	1.34310	3.39423	1.76546
H	1.57383	4.13486	2.54000
H	1.77877	2.45466	2.12130
C	2.10616	3.80748	0.49193
O	1.47288	4.32755	-0.45817
O	0.25006	1.16116	-0.37727
O	-1.49286	1.48868	2.55229
O	3.36736	3.60202	0.52194
O	4.53592	-2.76100	1.25152
N	5.64282	1.52171	2.42668
H	6.29400	1.48559	1.64931
H	5.22396	2.43524	2.56406
H	3.36561	1.47410	3.85182
H	1.67718	-0.34026	3.53355
P	3.85828	1.71169	-1.08270
O	5.34870	1.92683	-0.97216
O	3.09451	2.34262	-2.21555
O	3.16165	0.82379	-0.08936
H	4.09442	-2.78082	-3.83608
O	-2.27133	-3.13294	-1.74072
P	-2.32899	-2.34308	-0.45621
O	-0.95480	-2.59902	0.42626
O	-3.45584	-2.53063	0.52544
O	-2.16734	-0.73958	-0.78091
C	-2.09284	-0.24812	-2.12725
H	-2.32848	-1.05012	-2.82854
H	-1.07599	0.10526	-2.32248
C	-3.07090	0.89629	-2.29254
H	-3.01728	1.25150	-3.33031
O	-4.40761	0.42057	-2.03775
C	-5.08049	1.25043	-1.09396
H	-6.08666	1.42993	-1.46495
C	-2.84869	2.08256	-1.33770
H	-2.37471	1.73516	-0.42264
C	-4.28266	2.55248	-1.03171
H	-4.37146	3.07364	-0.07373
O	-1.99476	3.08873	-1.84692
H	-2.29577	3.31463	-2.74413
O	-4.78252	3.36691	-2.08895
H	-4.24317	4.17618	-2.09914
C	-4.39346	0.73604	1.25296
C	-6.23626	-0.45034	0.24207
C	-4.53867	0.02300	2.39541
H	-3.61866	1.48019	1.15604
C	-5.60058	-0.93396	2.43006
H	-3.86872	0.16433	3.23330
N	-5.23718	0.55801	0.19259
N	-6.41623	-1.14230	1.39549
N	-5.81589	-1.64489	3.55407
H	-6.47721	-2.40979	3.52868
H	-5.13506	-1.62812	4.30091
O	-6.92516	-0.65133	-0.77355

CC-2

E: -3901.31103
G: -3900.80596

O	-0.90448	3.17355	1.27742
C	0.39080	3.37092	0.69369
H	1.12132	3.00089	1.41508
H	0.58913	4.43206	0.51236
C	0.48855	2.59028	-0.61368
H	-0.26903	2.95120	-1.31093
O	1.79971	2.80941	-1.19564
C	2.47378	1.57378	-1.34181
H	2.35892	1.20620	-2.36623
C	0.32710	1.07769	-0.42572
H	-0.20231	0.83469	0.49452
C	1.77859	0.59674	-0.39494
H	2.19291	0.63355	0.61005
O	1.95482	-0.71545	-0.94706
P	-2.15060	4.16625	0.85449
O	-1.71450	5.58955	1.56470
O	-2.13949	4.45129	-0.62794
O	-3.35801	3.57880	1.54087
C	4.75087	1.13523	-2.04241
C	6.06856	0.95904	-1.78594
C	6.52540	1.33999	-0.48518
N	3.91121	1.68851	-1.11181
C	4.41221	2.08551	0.15827
N	5.73797	1.93716	0.40545
C	1.82424	-1.75877	-0.12549
C	2.02385	-3.06094	-0.90175
C	3.40744	-3.63766	-1.01757
H	3.72407	-3.91751	-0.00344
H	3.32255	-4.54982	-1.61246
C	4.45627	-2.67386	-1.64423
H	5.11207	-3.23755	-2.30941
H	3.96610	-1.89677	-2.24055
C	5.30906	-2.01342	-0.57963
O	6.53226	-2.11194	-0.55762
O	1.50995	-1.71974	1.04476
O	1.02087	-3.58724	-1.35886
O	4.58081	-1.35878	0.31320
O	3.62823	2.55887	0.99555
N	7.81910	1.12988	-0.14415
H	8.01971	1.13692	0.84932
H	8.33314	0.44393	-0.68374
H	6.73002	0.50733	-2.51343
H	4.29532	0.84791	-2.98236
P	4.96578	-1.35007	2.06786
O	3.88772	-0.37334	2.53162
O	6.40587	-0.85812	2.17863

O	4.74326	-2.82460	2.40598
H	-1.74861	5.51207	2.53493
O	-2.12648	-0.86532	-2.64501
P	-1.68492	-0.39928	-1.28281
O	-0.32820	0.50984	-1.56165
O	-1.42070	-1.36594	-0.15695
O	-2.68964	0.75314	-0.67580
C	-3.61509	1.43860	-1.53185
H	-3.38608	1.23561	-2.58032
H	-3.51536	2.51074	-1.34729
C	-5.02861	0.99796	-1.20681
H	-5.72074	1.49171	-1.90520
O	-5.11106	-0.42671	-1.36236
C	-6.06005	-0.97335	-0.45643
H	-6.95949	-1.27193	-1.00092
C	-5.48646	1.29213	0.23240
H	-4.63830	1.27037	0.91099
C	-6.45748	0.12970	0.54179
H	-6.39778	-0.20409	1.57747
O	-6.08673	2.56899	0.36422
H	-6.94591	2.50418	-0.09213
O	-7.80159	0.50577	0.21731
H	-8.14060	1.02130	0.96851
C	-5.94202	-3.40358	-0.38682
C	-4.45781	-2.15825	1.05858
C	-5.40289	-4.57381	0.03122
H	-6.73150	-3.35570	-1.12773
C	-4.35851	-4.48864	1.00591
H	-5.74313	-5.52465	-0.35812
N	-5.51733	-2.20397	0.11574
N	-3.91905	-3.32629	1.49097
N	-3.80112	-5.61997	1.47902
H	-2.98104	-5.55183	2.06738
H	-3.99804	-6.50612	1.03464
O	-4.07307	-1.05294	1.47086

CC-TS-2

E: -4091.06195
G: -4090.52834

O	0.74894	-2.75301	1.84835
C	-0.45326	-3.23630	1.23226
H	-1.28676	-2.75433	1.74493
H	-0.55504	-4.31995	1.34766
C	-0.44623	-2.86626	-0.24909
H	0.39469	-3.35593	-0.74271
O	-1.67614	-3.31850	-0.86806
C	-2.41631	-2.20641	-1.35625
H	-2.22724	-2.06990	-2.42529
C	-0.37943	-1.36028	-0.48582
H	0.11122	-0.83354	0.32880
C	-1.85658	-0.99185	-0.59991
H	-2.31377	-0.88875	0.38066
P	2.08765	-3.71191	1.89038
O	1.66879	-4.83745	3.02030
O	2.23722	-4.49007	0.60553
O	3.18389	-2.81650	2.41175
C	-4.62048	-2.39512	-2.34758
C	-5.97119	-2.45003	-2.27791
C	-6.54484	-2.54205	-0.97000
N	-3.84580	-2.44725	-1.22066
C	-4.45195	-2.51960	0.06119
N	-5.80496	-2.57819	0.13970
O	-3.72864	-2.53356	1.07125
N	-7.88201	-2.61376	-0.83818
H	-8.28989	-2.57072	0.08635
H	-8.48239	-2.46587	-1.63760
H	-6.58537	-2.41850	-3.16850
H	-4.08793	-2.31071	-3.28732
H	1.57360	-4.42539	3.89757
O	2.03149	0.00145	-3.14714
P	1.54648	-0.05065	-1.72294
O	0.26895	-1.10404	-1.73751
O	1.16438	1.19426	-0.96665
O	2.60155	-0.85614	-0.75067
C	3.46951	-1.84980	-1.32122
H	3.25146	-1.97893	-2.38342
H	3.29940	-2.79695	-0.80274
C	4.91152	-1.41881	-1.13465
H	5.56063	-2.13753	-1.65145
O	5.11282	-0.11818	-1.72458
C	5.68692	0.78673	-0.78628
H	6.45785	1.35395	-1.30314
C	5.35733	-1.30663	0.33532
H	4.49697	-1.12695	0.98113
C	6.29301	-0.08201	0.31550
H	6.37169	0.42488	1.28042
O	5.96317	-2.48670	0.82970
H	6.73168	-2.66776	0.25958
O	7.58933	-0.45967	-0.14822
H	8.03436	-0.90914	0.59014
O	-4.20733	0.45359	-0.96984
C	-3.13083	0.89884	-1.32810
C	-2.97655	2.32665	-1.88369
C	-1.64800	3.02321	-1.74471
H	-0.86603	2.32894	-1.43752
H	-1.38974	3.41574	-2.73533
C	-1.73399	4.19798	-0.75985
H	-0.79754	4.76219	-0.79588
H	-2.54257	4.87712	-1.05676
C	-1.98131	3.83174	0.71226
O	-2.00704	4.78577	1.55101
O	-1.96878	0.25597	-1.30334
O	-3.96090	2.83174	-2.39575
O	-3.13462	2.88687	0.70257
P	-3.77165	2.30802	2.11151
O	-3.80523	0.68888	1.80051
O	-5.18441	2.83968	2.21985
O	-2.81265	2.46341	3.26703
H	-4.21409	0.48833	0.93486
C	-0.30750	2.19837	1.95032
H	-1.08615	3.02049	0.98324
O	-0.46800	1.04667	1.65031
O	0.20651	3.01557	2.67461
C	4.06279	1.69427	0.88963
C	4.31638	2.76579	-1.25740
C	3.12254	2.60004	1.25025
H	4.35223	0.89516	1.55584
C	2.78659	3.60419	0.28817
H	2.62231	2.53853	2.20643
N	4.69384	1.76769	-0.32082
N	3.38923	3.68987	-0.89841
N	1.85019	4.53154	0.59002
H	1.49726	5.09818	-0.17144
H	1.20503	4.34166	1.35010
O	4.86354	2.76476	-2.37467

CC-3

E: -3259.25027
G: -3258.74958

O	-0.45244	-3.12250	1.61534
C	-1.70613	-3.10540	0.91918
H	-2.46492	-2.77905	1.63318
H	-1.97694	-4.09940	0.55138
C	-1.61081	-2.12369	-0.24566
H	-0.83922	-2.45963	-0.94033
O	-2.87894	-2.08510	-0.94541
C	-3.38654	-0.76060	-0.96079
H	-3.10541	-0.25758	-1.89135

C	-1.30950	-0.69202	0.20743
H	-0.84130	-0.65879	1.19040
C	-2.69696	-0.04710	0.20791
H	-3.22411	-0.22014	1.14322
P	0.72053	-4.19725	1.17655
O	0.25350	-5.56286	1.97304
O	0.60447	-4.54123	-0.28822
O	1.99384	-3.64827	1.77051
C	-5.52384	-0.09509	-1.89955
C	-6.86335	0.08480	-1.83054
C	-7.52448	-0.43422	-0.67139
N	-4.84208	-0.75688	-0.91296
C	-5.52898	-1.23588	0.23497
N	-6.87227	-1.06786	0.30628
O	-4.88043	-1.79783	1.13442
N	-8.85319	-0.28404	-0.54681
H	-9.32729	-0.65111	0.26724
H	-9.39291	0.18336	-1.26156
H	-7.40597	0.59932	-2.61302
H	-4.92875	0.26487	-2.73052
H	0.36006	-5.45621	2.93514
O	1.39452	1.44916	-1.46276
P	0.94419	0.62613	-0.28417
O	-0.50087	-0.02360	-0.76175
O	0.83622	1.21077	1.10270
O	1.85104	-0.73183	-0.10836
C	2.06050	-1.58110	-1.25310
H	1.57060	-1.16622	-2.13777
H	1.62848	-2.56076	-1.03675
C	3.54216	-1.73175	-1.51990
H	3.67306	-2.45316	-2.33649
O	4.09101	-0.46866	-1.94707
C	5.17624	-0.07240	-1.12056
H	5.95352	0.33506	-1.76313
C	4.37119	-2.18780	-0.30148
H	3.84617	-1.95433	0.62497
C	5.66028	-1.34633	-0.42698
H	6.15862	-1.16662	0.52865
O	4.60053	-3.58453	-0.26670
H	5.07453	-3.81165	-1.08657
O	6.56301	-1.96577	-1.34313
H	6.99470	-2.69505	-0.86752
O	-4.61392	1.93278	0.72355
C	-3.48832	2.21064	0.36640
C	-2.92672	3.65302	0.34077
C	-1.43474	3.77635	0.23264
H	-1.12876	3.40140	-0.75337
H	-1.00099	3.06024	0.94094
C	-0.89180	5.18109	0.49066
H	-1.04907	5.86675	-0.34572
H	-1.39398	5.60690	1.37308
C	0.56685	5.10300	0.83783
O	1.41903	5.90416	0.48218
O	-2.53935	1.35671	-0.01198
O	-3.70769	4.57648	0.46678
C	4.48044	0.85363	1.10167
C	4.52084	2.26995	-0.85277
C	4.03415	1.88605	1.85865
H	4.63790	-0.12671	1.52701
C	3.84261	3.13987	1.19992
H	3.81353	1.75648	2.91024
N	4.76760	1.02039	-0.22321
N	4.07775	3.31003	-0.10111
N	3.44659	4.21853	1.91517
H	3.04602	4.99918	1.39660
H	3.06201	4.05467	2.83719
O	4.74029	2.37190	-2.07200
H	0.84959	4.24536	1.47666

CC-TS-3

E: -3412.09935
G: -3411.55128

O	1.43238	3.38838	-1.09174
C	0.11614	3.71989	-0.63125
H	-0.56468	3.49222	-1.45360
H	0.04176	4.78948	-0.40800
C	-0.27218	2.90398	0.60235
H	0.28482	3.24287	1.47483
O	-1.69238	3.10023	0.85618
C	-2.35693	1.84282	0.78198
H	-2.40727	1.38406	1.77336
C	-0.07756	1.39344	0.43088
H	0.73516	1.14392	-0.24783
C	-1.44523	0.99797	-0.10471
H	-1.54660	1.28850	-1.14536
P	2.76722	3.97191	-0.31686
O	2.64631	5.59247	-0.59131
O	2.65155	3.81226	1.17896
O	3.92726	3.38379	-1.08163
C	-4.72898	1.61143	1.20317
C	-6.03108	1.65704	0.83906
C	-6.31069	2.09477	-0.49579
N	-3.73279	1.98975	0.34034
C	-4.05018	2.43173	-0.97223
N	-5.35317	2.47297	-1.34497
O	-3.12993	2.77408	-1.73390
N	-7.58371	2.14340	-0.92014
H	-7.78348	2.40899	-1.87512
H	-8.33995	1.82316	-0.33177
H	-6.81971	1.35983	1.51804
H	-4.40838	1.28499	2.18529
H	2.79539	5.79320	-1.53242
O	1.60171	-0.16799	3.51596
P	1.34769	-0.23378	2.03198
O	0.12381	0.82647	1.73071
O	1.04817	-1.55808	1.37252
O	2.54662	0.53674	1.20094
C	3.87993	0.56876	1.72046
H	3.88991	0.22353	2.75654
H	4.22551	1.60649	1.69193
C	4.80259	-0.28123	0.86926
H	5.79957	-0.27668	1.33224
O	4.32475	-1.63958	0.83793
C	4.30743	-2.15329	-0.48507
H	4.78290	-3.13149	-0.48597
C	4.92274	0.16924	-0.59466
H	4.01185	0.68072	-0.91313
C	5.09319	-1.16507	-1.34811
H	4.74608	-1.12510	-2.38583
O	6.00802	1.05163	-0.84710
H	6.59508	1.06179	-0.07271
O	6.45423	-1.56716	-1.29312
H	6.96207	-0.73526	-1.36466
O	-2.19422	-0.59192	-2.17180
C	-2.06111	-1.07500	-1.06396
C	-2.28938	-2.58462	-0.79158
C	-1.97094	-3.04639	0.63231
H	-1.85748	-4.13521	0.61537
H	-1.03554	-2.61280	0.99124
C	-3.13566	-2.67419	1.56744
H	-3.24385	-1.59264	1.68561
H	-4.06472	-3.07219	1.13550
C	-2.95948	-3.31052	2.91287
O	-3.02195	-2.71579	3.97779
O	-1.72240	-0.39650	0.03899
O	-3.39010	-3.01686	-1.32588
C	2.19830	-1.49581	-1.64263
C	2.29245	-3.54789	-0.38321
C	0.89199	-1.73401	-1.96695

H	2.69957	-0.59709	-1.97058
C	0.35730	-2.94845	-1.49466
H	0.32430	-1.03104	-2.55924
N	2.91307	-2.39098	-0.92019
N	1.00411	-3.82735	-0.77193
N	-1.01303	-3.25940	-1.77941
H	-1.22532	-4.27639	-1.69367
H	-1.28660	-2.94211	-2.71430
O	2.92658	-4.28304	0.37666
H	-2.76653	-4.40228	2.89470
O	-5.17837	-1.00975	-0.83516
H	-4.59739	-1.78156	-1.03490
H	-5.18016	-0.96776	0.13357
O	-2.54291	-5.58795	-1.55372
H	-2.62576	-5.80445	-2.49804
H	-3.09422	-4.76941	-1.45364

CC-4

E: -3182.76396
G: -3182.28854

O	-0.34962	-2.68601	-0.79636
C	0.99456	-2.58440	-0.31428
H	1.61069	-2.30520	-1.16776
H	1.34927	-3.55198	0.05370
C	1.13739	-1.55897	0.81175
H	0.47403	-1.81463	1.63880
O	2.50934	-1.63011	1.28456
C	3.19571	-0.44044	0.93534
H	3.26281	0.22022	1.80456
C	0.91422	-0.09310	0.40697
H	0.13753	0.00521	-0.34283
C	2.31859	0.25351	-0.11386
H	2.45438	-0.19476	-1.09396
P	-1.40542	-3.74945	-0.11404
O	-0.60061	-5.18142	-0.23134
O	-1.55638	-3.48503	1.36367
O	-2.59643	-3.73611	-1.04293
C	5.58054	-0.21436	1.32091
C	6.88124	-0.40116	0.99767
C	7.14745	-1.14270	-0.19785
N	4.57101	-0.71232	0.53892
C	4.87449	-1.45142	-0.63540
N	6.17600	-1.63891	-0.96764
O	3.94286	-1.89929	-1.32493
N	8.41896	-1.35480	-0.57460
H	8.61364	-1.88731	-1.41166
H	9.19032	-1.01030	-0.02065
H	7.67937	-0.00840	1.61435
H	5.27165	0.33140	2.20437
H	-0.51007	-5.44697	-1.16427
O	-0.89697	1.77923	3.25103
P	-0.92958	1.23762	1.84454
O	0.60221	0.68022	1.56885
O	-1.39140	2.10315	0.70245
O	-1.76933	-0.19030	1.79501
C	-3.07733	-0.17956	2.40098
H	-3.17810	0.68733	3.05901
H	-3.15601	-1.08220	3.01588
C	-4.22436	-0.21992	1.40241
H	-5.15209	-0.15368	1.98878
O	-4.18575	0.88672	0.47996
C	-4.48392	0.45986	-0.84982
H	-5.25820	1.10378	-1.25830
C	-4.27790	-1.48210	0.53749
H	-3.27126	-1.82536	0.31993
C	-4.96506	-0.98839	-0.74829
H	-4.72208	-1.59183	-1.62637
O	-4.92902	-2.57025	1.16364
H	-5.83998	-2.28315	1.35414
O	-6.37896	-0.92605	-0.56647
H	-6.71742	-1.83234	-0.65788
O	3.31791	3.29592	-1.51418
C	2.48147	2.50353	-1.13678
C	1.02530	2.59132	-1.52828
C	0.34098	3.79836	-0.94300
H	0.49417	4.61646	-1.66061
H	-0.72592	3.60239	-0.89801
C	0.89046	4.20154	0.44077
H	1.13689	3.33554	1.05731
H	1.80259	4.80407	0.32744
C	-0.10413	5.04514	1.18590
O	-0.30670	4.96349	2.38662
O	2.79412	1.61317	-0.16476
C	-2.35613	-0.31163	-1.87588
C	-3.01319	2.00023	-2.08973
C	-1.10704	0.00098	-2.32191
H	-2.62369	-1.33276	-1.63948
C	-0.79661	1.38298	-2.38628
H	-0.35070	-0.76008	-2.43990
N	-3.28879	0.66094	-1.69450
N	-1.71946	2.32221	-2.41264
N	0.58205	1.63596	-2.26467
O	-3.92117	2.83995	-2.07447
H	-0.65834	5.78641	0.57597

CC-TS-4

E: -3315.76029
G: -3315.22804

C	0.53419	-1.69041	0.96510
H	-0.22010	-1.92089	1.71837
O	1.84486	-2.00225	1.52029
C	2.74258	-0.93412	1.27103
H	2.94741	-0.37194	2.18084
C	0.56834	-0.17754	0.67700
H	-0.14922	0.10775	-0.08780
C	2.03164	-0.05392	0.24199
H	2.12886	-0.49729	-0.74766
O	-0.96869	1.56438	3.75129
P	-1.08437	1.23492	2.28541
O	0.36456	0.56472	1.88070
O	-1.50411	2.31082	1.31164
O	-2.06497	-0.07601	2.05317
C	-3.43780	0.10178	2.45636
H	-3.55669	1.02462	3.03143
H	-3.69685	-0.73676	3.11062
C	-4.41282	0.08426	1.29554
H	-5.41942	0.18028	1.72693
O	-4.19247	1.18033	0.38603
C	-4.39336	0.76607	-0.96600
H	-5.10782	1.43728	-1.43699
C	-4.36509	-1.17418	0.42752
H	-3.33154	-1.49424	0.30584
C	-4.93438	-0.66371	-0.90746
H	-4.66029	-1.28249	-1.76288
O	-5.06709	-2.26899	0.97953
H	-6.00195	-2.00071	1.03451
O	-6.35564	-0.55100	-0.82413
H	-6.72495	-1.43023	-1.01109
O	3.83691	2.32299	-1.33608
C	2.76985	1.98451	-0.89168
C	1.38030	2.38832	-1.33430
C	0.68948	3.38754	-0.44834
H	0.21415	4.13567	-1.07979
H	-0.11754	2.85806	0.07165
C	1.58659	4.06774	0.60116
H	2.08922	3.34155	1.24306
H	2.33451	4.69939	0.10340
C	0.72273	4.93820	1.46759

O	0.56223	4.77146	2.66659
O	2.71168	1.21278	0.24512
C	-2.29197	-0.11215	-1.99185
C	-2.68430	2.26584	-1.88784
C	-1.00436	0.11121	-2.38253
H	-2.67461	-1.11523	-1.87922
C	-0.54458	1.44875	-2.28287
H	-0.32957	-0.70869	-2.57974
N	-3.11687	0.92524	-1.69264
N	-1.35894	2.47843	-2.15419
N	0.85143	1.56915	-2.18834
O	-3.49771	3.18907	-1.76214
H	0.19973	5.76335	0.94467
N	2.23671	4.30692	-2.90335
H	2.91010	3.76205	-3.43842
H	2.64886	5.23059	-2.77446
H	1.42028	4.43279	-3.50041
O	2.49235	-0.68051	-3.02624
H	3.33704	-0.49518	-2.58259
H	1.94543	0.11491	-2.82601
O	-0.98936	-2.59224	-0.75634
C	0.34872	-2.59540	-0.25663
H	0.98512	-2.27180	-1.08416
H	0.65613	-3.60495	0.02857
P	-2.01826	-3.81631	-0.34799
O	-1.13565	-5.15950	-0.70696
O	-2.22151	-3.84456	1.14598
O	-3.17532	-3.67118	-1.30662
H	-0.98495	-5.22592	-1.66714
C	4.08196	-2.31984	-0.23975
C	5.26377	-2.70208	-0.77813
C	6.44615	-2.12222	-0.21355
N	4.03213	-1.42904	0.79654
C	5.21993	-0.84248	1.30620
N	6.41003	-1.23681	0.78454
O	5.13649	0.00722	2.20993
N	7.64787	-2.47211	-0.70177
H	8.48783	-2.07927	-0.29857
H	7.73009	-3.15303	-1.44346
H	5.31146	-3.41821	-1.58848
H	3.13420	-2.71052	-0.58314

CC-5

E: -3239.84398
G: -3239.31613

O	0.88902	3.15683	-0.39355
C	-0.36098	3.29182	0.29266
H	-1.13233	3.24762	-0.47500
H	-0.42024	4.26533	0.79036
C	-0.62039	2.18267	1.32217
H	-0.04577	2.34747	2.23300
O	-2.03473	2.23050	1.66137
C	-2.68242	1.05418	1.19731
H	-2.80315	0.34625	2.02255
C	-0.36314	0.76676	0.80588
H	0.45720	0.72245	0.09679
C	-1.71320	0.44135	0.17702
H	-1.81624	0.90102	-0.80237
P	2.31523	3.51157	0.35161
O	2.12419	5.09768	0.75133
O	2.43908	2.77459	1.66188
O	3.34727	3.35821	-0.74086
C	-5.07801	0.71601	1.33047
C	-6.33795	0.81263	0.84725
C	-6.51263	1.58131	-0.34819
N	-4.02459	1.33425	0.70826
C	-4.23517	2.06939	-0.48932
N	-5.49615	2.18056	-0.97274
O	-3.25980	2.58964	-1.05727
N	-7.74098	1.71452	-0.87342
H	-7.86963	2.24235	-1.72614
H	-8.54031	1.26722	-0.44687
H	-7.17187	0.33066	1.34094
H	-4.83764	0.15580	2.22606
H	2.11003	5.65370	-0.04842
O	0.83399	-2.12530	3.06385
P	0.98176	-1.30688	1.80568
O	-0.13375	-0.09849	1.92300
O	0.91455	-1.97385	0.45255
O	2.32254	-0.35401	1.89697
C	3.60208	-0.97057	2.11353
H	3.48218	-2.00326	2.45514
H	4.10374	-0.40600	2.90593
C	4.47647	-0.92021	0.87647
H	5.46347	-1.32086	1.14988
O	3.91494	-1.72940	-0.17051
C	4.05314	-1.09435	-1.44385
H	4.56503	-1.77948	-2.11634
C	4.64856	0.47584	0.26756
H	3.72768	1.04429	0.37908
C	4.90007	0.16116	-1.21688
H	4.64553	0.99156	-1.87901
O	5.66188	1.24830	0.88361
H	6.49517	0.75353	0.78809
O	6.25785	-0.23236	-1.42341
H	6.79178	0.57965	-1.43170
O	-3.61984	-0.80707	-1.39610
C	-2.79890	-1.44020	-0.76591
C	-2.71242	-2.95834	-0.87787
C	-1.70285	-3.76071	-0.15950
H	-1.70019	-4.77936	-0.55654
H	-0.72373	-3.29457	-0.33129
C	-1.95884	-3.77528	1.36554
H	-1.95567	-2.75235	1.76020
H	-2.92047	-4.23201	1.61399
C	-0.85088	-4.52001	2.07317
O	-1.01787	-5.11606	3.12239
O	-1.87702	-0.98164	0.05756
C	2.07676	0.33857	-1.96310
C	2.03770	-2.01919	-2.48187
C	0.80186	0.46993	-2.40935
H	2.61442	1.18555	-1.55794
C	0.15322	-0.71019	-2.88423
H	0.29205	1.42291	-2.36711
N	2.72535	-0.86130	-2.02711
N	0.76090	-1.89474	-2.93059
N	-1.11721	-0.63224	-3.34277
O	2.63414	-3.10912	-2.46500
H	0.13997	-4.48953	1.58644
H	-1.61754	-1.49854	-3.49971
H	-1.67484	0.17977	-3.10958
N	-3.58445	-3.46940	-1.67061
H	-3.62166	-4.47409	-1.84644
H	-4.25597	-2.86172	-2.14682

CC-TS-5

E: -3429.14876
G: -3428.60451

O	-1.19191	3.22520	0.63454
C	-0.00492	3.51493	-0.11736
H	0.81105	3.53332	0.60323
H	-0.07944	4.50263	-0.58404
C	0.30447	2.46082	-1.19181
H	-0.28388	2.63081	-2.09272
O	1.71309	2.57819	-1.54461
C	2.40997	1.41844	-1.10518
H	2.53874	0.72224	-1.93865
C	0.12090	1.01550	-0.72929

H	-0.71210	0.88987	-0.04542
C	1.47488	0.75813	-0.08163
H	1.53835	1.23861	0.89170
P	-2.68090	3.51765	-0.00895
O	-2.67560	5.15524	-0.18653
O	-2.78092	2.96199	-1.40688
O	-3.64322	3.10561	1.08055
C	4.80224	1.07924	-1.23447
C	6.05738	1.15711	-0.73508
C	6.23431	1.95420	0.44062
N	3.75366	1.73064	-0.63821
C	3.96492	2.48802	0.54469
N	5.22478	2.59401	1.03482
O	2.99359	3.03515	1.09254
N	7.46182	2.08383	0.97195
H	7.58067	2.59060	1.83883
H	8.24290	1.56117	0.60088
H	6.88385	0.63891	-1.20363
H	4.56223	0.49595	-2.11410
H	-2.68316	5.59497	0.68256
O	-0.93073	-1.77138	-3.19037
P	-1.11785	-1.04198	-1.88471
O	-0.02470	0.19073	-1.89183
O	-1.04989	-1.78507	-0.57421
O	-2.48065	-0.11407	-1.94156
C	-3.72499	-0.71836	-2.32449
H	-3.54929	-1.67622	-2.82334
H	-4.20744	-0.04399	-3.03945
C	-4.65838	-0.89728	-1.14379
H	-5.60981	-1.29453	-1.52584
O	-4.10300	-1.83398	-0.20456
C	-4.29284	-1.39107	1.14054
H	-4.77166	-2.19237	1.69941
C	-4.93370	0.38230	-0.34274
H	-4.05225	1.01992	-0.34094
C	-5.20740	-0.16526	1.06843
H	-5.02405	0.56590	1.85806
O	-5.98662	1.16759	-0.86839
H	-6.79572	0.63157	-0.78480
O	-6.54907	-0.65200	1.15214
H	-7.11665	0.10568	1.37120
O	3.35813	-0.25457	1.59514
C	2.67703	-1.00553	0.92404
C	2.85850	-2.51201	0.96118
C	1.69478	-3.42947	0.65798
H	1.89159	-4.38960	1.14429
H	0.81796	-2.99025	1.15085
C	1.38939	-3.65651	-0.82506
H	1.40292	-2.71866	-1.38696
H	2.14391	-4.30665	-1.28310
C	0.03032	-4.28488	-1.02370
O	-0.34577	-4.71851	-2.10163
O	1.73295	-0.64672	0.05685
C	-2.42569	0.08199	1.88963
C	-2.24541	-2.31149	2.12597
C	-1.18451	0.24045	2.41525
H	-2.99563	0.93787	1.55060
C	-0.49240	-0.94168	2.81783
H	-0.73594	1.22148	2.49858
N	-2.99662	-1.15482	1.79112
N	-1.02099	-2.15858	2.69550
N	0.73330	-0.83884	3.38075
O	-2.74379	-3.42944	1.90813
H	-0.61827	-4.36202	-0.13156
H	1.28056	-1.68441	3.48694
H	1.24682	0.02774	3.28484
N	3.75893	-2.89835	1.86439
H	3.93239	-3.88586	2.01343
H	4.45653	-2.23405	2.18384
C	4.47522	-2.25922	-1.17526
H	3.55260	-2.47681	-0.36433
O	4.02763	-1.96905	-2.28203
O	5.55360	-2.39042	-0.60674

CC-6

E: -3240.61261
G: -3240.07392

O	1.46352	2.84961	-0.82868
C	0.20890	3.22859	-0.24743
H	-0.53378	3.11779	-1.03606
H	0.23412	4.27869	0.06192
C	-0.19722	2.35447	0.94697
H	0.36064	2.62825	1.84178
O	-1.61151	2.59165	1.20321
C	-2.34279	1.40216	0.94089
H	-2.53228	0.87147	1.87872
C	-0.05938	0.85258	0.69494
H	0.77085	0.61535	0.03716
C	-1.41865	0.53335	0.08144
H	-1.45265	0.82720	-0.96507
P	2.88945	3.20074	-0.08138
O	2.86112	4.84670	-0.02157
O	2.87752	2.74339	1.35538
O	3.94198	2.72100	-1.05310
C	-4.74965	1.13182	0.98680
C	-5.98179	1.19366	0.42979
C	-6.08123	1.84062	-0.84242
N	-3.65532	1.67471	0.36628
C	-3.78578	2.25761	-0.92315
N	-5.02094	2.34703	-1.47586
O	-2.76957	2.67451	-1.50342
N	-7.28302	1.95691	-1.43285
H	-7.34710	2.34797	-2.36313
H	-8.09984	1.51964	-1.02940
H	-6.84003	0.75309	0.91934
H	-4.56982	0.64831	1.93927
H	2.92736	5.22573	-0.91657
O	0.81127	-1.69809	3.46097
P	1.05482	-1.13171	2.08488
O	0.07185	0.18433	1.95581
O	0.94288	-2.01548	0.86683
O	2.48456	-0.30722	2.06151
C	3.67902	-0.96144	2.51327
H	3.43841	-1.89173	3.03658
H	4.17365	-0.28987	3.22298
C	4.63830	-1.22804	1.37114
H	5.55936	-1.65309	1.79550
O	4.06475	-2.17218	0.45069
C	4.32755	-1.79623	-0.90334
H	4.80303	-2.63494	-1.40690
C	4.99129	0.00817	0.53342
H	4.13704	0.68015	0.48326
C	5.27860	-0.59785	-0.85037
H	5.13661	0.11128	-1.66822
O	6.06200	0.76732	1.06191
H	6.84901	0.19443	1.02249
O	6.60575	-1.12844	-0.88781
H	7.20189	-0.39295	-1.10682
O	-3.15915	-0.79936	-1.54194
C	-2.58422	-1.41058	-0.65765
C	-2.74997	-2.89603	-0.35677
C	-3.62760	-3.04039	0.90327
H	-3.63448	-4.09561	1.19333
H	-3.16090	-2.47546	1.71682
C	-5.07292	-2.54730	0.68613
H	-5.10054	-1.54066	0.26136
H	-5.58884	-3.23254	-0.00033
C	-5.82277	-2.54998	1.98324
O	-6.32195	-1.56115	2.50121

O	-1.73025	-0.85130	0.21685
C	2.51769	-0.31694	-1.77344
C	2.30237	-2.71851	-1.92049
C	1.28903	-0.16300	-2.32814
H	3.10181	0.54176	-1.46714
C	0.57217	-1.35044	-2.66622
H	0.86251	0.82046	-2.47315
N	3.06174	-1.55874	-1.60711
N	1.07149	-2.57054	-2.47687
N	-0.64951	-1.24659	-3.24194
O	2.79836	-3.83317	-1.68532
H	-5.89633	-3.53323	2.48894
H	-1.22048	-2.08331	-3.26631
H	-1.15807	-0.37904	-3.12446
N	-3.28115	-3.65784	-1.48051
H	-4.08123	-3.15295	-1.86236
H	-2.58312	-3.65441	-2.22398
H	-1.75448	-3.27692	-0.11149

CC-TS-6

E: -3317.02714
G: -3316.46443

O	-1.16237	-1.71041	-2.24603
C	0.08095	-2.37271	-1.99959
H	0.85513	-1.66987	-2.31758
H	0.15972	-3.27903	-2.60839
C	0.29472	-2.72970	-0.52535
H	-0.38730	-3.51861	-0.21167
O	1.65500	-3.22994	-0.39827
C	2.40949	-2.39727	0.45810
H	2.53085	-2.84276	1.44509
C	0.16280	-1.52642	0.41152
H	-0.45428	-0.74590	-0.02651
C	1.62024	-1.08461	0.54623
H	1.86511	-0.45159	-0.30486
P	-2.61156	-2.48264	-2.06562
O	-2.43383	-3.79866	-3.03789
O	-2.76051	-3.02346	-0.66587
O	-3.61601	-1.51442	-2.64019
H	-2.40509	-3.53380	-3.97506
O	-1.05139	-1.20165	4.00535
P	-1.22309	-0.83707	2.55280
O	-0.36311	-1.93719	1.67009
O	-0.93621	0.56689	2.07509
O	-2.71468	-1.32059	2.03643
C	-3.87218	-0.78724	2.69896
H	-3.59194	-0.26240	3.61762
H	-4.51538	-1.62978	2.97237
C	-4.65925	0.13022	1.78686
H	-5.56944	0.44349	2.32112
O	-3.86549	1.27625	1.45333
C	-4.30202	1.81959	0.20779
H	-4.86795	2.73554	0.36857
C	-5.05621	-0.47756	0.43474
H	-4.25526	-1.11749	0.06395
C	-5.20500	0.76658	-0.46652
H	-4.91050	0.56599	-1.49887
O	-6.22195	-1.27835	0.49405
H	-6.95845	-0.66560	0.67424
O	-6.54305	1.26523	-0.42021
H	-7.06744	0.73410	-1.04298
C	3.91350	-1.94226	-1.42210
C	5.08161	-1.46087	-1.91191
C	6.11524	-1.18550	-0.96008
N	3.75711	-2.18713	-0.08610
C	4.79496	-1.87759	0.83251
N	5.97177	-1.40554	0.34697
O	4.58788	-2.03812	2.04781
N	7.30040	-0.70426	-1.39184
H	7.93671	-0.32359	-0.70259
H	7.36885	-0.33862	-2.33277
H	5.22161	-1.28225	-2.97030
H	3.05967	-2.17054	-2.04616
O	2.80284	1.13858	3.01778
C	2.58198	0.69819	1.90496
C	3.02037	1.41278	0.64166
C	1.82428	2.13524	-0.04911
H	1.88666	1.95927	-1.12333
H	0.86015	1.76920	0.30741
C	2.04337	3.61725	0.28065
H	1.57224	4.28294	-0.44583
H	1.65706	3.85092	1.27873
C	3.55979	3.76449	0.26342
O	4.08430	3.71744	-1.01090
O	1.87917	-0.43752	1.79311
H	3.93319	4.60060	0.87210
N	3.99488	2.48180	1.00976
H	3.98927	2.61573	2.02732
H	4.97673	2.25019	0.60956
H	3.53266	0.73613	-0.04043
O	6.02916	2.33000	-0.57971
H	5.07925	3.12415	-0.92979
H	5.81290	1.52242	-1.07395
C	-2.27790	1.23959	-1.03298
C	-1.19083	1.56489	-1.77338
C	-0.97138	2.95766	-2.01567
N	-3.12984	2.20278	-0.56996
C	-2.84974	3.57820	-0.77748
N	-1.76022	3.91087	-1.51935
O	-3.60618	4.43298	-0.28525
N	0.05570	3.33975	-2.80645
H	0.31190	4.31924	-2.81067
H	0.78756	2.67358	-3.01626
H	-0.52478	0.80439	-2.15742
H	-2.51925	0.21718	-0.78091

CC-7

E: -3164.16769
G: -3163.65337

O	-0.00289	2.19852	1.90996
C	1.25529	2.48677	1.28509
H	2.03763	2.18852	1.98946
H	1.36561	3.55440	1.07448
C	1.37480	1.68429	-0.00662
H	0.57466	1.96762	-0.68966
O	2.64358	1.99819	-0.63015
C	3.42593	0.82393	-0.78779
H	3.35792	0.43071	-1.80222
C	1.35756	0.17200	0.23552
H	0.89124	-0.08227	1.18558
C	2.84903	-0.17403	0.21675
H	3.28199	-0.02218	1.20755
P	-1.30182	3.17188	1.60825
O	-0.98750	4.48222	2.55945
O	-1.25542	3.69374	0.19433
O	-2.48829	2.41111	2.14866
H	-1.11223	4.25968	3.49956
O	-1.18921	-1.88685	-1.76606
P	-0.84867	-1.02402	-0.57950
O	0.71615	-0.53592	-0.82726
O	-0.99476	-1.52002	0.83519
O	-1.64835	0.41044	-0.64466
C	-1.82744	1.05337	-1.91708
H	-1.40180	0.44413	-2.71741
H	-1.31713	2.02004	-1.89508
C	-3.30521	1.27824	-2.16883
H	-3.42303	1.72313	-3.16513
O	-3.99830	0.01433	-2.15491

C	-5.05183	0.02311	-1.19803
H	-5.89838	-0.50096	-1.63569
C	-4.00401	2.17729	-1.12909
H	-3.45295	2.17561	-0.18963
C	-5.37666	1.49584	-0.95665
H	-5.84263	1.68987	0.01269
O	-4.08181	3.53595	-1.52116
H	-4.57799	3.55929	-2.35875
O	-6.25679	1.88245	-2.01181
H	-6.57149	2.77865	-1.80496
C	5.17617	1.90334	0.52885
C	6.47212	2.20395	0.78066
C	7.44205	1.69928	-0.14567
N	4.82850	1.15365	-0.56031
C	5.81436	0.63643	-1.43698
N	7.11392	0.95105	-1.20176
O	5.45936	-0.08475	-2.38596
N	8.74144	1.98669	0.04153
H	9.43505	1.61355	-0.59228
H	9.04516	2.52445	0.84094
H	6.76057	2.80354	1.63455
H	4.35900	2.24470	1.15217
N	3.10076	-4.93516	0.79077
O	2.40202	-2.31394	1.74843
C	2.75116	-2.49841	0.59735
C	2.74230	-3.82443	-0.11531
C	1.31008	-4.12349	-0.64511
H	1.16924	-3.78817	-1.67303
H	0.57409	-3.61789	-0.01429
C	1.20397	-5.64671	-0.45259
H	0.21704	-5.98094	-0.11725
H	1.44424	-6.20505	-1.36801
C	2.27392	-5.88828	0.57570
O	3.11524	-1.50104	-0.23998
H	2.37689	-6.83392	1.10666
H	3.46765	-3.79028	-0.93326
C	-4.28750	-0.13617	1.18049
C	-3.86900	-0.87469	2.23593
C	-3.80475	-2.28996	2.05071
N	-4.67154	-0.72706	0.00928
C	-4.56368	-2.13431	-0.14625
N	-4.15306	-2.88353	0.90820
O	-4.86725	-2.63236	-1.24471
N	-3.40550	-3.07514	3.07191
H	-3.21215	-4.05028	2.88359
H	-2.96558	-2.65613	3.88023
H	-3.55511	-0.40219	3.15758
H	-4.33010	0.94010	1.24168

CC-8

E: -3164.6261
G: -3164.09935

O	-0.00631	-2.21793	1.90922
C	-1.25951	-2.51242	1.27792
H	-2.04723	-2.21494	1.97657
H	-1.36521	-3.58085	1.06914
C	-1.37344	-1.71343	-0.01647
H	-0.57351	-2.00135	-0.69780
O	-2.64288	-2.02148	-0.64221
C	-3.42329	-0.84614	-0.79627
H	-3.35625	-0.44968	-1.80960
C	-1.34915	-0.20081	0.22104
H	-0.87672	0.05588	1.16766
C	-2.83956	0.14670	0.20935
H	-3.27357	0.00227	1.20029
P	1.30070	-3.18311	1.61370
O	0.99035	-4.49535	2.56321
O	1.26455	-3.70487	0.19955
O	2.47887	-2.41386	2.16010
H	1.11373	-4.27360	3.50367
O	1.17520	1.86129	-1.80285
P	0.84483	1.00833	-0.60667
O	-0.71583	0.50125	-0.84912
O	0.98005	1.52621	0.80145
O	1.65655	-0.41848	-0.65311
C	1.84386	-1.07228	-1.91957
H	1.42028	-0.47068	-2.72667
H	1.33629	-2.04012	-1.89152
C	3.32370	-1.29363	-2.16126
H	3.44894	-1.74192	-3.15511
O	4.01250	-0.02744	-2.14768
C	5.05948	-0.02925	-1.18356
H	5.90718	0.49628	-1.61719
C	4.01865	-2.18702	-1.11405
H	3.46084	-2.18481	-0.17858
C	5.38767	-1.49995	-0.93440
H	5.84719	-1.68827	0.03919
O	4.10407	-3.54637	-1.50179
H	4.60797	-3.57018	-2.33477
O	6.27664	-1.88788	-1.98156
H	6.59565	-2.78072	-1.76688
C	-5.17368	-1.93889	0.51143
C	-6.47073	-2.23377	0.76391
C	-7.44138	-1.70517	-0.14849
N	-4.82600	-1.17157	-0.56564
C	-5.81268	-0.62556	-1.42397
N	-7.11285	-0.93747	-1.19060
O	-5.45611	0.11695	-2.35580
N	-8.74184	-1.98481	0.04077
H	-9.43400	-1.61466	-0.59636
H	-9.04349	-2.55886	0.81533
H	-6.75982	-2.84733	1.60760
H	-4.35591	-2.29827	1.12373
N	-2.90903	4.91559	0.78106
O	-2.42657	2.31896	1.75121
C	-2.74502	2.46565	0.58896
C	-2.71613	3.78441	-0.14859
C	-1.30884	4.08046	-0.73076
H	-1.24295	3.79317	-1.77835
H	-0.55879	3.52787	-0.16239
C	-1.12057	5.59420	-0.49026
H	-0.10309	5.88227	-0.21294
H	-1.40858	6.20656	-1.35690
C	-2.08257	5.88298	0.59745
O	-3.09627	1.47997	-0.24844
H	-2.15270	6.79136	1.18622
H	-3.50220	3.81744	-0.90602
C	4.27473	0.13454	1.18758
C	3.84726	0.87476	2.23821
C	3.78615	2.28994	2.05080
N	4.66851	0.72348	0.01867
C	4.55910	2.13015	-0.14102
N	4.14175	2.88148	0.90931
O	4.86788	2.62595	-1.23905
N	3.38066	3.07668	3.06780
H	3.19653	4.05371	2.88065
H	2.94308	2.66035	3.87864
H	3.52665	0.40381	3.15834
H	4.31806	-0.94150	1.25159
H	-3.66557	4.93662	1.46449

CC-TS-8

E: -3353.92415
G: -3353.38204

O	-0.05497	-3.41721	1.55593
C	-1.34949	-3.50885	0.94787
H	-2.08010	-3.22099	1.70966
H	-1.57094	-4.52898	0.62067

C	-1.41409	-2.54903	-0.23547
H	-0.66562	-2.83089	-0.97798
O	-2.72824	-2.63983	-0.83805
C	-3.34885	-1.36278	-0.85964
H	-3.21787	-0.86879	-1.82271
C	-1.21264	-1.08929	0.17951
H	-0.70368	-1.00460	1.13789
C	-2.65367	-0.57261	0.24847
H	-3.09240	-0.80023	1.22200
P	1.15603	-4.41964	1.05262
O	0.77765	-5.82708	1.82292
O	1.01574	-4.73184	-0.41723
O	2.41677	-3.82221	1.62543
H	0.88882	-5.73576	2.78611
C	-5.24049	-2.34317	0.33197
C	-6.56720	-2.47652	0.56761
C	-7.44938	-1.72137	-0.27182
N	-4.78394	-1.52440	-0.66281
C	-5.68013	-0.75704	-1.44834
N	-7.01235	-0.90448	-1.23319
O	-5.21889	0.01946	-2.30288
N	-8.77739	-1.81905	-0.08549
H	-9.40676	-1.34448	-0.71885
H	-9.15858	-2.48290	0.57385
H	-6.94283	-3.12828	1.34595
H	-4.48315	-2.87715	0.89215
N	-1.90634	4.01088	1.46438
O	-2.06856	1.31887	2.06092
C	-2.38040	1.66888	0.94040
C	-2.40187	3.10404	0.43415
C	-1.43229	3.36277	-0.75113
H	-1.90075	4.07238	-1.43676
H	-1.19591	2.45225	-1.30016
C	-0.19308	3.99303	-0.08900
H	0.49781	3.22056	0.26260
H	0.34793	4.68214	-0.73960
C	-0.78692	4.66902	1.12247
O	-2.77597	0.82465	-0.03126
H	-0.17983	5.09810	1.91677
H	-3.43431	3.33868	0.15783
H	-2.39570	4.14982	2.33865
C	-1.90043	6.73007	-0.06415
H	-1.16818	5.91756	0.57886
O	-3.07081	6.37369	0.01824
O	-1.22391	7.61337	-0.58041
O	1.30845	1.16799	-1.61233
P	0.93072	0.34017	-0.41210
O	-0.50767	-0.36701	-0.83060
O	0.82122	0.92708	0.97166
O	1.91210	-0.96581	-0.24493
C	2.30487	-1.71476	-1.40999
H	1.79642	-1.33749	-2.30113
H	2.01818	-2.75725	-1.25219
C	3.80461	-1.62736	-1.60065
H	4.07961	-2.26032	-2.45446
O	4.18352	-0.27043	-1.90627
C	5.20139	0.19178	-1.02764
H	5.93298	0.73582	-1.62084
C	4.63525	-2.04636	-0.37206
H	4.05707	-1.90895	0.54255
C	5.82260	-1.06256	-0.41341
H	6.28683	-0.89581	0.56181
O	5.01557	-3.41000	-0.38474
H	5.50198	-3.56211	-1.21446
O	6.79982	-1.50754	-1.35316
H	7.26685	-2.25509	-0.94387
C	4.37679	0.86151	1.24199
C	3.76461	1.75756	2.05389
C	3.34892	2.98997	1.46160
N	4.64316	1.15605	-0.06518
C	4.23642	2.40354	-0.61138
N	3.60375	3.29824	0.19084
O	4.48424	2.63594	-1.80645
N	2.70001	3.90591	2.21663
H	2.22431	4.65885	1.73403
H	2.29688	3.60848	3.09586
H	3.55288	1.52379	3.08913
H	4.68287	-0.10810	1.60685

CC-Pro

E: -3165.38104
G: -3164.84329

O	-0.60223	2.01436	2.01952
C	0.63984	2.45447	1.45524
H	1.42372	2.22103	2.18204
H	0.64177	3.53354	1.27584
C	0.89171	1.70838	0.15079
H	0.07632	1.89953	-0.54551
O	2.12162	2.21623	-0.42810
C	2.98934	1.13763	-0.71973
H	2.86879	0.78539	-1.74525
C	1.07171	0.19313	0.35521
H	0.68401	-0.13151	1.31931
C	2.59022	0.04478	0.26921
H	3.03637	0.23206	1.24948
P	-1.98093	2.86293	1.69352
O	-1.82210	4.17987	2.67433
O	-1.94587	3.41553	0.29088
O	-3.10247	1.98120	2.18562
H	-1.95346	3.92950	3.60641
O	-1.30935	-2.12369	-1.55948
P	-1.04827	-1.17757	-0.41729
O	0.47408	-0.57553	-0.68893
O	-1.16878	-1.60593	1.02091
O	-1.96482	0.17946	-0.56176
C	-2.12321	0.77870	-1.85701
H	-1.59373	0.19892	-2.61674
H	-1.70575	1.78885	-1.82837
C	-3.59446	0.86482	-2.21012
H	-3.68154	1.30165	-3.21309
O	-4.16522	-0.45861	-2.24512
C	-5.27851	-0.55509	-1.36494
H	-6.03803	-1.15747	-1.85787
C	-4.45028	1.69013	-1.22714
H	-3.97303	1.74019	-0.24919
C	-5.76043	0.87943	-1.15455
H	-6.31499	1.02591	-0.22450
O	-4.63104	3.03522	-1.63012
H	-5.09195	3.01003	-2.48757
O	-6.58923	1.18406	-2.27648
H	-7.04774	2.01695	-2.07542
O	2.45158	-2.28201	1.62541
C	3.01209	-2.27642	0.54300
C	3.70576	-3.47704	-0.10397
C	4.58102	-4.22504	0.92884
H	4.48674	-5.30326	0.78134
H	4.25865	-4.00137	1.94752
C	6.02688	-3.74324	0.63112
H	6.51967	-3.33065	1.51547
H	6.63597	-4.57569	0.26645
C	5.85441	-2.68460	-0.46666
O	3.06904	-1.19178	-0.24808
H	6.70511	-2.62578	-1.15105
H	5.72179	-1.68892	-0.01405
C	4.73469	2.37213	0.46545
C	6.03108	2.70372	0.67435
C	6.99050	2.16122	-0.24058
N	4.37668	1.56854	-0.58032
C	5.35532	1.00112	-1.43392

N	6.65302	1.34762	-1.24494
O	4.99404	0.21303	-2.32773
N	8.28876	2.47738	-0.09760
H	8.97610	2.07013	-0.71709
H	8.60116	3.05377	0.67099
H	6.32712	3.35180	1.48933
H	3.92224	2.72931	1.08552
C	-4.66150	-0.64154	1.05694
C	-4.24088	-1.33364	2.14227
C	-4.03112	-2.73669	1.97290
N	-4.91070	-1.26780	-0.13174
C	-4.64883	-2.65627	-0.27260
N	-4.23837	-3.36109	0.81246
O	-4.82177	-3.18171	-1.38657
N	-3.63356	-3.48003	3.02548
H	-3.33845	-4.43339	2.85935
H	-3.29399	-3.02140	3.86010
H	-4.03407	-0.83191	3.07851
H	-4.81609	0.42424	1.10670
N	4.64396	-3.13719	-1.17933
H	4.27179	-2.37266	-1.73939
H	2.89314	-4.10945	-0.47980

AC-1

E: -3934.42945
G: -3933.93680

O	-2.73032	2.27043	-1.29530
C	-1.97103	3.48122	-1.36476
H	-2.67889	4.31463	-1.33939
H	-1.39476	3.53162	-2.29240
C	-1.03002	3.58083	-0.17872
H	-0.53320	4.55370	-0.21591
O	-1.76480	3.52245	1.07369
C	-1.62871	2.25955	1.68107
H	-1.23350	2.37807	2.69322
C	0.02335	2.47056	-0.08501
H	0.27432	2.05368	-1.06094
C	-0.65491	1.43223	0.81466
H	-1.18684	0.70288	0.20905
O	0.27381	0.76953	1.67489
P	-2.68569	1.09993	-2.43423
O	-4.08598	0.92766	-2.99931
O	-1.51028	1.29989	-3.35478
O	-2.41068	-0.16127	-1.40923
C	0.37748	-0.56144	1.59617
C	1.20155	-1.10813	2.77502
C	1.21402	-2.58806	2.91030
H	0.28912	-3.03083	2.54326
H	1.37091	-2.83992	3.96328
C	2.44048	-3.18992	2.11188
O	2.18221	-4.16465	1.36715
O	-0.11583	-1.26798	0.74191
O	1.81786	-0.33934	3.49846
O	3.55565	-2.65134	2.33767
P	-3.21785	-1.61501	-1.52063
O	-4.76847	-1.12423	-1.37888
O	-2.98083	-2.16904	-2.91365
O	-2.83573	-2.41083	-0.30452
O	1.18951	2.96066	0.56897
P	2.52238	3.32838	-0.33449
O	2.56333	4.81130	-0.61245
O	2.62451	2.35828	-1.48931
O	3.66310	3.06529	0.80761
C	3.67417	1.94871	1.71309
H	2.68765	1.81691	2.16045
H	4.37789	2.23522	2.49863
C	4.14980	0.63153	1.12782
H	4.47589	-0.00166	1.96219
O	3.06618	-0.04904	0.45892
C	3.68396	-0.95180	-0.44791
H	4.13754	-1.79022	0.08437
C	5.31190	0.71045	0.10622
H	5.51826	1.74593	-0.17806
C	4.77895	-0.10965	-1.10131
H	4.34669	0.55075	-1.85477
O	6.49035	0.11109	0.63022
H	6.76186	-0.53604	-0.05349
O	5.77067	-0.95979	-1.66053
H	6.27264	-0.44022	-2.31126
N	-2.94109	1.60319	1.77946
C	-3.13694	0.30933	2.22265
C	-4.11957	1.98225	1.17094
C	-4.44651	-0.01423	1.85538
H	-4.23312	2.96328	0.74117
N	-5.05436	1.05615	1.21031
C	-4.86621	-1.32660	2.14421
N	-2.27015	-0.48820	2.86778
C	-2.79617	-1.69315	3.10964
N	-4.02044	-2.14930	2.80170
H	-2.15025	-2.39619	3.62942
N	-6.10480	-1.78508	1.81770
H	-6.57376	-1.30927	1.05633
H	-6.21687	-2.79217	1.82589
C	2.71847	-2.89385	-1.61380
C	1.65267	-0.72361	-1.79266
C	0.62066	-1.27112	-2.48058
H	1.73738	0.33596	-1.58022
C	0.63437	-2.69396	-2.64197
H	-0.19520	-0.66301	-2.84809
N	2.67186	-1.50890	-1.32553
N	1.66087	-3.45471	-2.24890
N	-0.40792	-3.30250	-3.24713
H	-1.32328	-2.83395	-3.23005
H	-0.42070	-4.31395	-3.18816
O	3.72394	-3.54526	-1.27465
H	-4.86621	-0.34634	-1.99104

AC-TS-1

E: -3934.36924
G: -3933.87529

O	-2.99759	-2.78354	1.09725
C	-2.19406	-3.95381	1.05985
H	-2.84688	-4.81085	0.85676
H	-1.68468	-4.13730	2.01306
C	-1.14167	-3.88757	-0.04980
H	-0.72329	-4.88668	-0.19676
O	-1.77254	-3.48753	-1.30787
C	-1.35745	-2.18167	-1.65407
H	-0.67012	-2.19981	-2.50416
C	-0.00549	-2.87895	0.16346
H	0.27901	-2.77285	1.20914
C	-0.66961	-1.63957	-0.39682
H	-1.42131	-1.29897	0.30507
O	0.12794	-0.50614	-0.75246
P	-3.03959	-1.81525	2.48651
O	-3.82441	-2.61094	3.52969
O	-1.58668	-1.50890	2.84548
O	-3.85655	-0.60552	1.92965
C	0.26569	0.43331	0.20914
C	0.14485	1.84652	-0.37779
C	-0.45374	2.90413	0.52301
H	-0.62787	2.51359	1.52261
H	0.26593	3.72649	0.56305
C	-1.74278	3.46888	-0.10361
O	-2.88929	3.07043	0.35438
O	0.48379	0.20422	1.37837
O	0.52654	2.07349	-1.51286
O	-1.64386	4.30527	-1.01286

P	-3.17920	1.31312	1.21206
O	-4.69442	1.40953	0.63228
O	-3.00401	1.82029	2.61764
O	-2.16931	0.64241	0.31727
N	-2.49566	-1.35503	-2.05930
C	-2.33653	-0.16686	-2.75991
C	-3.70102	-1.24964	-1.39957
C	-3.48090	0.58641	-2.48747
H	-4.04031	-2.02524	-0.73301
N	-4.34348	-0.12872	-1.66044
C	-3.46845	1.92005	-2.93342
N	-1.30346	0.20974	-3.52601
C	-1.44986	1.46434	-3.96509
N	-2.44015	2.33185	-3.70517
H	-0.65601	1.83935	-4.60529
N	-4.45587	2.81216	-2.62638
H	-4.96822	2.60768	-1.77494
H	-4.16448	3.78218	-2.68998
H	-4.76265	0.80401	-0.15735
O	1.10957	-3.26266	-0.65107
P	2.64505	-3.19748	-0.04472
O	3.13728	-4.59786	0.22731
O	2.71316	-2.15257	1.04266
O	3.43357	-2.74004	-1.40702
C	3.06967	-1.60967	-2.21206
H	1.98530	-1.47550	-2.22558
H	3.40071	-1.85890	-3.22342
C	3.74244	-0.30688	-1.81730
H	3.72422	0.35140	-2.69993
O	3.04919	0.33593	-0.74004
C	3.89267	1.39199	-0.28453
H	3.71933	2.29917	-0.86390
C	5.18178	-0.40437	-1.29186
H	5.25138	-1.25072	-0.60669
C	5.34780	0.92466	-0.50939
H	5.88528	0.78209	0.43156
O	6.15577	-0.59274	-2.29967
H	6.24490	0.27141	-2.74319
O	5.99640	1.91500	-1.30504
H	6.95401	1.76364	-1.23438
C	3.15778	0.91625	3.29074
C	3.60894	0.71224	2.03098
C	2.90880	2.93895	1.38316
C	2.57021	2.19162	3.56606
H	3.21156	0.14110	4.04432
H	4.01975	-0.23379	1.70552
N	3.53056	1.69901	1.08640
N	2.11443	2.45649	4.80453
H	2.03639	1.71358	5.48552
H	1.58474	3.30429	4.95955
O	2.79918	3.79167	0.48255
N	2.46862	3.15512	2.64743

AC-2

E: -3934.40830
G: -3933.91642

O	-1.70422	3.30390	1.18780
C	-0.33222	3.55620	0.85869
H	0.26119	2.96068	1.55752
H	-0.08492	4.61260	1.00395
C	-0.02163	3.14414	-0.57924
H	-0.56962	3.77167	-1.28069
O	1.39999	3.33981	-0.80332
C	2.02831	2.09597	-1.04382
H	2.22973	1.94905	-2.10921
C	-0.31427	1.66952	-0.86496
H	-1.11210	1.28136	-0.23606
C	1.04053	1.03842	-0.54642
H	1.14446	0.88644	0.53075
O	1.34998	-0.18250	-1.21908
P	-2.89619	4.30263	0.65794
O	-2.54909	5.71695	1.41166
O	-2.78867	4.57571	-0.81891
O	-4.16054	3.68369	1.22965
C	0.84524	-1.31391	-0.71993
C	1.56837	-2.53459	-1.32011
C	3.06170	-2.42772	-1.49255
H	3.44171	-3.32252	-1.98551
H	3.29387	-1.54208	-2.09611
C	3.76221	-2.24434	-0.14128
O	4.81365	-2.81259	0.12049
O	-0.03565	-1.40200	0.10851
O	0.93203	-3.54195	-1.56419
O	3.09881	-1.42480	0.64517
P	3.33593	-1.28794	2.43758
O	2.38450	-0.11612	2.67028
O	4.82299	-0.99909	2.60911
O	2.85828	-2.65766	2.90452
H	-2.61021	5.62399	2.37969
O	-2.41701	0.99704	-3.95169
P	-1.68175	0.36578	-2.79744
O	-0.64677	1.53814	-2.24740
O	-0.95687	-0.94763	-2.96579
O	-2.66706	0.15476	-1.50845
C	-3.77505	1.02465	-1.25232
H	-4.41299	1.08057	-2.13935
H	-3.42246	2.03222	-1.01481
C	-4.56470	0.46489	-0.08566
H	-5.38005	1.16178	0.14015
O	-5.14841	-0.80795	-0.47693
C	-5.02378	-1.76794	0.55019
H	-5.98500	-2.25596	0.71291
C	-3.77349	0.17025	1.20232
H	-2.75211	-0.14035	0.95452
C	-4.55425	-1.01521	1.79626
H	-3.94006	-1.63894	2.45167
O	-3.76849	1.21647	2.15570
H	-3.89861	2.10289	1.72117
O	-5.69539	-0.52123	2.48217
H	-5.41628	0.37161	2.77732
N	3.30585	2.05890	-0.35897
C	4.40426	1.31406	-0.74343
C	3.57556	2.51777	0.91676
C	5.29498	1.39588	0.33092
H	2.86132	3.12626	1.45137
N	4.76571	2.17208	1.35398
C	6.48356	0.64958	0.21467
N	4.60052	0.64498	-1.89069
C	5.77728	0.00805	-1.89178
N	6.71089	-0.03230	-0.92831
H	6.00844	-0.55816	-2.79004
N	7.42513	0.61994	1.19555
H	7.09239	0.80250	2.13508
H	8.10190	-0.13144	1.12583
C	-3.25050	-2.69642	-0.92949
C	-3.92419	-3.93081	1.02664
C	-2.27046	-3.60088	-1.18222
H	-3.43304	-1.83754	-1.55373
C	-2.09742	-4.64677	-0.22912
H	-1.62269	-3.49109	-2.03903
N	-4.04702	-2.82119	0.16512
N	-2.91680	-4.81609	0.81266
N	-1.08656	-5.53568	-0.39445
H	-0.86679	-6.12746	0.39747
H	-0.30234	-5.24055	-0.96423
O	-4.73956	-4.04344	1.96436

AC-TS-2

E: -4124.12997

G: -4123.61242

O	1.38200	-2.63517	1.96400
C	0.25853	-3.15259	1.24382
H	-0.63518	-2.88453	1.81511
H	0.30050	-4.24243	1.15648
C	0.20834	-2.51932	-0.14458
H	1.08222	-2.81875	-0.72354
O	-0.98120	-2.99499	-0.83096
C	-1.86464	-1.91788	-1.07858
H	-1.81374	-1.58702	-2.11952
C	0.10076	-0.99501	-0.09407
H	0.54602	-0.57983	0.80823
C	-1.41204	-0.80002	-0.13726
H	-1.84489	-0.93254	0.85615
O	-1.84491	0.44732	-0.68079
P	2.84728	-3.38678	1.84892
O	2.66977	-4.64731	2.89715
O	3.01205	-4.02800	0.49330
O	3.83246	-2.38289	2.39189
C	-1.87860	1.49033	0.15626
C	-2.53090	2.71999	-0.49724
C	-3.68684	2.49440	-1.42223
H	-3.89738	3.40897	-1.97891
H	-3.49249	1.66505	-2.10801
C	-4.96041	2.11618	-0.60517
O	-6.06437	1.99886	-1.19863
O	-1.43889	1.49812	1.28681
O	-2.13605	3.81747	-0.14120
O	-4.53435	1.03899	0.28148
P	-5.36152	0.69847	1.67755
O	-4.42855	-0.55999	2.16922
O	-6.75919	0.21996	1.36286
O	-5.14441	1.82089	2.66290
H	2.61047	-4.32460	3.81419
O	2.18618	1.21052	-2.44028
P	1.90794	0.66760	-1.06306
O	0.67131	-0.40901	-1.26559
O	1.63088	1.57278	0.10983
O	3.13198	-0.32000	-0.57673
C	3.70342	-1.23927	-1.52449
H	3.32956	-1.03435	-2.53046
H	3.42017	-2.25453	-1.23574
C	5.21413	-1.15399	-1.51451
H	5.59495	-1.94268	-2.17695
O	5.66279	0.12411	-2.02943
C	6.75300	0.61880	-1.27931
H	7.56996	0.89090	-1.94923
C	5.87798	-1.29483	-0.13807
H	5.25512	-0.83852	0.63268
C	7.17947	-0.49531	-0.31649
H	7.57537	-0.10498	0.62127
O	6.08550	-2.63952	0.25025
H	6.71345	-3.01632	-0.39240
O	8.15728	-1.28643	-0.99150
H	8.60788	-1.82458	-0.31966
N	-3.23056	-2.34026	-0.83131
C	-4.35304	-1.83923	-1.46721
C	-3.68182	-3.01844	0.27994
C	-5.43605	-2.28991	-0.70885
H	-2.99430	-3.49058	0.96661
N	-4.99648	-3.03084	0.38528
C	-6.71650	-1.90525	-1.15406
N	-4.41278	-1.08309	-2.57292
C	-5.67699	-0.77645	-2.88519
N	-6.80839	-1.14486	-2.26479
H	-5.80931	-0.15252	-3.76460
N	-7.85890	-2.30848	-0.54070
H	-7.78106	-2.57518	0.43328
H	-8.69148	-1.77938	-0.77297
H	-4.73110	-1.39426	1.75832
C	-5.54857	4.14808	0.98447
H	-5.00941	3.08819	0.21382
O	-4.63618	4.74429	1.50981
O	-6.74261	4.09210	0.80059
C	4.69185	3.37029	0.17517
C	5.05494	2.27405	-0.53881
C	7.35578	2.56813	0.11976
C	5.72713	4.04516	0.89104
H	3.66359	3.70685	0.19837
H	4.37235	1.70466	-1.14746
N	6.34577	1.84649	-0.54982
N	5.42003	5.10884	1.66121
H	4.49773	5.51923	1.61318
H	6.16955	5.66059	2.05658
O	8.53240	2.16711	0.01792
N	7.00723	3.66115	0.84375

AC-3

E: -3292.33148
G: -3291.84306

O	-0.66958	-2.57448	-1.38711
C	0.68234	-2.58322	-0.91209
H	1.30225	-2.15259	-1.70268
H	1.03560	-3.59978	-0.72042
C	0.79565	-1.77083	0.37699
H	0.13187	-2.18715	1.13769
O	2.16007	-1.86701	0.86075
C	2.85628	-0.64685	0.62810
H	3.03986	-0.12381	1.56921
C	0.54346	-0.28056	0.18247
H	-0.24066	-0.07394	-0.54346
C	1.93340	0.18070	-0.27075
H	2.09507	-0.04064	-1.32593
O	2.15715	1.56922	-0.02582
P	-1.58938	-3.92310	-1.13587
O	-0.81954	-5.04330	-2.06622
O	-1.43680	-4.40070	0.28486
O	-2.93540	-3.58282	-1.72878
C	1.61075	2.41080	-0.91531
C	1.58132	3.83964	-0.35531
C	0.76133	4.83663	-1.13123
H	1.30212	5.79232	-1.11032
C	-0.56341	5.09887	-0.43646
O	-1.61791	5.22203	-1.03158
O	1.22703	2.10277	-2.02712
O	2.17575	4.11412	0.67010
H	-0.92546	-4.83572	-3.01195
O	-0.37747	2.26659	2.90207
P	-0.68689	1.66557	1.55913
O	0.25351	0.30964	1.45366
O	-0.59601	2.47581	0.28348
O	-2.19239	1.02450	1.52787
C	-2.71837	0.42578	2.72155
H	-3.07585	1.21567	3.38920
H	-1.95157	-0.15706	3.24470
C	-3.85042	-0.50470	2.33687
H	-4.33593	-0.85064	3.25680
O	-4.83627	0.20178	1.55268
C	-4.99131	-0.41368	0.27838
H	-6.03727	-0.32047	-0.00503
C	-3.42409	-1.73406	1.50736
H	-2.47470	-1.55345	0.99872
C	-4.58061	-1.86696	0.49432
H	-4.30556	-2.40690	-0.41030
O	-3.24189	-2.89517	2.29460
H	-4.14101	-3.17152	2.55459
O	-5.68239	-2.51517	1.14018
H	-5.65565	-3.45019	0.87971

N	4.15419	-0.91925	0.05007
C	5.38166	-0.58299	0.58990
C	4.42010	-1.59304	-1.12763
C	6.32552	-1.07456	-0.31550
H	3.62100	-1.98140	-1.74262
N	5.70334	-1.70824	-1.38531
C	7.67871	-0.84430	0.00503
N	5.63455	0.07865	1.72900
C	6.94987	0.22962	1.92100
N	7.96675	-0.17824	1.14572
H	7.23819	0.75748	2.82606
N	8.69895	-1.29597	-0.76212
H	8.49688	-1.57710	-1.71258
H	9.61857	-0.91082	-0.58757
H	-0.49954	5.24505	0.65477
H	0.59322	4.52930	-2.16246
C	-2.25535	0.53820	-2.09281
C	-3.08006	-0.23254	-1.33905
C	-4.51057	1.66552	-0.92808
C	-2.55244	1.93296	-2.16379
H	-1.38523	0.11408	-2.57701
H	-2.89811	-1.29088	-1.21959
N	-4.18544	0.29753	-0.73660
N	-1.71627	2.76624	-2.83506
H	-0.75267	2.45534	-2.91587
H	-1.80364	3.74847	-2.58440
O	-5.56952	2.09722	-0.43975
N	-3.65566	2.45450	-1.62895

AC-TS-3

E: -3445.17141
G: -3444.63442

O	-0.41050	-3.11625	-1.05033
C	0.22668	-3.67297	0.10733
H	1.26733	-3.83707	-0.17692
H	-0.21535	-4.63985	0.36152
C	0.17907	-2.73436	1.33538
H	-0.53196	-3.09099	2.07890
O	1.47912	-2.70178	1.99868
C	2.22653	-1.58883	1.54766
H	2.47555	-0.93914	2.39068
C	-0.07223	-1.26376	1.01080
H	-0.83467	-1.08581	0.25862
C	1.31745	-0.86742	0.53586
H	1.49510	-1.24449	-0.47001
O	1.49371	0.55040	0.54358
P	-2.04941	-3.26516	-1.20742
O	-2.21479	-4.72940	-1.93948
O	-2.67758	-3.41403	0.15374
O	-2.44068	-2.16394	-2.16559
C	1.84473	1.12657	-0.61159
C	1.90234	2.67559	-0.50485
C	1.42325	3.20668	0.85620
H	0.39024	2.91316	1.07297
C	1.55707	4.70158	0.92792
O	2.37112	5.27743	1.63456
O	2.11393	0.52030	-1.63110
O	2.98258	3.19019	-0.97932
H	-1.97815	-4.66806	-2.88250
O	-1.95991	0.43807	3.89722
P	-1.71162	0.33321	2.41410
O	-0.35304	-0.58193	2.23596
O	-1.59367	1.58550	1.57925
O	-2.77293	-0.71966	1.72606
C	-4.15428	-0.71367	2.10732
H	-4.29489	-0.13921	3.02682
H	-4.44557	-1.75173	2.29838
C	-5.03036	-0.17605	0.99472
H	-6.07616	-0.23663	1.32318
O	-4.70404	1.20448	0.72644
C	-4.62103	1.44593	-0.67166
H	-5.22287	2.31927	-0.91373
C	-4.87348	-0.91204	-0.34559
H	-3.85242	-1.26593	-0.45890
C	-5.16700	0.19238	-1.36621
H	-4.72916	0.00975	-2.35005
O	-5.71659	-2.03939	-0.48203
H	-6.57765	-1.68261	-0.77173
O	-6.58690	0.28262	-1.45479
H	-6.81211	0.71169	-2.29631
H	0.86033	5.29291	0.30697
H	2.08128	2.76011	1.60567
C	-1.04222	1.29397	-1.77498
C	-2.33528	0.91834	-1.52039
C	-2.84015	3.12424	-0.68414
C	-0.72080	2.63009	-1.47429
H	-0.33232	0.59117	-2.18567
H	-2.65721	-0.09858	-1.71633
N	-3.23370	1.80837	-1.03614
N	0.61494	3.10837	-1.66334
H	1.06392	2.83020	-2.56685
H	0.60784	4.13131	-1.62836
O	-3.65347	3.88517	-0.15243
N	-1.55797	3.51161	-0.97993
O	4.68767	3.48420	1.21910
H	4.10192	4.13424	1.64846
H	4.19458	3.29422	0.39125
N	3.48104	-2.04893	0.94539
C	4.46630	-1.23439	0.42668
C	3.89077	-3.34668	0.71994
C	5.42493	-2.10703	-0.09797
H	3.29381	-4.18429	1.04427
N	5.05025	-3.42869	0.09919
C	6.53918	-1.50588	-0.71939
N	4.52013	0.10804	0.42416
C	5.61672	0.55349	-0.19542
N	6.60687	-0.15689	-0.76194
H	5.73128	1.63187	-0.24200
N	7.56085	-2.22800	-1.24022
H	7.40188	-3.20882	-1.43060
H	8.19970	-1.74567	-1.86001
O	2.52297	2.50373	-3.59580
H	2.96079	2.79724	-2.75868
H	2.46865	1.54341	-3.43909

AC-4

E: -3215.83490
G: -3215.37204

O	0.75177	2.56652	-1.15197
C	-0.48088	2.83220	-0.47431
H	-1.27384	2.58761	-1.18464
H	-0.56880	3.89294	-0.21918
C	-0.63328	1.99026	0.79598
H	0.05474	2.33260	1.56830
O	-1.99104	2.16773	1.29609
C	-2.75546	0.99848	1.05899
H	-2.96394	0.46784	1.99168
C	-0.48049	0.48433	0.58485
H	0.28330	0.23216	-0.14699
C	-1.89787	0.13051	0.13034
H	-2.04004	0.46164	-0.90068
P	2.13782	3.34488	-0.71109
O	1.77856	4.91950	-1.02993
O	2.35041	3.27327	0.77964
O	3.17321	2.82877	-1.68290
H	1.69547	5.06816	-1.98904
O	1.13755	-1.30454	3.60386
P	1.17435	-0.94996	2.14055

O	-0.22455	-0.13552	1.84859
O	1.38255	-2.02758	1.10174
O	2.25286	0.27044	1.86654
C	3.54823	0.16985	2.48477
H	3.52673	-0.58043	3.27935
H	3.77009	1.14143	2.93823
C	4.66146	-0.13698	1.49825
H	5.58749	-0.23858	2.08227
O	4.41890	-1.37300	0.79857
C	4.75649	-1.25491	-0.58379
H	5.41310	-2.07704	-0.85621
C	4.87910	0.92160	0.41158
H	3.93074	1.36715	0.12244
C	5.45895	0.09406	-0.75079
H	5.29523	0.55191	-1.72963
O	5.69798	1.99771	0.82676
H	6.55656	1.61782	1.08542
O	6.84960	-0.15174	-0.54654
H	7.32168	0.66302	-0.78827
O	-3.35571	-1.72003	-1.69828
C	-2.41130	-1.90051	-0.97476
C	-1.08793	-2.57216	-1.26705
C	-0.63711	-3.68941	-0.37798
H	-0.06327	-4.43250	-0.93707
H	0.07487	-3.28087	0.36778
C	-1.75780	-4.35970	0.34854
O	-2.91079	-3.95259	0.35243
O	-2.25052	-1.25227	0.24668
C	2.76965	-0.35345	-1.76083
C	3.04002	-2.74449	-1.53590
C	1.49917	-0.53893	-2.21784
H	3.18334	0.64453	-1.67420
C	0.97880	-1.85160	-2.11201
H	0.87648	0.29991	-2.49102
N	3.53052	-1.41633	-1.38981
N	1.72568	-2.91236	-1.89093
N	-0.43017	-1.87261	-2.12329
O	3.78940	-3.69881	-1.29702
H	-1.48276	-5.26897	0.91022
N	-4.03670	1.35308	0.47240
C	-5.19691	0.60960	0.57240
C	-4.27365	2.31450	-0.49146
C	-6.08783	1.20042	-0.32886
H	-3.51300	3.03004	-0.76465
N	-5.49264	2.27109	-0.98401
C	-7.36999	0.62033	-0.41191
N	-5.44273	-0.44810	1.36210
C	-6.68724	-0.90529	1.18853
N	-7.64592	-0.44984	0.36603
H	-6.96332	-1.76612	1.79173
N	-8.34526	1.11477	-1.21157
H	-8.07905	1.74439	-1.95709
H	-9.14987	0.52463	-1.38180

AC-TS-4

E: -3348.84000
G: -3348.32128

O	-1.10928	-2.54669	-1.25414
C	0.13735	-2.93022	-0.66466
H	0.90628	-2.57755	-1.35480
H	0.21363	-4.01951	-0.59009
C	0.34473	-2.31245	0.72213
H	-0.34856	-2.74432	1.44324
O	1.70123	-2.63544	1.15379
C	2.50724	-1.46961	1.14217
H	2.72894	-1.13018	2.15694
C	0.25241	-0.78526	0.75027
H	-0.47255	-0.39797	0.03827
C	1.68751	-0.43007	0.37671
H	1.81028	-0.61497	-0.69009
P	-2.51688	-3.29929	-0.84134
O	-2.20384	-4.87018	-1.22222
O	-2.73164	-3.27856	0.65133
O	-3.53390	-2.71207	-1.79215
H	-2.09938	-4.97721	-2.18488
O	-1.35266	0.83752	3.86512
P	-1.33618	0.62275	2.37405
O	-0.04086	-0.34900	2.08007
O	-1.33455	1.80453	1.43222
O	-2.54090	-0.41376	1.92565
C	-3.85169	-0.19883	2.47776
H	-3.79942	0.50491	3.31244
H	-4.20793	-1.15972	2.86356
C	-4.85301	0.28085	1.44312
H	-5.80539	0.45034	1.96573
O	-4.42368	1.51778	0.84285
C	-4.68571	1.52405	-0.56069
H	-5.25631	2.41504	-0.80946
C	-5.09051	-0.68677	0.27848
H	-4.17113	-1.21269	0.03345
C	-5.48928	0.25844	-0.86877
H	-5.28745	-0.15744	-1.85867
O	-6.04645	-1.69062	0.56086
H	-6.88999	-1.23502	0.73340
O	-6.86546	0.62297	-0.75756
H	-7.38608	-0.10300	-1.13996
O	3.48628	1.38944	-1.06092
C	2.48619	1.63566	-0.43549
C	1.28046	2.47282	-0.81313
C	0.82906	3.52231	0.15388
H	0.31209	4.33631	-0.35213
H	0.08614	3.06829	0.83876
C	1.95452	4.05161	0.98164
O	3.03742	3.49427	1.09187
O	2.09078	0.90894	0.68310
C	-2.69442	0.55251	-1.67823
C	-2.80939	2.93496	-1.28867
C	-1.38832	0.67309	-2.04583
H	-3.18732	-0.41200	-1.68440
C	-0.78246	1.93355	-1.80944
H	-0.80943	-0.18916	-2.34174
N	-3.40268	1.64175	-1.27632
N	-1.46668	3.02779	-1.54784
N	0.61978	1.86691	-1.74561
O	-3.50339	3.92449	-1.02506
H	1.74982	4.98694	1.53024
N	3.78041	-1.73463	0.49245
C	4.96411	-1.07724	0.77465
C	3.96127	-2.34005	-0.73867
C	5.81207	-1.37145	-0.29679
H	3.17349	-2.91934	-1.19641
N	5.16765	-2.17044	-1.23355
C	7.10437	-0.81151	-0.24562
N	5.26001	-0.31797	1.84116
C	6.50878	0.15543	1.77022
N	7.43028	-0.03704	0.81311
H	6.82353	0.78523	2.59804
N	8.04023	-1.04565	-1.19616
H	7.73602	-1.41616	-2.08686
H	8.85287	-0.44224	-1.20735
O	1.85994	-0.45660	-3.04835
H	1.43329	0.35524	-2.69000
H	2.75419	-0.41614	-2.67097
N	2.89551	4.24940	-1.97220
H	3.21614	5.12831	-1.56762
H	2.75189	4.41870	-2.96692
H	3.67256	3.59540	-1.89954

AC-5

E: -3272.91130
G: -3272.39844

O	-1.23676	-2.95791	-0.95105
C	0.02047	-3.24248	-0.32994
H	0.77460	-3.05057	-1.09636
H	0.07961	-4.29768	-0.04444
C	0.30678	-2.36356	0.89619
H	-0.26204	-2.69550	1.76264
O	1.72190	-2.49017	1.20892
C	2.40266	-1.28489	0.89289
H	2.64778	-0.72039	1.79646
C	0.07069	-0.87802	0.64607
H	-0.76923	-0.69349	-0.01579
C	1.41013	-0.48359	0.03266
H	1.46888	-0.76280	-1.02084
P	-2.65881	-3.39325	-0.23464
O	-2.53093	-5.03273	-0.16507
O	-2.70271	-2.92686	1.19833
O	-3.71240	-2.97924	-1.23407
H	-2.57331	-5.42064	-1.05777
O	-0.85424	1.70643	3.35200
P	-1.13862	1.05942	2.02035
O	-0.08869	-0.20699	1.89719
O	-1.13462	1.89521	0.76167
O	-2.51229	0.15611	2.08903
C	-3.75493	0.77212	2.46339
H	-3.57997	1.74153	2.94039
H	-4.23186	0.11287	3.19542
C	-4.69561	0.92456	1.28451
H	-5.66376	1.27205	1.67338
O	-4.18384	1.89820	0.35902
C	-4.39867	1.48732	-0.99388
H	-4.94720	2.27308	-1.50884
C	-4.90951	-0.35207	0.46085
H	-3.98880	-0.93187	0.43095
C	-5.23660	0.20699	-0.93441
H	-5.02375	-0.49819	-1.73988
O	-5.90184	-1.21246	0.98680
H	-6.74190	-0.72033	0.95599
O	-6.60433	0.61850	-0.99339
H	-7.13418	-0.17603	-1.17285
O	3.24628	1.07954	-1.40090
C	2.46026	1.55430	-0.60971
C	2.32220	3.05429	-0.39252
C	1.46585	3.59977	0.67902
H	1.40281	4.69180	0.62768
H	0.44487	3.18141	0.56185
C	1.91380	3.19002	2.07106
O	2.86852	2.47601	2.30605
O	1.59165	0.92905	0.15818
C	-2.46511	0.17528	-1.86987
C	-2.44187	2.58766	-1.96777
C	-1.21695	0.13097	-2.40026
H	-2.98537	-0.73372	-1.59637
C	-0.58832	1.37914	-2.69156
H	-0.71176	-0.81230	-2.55884
N	-3.10863	1.36477	-1.68485
N	-1.19224	2.55086	-2.50057
N	0.65815	1.38671	-3.21649
O	-3.03286	3.65395	-1.72797
H	1.27980	3.60269	2.87232
N	3.65532	-1.57311	0.23141
C	4.86026	-0.94229	0.48286
C	3.83555	-2.30269	-0.92972
C	5.71265	-1.36193	-0.54089
H	3.03290	-2.89142	-1.34957
N	5.05362	-2.21976	-1.41454
C	7.02475	-0.84687	-0.51377
N	5.16438	-0.09962	1.48120
C	6.43076	0.32264	1.39282
N	7.36045	0.01027	0.47667
H	6.75326	1.01545	2.16553
N	7.96657	-1.20025	-1.41823
H	7.67085	-1.65540	-2.27153
H	8.80893	-0.64099	-1.46277
H	1.21863	0.54576	-3.16574
H	1.15636	2.26783	-3.24829
N	2.97368	3.78538	-1.22028
H	2.94208	4.80467	-1.16884
H	3.53618	3.34929	-1.95608

AC-TS-5

E: -3462.21600
G: -3461.68538

O	-1.73033	3.05598	0.78914
C	-0.50309	3.42234	0.15013
H	0.26291	3.36571	0.92713
H	-0.54902	4.45473	-0.21108
C	-0.12581	2.49146	-1.01077
H	-0.72970	2.69976	-1.89242
O	1.26746	2.74475	-1.34524
C	2.06786	1.63714	-0.95905
H	2.35752	1.03701	-1.82552
C	-0.19828	1.00708	-0.66493
H	-1.01916	0.77016	0.00465
C	1.16915	0.80752	-0.02563
H	1.18443	1.20632	0.98982
P	-3.18412	3.35973	0.06837
O	-3.18169	5.00196	-0.04608
O	-3.19853	2.85330	-1.35147
O	-4.20081	2.89559	1.08400
H	-3.23093	5.40923	0.83758
O	-0.96183	-1.74460	-3.25903
P	-1.25328	-1.07198	-1.94257
O	-0.27188	0.25187	-1.87723
O	-1.16992	-1.85116	-0.65258
O	-2.68065	-0.25055	-2.02268
C	-3.86832	-0.94124	-2.43921
H	-3.61547	-1.89363	-2.91483
H	-4.36786	-0.31098	-3.18209
C	-4.82843	-1.16102	-1.28750
H	-5.74339	-1.61512	-1.69525
O	-4.25131	-2.05312	-0.31989
C	-4.53926	-1.62376	1.01369
H	-5.01337	-2.44648	1.54435
C	-5.19642	0.10804	-0.50949
H	-4.34138	0.78013	-0.47140
C	-5.50312	-0.44044	0.89474
H	-5.38180	0.30680	1.68222
O	-6.24990	0.85192	-1.09199
H	-7.01953	0.25871	-1.15134
O	-6.81987	-0.99228	0.94132
H	-7.43874	-0.24408	0.98812
O	3.07170	-0.25205	1.63885
C	2.42782	-0.97023	0.90038
C	2.58050	-2.47705	0.89519
C	1.51141	-3.37372	0.33237
H	1.72163	-4.41175	0.60208
H	0.55807	-3.08915	0.80203
O	1.51980	-0.57973	0.01024
C	-2.75731	-0.09152	1.85063
C	-2.51637	-2.48257	2.08959
C	-1.53388	0.09850	2.40597
H	-3.34990	0.74927	1.51269
C	-0.80289	-1.06686	2.78817
H	-1.12046	1.09209	2.51579
N	-3.28632	-1.34419	1.72574
N	-1.29170	-2.29874	2.65049

N	0.42069	-0.92972	3.34697
O	-2.99810	-3.61096	1.89705
H	0.99664	-1.75696	3.44385
H	0.90230	-0.04357	3.26680
N	3.36011	-2.92761	1.86117
H	3.50001	-3.92466	1.98768
H	4.00185	-2.28670	2.31935
C	4.55344	-2.61840	-0.97469
H	3.51961	-2.38050	-0.38526
O	4.39745	-2.63516	-2.19796
O	5.46913	-2.79948	-0.17041
C	1.29796	-3.29991	-1.17267
H	1.54266	-2.35106	-1.67142
O	0.88184	-4.25642	-1.79796
N	3.29470	2.09713	-0.34689
C	4.53109	1.50432	-0.52128
C	3.42182	2.94139	0.74096
C	5.34901	2.06124	0.46405
H	2.58387	3.52383	1.09589
N	4.63657	2.96852	1.24168
C	6.67920	1.59566	0.50548
N	4.88339	0.58180	-1.42748
C	6.15953	0.21045	-1.27571
N	7.06081	0.65182	-0.38494
H	6.51629	-0.54819	-1.96626
N	7.59568	2.07503	1.37870
H	7.26991	2.59722	2.18120
H	8.45207	1.54793	1.49343

AC-6

E: -3273.68692
G: -3273.16252

O	-1.52063	-2.60566	-1.42385
C	-0.24954	-3.04824	-0.93590
H	0.48393	-2.71981	-1.67630
H	-0.21750	-4.14126	-0.88231
C	0.11336	-2.45919	0.43324
H	-0.46643	-2.92527	1.22849
O	1.52278	-2.75067	0.66071
C	2.25716	-1.53467	0.67445
H	2.47031	-1.21714	1.69841
C	-0.02399	-0.93723	0.50235
H	-0.84191	-0.56872	-0.10985
C	1.34358	-0.51625	-0.01232
H	1.38860	-0.62618	-1.09588
P	-2.92214	-3.19263	-0.77899
O	-2.80667	-4.80500	-1.09163
O	-2.91627	-3.07193	0.72391
O	-4.00525	-2.56085	-1.62016
H	-2.86861	-4.97668	-2.04825
O	-0.95459	1.06814	3.66275
P	-1.18779	0.72238	2.21441
O	-0.17359	-0.53210	1.86671
O	-1.09050	1.78835	1.14827
O	-2.59149	-0.12727	2.05546
C	-3.79000	0.33312	2.69489
H	-3.57575	1.16696	3.36950
H	-4.18980	-0.49526	3.28936
C	-4.83041	0.73187	1.66891
H	-5.75725	0.99341	2.19943
O	-4.36698	1.87354	0.92985
C	-4.67402	1.74814	-0.46064
H	-5.25152	2.61693	-0.76821
C	-5.12844	-0.34915	0.62119
H	-4.22941	-0.92899	0.42355
C	-5.51145	0.47602	-0.61918
H	-5.32927	-0.05455	-1.55641
O	-6.11479	-1.28009	1.02501
H	-6.93297	-0.77415	1.17655
O	-6.87855	0.88544	-0.54370
H	-7.42003	0.11714	-0.79157
O	2.72373	1.14688	-1.64165
C	2.38761	1.55068	-0.54366
C	2.65344	2.94510	0.01671
C	3.60166	2.85685	1.22318
H	3.70749	3.84957	1.67970
H	3.18892	2.20730	2.00598
C	4.98241	2.35562	0.91101
O	5.35808	1.98966	-0.19275
O	1.69402	0.81349	0.35093
C	-2.75525	0.66795	-1.62700
C	-2.81986	3.06782	-1.35347
C	-1.53697	0.75671	-2.21773
H	-3.23333	-0.29044	-1.47283
C	-0.97724	2.06270	-2.36154
H	-1.00911	-0.12938	-2.54470
N	-3.43048	1.78952	-1.23623
N	-1.60697	3.16413	-1.95583
N	0.22164	2.21130	-2.97311
O	-3.42363	4.05868	-0.90831
H	5.67768	2.33724	1.77076
N	3.53723	-1.68493	0.02169
C	4.74467	-1.24600	0.53455
C	3.76834	-1.98120	-1.30981
C	5.64727	-1.33253	-0.52760
H	2.97302	-2.34214	-1.94655
N	5.01744	-1.80234	-1.67520
C	6.97078	-0.92821	-0.25696
N	5.01227	-0.82705	1.78026
C	6.29810	-0.47810	1.91058
N	7.27454	-0.50603	0.99123
H	6.59559	-0.12805	2.89533
N	7.95521	-0.98225	-1.18326
H	7.69692	-1.07487	-2.15674
H	8.81374	-0.48518	-0.98305
H	0.84721	1.41535	-2.99679
H	0.67408	3.11197	-2.87109
N	3.14638	3.89227	-0.97413
H	3.96075	3.47627	-1.42430
H	2.44372	3.98561	-1.70641
H	1.68640	3.29578	0.39126

AC-TS-6

E: -3539.40148
G: -3538.83473

O	-2.92106	-3.14923	-1.03365
C	-1.54372	-3.55730	-0.97894
H	-1.15057	-3.49629	-1.99750
H	-1.45338	-4.59168	-0.63452
C	-0.77714	-2.63670	-0.04510
H	-1.30651	-2.54575	0.90192
O	0.50932	-3.25581	0.18783
C	1.45482	-2.23761	0.43598
H	1.44125	-1.90483	1.47782
C	-0.49056	-1.22465	-0.61954
H	-0.77331	-1.18196	-1.67190
C	1.04840	-1.09709	-0.49931
H	1.51030	-1.23538	-1.47754
P	-3.97049	-3.88164	0.00249
O	-4.01931	-5.41849	-0.56941
O	-3.39962	-3.96694	1.39171
O	-5.29768	-3.18587	-0.24735
H	-4.41330	-5.44580	-1.46015
O	1.35688	1.13088	-1.94188
C	1.63981	1.17849	-0.75512
C	2.01054	2.43832	0.00799
C	3.13220	2.20724	1.02145
H	3.32131	3.13552	1.57163

H	2.78738	1.46367	1.74754
C	4.42482	1.68233	0.38253
O	4.29444	0.84278	-0.61961
O	1.49141	0.12377	0.07458
H	5.16362	1.42003	1.16701
N	2.77866	-2.75133	0.14749
C	3.95810	-2.29298	0.70317
C	3.13601	-3.46414	-0.98353
C	4.96959	-2.79855	-0.11955
H	2.38484	-3.91527	-1.61496
N	4.43483	-3.53779	-1.16748
C	6.28827	-2.43279	0.21156
N	4.12458	-1.53052	1.79514
C	5.41330	-1.24578	2.00049
N	6.48455	-1.63898	1.28926
H	5.63170	-0.61784	2.85963
N	7.36873	-2.86766	-0.48201
H	7.20996	-3.22907	-1.41411
H	8.22708	-2.34509	-0.35381
N	2.26692	3.57600	-0.88134
H	3.22307	3.51629	-1.23162
H	1.68798	3.45179	-1.71320
H	1.10510	2.65038	0.58740
C	5.85653	3.50893	-0.89940
H	4.99120	2.71620	-0.01415
O	5.52191	3.18438	-2.01143
O	6.56399	4.18047	-0.19195
O	6.95497	0.49813	-0.86808
H	7.11405	0.03423	-0.02780
H	5.95802	0.54926	-0.88910
O	-0.97671	-0.93281	2.43935
P	-1.28308	0.26672	1.57397
O	-1.21520	-0.15482	-0.02876
O	-0.56204	1.57103	1.80155
O	-2.86718	0.66812	1.61293
C	-3.89570	-0.32262	1.48782
H	-4.35936	-0.44623	2.47155
H	-3.49022	-1.28896	1.18083
C	-4.94335	0.14809	0.48403
H	-5.85304	-0.44283	0.63729
O	-5.26253	1.53310	0.75232
C	-4.99730	2.33872	-0.38639
H	-5.75601	3.11862	-0.41410
C	-4.55028	0.06915	-1.01049
H	-3.46184	0.02480	-1.11511
C	-5.11929	1.38768	-1.57333
H	-4.61085	1.73604	-2.47744
O	-5.15787	-0.99576	-1.71968
H	-5.18367	-1.81039	-1.14458
O	-6.50633	1.21527	-1.83087
H	-6.59388	0.25197	-1.99237
C	-1.42274	3.33766	-0.91799
C	-2.60644	2.69423	-1.05569
C	-3.57190	4.01652	0.70957
C	-1.34176	4.34422	0.09465
H	-0.57779	3.06505	-1.53422
H	-2.72610	1.91156	-1.78793
N	-3.69164	3.02117	-0.29281
N	-0.19640	5.03274	0.27028
H	0.67719	4.72652	-0.16243
H	-0.13545	5.64782	1.07155
O	-4.57231	4.27917	1.40196
N	-2.38442	4.65557	0.87029

AC-Hsr

E: -3274.89375
G: -3274.34535

O	-1.52780	-2.81564	-1.12948
C	-0.28408	-3.21878	-0.54611
H	0.47780	-2.99739	-1.29659
H	-0.27898	-4.29764	-0.35966
C	0.04978	-2.47463	0.75423
H	-0.54501	-2.84917	1.58529
O	1.45234	-2.72553	1.05138
C	2.20600	-1.53611	0.85828
H	2.47276	-1.08257	1.81621
C	-0.08507	-0.95878	0.64586
H	-0.90934	-0.65747	0.00677
C	1.27875	-0.59801	0.06945
H	1.31614	-0.83123	-0.99587
P	-2.96261	-3.26293	-0.44784
O	-2.90240	-4.90535	-0.54476
O	-2.97817	-2.94401	1.02571
O	-4.00455	-2.70932	-1.39017
H	-2.95980	-5.19824	-1.47198
O	-0.94089	1.35731	3.61253
P	-1.19751	0.89760	2.20010
O	-0.21616	-0.40558	1.95945
O	-1.09683	1.87152	1.05136
O	-2.62525	0.07325	2.12586
C	-3.82825	0.70780	2.58367
H	-3.59930	1.61331	3.15393
H	-4.33519	0.00381	3.25175
C	-4.76619	1.02704	1.43725
H	-5.70902	1.39864	1.86427
O	-4.19441	2.04347	0.59645
C	-4.45090	1.77226	-0.78362
H	-4.96122	2.62973	-1.21663
C	-5.06245	-0.15667	0.50748
H	-4.18002	-0.78759	0.42131
C	-5.35555	0.53728	-0.83293
H	-5.17342	-0.10522	-1.69664
O	-6.10611	-0.99487	0.96542
H	-6.91416	-0.45092	0.97072
O	-6.70108	1.01930	-0.85706
H	-7.26800	0.27240	-1.11290
O	3.04446	0.79407	-1.45093
C	2.39890	1.39041	-0.60740
C	2.35923	2.90517	-0.43046
C	2.60824	3.31660	1.03355
H	2.70477	4.40754	1.05129
H	1.72984	3.05531	1.63206
C	3.82371	2.67433	1.68693
O	5.00488	2.98444	0.93070
O	1.57070	0.78067	0.26521
C	-2.58045	0.44511	-1.75811
C	-2.47668	2.85815	-1.73989
C	-1.35005	0.38415	-2.32677
H	-3.12731	-0.45570	-1.51196
C	-0.69566	1.62454	-2.59731
H	-0.88152	-0.56666	-2.54293
N	-3.17908	1.64574	-1.50303
N	-1.24953	2.80437	-2.32010
N	0.51638	1.61990	-3.19817
O	-3.01681	3.93004	-1.41927
H	3.69639	1.58788	1.74518
N	3.45188	-1.83322	0.18311
C	4.67352	-1.26684	0.49439
C	3.60425	-2.41332	-1.06272
C	5.50682	-1.56954	-0.58511
H	2.78258	-2.92287	-1.54488
N	4.82013	-2.29982	-1.54861
C	6.82535	-1.07517	-0.51582
N	5.01030	-0.57431	1.59253
C	6.27856	-0.15280	1.53573
N	7.18724	-0.35162	0.56791
H	6.62559	0.42217	2.39095
N	7.74976	-1.32039	-1.47360
H	7.43158	-1.64919	-2.37571

H	8.59224	-0.75933	-1.46296
H	1.06524	0.76994	-3.18104
H	1.03588	2.48955	-3.21177
N	3.24914	3.60707	-1.35021
H	4.20320	3.35752	-1.09012
H	3.10455	3.22385	-2.28444
H	1.32481	3.17991	-0.67189
H	5.70534	2.38137	1.22742
H	3.92002	3.05083	2.71556

H_2O

E: -76.42067
G: -76.41058

O	0.00000	0.00000	0.12100
H	0.00000	0.75903	-0.48400
H	0.00000	-0.75903	-0.48400

NH_3

E: -56.55397
G: -56.53462

N	0.00000	0.00000	0.12394
H	0.00000	0.93338	-0.28920
H	-0.80833	-0.46669	-0.28920
H	0.80833	-0.46669	-0.28920

PO_4H^{2-}-H_2O

E: -719.66177
G: -719.64134

P	-0.48284	0.11896	0.03045
O	-1.32631	-1.24243	-0.51814
O	0.34532	-0.37228	1.24169
O	-1.53559	1.17331	0.39912
O	0.36356	0.47287	-1.21197
O	2.76680	-0.05109	0.00386
H	2.09725	-0.26465	0.69679
H	2.10883	0.22052	-0.67961
H	-1.87364	-1.58332	0.20964

H_2CO_2

E: -189.76302
G: -189.75036

C	0.00000	0.42206	0.00000
H	-0.36054	1.45816	0.00000
O	1.16994	0.09534	0.00000
O	-1.03882	-0.42641	0.00000
H	-0.68837	-1.34201	0.00000

HCO_2^-

E: -189.29221
G: -189.29234

C	0.00000	0.33544	0.00000
H	-0.00070	1.45876	0.00000
O	1.13354	-0.21647	0.00000
O	-1.13345	-0.21746	0.00000

CO_2

E: -188.57488
G: -188.58114

C	0.00000	0.00000	0.00000
O	0.00000	0.00000	1.16963
O	0.00000	0.00000	-1.16963
